# A Mild and Regioselective Route to Fluoroalkyl Aromatic Compounds via Directed Cycloaddition Reactions

**DOI:** 10.1021/acs.joc.2c00800

**Published:** 2022-07-08

**Authors:** David L. Cousins, Yee Hwee Lim, Joseph P. A. Harrity

**Affiliations:** † Department of Chemistry, University of Sheffield, Sheffield S3 7HF, U.K.; ‡ Organic and Biomolecular Chemistry, Institute of Sustainability for Chemicals, Energy and Environment, A*STAR, 8 Biomedical Grove, Neuros, #07-01, Singapore 138665, Singapore

## Abstract

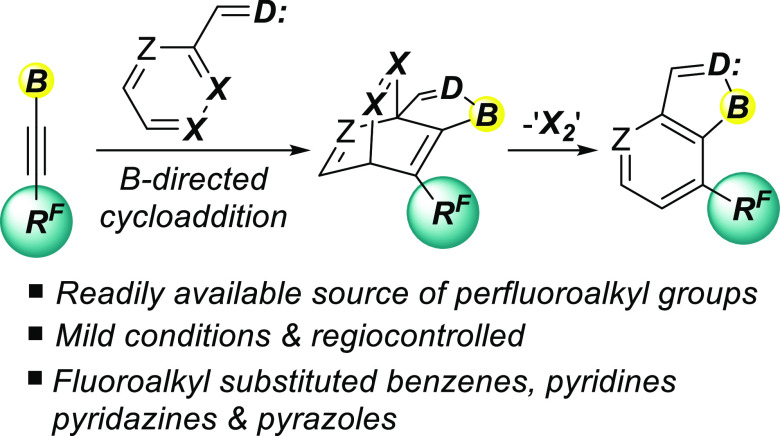

The synthesis of perfluoroalkyl-substituted (hetero)arenes by benzannulation strategies is complementary to ring functionalization approaches as it obviates the need for pre-existing functionality and innate regiocontrol. We report a mild and regiospecific boron-directed benzannulation method as a vehicle for accessing a range of perfluoroalkyl-substituted (hetero)aromatic building blocks that can be readily elaborated through established C–B bond functionalization processes.

## Introduction

**Figure 1:**
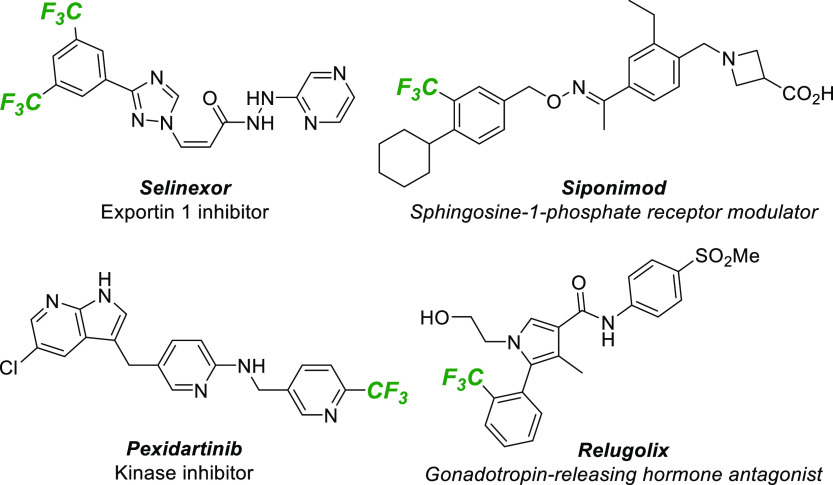
Prominent bioactive trifluoromethylated aromatic compounds.

Organofluorine compounds are widely established high-value materials because of their unique chemical and physical properties.^1^ For example, perfluoroalkyl chains can impart impressive thermal and chemical stability, and for this reason, such compounds have found application in numerous fields of material science. Of particular importance are CF_3_-substituted (hetero)aromatic compounds, and these are ubiquitous among marketed medicines because of their favorable physicochemical properties (Figure 1).^2^

**Scheme 1:**
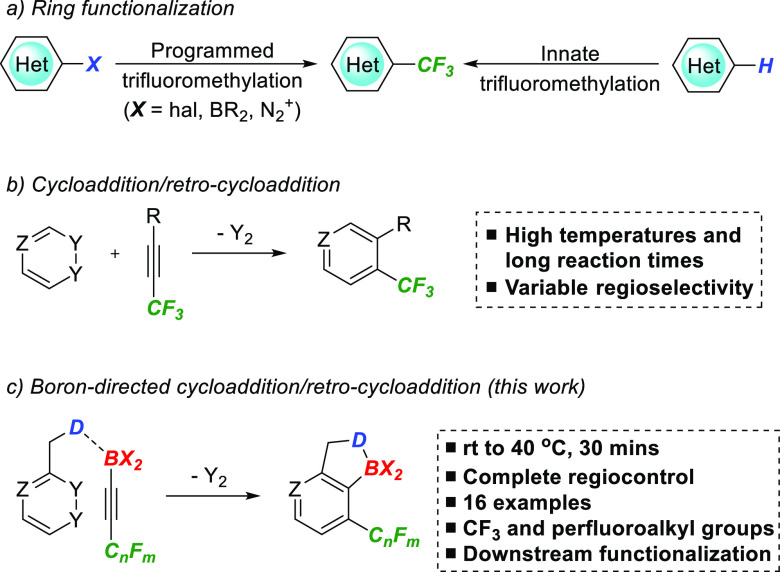
Strategies for the Synthesis of Fluoroalkyl-Substituted Arenes

Broadly speaking, there are three general approaches to incorporating trifluoromethyl groups into (hetero)aromatic compounds. “Programmed trifluoromethylation” is a popular approach that exploits a pre-existing functional handle, such as a (pseudo)halide or boronate, to deliver the CF_3_ group to a precise location on the substrate.^3^ An alternative strategy is “innate trifluoromethylation” of a C–H group, typically through the reaction of the parent (hetero)arene with a trifluoromethyl radical.^4^ A final strategy that has received relatively little recent attention is (hetero)benzannulation using one or more CF_3_-substituted precursors. Specifically, cycloaddition reactions of this type are complementary to the two strategies outlined earlier because the final position of the CF_3_ is dictated by neither the presence of existing functional groups nor by the innate preference of the parent (hetero)arene. However, a drawback is that these reactions typically require harsh conditions and deliver products with poor regiocontrol.^5^ We report herein that boron-directed cycloadditions^6^ allow rapid and regiocontrolled synthesis of fluoroalkyl-substituted (hetero)arenes under mild conditions to deliver products that can be further elaborated through the C–B bond (Scheme 1).

## Results and Discussion

**Scheme 2:**
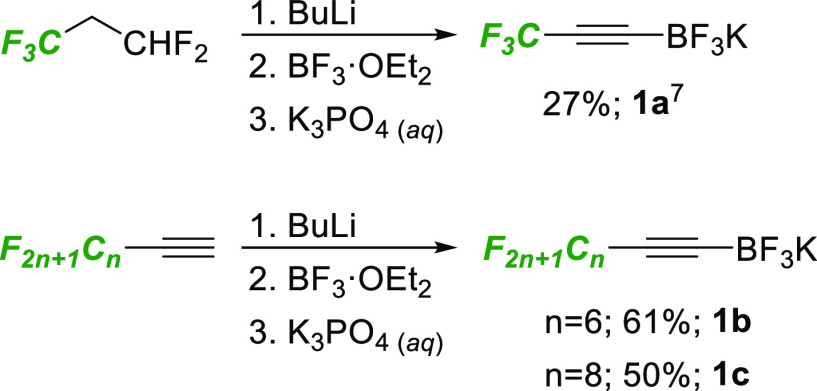
Synthesis of Fluoroalkyl Trifluoroborate Salts

We began our studies by devising an efficient synthesis of the required perfluoroalkyl-substituted alkynyl trifluoroborate salts. We were interested in pursuing a route that avoided the use of glassware-etching substances such as HF or KHF_2_ and were attracted to the work of Ramachandran^7^ that employed the hydrofluorocarbon R-245fa (1,1,1,3,3-pentafluoropropane) as a convenient trifluoromethylacetylide precursor. In addition to reproducing this route, we were able to extend this approach to commercial perfluoroalkyl chain-substituted terminal alkynes to produce a small family of alkyne substrates **1a-c** (Scheme 2).

Turning our attention to the arene forming step, we were disappointed to find that subjecting pyridine-substituted 2-pyrone **2a** to alkyne **1a** in the presence of BF_3_·OEt_2_ in CH_2_Cl_2_ at 40 °C resulted in very low conversion to the corresponding difluoroborane **3a** (Table 1, entry 1). Upon changing the solvent to 1,2-dichoroethane and heating the reaction at 80 °C, 100% conversion was achieved (Table 1, entry 2), providing a mixture of products **3a**, **4a**, and **5** that were characterized by X-ray crystallography. Changing the solvent to toluene provided a marginal improvement in the yield of **3a**, but a significant amount of byproduct **4a** persisted (entry 3). Attempts to converge this mixture to a single product by disproportionation (treatment with BF_3_·OEt_2_ to generate **3a** or with a combination of BF_3_·OEt_2_ and **1a** to generate **5**) failed to bring about a change in composition (see the Supporting Information for more details). We next investigated the use of a stronger Lewis acid in BCl_3_^6d^ (Table 1, entry 4) and were pleased to find that a vigorous reaction took place at room temperature in 30 min to deliver the dichloroborane **3b** which was isolated in 92% yield. BBr_3_ was also successful in promoting the reaction (Table 1, entry 5), affording the dibromoborane **3c** in 60% yield after subsequent purification. We attribute the lower yield in this case to the propensity of this compound to undergo hydrolysis to the corresponding boronic acid.

**Table 1:**
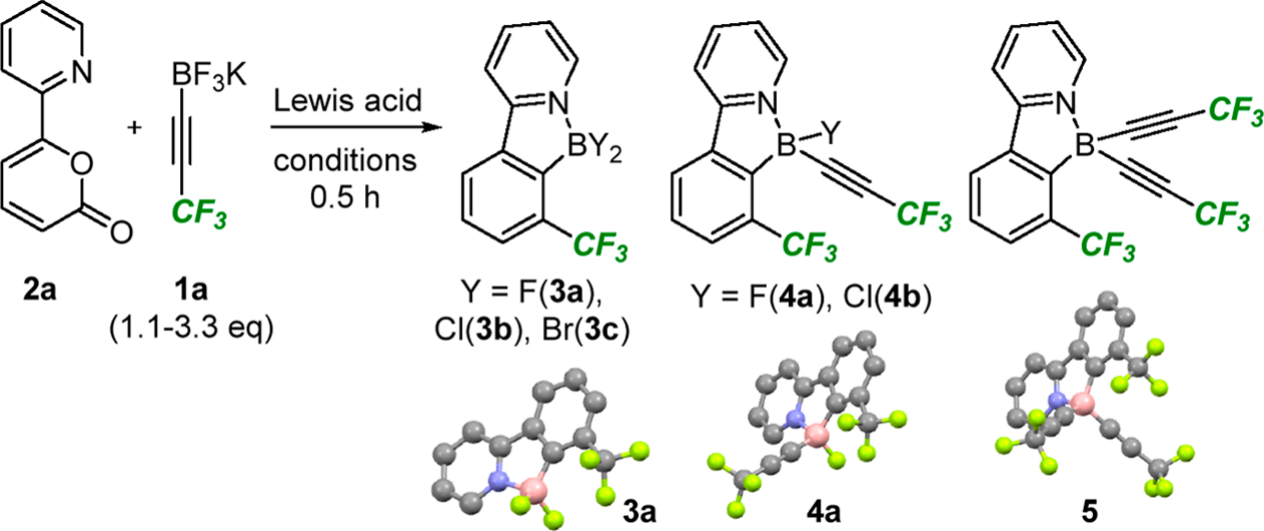
Optimization of the Boron-Directed Cycloaddition

entry	solvent	*T* (^o^C)	Lewis acid	Y	**3a**^a^	**4a**^a^	**5**^a^
1	CH_2_Cl_2_	40	BF_3_·OEt_2_ (3.3 equiv)	F	5%		
2	DCE	80	BF_3_·OEt_2_ (3.3 equiv)	F	64% (34%)^b^	27% (29%)^b^	9% (9%)^b^
3	toluene	80	BF_3_·OEt_2_ (3.3 equiv)	F	63%	29%	6%
4	CH_2_Cl_2_	20	BCl_3_ (1.1 equiv)	Cl	100% (92%)^b^		
5	CH_2_Cl_2_	20	BBr_3_ (1.1 equiv)	Br	82% (60%)^b^		

a Yield estimated by ^1^H NMR spectroscopy.

b Yields in parentheses are of isolated products. DCE: 1,2-Dichloroethane.

**Scheme 3:**
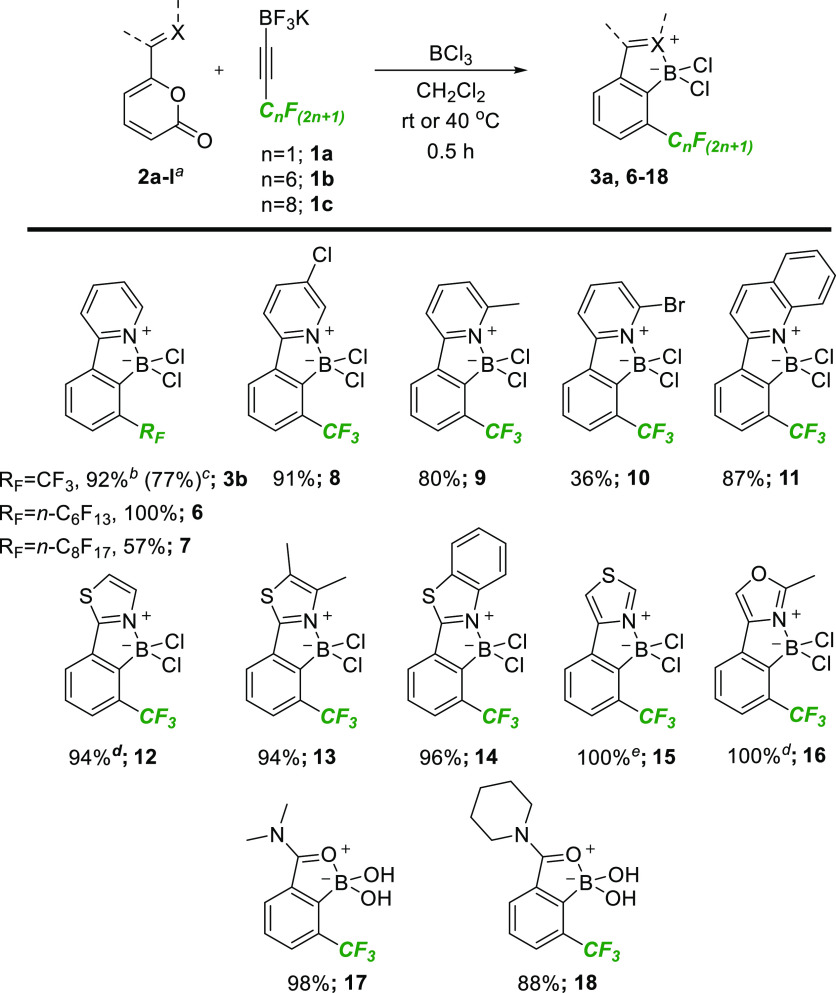
Scope of the Boron-Directed Cycloaddition Reactions carried out on 0.11 mmol of pyrone except where noted. ^b^Reaction carried out on 0.66 mmol of pyrone **2a**. ^c^Reaction carried out on 4.30 mmol of pyrone **2a**. ^d^Reaction stirred at 40 °C for 16 h. ^e^Reaction stirred at 40 °C for 24 h.

With this set of results in hand, we set about exploring the scope of the BCl_3_-promoted process, and our results are summarized in Scheme 3. The synthesis of **3b** could be conducted on gram scale with only a small diminution of yield. Perfluorohexyl-substituted alkynyl trifluoroborate salt **1b** was found to undergo the transformation efficiently, providing the expected product **6** in quantitative yield without the need for a subsequent purification step. The corresponding perfluorooctyl-substituted salt **1c** also underwent the expected reaction but proceeded to only 85% conversion, affording the product **7** in 57% yield after crystallization. In this instance, the low solubility of **7** in CH_2_Cl_2_ resulted in significant precipitation during the reaction, which hampered stirring and probably contributed to the drop in conversion. With respect to the directing group, a selection of substituted pyridines were tolerated in the reaction, providing the products **8–11** in high yield, with the exception of **10** which proceeded in lower conversion, presumably due to the sterically demanding bromide. Thiazole-based analogues **12–15** were also generated in excellent yield, although the reactions to form **12** and **15** were noticeably more sluggish for reasons that are unclear. Likewise, oxazol-4-yl-substituted product **16** was generated in excellent yield after gentle heating. Finally, amides also successfully promoted the arene-forming reaction, although, in this case, products **17** and **18** were not isolated as the expected dichloroboranes, but the corresponding boronic acids.

**Scheme 4:**
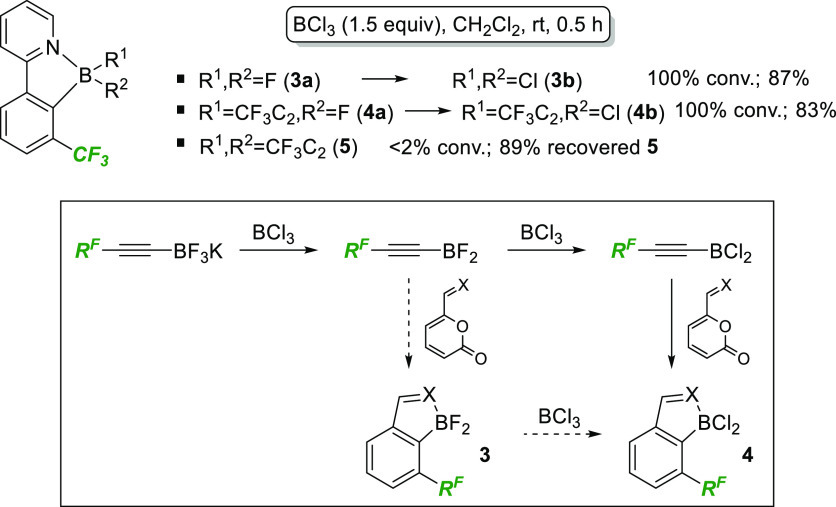
Investigation of Product Disproportionation Using BCl_3_ and the Proposed Mechanism

Given that these reactions had the potential to deliver a large and complex mixture of arylboranes substituted with combinations of alkyne, F, and Cl, it was gratifying that the reaction mixtures were generally extremely clean. As shown in Scheme 4, disproportionation experiments revealed that this was due in part to the efficient exchange of F to Cl in the presence of BCl_3_ (Scheme 4, **3a** to **3b** and **4a** to **4b**), although the alkyne unit resists transfer in this case (Scheme 4, <2% conversion of **5**). Furthermore, these experiments allowed us to put forward a proposed mechanism for the efficient formation of arene dichloroboranes under these conditions. Fluoride abstraction by BCl_3_ generates an alkynyl-BF_2_ intermediate that undergoes halide exchange to the corresponding alkynyl dichloroborane,^8^ which then participates in a rapid cycloaddition to generate the observed product. The cycloaddition reaction must out-compete alkyne disproportionation (to generate dialkynyl- and trialkynylboranes) as the products formed by these intermediates do not converge to the corresponding dichloroboranes and would therefore be observed in crude reaction mixtures. We cannot rule out cycloaddition via the initially formed alkynyl-BF_2_ intermediate (Scheme 4, dashed arrows), but the fact that BCl_3_-promoted reactions proceed faster than BF_3_-mediated cycloadditions suggests that, if this is in operation, it is a minor pathway.

**Scheme 5:**
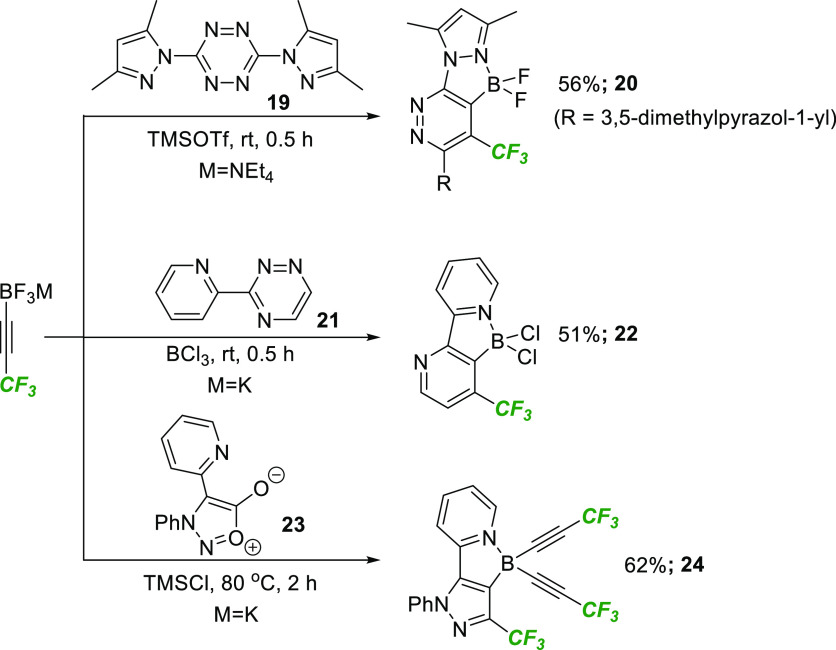
Accessing Fluorinated Heteroarenes

We next explored the suitability of this strategy for the synthesis of heteroaromatic compounds by exploring the boron-directed cycloaddition of alternative substituted heterodienes (Scheme 5). In the event, the Carboni–Lindsey reaction of tetrazine **19** took place at room temperature in the presence of TMSOTf to afford the expected CF_3_-substituted pyridazine **20** in 56% yield. BCl_3_ successfully promoted the cycloaddition of triazine **21** to generate the corresponding pyridine **22**. Finally, pyrazole **24** was generated from the boron-directed cycloaddition of sydnone **23**. In this case, the alkynylborane was formed instead of the corresponding dihaloborane analogue, in line with previous findings.^6e^ Overall, this study confirmed that fluoroalkyl trifluoroborate salts offer a convenient method to generate a range of fluorinated (hetero)arenes under mild conditions and with complete regiocontrol.

**Scheme 6:**
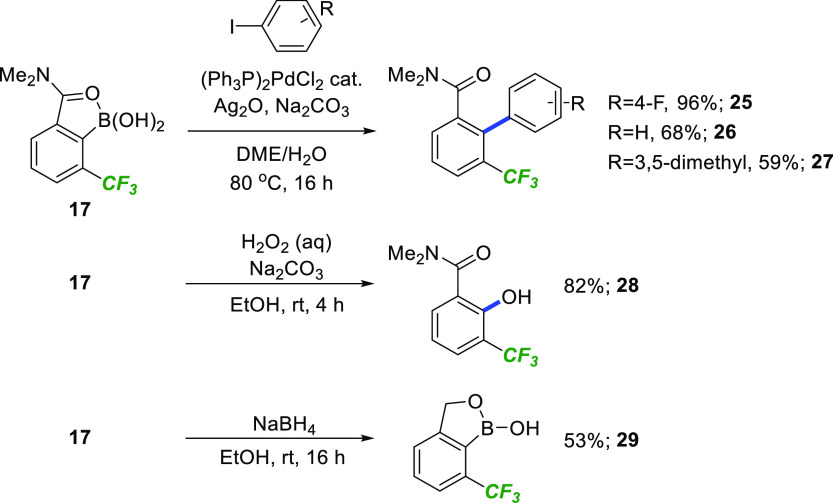
C–B Bond Functionalization

Our final objective was to investigate the reactivity of the boron handle for further elaboration. As shown in Scheme 6, efficient conditions for Suzuki–Miyaura cross coupling were uncovered using aryl iodides, affording the corresponding CF_3_-substituted biaryls **25–27** in good yield. **17** was also converted to the phenol **28** in excellent yield after treatment with H_2_O_2_ under mild, basic conditions. Finally, the CF_3_-substituted benzoxaborole **29** was prepared in useful yield by mild reduction of the amide by NaBH_4_, highlighting the versatility of the intermediate **17** in the synthesis of low-molecular-weight building blocks.

In summary, we present the boron-directed cycloaddition as a novel entry into the important and rapidly developing field of fluoroalkyl-substituted (hetero)aromatic synthesis. A mild, BCl_3_-promoted cycloaddition protocol was discovered, allowing convenient access to a range of fluoroalkyl-substituted benzene derivatives in good to excellent yield. The products obtained were amenable to further manipulation at the B center. Moreover, the directed cycloaddition concept was successfully extended to the synthesis of CF_3_-substituted heteroaromatic compounds.

## Supporting Information Available

Details of experimental procedures and spectroscopic data and NMR spectral data (PDF)
