# Supplementary material for: A Mild and Regioselective Route to Fluoroalkyl Aromatic Compounds via Directed Cycloaddition Reactions
Source: J Org Chem. 2022 Jul 8;87(15):9764–8. doi: 10.1021/acs.joc.2c00800 (PMC9365296; doi:10.1021/acs.joc.2c00800)
Supplement: Supplementary file 1 — jo2c00800_si_001.pdf [file jo2c00800_si_001.pdf]

A Mild and Regioselective Route to Fluoroalkyl Aromatic Compounds via Directed Cycloaddition Reactions

David L. Cousins, Yee Hwee Lim and Joseph P. A. Harrity*

Table of Contents

**General considerations**	**S2**
**Disproportionation studies**	**S3**
**Synthetic procedures and characterisation data**	**S4-S19**
**NMR spectra for new compounds**	**S22-S84**
**References**	**S85**
**X-ray data for 3a**	**S86-S91**
**X-ray data for 4a**	**S92-S96**
**X-ray data for 5**	**S97-S101**
**X-ray data for 9**	**S102-S106**

General considerations

All reactions were conducted in oven-dried glassware under an atmosphere of dry argon unless otherwise stated. All reactions which required heating were carried out using stirrer hot plates fitted with aluminium heating blocks. CH_2Cl_2, Et_2O, THF and toluene were dried before use over an alumina column. All commercially available solvents and reagents were used as supplied or purified using standard laboratory techniques according to methods described by Perrin and Armarego.[1]

Thin layer chromatography was performed on aluminium-backed plates pre-coated with silica, which were developed using standard visualizing agents: ultraviolet light or potassium permanganate. Flash chromatography was performed on silica gel (60 Å, mesh 40-63 µm) or florisil. Melting points were obtained using a either a Stuart or Büchi apparatus and are uncorrected.

^{1}H spectra were recorded at 298 K on a Bruker AVIII HD-400 (400 MHz), Bruker AVI-400 (400 MHz), Bruker AMX-400 (400 MHz) or DPX-400 (400 MHz). Proton magnetic resonance chemical shifts are reported from tetramethylsilane with the residual protic solvent resonance as the internal reference (DMSO-d^6: δ = 2.50 ppm, $CDCl_3$: δ = 7.26 ppm, CD_2Cl_2: δ = 5.32 ppm , acetone-d^6: δ = 2.05 ppm, CD_3CN: δ = 1.94 ppm). Data are reported as follows: chemical shift (ppm), multiplicity (s = singlet, d = doublet, t = triplet, q = quartet, quint = quintet, br = broad, m = multiplet), normalised peak integral (arbitrary units) then coupling constant (Hz). ^{13}C NMR spectra were recorded at 298 K on a Bruker AVIII HD-400 (101 MHz), Bruker AVI-400 (101 MHz), Bruker AMX-400 (101 MHz) or DPX-400 (101 MHz). Carbon magnetic resonance chemical shifts are reported from tetramethylsilane with the solvent as the internal reference (DMSO-d^6: δ = 39.52 ppm, $CDCl_3$: δ = 77.16 ppm, CD_2Cl_2: δ = 53.84 ppm , acetone-d^6: δ = 29.84 ppm, CD_3CN: δ = 1.32 ppm) Data are reported as follows: chemical shift (ppm), multiplicity (if appropriate) (s = singlet, d = doublet, t = triplet, q = quartet, quint = quintet, br = broad, m = multiplet). ^{19}F (376 MHz) and ^{11}B (128 MHz) NMR spectra were recorded at 298 K on a Bruker AMX-400, Bruker AVIII HD-400 or a Bruker AV Neo and the chemical shifts are uncorrected. Data are reported as follows: chemical shift (ppm), multiplicity (if appropriate) (s = singlet, d = doublet, t = triplet, q = quartet, quint = quintet, br = broad, m = multiplet).

Infrared spectra were recorded on a Perkin-Elmer Paragon 100 FTIR spectrometer. Spectra were obtained from neat compounds through the use of a standard ATR attachment, and the most structurally relevant bands are quoted in cm^{-1}. Bands are characterized as broad (br), strong (s), medium (m) or weak (w). High-resolution mass spectra (HRMS) were recorded on either a MicroMass LCT or an Agilent LC-QTOF 6545B operating in Electrospray mode (TOF ES).

Disproportionation Studies

Subjection of either pure **5** or a 1:1:1 mixture of **3a:4:5** to BF_3.OEt_2 under the reaction conditions (Figure S1) resulted in no change to the composition in either case. Similarly subjection of **3a** to the reaction conditions resulted in clean recovery of the starting compound:

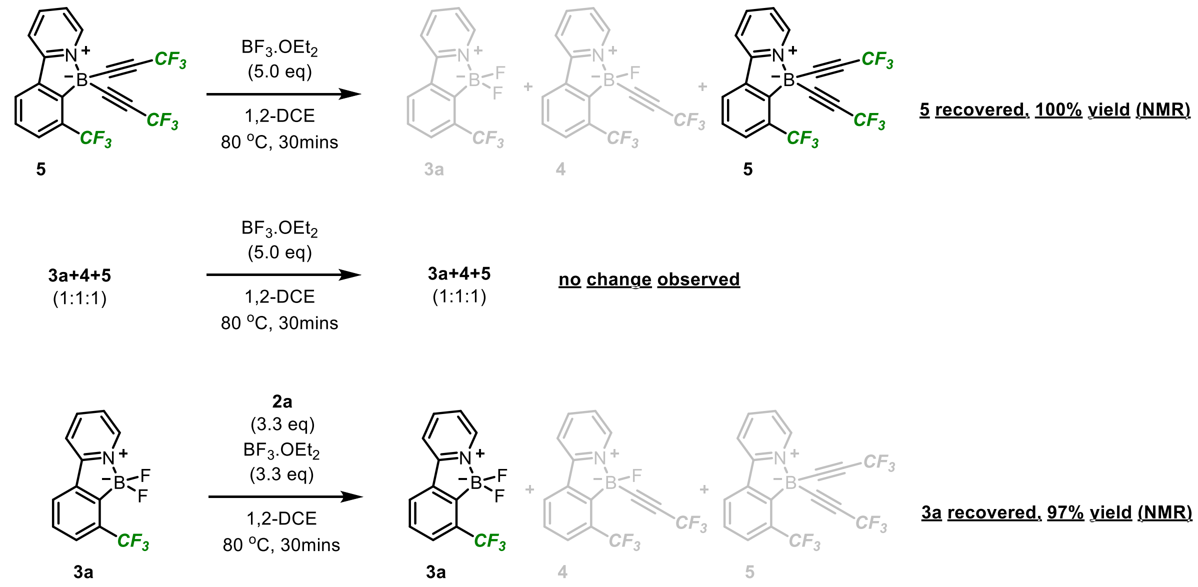

Figure S1 Disproportionation studies.

Synthetic procedures and compound characterisation

(1) Preparation of starting materials

Synthesis of potassium (1,1,1-trifluoroprop-2-yn-3-yl)trifluoroborate; 1a

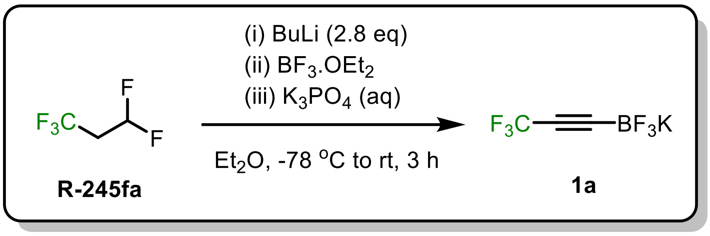

Scheme S1 Synthesis of alkyne trifluoroborate salts.

This method in Scheme S1 was adapted from that published by Ramachandran *et al*.[2]

A 3-neck round-bottomed flask under argon was charged with a solution of R-245fa in anhydrous Et_2O (90.0 mL, 45 mmol) and cooled to -35 °C. ^{n}BuLi in cyclohexane (66.3 mL, 128 mmol) was added slowly to the flask and the mixture was stirred at -35 °C for 1 hour. The mixture was then cooled to -78 °C by exchanging the cooling bath and allowing 15 minutes stirring to acclimatise. $BF_3.OEt_2$ (5.6 mL, 45 mmol) was added, dropwise, to the mixture which was then stirred for 15 minutes at -78 °C. The mixture was warmed to -20 °C by exchanging cooling baths and allowing 15 minutes of stirring to acclimatise, then a solution of K_3PO_4 (12.5 g, 59 mmol) in H_2O (14 mL) was added slowly *via* syringe. The mixture was allowed to warm to room temperature then concentrated *in vacuo* to dryness. Acetone (500 mL) was added to the flask and the product was extracted by vigorous stirring and agitation over a period of 20 minutes, followed by filtration and concentration of the filtrate *in vacuo*. The crude product was purified by dissolving the residue in acetone (10 mL) and adding dropwise CH_2Cl_2 (250 mL) without stirring. The resultant precipitate was collected and dried *in vacuo*, providing the crude **1a** as a colourless solid (3.8 g, 45%). Prior to recrystallisation, the precipitate was dissolved in acetone and the solution was dried over anhydrous $MgSO_4$ then concentrated *in vacuo*. The product was dispersed in toluene (200 mL) inside a flask fitted with a reflux condenser and heated at 80 °C, whereupon MeCN (50 mL) was added slowly. After cooling the mixture to ambient temperature, the mixture was further cooled to -20 C over a period of 1 hour. The cold mixture was filtered and the collected crystals were dried inside an evacuated desiccator over anhydrous P_2O_5 for 60 hours, affording **1a** as a colourless solid (2.3 g, 27%). **M.p.** 182 – 183 °C; **^{13}C{^{1}H} NMR (101 MHz, CD_3CN)** δ_C ppm 77.7 – 74.5 (m), 115.2 (q, J = 255.5 Hz); **^{19}F NMR (376 MHz, CD_3CN)** δ_F ppm -49.8, -137.5 (q, J = 32.0 Hz); **^{11}B NMR (128 MHz, CD_3CN)** δ_B ppm -2.4 (q, J = 32.0 Hz). (The data were in agreement with those published).[3]

General procedure A: Synthesis of perfluoroalkyl-substituted alkynyl trifluoroborate salts

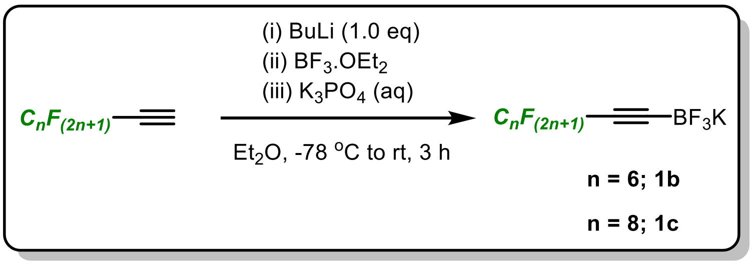

Scheme S2 Synthesis of perfluoroalkyl-substituted alkyne trifluoroborate salts

This method in Scheme S2 was adapted from that published by Bardin *et al.*,[3] deploying aspects of Ramachandran's[2] HF-free approach.

To a solution of the terminal alkyne (1.2 eq) in anhydrous Et_2O (0.3 M) at -60 °C was added dropwise ^nBuLi in cyclohexane (1.0 eq). The mixture was stirred, keeping the temperature between -55 and -60 °C for 1 hour then cooled to -78 °C. The resultant cold acetylide solution was transferred *via* cannula into a flask containing BF_3.OEt$_2$ (1.4 eq) in Et_2O (0.3 M) at -78 °C and stirred at this temperature for 1 hour. The mixture was warmed to -20 °C by exchanging cooling baths, allowing 15 minutes of stirring to acclimatise, then a solution of K_3PO_4 (1.3 eq) in H_2O (4.0 M) was added slowly *via* syringe. The mixture was allowed to warm to room temperature then concentrated *in vacuo* to dryness, the desired product was then extracted by vigorous stirring and agitation in acetone for 15 minutes followed by filtration and concentration *in vacuo*. The crude product was dissolved in acetone and CH_2Cl_2 was added, leading to precipitation. The precipitate was collected and dried inside an evacuated desiccator over anhydrous P_2O_5 for 24 hours, affording the desired product as a colourless solid.

Potassium (perfluorohexylethynyl)trifluoroborate **1b**

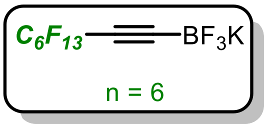

Following general procedure A, using (perfluorohexyl)acetylene (1.0 g, 2.9 mmol), ^nBuLi in cyclohexane (1.5 mL, 2.5 mmol), BF_3.OEt$_2$ (0.4 mL, 3.4 mmol) and K_3PO_4 (0.7 g, 3.2 mmol). The title compound was obtained as a colourless solid (676 mg, 61%). **M.p.** 214 – 215 °C; **^{13}C{^{1}H} NMR (101 MHz, acetone-d^6)** δ_C ppm 118.1 (qt, J = 33.0, 288.0 Hz), 104.5 – 114.2 (m, multiple signals), 74.2; **^{19}F NMR (376 MHz, acetone-d^6)** δ_F ppm -81.7 – -81.8 (m), -94.0 – -94.1 (m), -121.5 – -121.9 (m), -122.8 – -123.1 (m), -123.3 – -123.6 (m), -126.7 – -126.9 (m), -137.4 (q, J = 30.5 Hz); **^{11}B NMR (128 MHz, acetone-d^6)** δ_B ppm -2.3 (q, J = 30.5 Hz); **FTIR (neat)** ν_{max} / cm^{-1} 2227 (w), 1366 (w), 1227 (s), 1194 (s), 1144 (s), 706 (s); **HRMS (ESI-TOF)** *m/z* [M-K]$^-$ calculated for $[C_8{}^{11}B^{19}F_{16}]^-$ 410.9844, found 410.9839. The data were in agreement with those published.[3]

Potassium (perfluorooctylethynyl)trifluoroborate **1c**

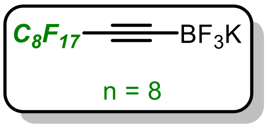

Following general procedure A, using (perfluorooctyl)acetylene (1.0 g, 2.3 mmol), ^nBuLi in cyclohexane (1.1 mL, 1.9 mmol), BF_3.OEt$_2$ (0.3 mL, 2.6 mmol) and K_3PO_4 (0.5 g, 2.4 mmol). The title compound was obtained as a colourless solid (513 mg, 50%). **M.p.** 241 – 244 °C; ^{13}**C{^{1}H} NMR (101 MHz, acetone-d^6)** δ_C ppm 118.0 (qt, J = 33.0, 288.0), 105.3 – 104.6 (m, multiple signals), 74.1; **^{19}F NMR (376 MHz, acetone-d^6)** δ_F ppm -81.7 (t, J = 9.5 Hz), -94.1 – -93.9 (m), -121.6 – -121.2 (m), -122.7 - -122.3 (m), -123.1 – -122.8 (m), -123.5 – -123.1 (m), -126.8 – -126.6 (m), -137.4 (q, J = 30.5 Hz); **^{11}B NMR (128 MHz,**

acetone-d^6) δ_B ppm -2.3 (q, J = 30.5 Hz); **FTIR (neat)** ν_{max} / cm^{-1} 2227 (w), 1371 (w), 1201 (s), 1146 (s), 1015 (m), 710 (m); **HRMS (ESI-TOF)** *m/z* [M-K]$^-$ calculated for $[C_{10}{}^{11}BF_{20}]^-$ 510.9780, found 510.9779.

Synthesis of tetra-*N*-ethylammonium (1,1,1-trifluoroprop-2-yn-3-yl)trifluoroborate; 1d

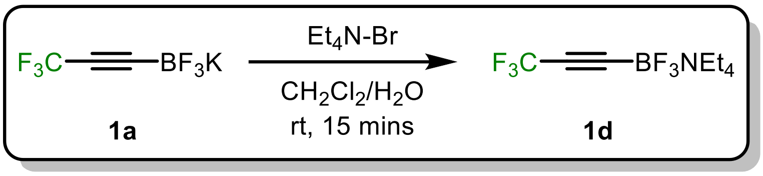

Scheme S3 Counterion exchange.

To a rapidly stirring surry of **1a** (1.0 g, 5.0 mmol) and tetra-*n*-ethylammonium bromide (1.0 g, 5.3 mmol) in CH_2Cl_2 (13.6 mL) was added dropwise H_2O (6.6 mL). The mixture was stirred rapidly for a further 15 minutes, then allowed to settle and the layers were separated. The aqueous layer was extracted with CH_2Cl_2 (3 x 25 mL) and the combined organic layers were dried over anhydrous $MgSO_4$ and concentrated *in vacuo*. The product obtained was dried dried inside an evacuated desiccator over anhydrous P_2O_5 for 24 hours, affording **1d** as a colourless solid (1.4 g, 95%). **M.p.** 48 – 49 °C; **^{1}H NMR (400 MHz, CD_3CN)** δ_H ppm 3.17 (q, 8H, J = 7.5 Hz), 1.11 – 1.30 (m, 12H); **^{13}C{^{1}H} NMR (101 MHz, CD_3CN)** δ_C ppm 115.2 (q, J = 252.0 Hz), 77.3 – 75.3 (m), 53.3 – 52.9 (m), 7.7 (q, J = 11.0 Hz); **^{19}F NMR (376 MHz, $CDCl_3$)** δ_F ppm -48.8, -136.6 (q, J = 33.5 Hz); **^{11}B NMR (128 MHz, CD_3CN)** δ_B ppm -2.2 (q, J = 33.5 Hz); **FTIR (neat)** ν_{max} / cm^{-1} 3000 (w), 2213 (w), 1491 (w), 1397 (w), 1255 (m), 1123 (s), 1031 (s), 787 (w); **HRMS (ESI-TOF)** *m/z* [M-NEt$_4$]$^-$ calculated for $[C_3{}^{11}BF_6]^-$ 160.9997, found 161.0008.

General procedure B: Synthesis of *N*-heterocycle-substituted 2-pyrones[4]

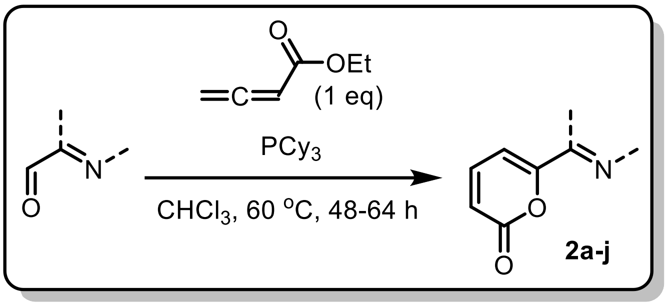

Scheme S4 Pyrone synthesis.

To a stirring solution of aldehyde (3.0 – 3.6 eq) and PCy_3 (1.0 – 1.1 eq) in anhydrous $CHCl_3$ under argon was added dropwise ethyl 2,3-butadienoate (1.0 eq). The mixture was sealed inside the reaction tube and heated at 60 °C with stirring for 48-64 hours, then cooled to room temperature and concentrated *in vacuo*. The crude residue was purified by flash column chromatography on silica gel (eluting with EtOAc in hexanes) then recrystallised from CH_2Cl_2/hexanes. The purified product was then dried over P_2O_5 inside an evacuated desiccator for a period of 24 hours.

2-Pyrones not mentioned here were prepared according to the general procedure and their data were in agreement with those published.[4]

Preparation of 6-(5-chloro-2-pyridyl)-2-pyrone, 2b

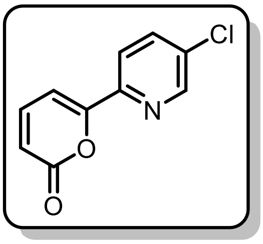

Following general procedure B, using 5-chloro-2-pyridinecarboxaldehyde (600 mg, 4.2 mmol), PCy_3 (390 mg, 1.4 mmol), ethyl 2,3-butadienoate (160 µL, 1.4 mmol) and $CHCl_3$ (10 mL). The crude product was purified by flash column chromatography on silica gel (gradient elution, 0-30% EtOAc in hexanes) followed by recrystallisation and drying, to afford **2b** as colourless crystals (216 mg, 74%). **M.p.** = 180 – 181 °C; **^{1}H NMR (400 MHz, CDCl$_3$)** δ_H ppm 8.58 (dd, 1H, J = 0.5, 2.5 Hz), 7.95 (dd, 1H, J = 0.5, 8.5 Hz), 7.80 (dd, 1H, J = 2.5, 8.5 Hz), 7.49 (dd, 1H, J = 7.0, 9.5 Hz), 7.32 (dd, 1H, J = 1.0, 7.0 Hz), 6.39 (dd, 1H, J = 1.0, 9.5 Hz); **^{13}C{^{1}H} NMR (101 MHz, CDCl$_3$)** δ_C ppm 161.3, 158.4, 148.9, 147.0, 143.8, 137.1, 133.6, 121.2, 116.5, 103.4; **FTIR (neat)** ν_{max} / cm^{-1} 3081 (w), 2832 (w), 1735 (s), 1630 (w), 1546 (m), 1103 (m), 805 (s); **HRMS (ESI-TOF)** *m/z* [M+H]$^+$ calculated for $[C_{10}H_7{}^{35}ClNO_2]^+$ 208.0160, found 208.0157.

Preparation of 6-(6-bromo-2-pyridyl)-2-pyrone, 2d

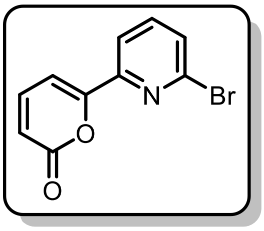

Following general procedure B, using 6-Bromo-2-pyridinecarboxaldehyde (930 mg, 5.0 mmol), PCy_3 (430 mg, 1.5 mmol), ethyl 2,3-butadienoate (160 µL, 1.4 mmol) and $CHCl_3$ (10 mL). The crude product was purified by flash column chromatography on silica gel (gradient elution, 0-30% EtOAc in hexanes) followed by recrystallisation and drying, to afford **2d** as yellow crystals (189 mg, 54%). **M.p.** = 104 – 105 °C; **^{1}H NMR (400 MHz, CDCl$_3$)** δ_H ppm 7.93 (dd, 1H, J = 1.0, 8.0 Hz), 7.66 (t, 1H, J = 8.0 Hz), 7.54 – 7.44 (m, 2H), 7.33 (dd, 1H, J = 1.0, 7.0 Hz), 6.39 (dd, 1H, J = 1.0, 9.5 Hz); **^{13}C{^{1}H} NMR (101 MHz, CDCl$_3$)** δ_C ppm 161.1, 157.7, 149.9, 143.7, 142.2, 139.5, 129.5, 119.2, 116.9, 103.9; **FTIR (neat)** ν_{max} / cm^{-1} 3101 (w), 2808 (w), 2568 (w), 2047 (w), 1743 (s), 1561 (m), 1108 (m), 789 (s); **HRMS (ESI-TOF)** *m/z* [M+H]$^+$ calculated for $[C_{10}H_7{}^{79}BrNO_2]^+$ 251.9655, found 251.9651.

Preparation of 6-(2-quinolinyl)-2-pyrone, 2e

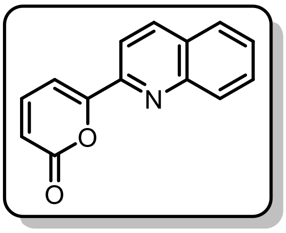

Following general procedure B, using 2-quinolinecarboxaldehyde (790 mg, 5.0 mmol), PCy_3 (420 mg, 1.5 mmol), ethyl 2,3-butadienoate (160 µL, 1.4 mmol) and $CHCl_3$ (10 mL). The crude product was purified by flash column chromatography on silica gel (gradient elution, 0-30% EtOAc in hexanes) followed by recrystallisation and drying, to afford **2e** as brown crystals (293 mg, 94%). **M.p.** = 160 – 161 °C; **^{1}H NMR (400 MHz, CDCl$_3$)** δ_H ppm 8.30 (d, 1H, J = 8.5 Hz), 8.14 (d, 1H, J = 8.5 Hz), 8.10 (ddd, 1H, J = 1.0, 1.5, 8.5 Hz), 7.86 (dd, 1H, J = 1.0, 8.0 Hz), 7.76 (ddd, 1H, J = 1.5, 7.0, 8.5 Hz), 7.64 – 7.53 (m, 3H), 6.47 – 6.40 (m, 1H); **^{13}C{^{1}H} NMR (101 MHz, CDCl$_3$)** δ_C ppm 161.8, 159.7, 149.0, 148.0, 144.0, 137.5, 130.4, 129.9, 128.6, 127.9, 117.7, 116.4, 103.7; **FTIR (neat)** ν_{max} / cm^{-1} 3063 (w), 2855 (w), 2758 (w), 1723 (s), 1500 (m), 1072 (m), ; **HRMS (ESI-TOF)** *m/z* [M+H]$^+$ calculated for $[C_{14}H_{10}NO_2]^+$ 224.0712, found 224.0702.

Preparation of 6-(4,5-dimethyl-1,3-thiazol-2-yl)-2-pyrone, 2g

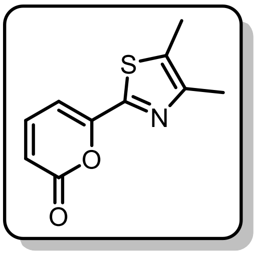

Following general procedure B, using 4,5-Dimethyl-1,3-thiazole-2-carbaldehyde (590 mg, 4.2 mmol), PCy_3 (390 mg, 1.4 mmol), ethyl 2,3-butadienoate (160 µL, 1.4 mmol) and $CHCl_3$ (10 mL). The crude product was purified by flash column chromatography on silica gel (gradient elution, 0-30% EtOAc in hexanes) followed by recrystallisation and drying, to afford **2g** as yellow crystals (188 mg, 65%). **M.p.** = 184 – 185 °C; **^{1}H NMR (400 MHz, $CDCl_3$)** δ_H ppm 7.41 (dd, 1H, J = 7.0, 9.5 Hz), 7.01 (dd, 1H, J = 0.5, 7.0 Hz), 6.30 (dd, 1H, J = 0.5, 9.5 Hz), 2.43 (s, 3H), 2.37 (s, 3H); **^{13}C{^{1}H} NMR (101 MHz, $CDCl_3$)** δ_C ppm 160.8, 155.4, 154.2, 151.4, 143.7, 131.6, 115.6, 101.0, 14.9, 11.8; **FTIR (neat)** ν_{max} / cm^{-1} 3307 (w), 3078 (w), 2928 (w), 1713 (s), 1619 (m), 1524 (s), 1269 (m); **HRMS (ESI-TOF)** *m/z* [M+H]$^+$ calculated for $[C_{10}H_{10}NO_2S]^+$ 208.0427, found 208.0423.

Preparation of 6-(1,3-benzothiazol-2-yl)-2-pyrone, 2h

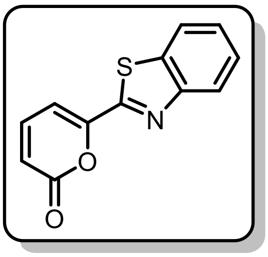

Following general procedure B, using 1,3-Benzothiazole-2-carbaldehyde (690 mg, 4.2 mmol), PCy_3 (390 mg, 1.4 mmol), ethyl 2,3-butadienoate (160 µL, 1.4 mmol) and $CHCl_3$ (10 mL). The crude product was purified by flash column chromatography on silica gel (gradient elution, 0-30% EtOAc in hexanes) followed by recrystallisation and drying, to afford **2h** as orange crystals (133 mg, 41%). **M.p.** = 205 – 206 °C; **^{1}H NMR (400 MHz, CD_2Cl_2)** δ_H ppm 8.09 (ddd, 1H, J = 0.5, 1.0, 8.5 Hz), 8.01 (ddd, 1H, J = 0.5, 1.0, 8.0 Hz), 7.57 (ddd, 1H, J = 1.0, 7.0, 8.5 Hz), 7.54 – 7.46 (m, 2H), 7.30 (dd, 1H, J = 1.0, 6.5 Hz), 6.42 (dd, 1H, J = 1.0, 9.5 Hz); **^{13}C{^{1}H} NMR (101 MHz, CD_2Cl_2)** δ_C ppm 159.9, 159.4, 154.8, 153.8, 143.2, 135.7, 127.1, 126.5, 124.0, 122.2, 117.5, 103.9; **FTIR (neat)** ν_{max} / cm^{-1} 3078 (w), 2828 (w), 2354 (w), 1728 (s), 1630 (w), 1546 (m), 1315 (m), 1258 (m), 1106 (m); **HRMS (ESI-TOF)** *m/z* [M+H]$^+$ calculated for $[C_{12}H_8NO_2S]^+$ 230.0270, found 230.0266.

General procedure C: Synthesis of amide-substituted 2-pyrones

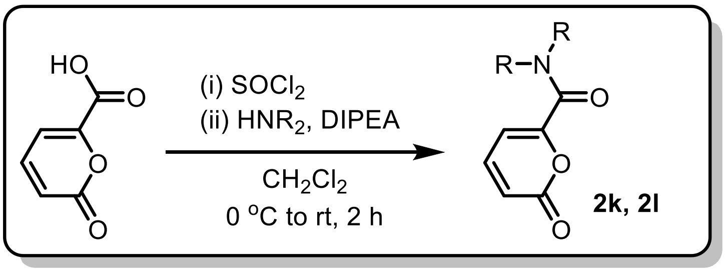

Scheme S5 Pyrone amide synthesis

A solution of 6-(hydroxycarbonyl)-2-pyrone[5] (1.0 eq) in $SOCl_2$ was heated at reflux for 16 hours, then concentrated *in vacuo*. The resultant residue was dissolved in CH_2Cl_2 (10 mL) and cooled to 0 °C, whereupon the secondary amine (1.0 eq) then DIPEA 1.0 eq) were added slowly. The mixture was allowed to warm to room temperature over 2 hours, then washed with saturated aqueous $NaHCO_3$ (10 mL), followed by H_2O (10 mL), followed by brine (10 mL). The layers were separated and the organic layer was dried over anhydrous $MgSO_4$ then concentrated *in vacuo*. The crude product was purified by flash column chromatography on silica gel, then dried over P_2O_5 inside an evacuated desiccator for a period of 24 hours.

6-(*N*,*N*-dimethylformamido)-2-pyrone **2k**

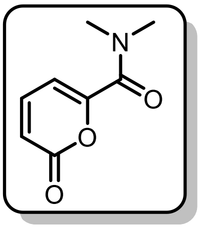

Following general procedure C, using 6-(hydroxycarbonyl)-2-pyrone (300 mg, 2.1 mmol), $SOCl_2$ (2.8 mL), dimethylamine (2.0 M in THF, 1.1 mL, 2.1 mmol) and DIPEA (370 µL, 2.1 mmol). The crude product was purified by flash column chromatography on silica gel (gradient elution, 50 – 100% EtOAc in hexanes), followed by drying, to afford the title compound **2k** as a colourless solid (157 mg, 44%). **M.p.** = 62 – 63 °C; **^{1}H NMR (400 MHz, $CDCl_3$)** δ_H ppm 7.39 (dd, 1H, J = 6.5, 9.5 Hz), 6.66 (dd, 1H, J = 1.0, 6.5 Hz), 6.36 (dd, 1H, J = 1.0, 9.5 Hz), 3.12 (s, 3H), 3.03 (s, 3H); **^{13}C{^{1}H} NMR (101 MHz, $CDCl_3$)** δ_C ppm 161.5, 159.9, 155.8, 143.0, 117.7, 107.2, 38.5, 36.3. The data were in agreement with those published.[6]

6-(piperidinocarbonyl)-2-pyrone **2l**

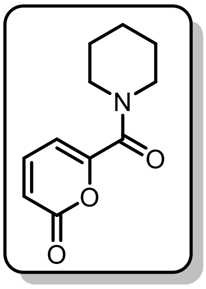

Following general procedure C, using 6-(hydroxycarbonyl)-2-pyrone (250 mg, 1.78 mmol), $SOCl_2$ (10 mL), piperidine (180 µL, 1.78 mmol) and DIPEA (310 µL, 1.78 mmol). The crude product was purified by flash column chromatography on silica gel (eluting with EtOAc), followed by recrystallisation from CH_2Cl_2/hexanes then drying, to afford the title compound **2l** as colourless crystals (104 mg, 28 %). **M.p.** = 79 – 80 °C; **^{1}H NMR (400 MHz, acetone-d^6)** δ_H ppm 7.58 (dd, 1H, J = 6.5, 9.5 Hz), 6.56 (dd, 1H, J = 1.0, 6.5 Hz), 6.34 (dd, 1H, J = 1.0, 9.5 Hz), 3.61 – 3.46 (m, 4H), 1.75 – 1.65 (m, 2H), 1.65 – 1.56 (m, 4H); **^{13}C{^{1}H} NMR (101 MHz, acetone-d^6)** δ_C ppm 161.0, 160.4, 157.0, 144.3, 117.4, 106.3, 48.5, 43.8, 27.2, 26.2, 25.1; **FTIR (neat)** ν_{max} / cm^{-1} 3086 (w), 2942 (w), 2866 (w), 1721 (s), 1638 (s), 1618 (m), 1448 (m), 1274 (m), 1089 (m); **HRMS (ESI-TOF)** *m/z* $[M+H]^+$ calculated for $[C_{11}H_{14}NO_3]^+$ 208.0968, found 208.0965.

(2) Directed cycloaddition studies

$BF_3.OEt_2$-promoted cycloaddition between 2a and 1a; Synthesis of adducts 3a, 4a and 5

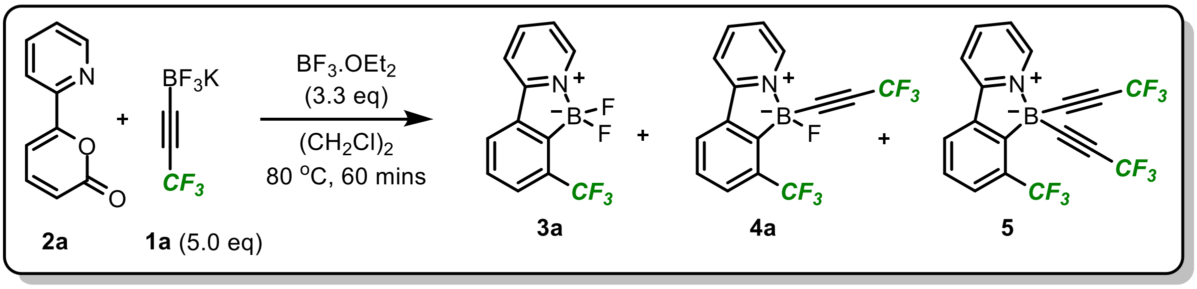

Scheme S6 $BF_3.OEt_2$ promoted cycloaddition.

To a suspension of **2a** (19 mg, 0.11 mmol) and **1a** (110 mg, 0.55 mmol) in $(CH_2Cl)_2$ (1 mL) at 80 °C was added dropwise $BF_3.OEt_2$ (44 µL, 0.36 mmol). The mixture was allowed to stir at 80 °C for 1 hour, then cooled to room temperature and diluted with CH_2Cl_2 (10 mL). The mixture was then washed with $NaHCO_3$ (sat. aq., 4 mL) and the layers were separated. The aqueous layer was extracted with CH_2Cl_2 (3 x 5 mL) and the combined organic layers were dried over anhydrous $MgSO_4$ and concentrated *in vacuo*. The compounds **3a**, **4a** and **5** were purified by flash column chromatography on silica gel (gradient elution, 0-50% ethyl acetate in petroleum ether (60-80)), providing; **3a** as a colourless solid (10 mg, 34%), **m.p.** = 217-218 °C (dec), **4a** as a colourless solid (11 mg, 29%), **m.p.** = 153-154 °C and **5** as a colourless solid (4 mg, 9%), **m.p.** = 210 °C.

[2-(2-Pyridyl)-6-(trifluoromethyl)phenyl]difluoroborane **3a**

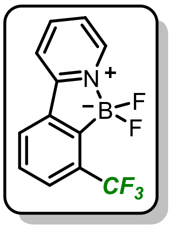

^{1}H NMR (400 MHz, acetone-d^{6}) δ_H ppm 8.71 (d, 1H, J = 5.5 Hz), 8.49 (dt, 1H, J = 1.5, 7.5 Hz), 8.40 (d, 1H, J = 8.0 Hz), 8.26 (d, 1H, J = 7.5 Hz), 7.87 (ddd, 1H, J = 1.5, 5.5, 7.5 Hz), 7.78 (d, 1H, J = 8.0 Hz), 7.66 (t, 1H, J = 7.5 Hz); **^{13}C{^{1}H} NMR (101 MHz, acetone-d^{6})** δ_C ppm 154.5, 145.9, 142.9, 139.3 (t, J = 4.0 Hz), 133.4 (q, J = 33.0 Hz), 130.5, 129.0 (q, J = 4.5 Hz), 126.5, 126.2, 125.6 (q, J = 273.0 Hz), 120.0; **^{19}F NMR (376 MHz, acetone-d^{6})** δ_F ppm -62.0 (t, J = 6.0 Hz), -158.6 – -159.1 (m); **^{11}B NMR (128 MHz, acetone-d^{6})** δ_B ppm 7.67 (t, J = 49.5 Hz); **FTIR (neat)** ν_{max} / cm^{-1} 2925 (w), 2851 (w), 1626 (m), 1496 (m), 1117 (s), 775 (s); **HRMS (ESI-TOF)** *m/z* [M+Na]$^+$ calculated for $[C_{12}H_7{}^{11}BF_5NNa]^+$ 294.0484, found 294.0484; **Single crystal X-ray analysis** see page S86 of this document.

[2-(2-Pyridyl)-6-(trifluoromethyl)phenyl](3,3,3-trifluoro-1-propynyl)fluoroborane **4a**

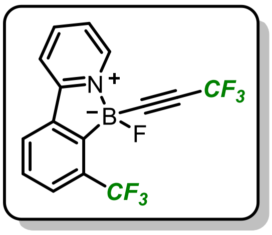

^{1}H NMR (400 MHz, acetone-d^{6}) δ_H ppm 8.92 (d, 1H, J = 5.5 Hz), 8.57 (dt, 1H, J = 1.5, 8.0 Hz), 8.49 (dd, 1H, J = 1.5, 8.0 Hz), 8.34 (d, 1H, J = 7.5 Hz), 7.93 – 7.99 (m, 1H), 7.84 (d, 1H, J = 8.0 Hz), 7.73 (t, 1H, J = 7.5 Hz); **^{13}C{^{1}H} NMR (101 MHz, acetone-d^{6})** δ_C ppm 155.8, 146.2, 144.3, 139.5 (d, J = 3.0 Hz), 133.6 (q, J = 33.0 Hz), 129.5 (q, J = 4.5 Hz), 130.8, 127.0, 126.5, 125.7 (q, J = 273.5 Hz), 120.6, 114.8 (q, J = 255.0 Hz), 80.9 (q, J = 49.5 Hz); **^{19}F NMR (376 MHz, acetone-d^{6})** δ_F ppm -50.3 (d, J = 2.5 Hz), -61.1 (d, J = 6.5 Hz), -186.4 – -187.0 (m); **^{11}B NMR (128 MHz, acetone-d^{6})** δ_B ppm 2.8 (d, J = 56.5 Hz); **FTIR (neat)** ν_{max} / cm^{-1} 2919 (w), 2850 (w), 2238 (w), 2213 (w), 1626 (m), 1496 (m), 1257 (s), 1136 (s), 1115 (s), 769 (s); **HRMS (ESI-TOF)** *m/z* [M+Na]$^+$ calculated for $[C_{15}H_7{}^{11}BF_7NNa]^+$ 368.0453, found 368.0453. **Single crystal X-ray analysis** see page S92 of this document.

[2-(2-Pyridyl)-6-(trifluoromethyl)phenyl]bis(3,3,3-trifluoro-1-propynyl)borane **5**

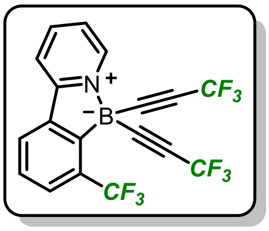

^{1}H NMR (400 MHz, acetone-d^{6}) δ_H ppm 9.09 (d, 1H, J = 5.5 Hz), 8.65 – 8.55 (m, 2H), 8.44 (d, 1H, J = 8.0 Hz), 8.02 (ddd, 1H, J = 6.5, 6.0, 2.0 Hz), 7.92 (d, 1H, J = 8.0 Hz), 7.78 (dt, 1H, J = 8.0, 1.0 Hz); **^{13}C{^{1}H} NMR (101 MHz, acetone-d^{6})** δ_C ppm 157.0, 145.8, 145.0, 139.6, 133.1 (q, J = 33.0 Hz), 130.5, 130.0 (q, J = 4.5 Hz), 127.5, 126.7, 125.6 (q, J = 274.0 Hz), 121.1, 114.8 (q, J = 255.0 Hz), 82.4 (q, J = 57.5 Hz); **^{19}F NMR (376 MHz, acetone-d^{6})** δ_F ppm -50.4 (s), -60.5 (s); **^{11}B NMR (128 MHz, acetone-d^{6})** δ_B ppm -11.1; **FTIR (neat)** ν_{max} / cm^{-1} 2927 (w), 2227 (m) 1627 (m), 1497 (m), 1254 (s), 1124 (s), 772 (s); **HRMS (ESI-TOF)** *m/z* [M+Na]$^+$ calculated for $[C_{18}H_7{}^{11}BF_9NNa]^+$ 442.0421, found 442.0426. **Single crystal X-ray analysis** see page S97 of this document.

TMS-Cl promoted cycloaddition between 2a and 1a: Synthesis of [2-(2-Pyridyl)-6-(trifluoromethyl)phenyl](3,3,3-trifluoro-1-propynyl)chloroborane 4b

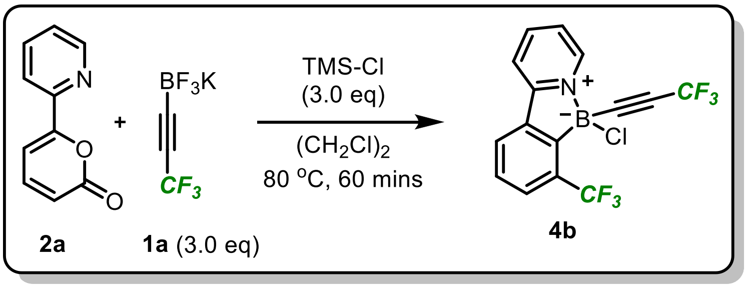

Scheme S7 TMSCl promoted cycloaddition.

To a suspension of **2a** (19 mg, 0.11 mmol) and **1a** (68 mg, 0.34 mmol) in $(CH_2Cl)_2$ (1.0 mL) at 50 °C was added dropwise TMS-Cl (43 µL, 0.34 mmol). The mixture was heated at 80 °C for 1 hour, then cooled to room temperature and diluted with CH_2Cl_2 (10 mL). The mixture was then washed with $NaHCO_3$ (sat. aq., 4 mL) and the layers were separated. The aqueous layer was extracted with CH_2Cl_2 (3 x 5 mL) and the combined organic layers were dried over anhydrous $MgSO_4$ and concentrated *in vacuo*. The crude product was purified by flash column chromatography on florisil (gradient elution, 0 – 50% EtOAc in hexanes), affording **4b** as a colourless solid (31 mg, 78%). **M.p.** = 205 – 206 °C (dec); **^{1}H NMR (400 MHz, acetone-d$_6$)** δ_H ppm 9.03 (d, 1H, J = 6.0 Hz), 8.61 (dt, 1H, J = 1.5, 8.0 Hz), 8.55 (d, 1H, J = 8.0 Hz), 8.41 (d, 1H, J = 7.5 Hz), 8.01 (ddd, 1H, J = 1.5, 6.0, 7.5 Hz), 7.89 (d, 1H, J = 8.0 Hz), 7.77 (ddt, 1H, J = 1.0, 1.5, 8.0 Hz); **^{13}C{^{1}H} NMR (101 MHz, acetone-d^6)** δ_C ppm 155.8, 146.3, 144.8, 138.8, 133.0 (q, J = 33.0 Hz), 130.8, 130.1 (q, J = 4.5 Hz), 127.3, 126.7, 125.5 (q, J = 273.5 Hz), 120.8, 114.9 (q, J = 255.0 Hz), 82.6 (q, J = 49.0 Hz); **^{19}F NMR (376 MHz, acetone-d^6)** δ_F ppm -50.5, -60.0; **^{11}B NMR (128 MHz, acetone-d^6)** δ_B ppm -2.4; **FTIR (neat)** ν_{max} / cm^{-1} 2917 (w), 2849 (w), 2219 (w), 1628 (w), 1497 (w), 1255 (m), 1136 (s); **HRMS (ESI-TOF)** *m/z* [M+Na]$^+$ calculated for $[C_{15}H_7{}^{11}BClF_6NNa]^+$ 384.0157, found 384.0161.

General procedure D: Synthesis of fluoroalkyl-substituted, stabilised boranes

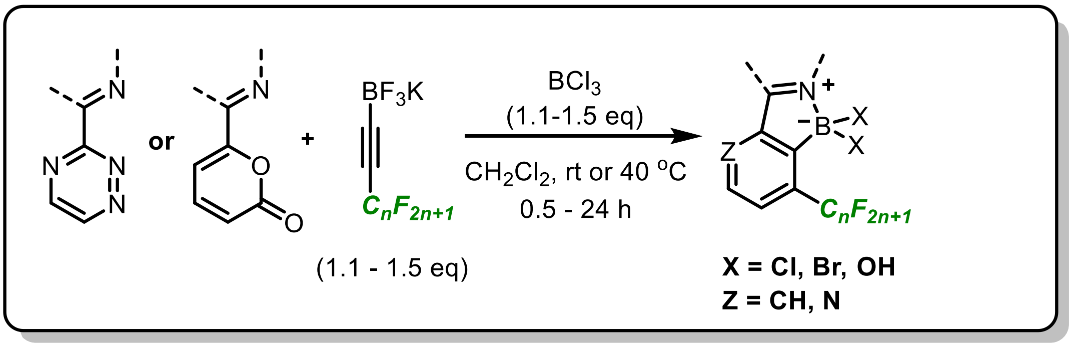

Scheme S8 General method for BCl_3 promoted cycloaddition.

To a stirring suspension of the diene (1.0 eq) and the fluoroalkyl-substituted potassium alkynyl trifluoroborate salt (1.1 – 1.5 eq) in CH_2Cl_2 (0.11 M) at room temperature (19 °C) or 40 °C was added dropwise BX_3 (1.0 M in CH_2Cl_2, 1.1 – 1.5 eq). The reaction mixture was stirred at the starting temperature for the stated amount of time, then cooled (if necessary) and diluted tenfold with CH_2Cl_2. The crude mixture was washed with $NaHCO_3$ (sat. aq., 4 mL per 10 mL diluting CH_2Cl_2) and the layers were separated. The aqueous layer was extracted with CH_2Cl_2 (3 x 5 mL per 10 mL diluting CH_2Cl_2, unless otherwise stated) and the combined organic layers were dried over anhydrous $MgSO_4$ then concentrated *in vacuo*. If necessary, the compound was purified by flash column chromatography on silica gel (gradient elution, 0-80% EtOAc in petroleum ether), precipitation from acetone *via* addition of petroleum ether or recrystallised from hot acetone.

2-(2-(Dichloroboryl)-3-(trifluoromethyl)phenyl)pyridine **3b**

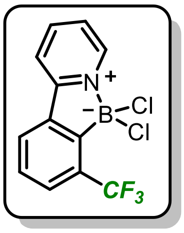

Following general procedure D, using pyrone **2a** (114 mg, 0.66 mmol), **1a** (144 mg, 0.72 mmol) and BCl_3 solution (0.72 mL, 0.72 mmol) stirring at room temperature (19 °C) for 30 minutes, the product was purified by flash column chromatography on silica gel (gradient elution, 0-80% EtOAc in petroleum ether), providing **3b** as a colourless solid (185 mg, 92%). **M.p.** = 279-280 °C; **^{1}H NMR (400 MHz, acetone-d^{6})** δ_H ppm 8.99 (d, 1H, J = 6.0 Hz), 8.61 – 8.54 (m, 1H), 8.53 – 8.46 (m, 1H), 8.37 (d, 1H, J = 7.5 Hz), 7.99 (ddd, 1H, J = 7.5, 6.0, 1.0 Hz), 7.74 (td, 1H, J = 8.0, 1.0 Hz), 7.87 (d, 1H, J = 8.0 Hz); **^{13}C{^{1}H} NMR (101 MHz, acetone-d^{6})** δ_C ppm 154.2, 146.3, 144.2, 137.4, 132.8 (q, J = 33.5), 130.7, 130.4 (q, J = 4.5 Hz), 127.1, 126.6, 125.53 (q, J = 274.0 Hz), 120.3; **^{19}F NMR (376 MHz, acetone-d^{6})** δ_F ppm -59.5; **^{11}B NMR (128 MHz, acetone-d^{6})** δ_B ppm 6.6; **FTIR (neat)** ν_{max} / cm^{-1} 3095 (w), 1626 (m), 1307 (s), 1165 (s), 1145 (s), 763 (s), 701(s); **HRMS (ESI-TOF)** *m/z* [M+Na]$^{+}$ calculated for $[C_{12}H_7{}^{11}B^{35}Cl_2F_3NNa]^+$ 325.9893, found 325.9893.

Gram scale synthesis: Following general procedure D with minor modifications to the work up, using pyrone **2a** (745 mg, 4.30 mmol), **1a** (946 mg, 4.73 mmol) and BCl_3 solution (4.73 mL, 473 mmol) stirring at room temperature (19 °C) for 30 minutes. The reaction mixture was diluted with DCM (200 mL) and quenched with $NaHCO_3$ (50 mL). The aqueous layer was extracted with DCM (3 x 50 mL) and the crude product was purified by flash column chromatography on silica gel (gradient elution, 0-80% EtOAc in petroleum ether), providing **3b** as a colourless solid (1.01 g, 77%). Analytical data consistent with those above.

2-(2-(Dichloroboryl)-3-(perfluorohexyl)phenyl)pyridine **6**

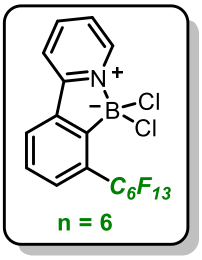

Following general procedure D, using pyrone **2a** (19 mg, 0.11 mmol), **1b** (76 mg, 0.17 mmol) and BCl_3 solution (0.17 mL, 0.17 mmol) stirring at 40 °C for 30 minutes, further purification was not deemed necessary, providing **6** as a colourless solid (61 mg, 100%). **M.p.** = 228 – 229 °C; **^{1}H NMR (400 MHz, acetone-d^{6})** δ_H ppm 9.04 – 8.96 (m, 1H), 8.58 (ddd, 1H, J = 1.5, 7.5, 8.0 Hz), 8.54 – 8.49 (m, 1H), 8.43 (dd, 1H, J = 1.0, 7.5 Hz), 7.99 (ddd, 1H, J = 1.0, 6.0, 7.5 Hz), 7.85 – 7.76 (m, 2H); **^{13}C{^{1}H} NMR (101 MHz, acetone-d^{6})** δ_C ppm 154.1, 146.3, 144.1, 137.7, 132.4 – 132.1 (m), 131.4 (t, J = 25.5 Hz), 130.6, 127.4, 126.7, 120.3, 123.0 – 105.4 (m, multiple peaks); **^{19}F NMR (376 MHz, acetone-d^{6})** δ_F ppm -81.6 – -81.7 (m), -105.4 – -105.6 (m), -119.3 – -119.7 (m), -121.7 – -122.2 (m), -123.2 – -123.4 (m), -126.6 – -126.9 (m); **^{11}B NMR (128 MHz, acetone-d^{6})** δ_B ppm 6.8; **FTIR (neat)** ν_{max} / cm^{-1} 3086 (w), 2981 (w), 1626 (w), 1497 (w), 1215 (s), 1143 (s), 714 (s); **HRMS (ESI-TOF)** *m/z* [M-Cl]$^{+}$ calculated for $[C_{17}H_7{}^{11}B^{35}ClF_{13}N]^+$ 518.0148, found 518.0144.

2-(2-(Dichloroboryl)-3-(perfluorooctyl)phenyl)pyridine **7**

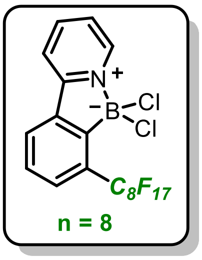

Following general procedure D, using pyrone **2a** (19 mg, 0.11 mmol), **1c** (91 mg, 0.17 mmol) and BCl_3 solution (0.17 mL, 0.17 mmol) stirring at room temperature (19 °C) for 30 minutes, the product was recrystallised from acetone, providing **7** as a colourless solid (41 mg, 57%). **M.p.** = 242 – 243 °C; **^{1}H NMR (400 MHz, acetone-d^{6})** δ_H ppm 9.00 (d, 1H, J = 6.0 Hz), 8.59 (ddd, 1H, J = 1.5, 7.5, 8.0 Hz), 8.52 (dt, 1H, J = 1.0, 8.0 Hz), 8.46 – 8.40 (m, 1H), 7.99 (ddd, 1H, J = 1.0, 6.0, 7.5 Hz), 7.86 – 7.75 (m, 2H); **^{13}C{^{1}H} NMR (101 MHz, acetone-d^{6})** δ_C ppm 154.1, 146.3, 144.1, 137.7, 132.1 – 132.5 (m), 131.4 (t, J = 25.5 Hz), 130.6, 127.4, 126.7, 120.3, 121.0 – 107.0 (m, multiple signals); **^{19}F NMR (376 MHz, acetone-d^{6})** δ_F ppm -81.6 – -81.7 (m), -105.4 – -105.6 (m), -119.2 – -119.6 (m), -121.5 – -121.7 (m), -122.1 – -122.6 (m), -123.0 – -123.4 (m), -126.5 – -126.9 (m); **^{11}B NMR (128 MHz,

acetone-d^6) δ_B ppm 6.8; **FTIR (neat)** ν_{max} / cm^{-1} 3091 (w), 2924 (w), 2853 (w), 1626 (w), 1497 (w), 1220 (s), 1207 (s), 1146 (s), 761 (s); **HRMS (ESI-TOF)** *m/z* [M+H]$^+$ calculated for $[C_{19}H_7{}^{11}B^{35}ClF_{17}N]^+$ 618.0085, found 618.0073.

2-(2-(Dibromoboryl)-3-(trifluoromethyl)phenyl)pyridine **3c**

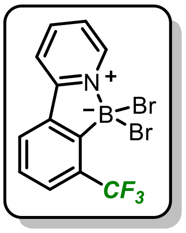

Following general procedure D, using pyrone **2a** (19 mg, 0.11 mmol), **1a** (34 mg, 0.17 mmol) and BBr_3 solution (0.17 mL, 0.17 mmol) stirring at 40 °C for 30 minutes, the product was purified by flash column chromatography on silica gel (gradient elution, 0-80% EtOAc in petroleum ether), providing **3c** as a colourless solid (26 mg, 60%). **M.p.** = 218 – 219 °C (dec); **^{1}H NMR (400 MHz, acetone-d^6)** δ_H ppm 9.15 (d, 1H, J = 6.0 Hz), 8.58 (ddd, 1H, J = 1.5, 7.5, 8.0 Hz), 8.53 – 8.48 (m, 1H), 8.41 (d, 1H, J = 7.5 Hz), 8.00 (ddd, 1H, J = 1.0, 6.0, 7.5 Hz), 7.90 (d, 1H, J = 8.0 Hz), 7.76 (ddd, 1H, J = 1.0, 1.5, 8.5 Hz); **^{13}C{^{1}H} NMR (101 MHz, acetone-d^6)** δ_C ppm 153.7, 146.4, 145.3, 136.7, 132.8 (q, J = 33.0 Hz), 130.8 (q, J = 4.5 Hz), 130.7, 127.3, 126.7, 125.6 (q, J = 274.5 Hz), 121.5; **^{19}F NMR (376 MHz, acetone-d^6)** δ_F ppm -58.5; **^{11}B NMR (128 MHz, acetone-d^6)** δ_B ppm -1.9; **FTIR (neat)** ν_{max} / cm^{-1} 3088 (w), 2927 (w), 2855 (w), 1628 (m), 1495 (m), 1306 (m), 1132 (m), 780 (m); **HRMS (ESI-TOF)** *m/z* [M-Br]$^+$ calculated for $[C_{12}H_7{}^{11}B^{79}BrF_3N]^+$ 311.9802, found 311.9802.

5-Chloro-2-(2-(dichloroboryl)-3-(trifluoromethyl)phenyl)pyridine **8**

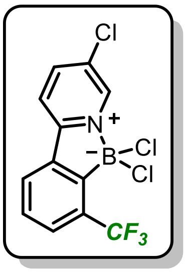

Following general procedure D, using pyrone **2b** (23 mg, 0.11 mmol), **1a** (34 mg, 0.17 mmol) and BCl_3 (0.17 mL, 0.17 mmol) stirring at room temperature (19 °C) for 30 minutes, further purification was not deemed necessary, providing **8** as a brown solid (34 mg, 91%). **M.p.** = 297-298 °C; **^{1}H NMR (400 MHz, acetone-d^6)** δ_H ppm 9.02 (dd, 1H, J = 2.0, 0.5 Hz), 8.63 (dd, 1H, J = 8.5, 2.0 Hz), 8.54 (dd, 1H, J = 8.5, 0.5 Hz), 8.38 (d, 1H, J = 8.0 Hz), 7.89 (d, 1H, J = 8.0 Hz), 7.78 – 7.72 (m, 1H); **^{13}C{^{1}H} NMR (101 MHz, acetone-d^6)** δ_C ppm 153.2, 146.2, 142.8, 136.7, 133.7, 132.8 (q, J = 33.5 Hz), 130.9, 130.6 (q, J = 4.5 Hz), 127.3, 125.4 (q, J = 274.0 Hz), 121.6; **^{19}F NMR (376 MHz, acetone-d^6)** δ_F ppm -59.6; **^{11}B NMR (128 MHz, acetone-d^6)** δ_B ppm 6.8; **FTIR (neat)** ν_{max} / cm^{-1} 3128 (w), 3070 (w), 1494 (m), 1312 (s), 1131 (s), 815 (s), 778 (s), 751 (s), 707 (s); **HRMS (ESI-TOF)** *m/z* [M+Na]$^+$ calculated for $[C_{12}H_6{}^{11}B^{35}Cl_3F_3NNa]^+$ 359.9503, found 359.9505.

2-(2-(Dichloroboryl)-3-(trifluoromethyl)phenyl)-6-methylpyridine **9**

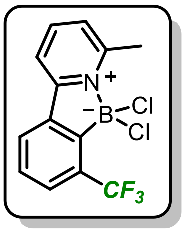

Following general procedure D, using pyrone **2c** (21 mg, 0.11 mmol), **1a** (34 mg, 0.17 mmol) and BCl_3 (0.17 mL, 0.17 mmol) stirring at 40 °C for 30 minutes, the product was purified by flash column chromatography on silica gel (gradient elution, 0-80% EtOAc in petroleum ether), providing **9** as a colourless solid (28 mg, 80%). **M.p.** = 288-289 °C; **^{1}H NMR (400 MHz, acetone-d^6)** δ_H ppm 8.38 (t, 1H, J = 8.0 Hz), 8.34 – 8.27 (m, 2H), 7.85 (d, 1H, J = 8.0 Hz), 7.77 – 7.66 (m, 2H), 3.21 (s, 3H); **^{13}C{^{1}H} NMR (101 MHz, acetone-d^6)** δ_C ppm 158.6, 155.2, 145.5, 137.1, 132.3 (q, J = 33.0 Hz), 130.51 (q, J = 4.5 Hz), 130.50, 128.6, 126.5, 125.6 (q, J = 274.0 Hz), 117.6, 21.0; **^{19}F NMR (376 MHz, acetone-d^6)** δ_F ppm -58.8; **^{11}B NMR (128 MHz, acetone-d^6)** δ_B ppm 7.4; **FTIR (neat)** ν_{max} / cm^{-1} 3072 (w), 2926 (w), 1492 (m), 1312 (s), 1167 (s), 1109 (s), 707 (s), 698 (s); **HRMS (ESI-TOF)** *m/z* [M+Na]$^+$ calculated for $[C_{13}H_9{}^{11}B^{35}Cl_2F_3NNa]^+$ 340.0049, found 340.0053; **Single crystal X-ray analysis** see page S102 of this document.

2-Bromo-6-(2-(dichloroboryl)-3-(trifluoromethyl)phenyl)pyridine **10**

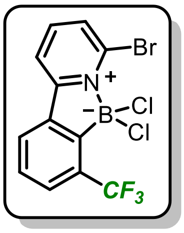

Following general procedure D, using pyrone **2d** (28 mg, 0.11 mmol), **1a** (34 mg, 0.17 mmol) and BCl_3 (0.17 mL, 0.17 mmol) stirring at room temperature (19 °C) for 30 minutes, the product was purified by flash column chromatography on silica gel (gradient elution, 0-80% EtOAc in petroleum ether), providing **10** as a colourless solid (15 mg, 36%). **M.p.** = 253 °C; **^{1}H NMR (400 MHz, DMSO-d^6)** δ_H ppm 8.59 (dd, 1H, J = 8.0, 1.0 Hz), 8.44 (d, 1H, J = 8.0 Hz), 8.36 (t, 1H, J = 8.0 Hz), 8.17 (dd, 1H, J = 8.0, 1.0 Hz), 7.89 (d, 1H, J = 8.0 Hz), 7.78 – 7.71 (m, 1H); **^{13}C{^{1}H} NMR (101 MHz, DMSO-d^6)** δ_C ppm 156.6, 146.3, 137.9, 135.1, 132.3, 130.3 (q, J = 32.5 Hz), 130.2 (q, J = 5.0 Hz), 130.1, 126.8, 124.3 (q, J = 275.0 Hz), 118.9; **^{19}F NMR (376 MHz, acetone-d^6)** δ_F ppm -58.7; **^{11}B NMR (128 MHz, acetone-d^6)** δ_B ppm 8.8; **FTIR (neat)** ν_{max} / cm^{-1} 3128 (w), 3071 (w), 1494 (m), 1312 (s), 1131 (s), 815 (s), 708 (s); **HRMS (ESI-TOF)** *m/z* [M+Na]$^+$ calculated for $[C_{12}H_6{}^{11}B^{79}Br^{35}Cl_2F_3NNa]^+$ 403.8998, found 403.9001.

2-(2-(Dichloroboryl)-3-(trifluoromethyl)phenyl)quinoline **11**

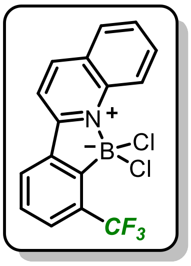

Following general procedure D, using pyrone **2e** (25 mg, 0.11 mmol), **1a** (34 mg, 0.17 mmol) and BCl_3 (0.17 mL, 0.17 mmol) stirring at 40 °C for 30 minutes. The aqueous layer was extracted first with CH_2Cl_2 (7 x 5 mL), then with EtOAc (3 x 5 mL). Further purification was not deemed necessary, providing **11** as a brown solid (34 mg, 87%). **M.p.** = >300 °C; **^{1}H NMR (400 MHz, DMSO-d^6)** δ_H ppm 9.22 (d, 1H, J = 8.5 Hz), 8.95 (d, 1H, 9.0 Hz), 8.74 (d, 1H, J = 8.5 Hz), 8.66 (d, 1H, J = 8.0 Hz), 8.38 (dd, 1H, J = 8.0, 1.5 Hz), 8.20 (ddd, 1H, J = 8.5, 7.0, 1.5 Hz), 7.98 (d, 1H, J = 8.0 Hz), 7.92 (ddd, 1H, J = 8.0, 7.0, 1.0 Hz), 7.86 – 7.79 (m, 1H); **^{13}C{^{1}H} NMR (101 MHz, DMSO-d^6)** δ_C ppm 155.4, 147.4, 138.4, 136.3, 133.8, 130.4 (q, J = 33.0 Hz), 130.4 (q, J = 4.5 Hz), 130.2 (2 x C), 129.0, 128.4, 128.0, 124.4 (q, J = 274.5 Hz), 122.5, 116.3,; **^{19}F NMR (376 MHz, DMSO-d^6)** δ_F ppm -57.3; **^{11}B NMR (128 MHz, DMSO-d^6)** δ_B ppm 6.9; **FTIR (neat)** ν_{max} / cm^{-1} 3125 (w), 3070 (w), 3046 (w), 1494 (m), 1312 (s), 1130 (s), 815 (s), 707 (s); **HRMS (ESI-TOF)** *m/z* [M+Na]$^+$ calculated for $[C_{16}H_9{}^{11}B^{35}Cl_2F_3NNa]^+$ 376.0049, found 376.0055.

2-(2-(Dichloroboryl)-3-(trifluoromethyl)phenyl)thiazole **12**

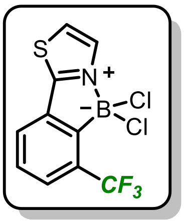

Following general procedure D, using pyrone **2f** (20 mg, 0.11 mmol), **1a** (34 mg, 0.17 mmol) and BCl_3 (0.17 mL, 0.17 mmol) stirring at 40 °C for 16 hours inside a sealed tube, further purification was not deemed necessary, providing **12** as a brown solid (32 mg, 94%). **M.p.** = 250-251 °C (dec); **^{1}H NMR (400 MHz, acetone-d^6)** δ_H ppm 8.30 (d, 1H, J = 3.5 Hz), 8.24 (d, 1H, J = 7.5 Hz), 8.17 (d, 1H, J = 3.5 Hz), 7.88 (d, 1H, J = 8.0 Hz), 7.75 – 7.70 (m, 1H); **^{13}C{^{1}H} NMR (101 MHz, acetone-d^6)** δ_C ppm 171.7, 134.3, 134.0, 132.7 (q, J = 33.5 Hz), 130.7, 130.5 (q, J = 4.5 Hz), 127.6, 126.3, 125.3 (q, J = 274.0 Hz); **^{19}F NMR (376 MHz, acetone-d^6)** δ_F ppm -59.6; **^{11}B NMR (128 MHz, acetone-d^6)** δ_B ppm 4.5; **FTIR (neat)** ν_{max} / cm^{-1} 3123 (w), 1312 (s), 1166 (s), 1130 (s), 816 (s), 709 (s), 701 (s); **HRMS (ESI-TOF)** *m/z* [M+Na]$^+$ calculated for $[C_{10}H_5{}^{11}B^{35}Cl_2F_3NNaS]^+$ 331.9457, found 331.9460.

2-(2-(Dichloroboryl)-3-(trifluoromethyl)phenyl)-4,5-dimethylthiazole **13**

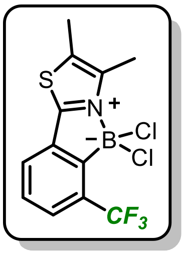

Following general procedure D, using pyrone **2g** (23 mg, 0.11 mmol), **1a** (34 mg, 0.17 mmol) and BCl_3 (0.17 mL, 0.17 mmol) stirring at 40 °C for 30 minutes, further purification was not deemed necessary, providing **13** as a brown solid (35 mg, 94%). **M.p.** = >300 °C; **^{1}H NMR (400 MHz, acetone-d^{6})** δ_H ppm 8.07 (d, 1H, J = 7.5 Hz), 7.83 (d, 1H, J = 8.0 Hz), 7.70 – 7.65 (m, 1H), 2.70 (q, 3H, J = 1.0 Hz), 2.59 (q, 3H, J = 1.0 Hz); **^{13}C{^{1}H} NMR (101 MHz, acetone-d^{6})** δ_C ppm 168.2, 142.8, 134.0, 133.1, 132.5 (q, J = 33.5 Hz), 130.6, 130.2 (q, J = 4.5 Hz), 126.7, 125.3 (q, J = 274.5 Hz), 12.1 (q, J = 4.0 Hz), 11.9 (q, J = 4.0 Hz); **^{19}F NMR (376 MHz, acetone-d^{6})** δ_F ppm -59.3; **^{11}B NMR (128 MHz, acetone-d^{6})** δ_B ppm 5.0; **FTIR (neat)** ν_{max} / cm^{-1} 3092 (w), 3029 (w), 1570 (w), 1305 (s), 1169 (s), 1112 (s), 815 (s), 717 (s); **HRMS (ESI-TOF)** *m/z* [M+Na]$^+$ calculated for $[C_{12}H_9{}^{11}B^{35}Cl_2F_3NNaS]^+$ 359.9770, found 359.9773.

2-(2-(Dichloroboryl)-3-(trifluoromethyl)phenyl)benzo[d]thiazole **14**

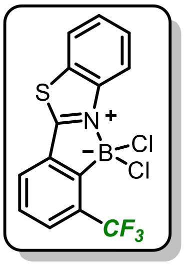

Following general procedure D, using pyrone **2h** (25 mg, 0.11 mmol), **1a** (34 mg, 0.17 mmol) and BCl_3 (0.17 mL, 0.17 mmol) stirring at 40 °C for 30 minutes, further purification was not deemed necessary, providing **14** as a brown solid (38 mg, 96%). **M.p.** = >300 °C; **^{1}H NMR (400 MHz, DMSO-d^{6})** δ_H ppm 8.52 (d, 1H, J = 3.0 Hz), 8.50 (d, 1H, J = 3.0 Hz), 8.29 (d, 1H, J = 8.0 Hz), 8.01 (d, 1H, J = 8.0 Hz), 7.89 (ddd, 1H, J = 8.5, 7.5, 1.0 Hz), 7.81 (td, 1H, J = 8.0, 1.0 Hz), 7.76 (ddd, 1H, J = 8.5, 7.5, 1.0 Hz); **^{13}C{^{1}H} NMR (101 MHz, DMSO-d^{6})** δ_C ppm 173.5, 139.2, 134.2, 133.0, 130.8 (q, J = 5.0 Hz), 130.7 (q, J = 33.0 Hz), 130.5, 129.3, 128.9, 127.6, 125.6, 124.1 (q, J = 275.0 Hz), 118.5; **^{19}F NMR (376 MHz, DMSO-d^{6})** δ_F ppm -57.9; **^{11}B NMR (128 MHz, DMSO-d^{6})** δ_B ppm 4.8; **FTIR (neat)** ν_{max} / cm^{-1} 3074 (w), 2926 (w), 1425 (m), 1302 (s), 1124 (s), 755 (s), 710 (s), 714 (s); **HRMS (ESI-TOF)** *m/z* [M+Na]$^+$ calculated for $[C_{14}H_7{}^{11}B^{35}Cl_2F_3NNaS]^+$ 381.9614, found 381.9623.

4-(2-(Dichloroboryl)-3-(trifluoromethyl)phenyl)thiazole **15**

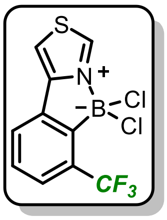

Following general procedure D, using pyrone **2i** (20 mg, 0.11 mmol), **1a** (34 mg, 0.17 mmol) and BCl_3 (0.17 mL, 0.17 mmol) stirring at 40 °C for 24 hours inside a sealed tube, further purification was not deemed necessary, providing **15** as a brown solid (34 mg, 100%). **M.p.** = 209-210 °C (dec); **^{1}H NMR (400 MHz, acetone-d^{6})** δ_H ppm 10.02 (d, 1H, J = 2.0 Hz), 8.36 (d, 1H, J = 2.0 Hz), 8.14 (d, 1H, J = 7.5 Hz), 7.75 (d, 1H, J = 8.0 Hz), 7.68 – 7.62 (m, 1H); **^{13}C{^{1}H} NMR (101 MHz, acetone-d^{6})** δ_C ppm 157.2, 153.4, 134.3, 133.1 (q, J = 33.0 Hz), 130.5, 128.6 (q, J = 4.5 Hz), 126.3, 125.5 (q, J = 274.0 Hz), 113.9; **^{19}F NMR (376 MHz, acetone-d^{6})** δ_F ppm -59.2; **^{11}B NMR (128 MHz, acetone-d^{6})** δ_B ppm 4.4; **FTIR (neat)** ν_{max} / cm^{-1} 3122 (w), 1319 (s), 1165 (s), 1165 (s), 1115 (s), 1077 (s), 815 (s), 756 (s); **HRMS (ESI-TOF)** *m/z* [M+Na]$^+$ calculated for $[C_{10}H_5{}^{11}B^{35}Cl_2F_3NNaS]^+$ 331.9457, found 331.9460.

4-(2-(Dichloroboryl)-3-(trifluoromethyl)phenyl)-2-methyloxazole **16**

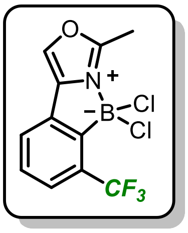

Following general procedure D, using pyrone **2j** (19 mg, 0.11 mmol), **1a** (34 mg, 0.17 mmol) and BCl_3 (0.17 mL, 0.17 mmol) stirring at 40 °C for 16 hours inside a sealed tube, further purification was not deemed necessary, providing **16** as a brown solid (34 mg, 100%). **M.p.** = 252-253 °C (dec); **^{1}H NMR (400 MHz, acetone-d^{6})** δ_H ppm 8.55 (s, 1H), 7.97 (d, 1H, J = 7.5 Hz), 7.74 (d, 1H, J = 8.0 Hz), 7.68 – 7.59 (m, 1H), 2.98 (s, 3H); **^{13}C{^{1}H} NMR (101 MHz, acetone-d^{6})** δ_C ppm 164.6, 139.6, 133.4, 133.1 (q, J

= 33.0 Hz), 130.9, 130.5, 128.6 (q, J = 5.0 Hz), 126.7, 125.5 (q, J = 274.0 Hz), 12.8; **^{19}F NMR (376 MHz, acetone-d^6)** δ_F ppm -59.0; **^{11}B NMR (128 MHz, acetone-d^6)** δ_B ppm 4.0; **FTIR (neat)** ν_{max} / cm^{-1} 3174 (w), 1302 (s), 1174 (s), 1123 (s), 823 (s), 762 (s); **HRMS (ESI-TOF)** *m/z* [M+Na]$^+$ calculated for $[C_{11}H_7{}^{11}B^{35}Cl_2F_3NNaO]^+$ 329.9842, found 329.9845.

(2-(Dimethylcarbamoyl)-6-(trifluoromethyl)phenyl)boronic acid **17**

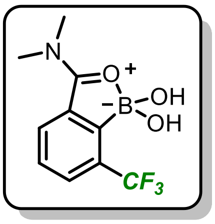

Following general procedure D, using pyrone **2k** (18 mg, 0.11 mmol), **1a** (34 mg, 0.17 mmol) and BCl_3 (0.17 mL, 0.17 mmol) stirring at 40 °C for 30 minutes, the product was purified by precipitation from a saturated acetone solution *via* the addition of petroleum ether (5 x $V_{acetone}$) followed by agitation, then chilling to -20 °C over 30 minutes. The precipitation was performed two times providing **17** as a colourless solid (28 mg, 98%). **M.p.** = 208-209 °C (dec); **^{1}H NMR (400 MHz, acetone-d^6)** δ_H ppm 8.47 (d, 1H, J = 8.0 Hz), 8.01 (d, 1H, J = 8.0 Hz), 7.80 – 7.73 (m, 1H), 3.98 (s, 3H), 3.62 (s, 3H); **^{13}C{^{1}H} NMR (101 MHz, acetone-d^6)** δ_C ppm 171.8, 132.5 (q, J = 4.5 Hz), 132.2, 132.1 (q, J = 34.0), 132.0, 130.2, 125.2 (q, J = 274.0 Hz), 42.2, 41.5; **^{19}F NMR (376 MHz, acetone-d^6)** δ_F ppm -59.2; **^{11}B NMR (128 MHz, acetone-d^6)** δ_B ppm 9.6; **FTIR (neat)** ν_{max} / cm^{-1} 3394 (br), 3092 (w), 2957 (w), 2899 (w), 2878 (w), 1655 (s), 1429 (w), 1392 (s), 3120 (s), 1169 (s), 1125 (s), 733 (s), 701 (s); **HRMS (ESI-TOF)** *m/z* [M+Na]$^+$ calculated for $[C_{10}H_{11}{}^{11}BF_3NNaO_3]^+$ 284.0676, found 284.0677.

(2-(Piperidine-1-carbonyl)-6-(trifluoromethyl)phenyl)boronic acid **18**

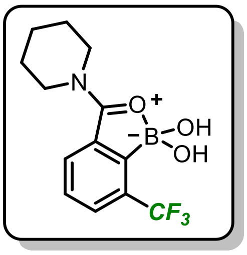

Following general procedure D, using pyrone **2l** (23 mg, 0.11 mmol), **1a** (34 mg, 0.17 mmol) and BCl_3 (0.17 mL, 0.17 mmol) stirring at 40 °C for 30 minutes, the product was purified by precipitation from a saturated acetone solution *via* the addition of petroleum ether (5 x $V_{acetone}$) followed by agitation, then chilling to -20 °C over 30 minutes. The supernatant was decanted, and the resultant solid was washed once with petroleum ether and the remaining solid was dried thoroughly *in vacuo*. The precipitation was performed two times providing **18** as a colourless solid (29 mg, 88%). **M.p.** = 195-196 °C (dec); **^{1}H NMR (400 MHz, CD_3CN)** δ_H ppm 8.28 (d, 1H, J = 8.0 Hz), 7.98 (d, 1H, J = 8.0 Hz), 7.72 – 7.65 (m, 1H), 4.25 – 4.17 (m, 2H), 4.05 – 3.97 (m, 2H), 1.92 – 1.87 (m, 2H), 1.86 – 1.76 (m, 4H); **^{13}C{^{1}H} NMR (101 MHz, acetone-d^6)** δ_C ppm 169.8, 132.5 (q, J = 5.0 Hz), 132.2 (q, J = 34.0 Hz), 131.9, 131.7, 130.2, 125.2 (q, J = 274.0 Hz), 50.3, 26.5, 26.4, 23.8; **^{19}F NMR (376 MHz, acetone-d^6)** δ_F ppm -59.2; **^{11}B NMR (128 MHz, acetone-d^6)** δ_B ppm 9.5; **FTIR (neat)** ν_{max} / cm^{-1} 3484 (br), 3224 (br), 3023 (w), 2975 (w), 2947 (w), 2949 (w), 2864 (w), 1633 (s), 1456 (m), 1385 (s), 1318 (s), 1174 (s), 1093 (s), 734 (s), 701 (s); **HRMS (ESI-TOF)** *m/z* [M+Na]$^+$ calculated for $[C_{13}H_{15}{}^{11}BF_3NNaO_3]^+$ 324.0989, found 324.0989.

[3,6-bis(3,5-dimethyl-1H-pyrazol-1-yl)-5-(trifluoromethyl)pyridazin-4-yl]difluoroborane 20

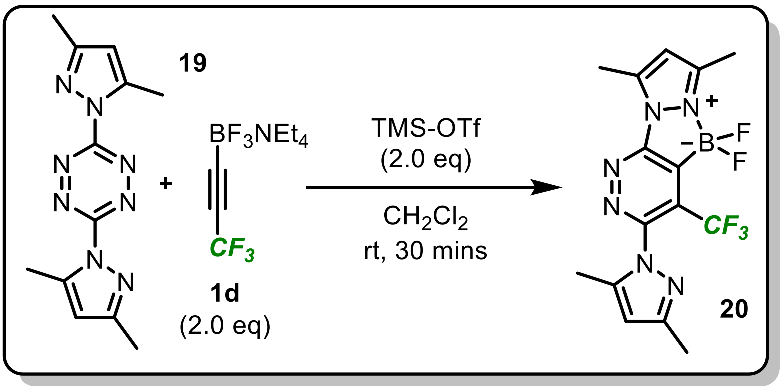

Scheme S9 TMS-OTf promoted cycloaddition of tetrazines.

3,6-Bis(3,5-dimethyl-1*H*-pyrazol-1-yl)-1,2,4,5-tetrazine (**Scheme S9, 19)** was prepared by the published method.[7] The purified product gave satisfactory spectral data.

To a solution of **19** (38 mg, 0.14 mmol) and **1d** (81 mg, 0.28 mmol) in CH_2Cl_2 (1.0 mL) under argon was added dropwise TMS-OTf (52 µL, 0.28 mmol). The reaction mixture was stirred at room temperature (19 °C) for 30 minutes, then diluted with CH_2Cl_2 (10 mL) and washed with saturated aqueous $NaHCO_3$ (2.5 mL). The layers were separated and the aqueous layer was extracted with CH_2Cl_2 (3 x 5 mL). The combined organic layers were dried over anhydrous $MgSO_4$ then concentrated *in vacuo*. The product was purified by flash column chromatography on florisil (eluting with 50% EtOAc in petroleum ether), affording **20** as a colourless solid (30 mg, 56%). **M.p.** = 206 – 207 °C; **^{1}H NMR (400 MHz, CD_3CN)** δ_H ppm 6.53 (s, 1H), 6.13 (s, 1H), 2.87 (s, 3H), 2.51 (s, 3H), 2.25 – 2.23 (m, 3H), 2.23 (s, 3H); **^{13}C{^{1}H} NMR (101 MHz, CD_3CN)** δ_C ppm 159.6, 151.7, 151.6, 150.8, 144.8, 143.6, 131.6 (q, J = 36.5 Hz), 122.8 (q, J = 275.0), 114.4, 108.2, 13.6, 13.1, 11.6, 11.4; **^{19}F NMR (376 MHz, CD_3CN)** δ_F ppm -61.7 (t, J = 7.0 Hz), -153.3 – -154.1 (m); **^{11}B NMR (128 MHz, CD_3CN)** δ_B ppm 3.7 (t, J = 33.5 Hz); **FTIR (neat)** ν_{max} / cm^{-1} 3146 (w), 2930 (w), 1538 (w), 1483 (w), 1428 (w), 1190 (m), 1148 (s); **HRMS (ESI-TOF)** *m/z* [M+H]$^+$ calculated for $[C_{15}H_{15}{}^{11}BF_5N_6]^+$ 385.1366, found 385.1367.

2-(3-(Dichloroboryl)-4-(trifluoromethyl)pyridin-2-yl)pyridine **22**

3-(Pyridin-2-yl)-1,2,4-triazine (**Scheme 5 (main text), 21**) was prepared by the published method.[8] The purified product gave satisfactory spectral data.

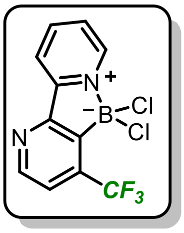

Following general procedure D, using **21** (17 mg, 0.11 mmol), **1a** (33 mg, 0.17 mmol) and BCl_3 solution (0.17 mL, 0.17 mmol) stirring at room temperature (19 °C) for 30 minutes, the product was purified by passing through a small plug of silica gel (eluting with EtOAc), providing **22** as a colourless solid (17 mg, 51%). **M.p.** = 177 – 178 °C; **^{1}H NMR (400 MHz, acetone-d^6)** δ_H ppm 9.10 (d, 1H, J = 5.5 Hz), 8.97 (dd, 1H, J = 0.5, 5.0 Hz), 8.71 (dt, 1H, J = 1.5, 8.0 Hz), 8.58 – 8.52 (m, 1H), 8.16 (ddd, 1H, J = 2.5, 5.5, 9.5 Hz), 7.82 (d, 1H, J = 5.0 Hz); **^{13}C{^{1}H} NMR (101 MHz, acetone-d^6)** δ_C ppm 156.0, 153.3, 152.9, 147.1, 144.3, 140.3 (q, J = 35.0 Hz), 128.6, 124.4 (q, J = 124.5 Hz), 123.3 (q, J = 4.5 Hz), 120.8; **^{19}F NMR (376 MHz, acetone-d^6)** δ_F ppm -62.1; **^{11}B NMR (128 MHz, acetone-d^6)** δ_B ppm 6.1; **FTIR (neat)** ν_{max} / cm^{-1} 3085 (w), 2926 (w), 2855 (w), 1630 (w), 1482 (w), 1314 (s), 1134 (s), 1087 (m), 772 (s); **HRMS (ESI-TOF)** *m/z* [M+Na]$^+$ calculated for $[C_{11}H_6{}^{11}B^{35}Cl_2F_3N_2Na]^+$ 326.9846, found 326.9849.

Synthesis of [1-Phenyl-5-(2-pyridyl)-3-(trifluoromethyl)-4-pyrazolyl]bis(3,3,3-trifluoro-1-propynyl)borane 24

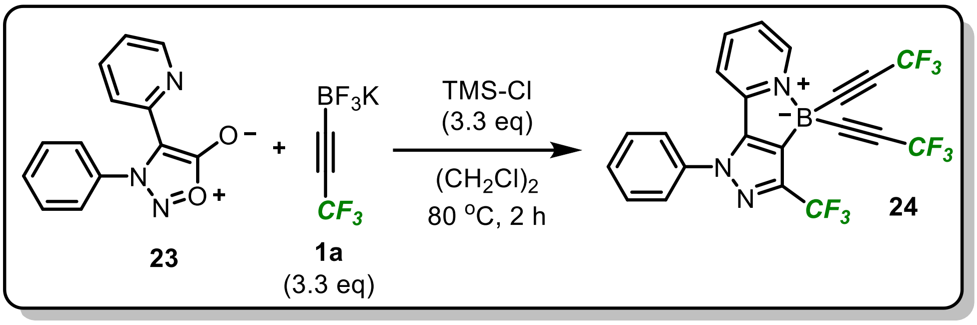

Scheme S10 TMSCl promoted cycloaddition of sydnones.

3-Phenyl-4-(pyridin-2-yl)-3*H*-1,2,3-oxadiazol-1-ium-5-olate (**Scheme S10, 23**) was prepared by the published method.[9] The purified product gave satisfactory spectral data.

To a suspension of **23** (26 mg, 0.11 mmol) and **1a** (72 mg, 0.36 mmol) in 1,2-dichloroethane (1.0 mL) at 50 °C under argon was added dropwise TMS-Cl (46 μL, 0.36 mmol). The mixture was sealed and heated at 80 °C for 2 hours, then cooled to room temperature and concentrated *in vacuo*. The crude product was purified by flash column chromatography on silica gel (gradient elution, 0-60% EtOAc in petroleum ether), affording **24** as a tan solid (33 mg, 62%). **M.p.** = 180 – 183 °C; **^{1}H NMR (400 MHz, $CDCl_3$)** δ_H ppm 8.76 (td, 1H, J = 1.0, 6.0 Hz), 8.11 (dt, 1H, J = 1.5, 8.0 Hz), 7.73 – 7.58 (m, 6H), 7.47 (td, 1H, J = 1.0, 8.0 Hz); **^{13}C{^{1}H} NMR (101 MHz, $CDCl_3$)** δ_C ppm 146.4, 146.1, 143.9, 143.8 (q, J = 40.0 Hz), 138.4, 131.1, 130.3, 130.1, 125.1, 124.2, 121.2 (q, J = 271.0 Hz), 118.5, 113.8 (q, J = 256.5 Hz), 83.1 (q, J = 50.0 Hz); **^{19}F NMR (376 MHz, $CDCl_3$)** δ_F ppm -50.0, -62.4; **^{11}B NMR (128 MHz, $CDCl_3$)** δ_B ppm -13.1; **FTIR (neat)** ν_{max} / cm^{-1} 2918 (w), 2230 (w), 1629 (m), 1506 (m), 1250 (s), 1113 (s); **HRMS (ESI-TOF)** *m/z* $[M+H]^+$ calculated for $[C_{21}H_{10}BF_9N_3]^+$ 486.0819, found 486.0829.

(3) Product functionalisation studies

General procedure E: Suzuki-Miyaura cross coupling of boronic acid 17

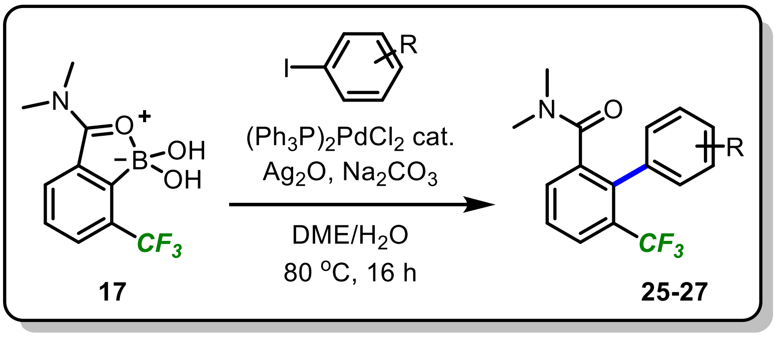

Scheme S11 General method for Suzuki cross-coupling reactions.

To a 10 mL Biotage microwave vial containing **17** (1.2 eq), aryl iodide (1.0 eq), $(Ph_3P)_2PdCl_2$ (10 mol%), Ag_2O (1.2 eq) and Na_2CO_3 (1.2 eq) was added 1,2-dimethoxyethane (0.4 mL) and water (0.4 mL). The mixture was degassed by bubbling a stream of argon through it for 30 minutes, the tube was then

sealed and heated at 80 °C for 16 hours. The mixture was cooled to room temperature, then filtered through a bed of celite (washing with acetone) and concentrated *in vacuo*. The crude product was purified by flash column chromatography on silica gel (gradient elution, 0-60% EtOAc in petroleum ether), then crystallised and dried thoroughly *in vacuo*.

4'-fluoro-N,N-dimethyl-6-(trifluoromethyl)-[1,1'-biphenyl]-2-carboxamide **25**

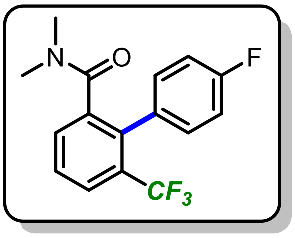

Following general procedure E, using: **17** (29 mg, 0.11 mmol), 4-fluoroiodobenzene (10 µL, 0.09 mmol), $(Ph_3P)_2PdCl_2$ (8 mg, 0.01 mmol), Ag_2O (26 mg, 0.11 mmol) and Na_2CO_3 (12 mg, 0.11 mmol), the product was purified by recrystallisation from petroleum ether affording **25** as colourless crystals (27 mg, 96%). **M.p.** = 145-146 °C; **^{1}H NMR (400 MHz, acetone-d^6)** δ_H ppm 7.87 (ddd, 1H, J = 0.5, 1.5, 8.0 Hz), 7.68 (td, 1H, J = 1.5, 8.0 Hz), 7.59 (ddd, 1H, J = 0.5, 1.5, 8.0 Hz), 7.35 (br, 2H), 7.20 – 7.11 (m, 2H), 2.70 (s, 3H), 2.68 (s, 3H); **^{13}C{^{1}H} NMR (101 MHz, acetone-d^6)** δ_C ppm 168.8, 163.5 (d, J = 245.0 Hz), 141.6, 137.3 (q, J = 2.0 Hz), 133.3 (br), 133.0 (d, J = 3.5 Hz), 131.7 (br), 130.7, 129.9 (q, J = 29.5 Hz), 129.3, 127.0 (q, J = 5.5 Hz), 125.0 (q, J = 273.5 Hz), 115.0 (br, 2C), 38.6 (d, J = 3.0 Hz), 34.0 (d, J = 3.0 Hz); **^{19}F NMR (376 MHz, acetone-d^6)** δ_F ppm -57.4, -115.7; **FTIR (neat)** ν_{max} / cm^{-1} 3042 (w), 2933 (w), 1635 (s), 1158 (s), 1126 (s); **HRMS (ESI-TOF)** *m/z* $[M+H]^+$ calculated for $[C_{16}H_{14}F_4NO]^+$ 312.1006, found 312.1004.

N,N-dimethyl-6-(trifluoromethyl)-[1,1'-biphenyl]-2-carboxamide **26**

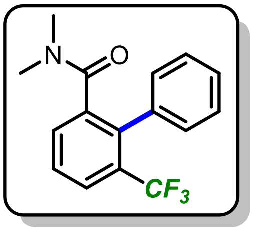

Following general procedure E, using: **17** (29 mg, 0.11 mmol), iodobenzene (10 µL, 0.09 mmol), $(Ph_3P)_2PdCl_2$ (8 mg, 0.01 mmol), Ag_2O (26 mg, 0.11 mmol) and Na_2CO_3 (12 mg, 0.11 mmol), the product was purified by recrystallisation from petroleum ether affording **26** as colourless crystals (18 mg, 68%). **M.p.** = 132-133 °C; **^{1}H NMR (400 MHz, acetone-d^6)** δ_H ppm 7.87 (ddd, 1H, J = 0.5, 1.5, 8.0 Hz), 7.67 (dt, 1H, J = 0.5, 8.0 Hz), 7.58 (ddd, 1H, J = 0.5, 1.5, 8.0 Hz), 7.45 – 7.35 (m, 3H), 7.35 – 7.22 (m, 2H), 2.67 (s, 3H), 2.64 (s, 3H); **^{13}C{^{1}H} NMR (101 MHz, acetone-d^6)** δ_C ppm 168.9, 141.4, 138.3, 136.9, 131.1 (br), 130.7, 129.7 (q, J = 29.5 Hz), 129.6 (br), 129.1, 128.9, 128.2 (br), 128.0 (br), 126.9 (q, J = 5.5 Hz), 125.0 (q, J = 273.5 Hz), 38.6 (d, J = 3.0 Hz), 34.0 (d, J = 3.0 Hz); **^{19}F NMR (376 MHz, acetone-d^6)** δ_F ppm -57.3; **FTIR (neat)** ν_{max} / cm^{-1} 3033 (w), 2931 (w), 1637 (s), 1162 (s), 1116 (s); **HRMS (ESI-TOF)** *m/z* $[M+H]^+$ calculated for $[C_{16}H_{15}F_3NO]^+$ 294.1100, found 294.1097.

N,N,3',5'-tetramethyl-6-(trifluoromethyl)-[1,1'-biphenyl]-2-carboxamide **27**

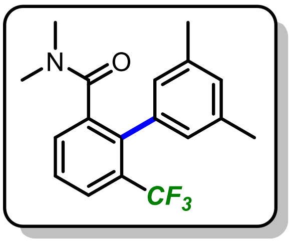

Following general procedure E, using: **17** (29 mg, 0.11 mmol), 3,5-dimethyliodobenzene (13 µL, 0.09 mmol), $(Ph_3P)_2PdCl_2$ (8 mg, 0.01 mmol), Ag_2O (26 mg, 0.11 mmol) and Na_2CO_3 (12 mg, 0.11 mmol), following chromatography on silica gel, the product was further purified by allowing the eluted and concentrated material to stand at room temperature for 48 h, leading to the formation of pale yellow crystals of **27** which were collected and dried *in vacuo* (17 mg, 59%). **M.p.** = 66-67 °C; **^{1}H NMR (400 MHz, $CDCl_3$)** δ_H ppm 7.80 – 7.73 (m, 1H), 7.53 – 7.44 (m, 2H), 6.99 (s, 2H), 6.83 (s, 1H), 2.73 (s, 3H), 2.61 (s, 3H), 2.32 (s, 6H); **^{13}C{^{1}H} NMR (101 MHz, $CDCl_3$)** δ_C ppm 169.6, 139.6, 138.1, 137.4 (br), 136.3 (br), 135.7, 129.9, 129.6 (q, J = 29.5 Hz), 129.6, 127.9 (br), 127.8, 126.7 (q, J = 5.5 Hz), 126.4 (br), 123.9 (q, J = 274.5 Hz), 38.8, 34.4, 21.4; **^{19}F NMR (376 MHz, $CDCl_3$)** δ_F ppm -56.8; **FTIR (neat)** ν_{max} / cm^{-1} 2963

(w), 2930 (w), 2867 (w), 1635 (s), 1178 (s), 1130 (s); **HRMS (ESI-TOF)** *m/z* $[M+H]^+$ calculated for $[C_{18}H_{19}F_3NO]^+$ 322.1413, found 322.1410.

Oxidation of 17: Synthesis of 2-hydroxy-N,N-dimethyl-3-(trifluoromethyl)benzamide 28

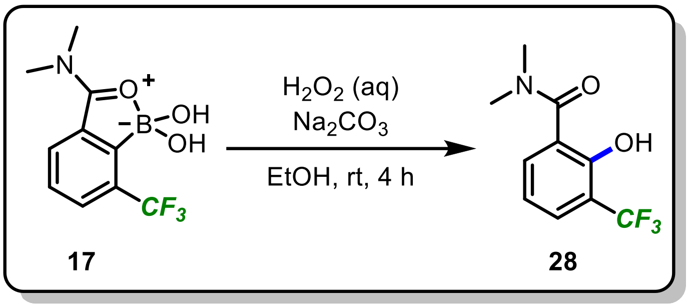

Scheme S12 Boronic acid oxidation.

To a solution of **17** (34 mg, 0.13 mmol) and Na_2CO_3 (14 mg, 0.13 mmol) in EtOH (4.3 mL) under air was added dropwise H_2O_2 (30% v/v aq, 1.7 mL). The mixture was allowed to stir at room temperature for 4 hours, then diluted with H_2O (18 mL) and extracted with CH_2Cl_2 (5 x 18 mL). The combined organic layers were dried over anhydrous $MgSO_4$ then concentrated *in vacuo*. The crude product was purified by passage through a small plug of silica gel, eluting with 50% EtOAc in petroleum ether. The eluate was concentrated *in vacuo* to afford **28** as a colourless solid (25 mg, 82%). **M.p.** = 75-76 °C; **^{1}H NMR (400 MHz, acetone-d^6)** δ_H ppm 11.20 (s, 1H), 7.77 (ddd, 1H, J = 1.0, 1.5, 8.0 Hz), 7.69 (ddd, 1H, J = 1.0, 1.5, 8.0 Hz), 7.06 (dt, 1H, J = 1.0, 8.0 Hz), 3.18 (s, 6H); **^{13}C{^{1}H} NMR (101 MHz, acetone-d^6)** δ_C ppm 171.0, 158.3, 133.9, 130.2, 124.7 (q, J = 271.5 Hz), 120.1, 118.9 (q, J = 31.0 Hz), 118.7, 38.4; **^{19}F NMR (376 MHz, acetone-d^6)** δ_F ppm -63.1; **FTIR (neat)** ν_{max} / cm^{-1} 3201 (br), 2931 (w), 2857 (w), 1635 (s), 1156 (s), 1120 (s); **HRMS (ESI-TOF)** *m/z* $[M+H]^+$ calculated for $[C_{10}H_{11}F_3NO_2]^+$ 234.0736, found 234.0735.

Reduction of 17: Synthesis of 7-(trifluoromethyl)benzo[c][1,2]oxaborole 29

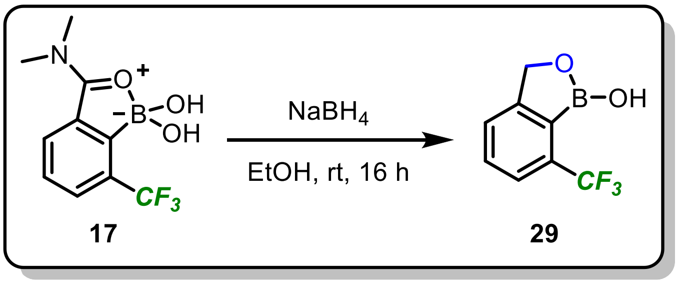

Scheme S13 Reduction to benzoxaboroles.

To a solution of **17** (36 mg, 0.14 mmol) in EtOH (1.2 mL) under argon was added $NaBH_4$ (31 mg, 0.84 mmol) in one portion and the mixture was stirred at room temperature for 16 hours. HCl (1.0 M aq, 2.4 mL) was then added and the mixture was stirred for a further 1 hour, before extracting the mixture with CH_2Cl_2 (5 x 10 mL). The combined organic layers were dried over $MgSO_4$ then concentrated *in vacuo* and the crude product was purified by passage through a small plug of silica gel, eluting with 50% EtOAc in petroleum ether. The eluate was concentrated *in vacuo*, affording **29** as a colourless solid (15 mg, 53%). **M.p.** = 124-125 °C; **^{1}H NMR (400 MHz, $CDCl_3$)** δ_H ppm 7.66 (d, 1H, J = 7.5 Hz), 7.60 (t, 1H, J = 7.5 Hz), 5.16 (s, 1H), 7.55 (d, 1H, J = 7.5 Hz), 5.15 (s, 2H); **^{13}C{^{1}H} NMR (101 MHz, $CDCl_3$)** δ_C

ppm 155.8, 132.5 (q, J = 33.0 Hz), 131.4, 125.0, 124.5 (q, J = 273.0 Hz), 124.4 (q, J = 5.0 Hz), 70.8; **^{19}F NMR (376 MHz, $CDCl_3$)** δ_F ppm -60.7; **^{11}B NMR (128 MHz, $CDCl_3$)** δ_B ppm 31.7; **FTIR (neat)** ν_{max} / cm^{-1} 3336 (br), 2953 (w), 2929 (w), 2855 (w), 1333 (s); **HRMS (ESI-TOF)** *m/z* [M+H]$^+$ calculated for $[C_8H_7{}^{11}BF_3O_2]^+$ 203.0486, found 203.0487.

$^{13}C\{^1H\}$ NMR (101 MHz, acetone-d^6) spectrum of 1c

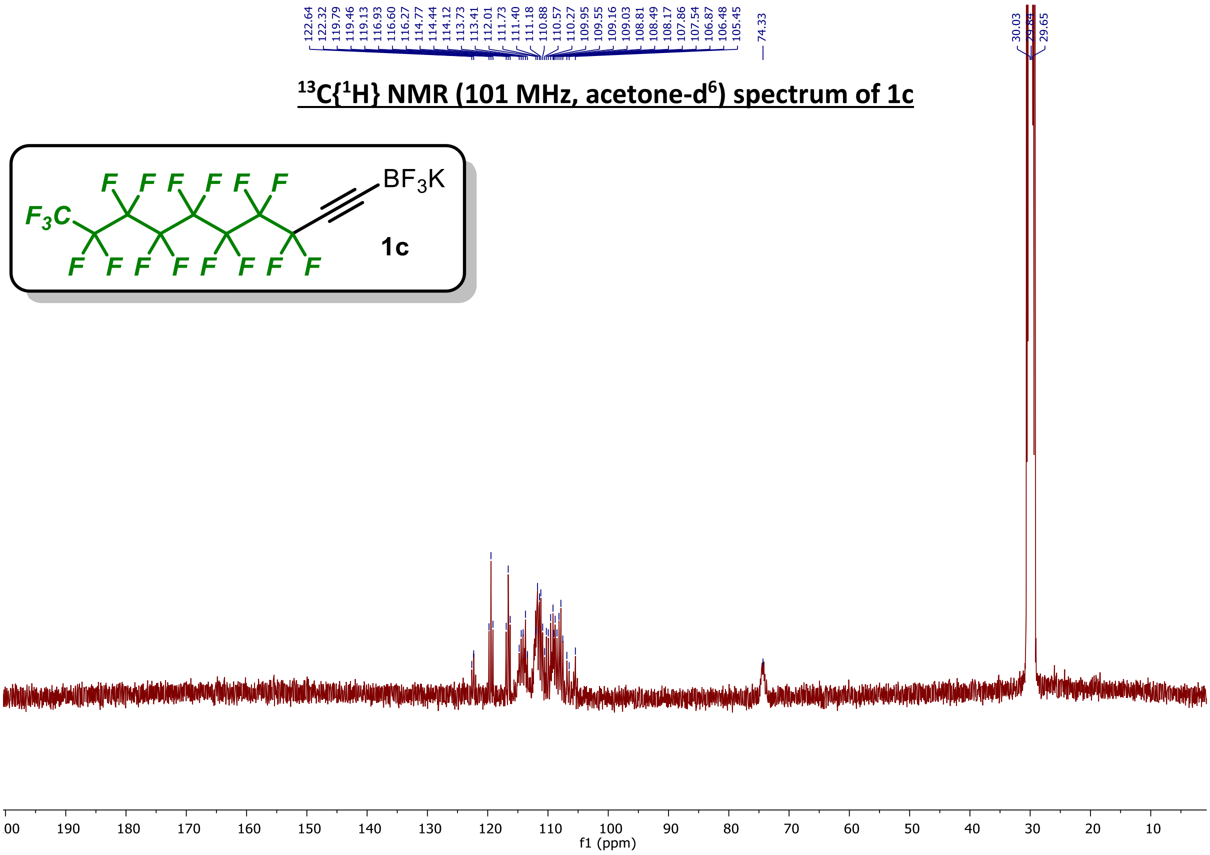

^{19}F NMR (376 MHz, acetone-d^6) spectrum of 1c

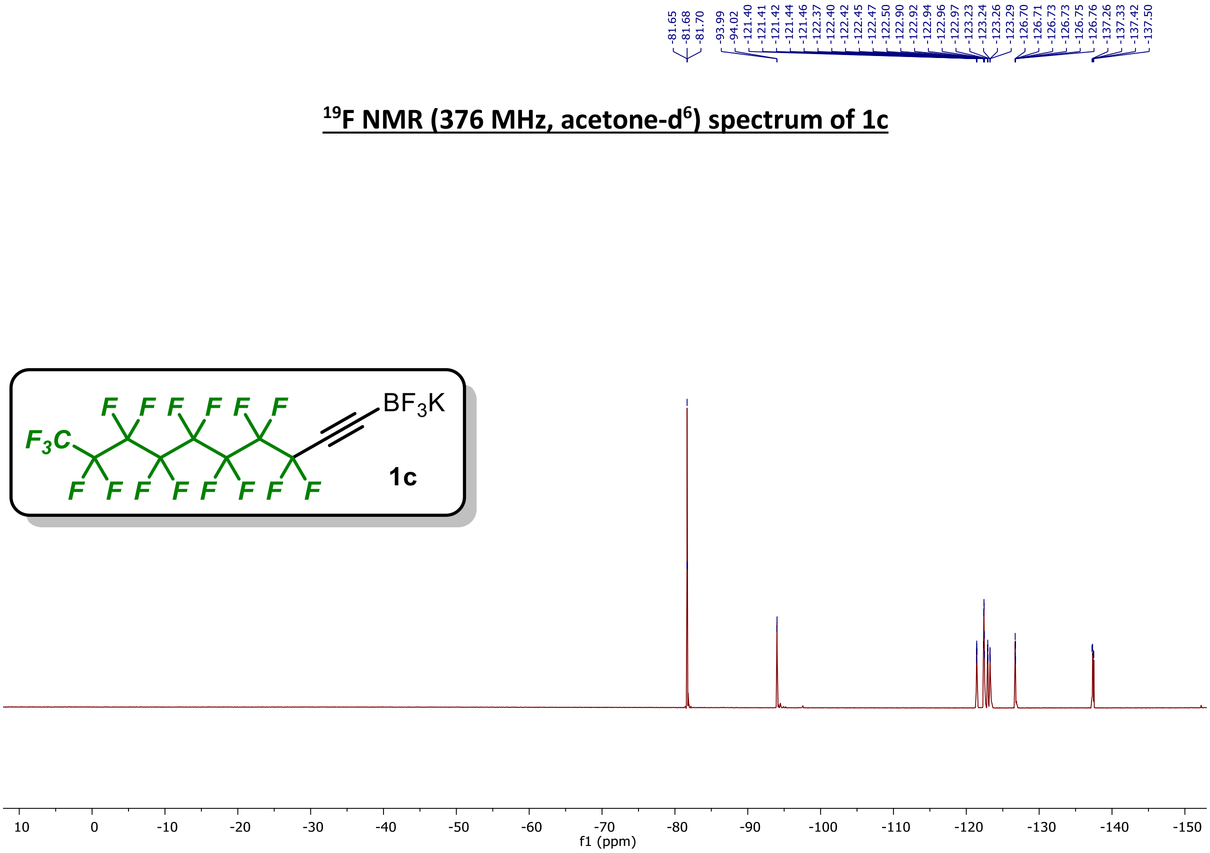

^{11}B NMR (128 MHz, acetone-d^6) spectrum of 1c

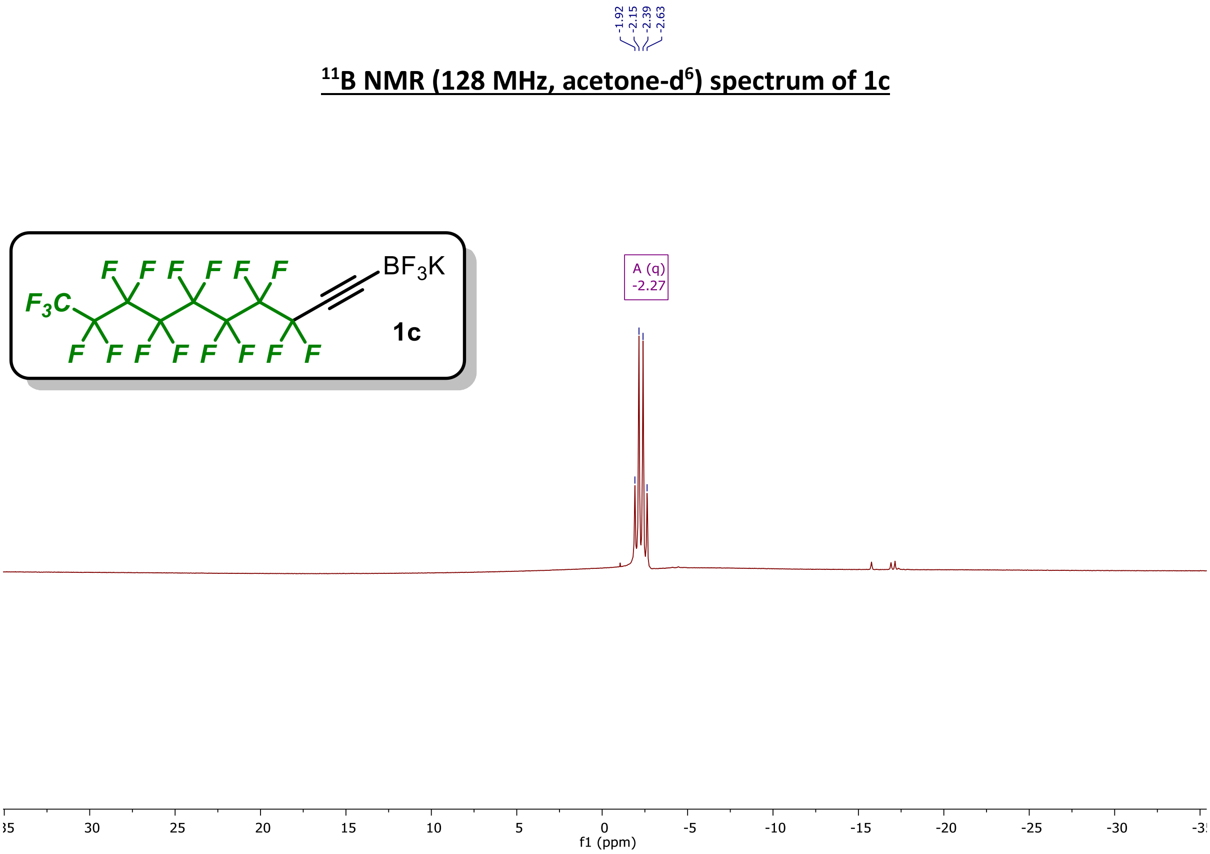

^{1}H NMR (400 MHz, CD_3CN) spectrum of 1d

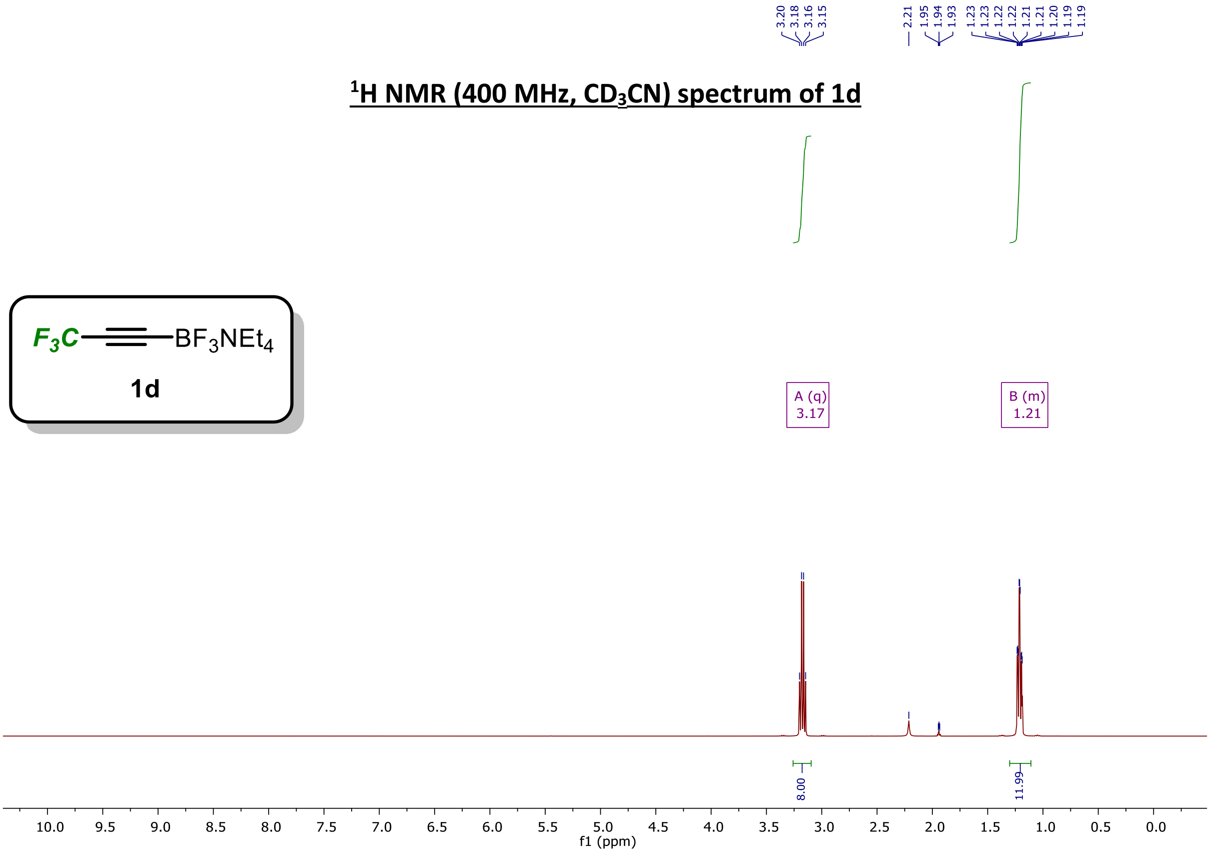

$^{13}C\{^1H\}$ NMR (101 MHz, CD_3CN) spectrum of 1d

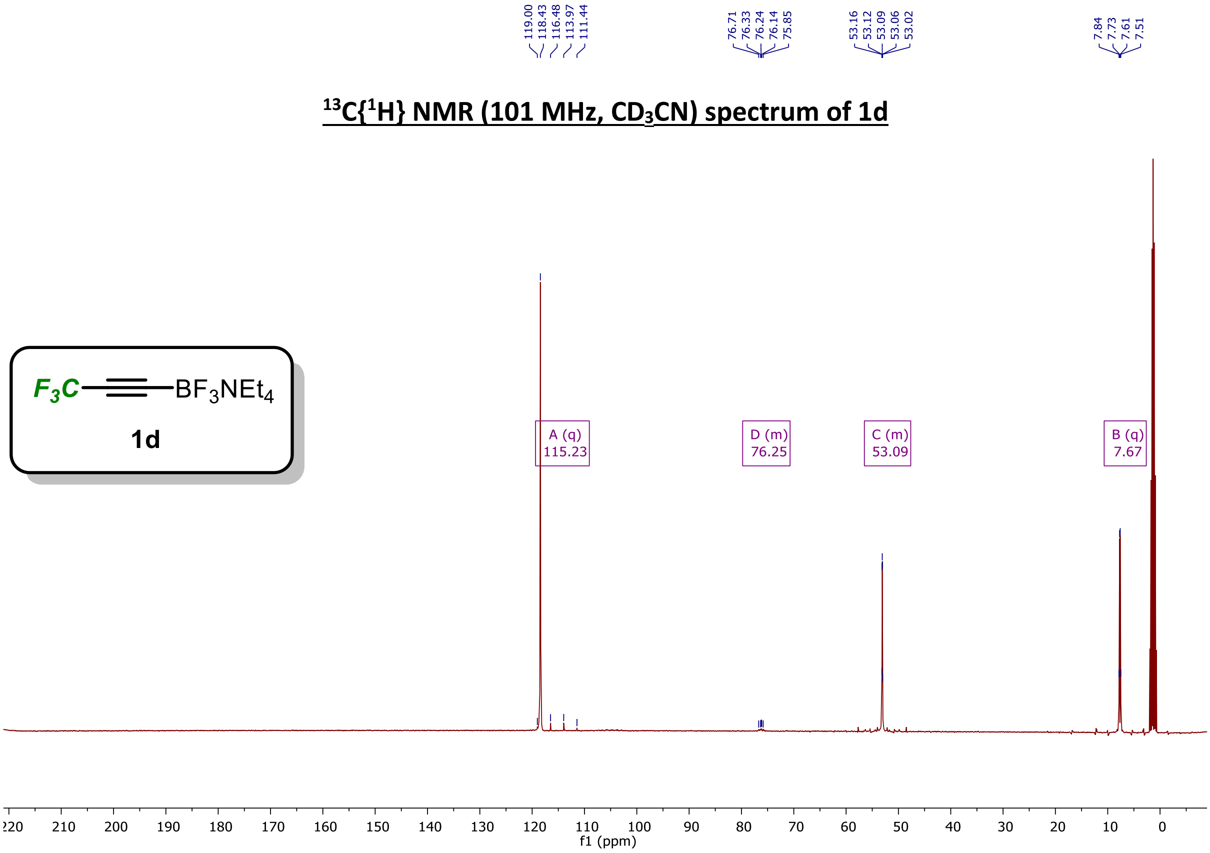

^{19}F NMR (376 MHz, $CDCl_3$) spectrum of 1d

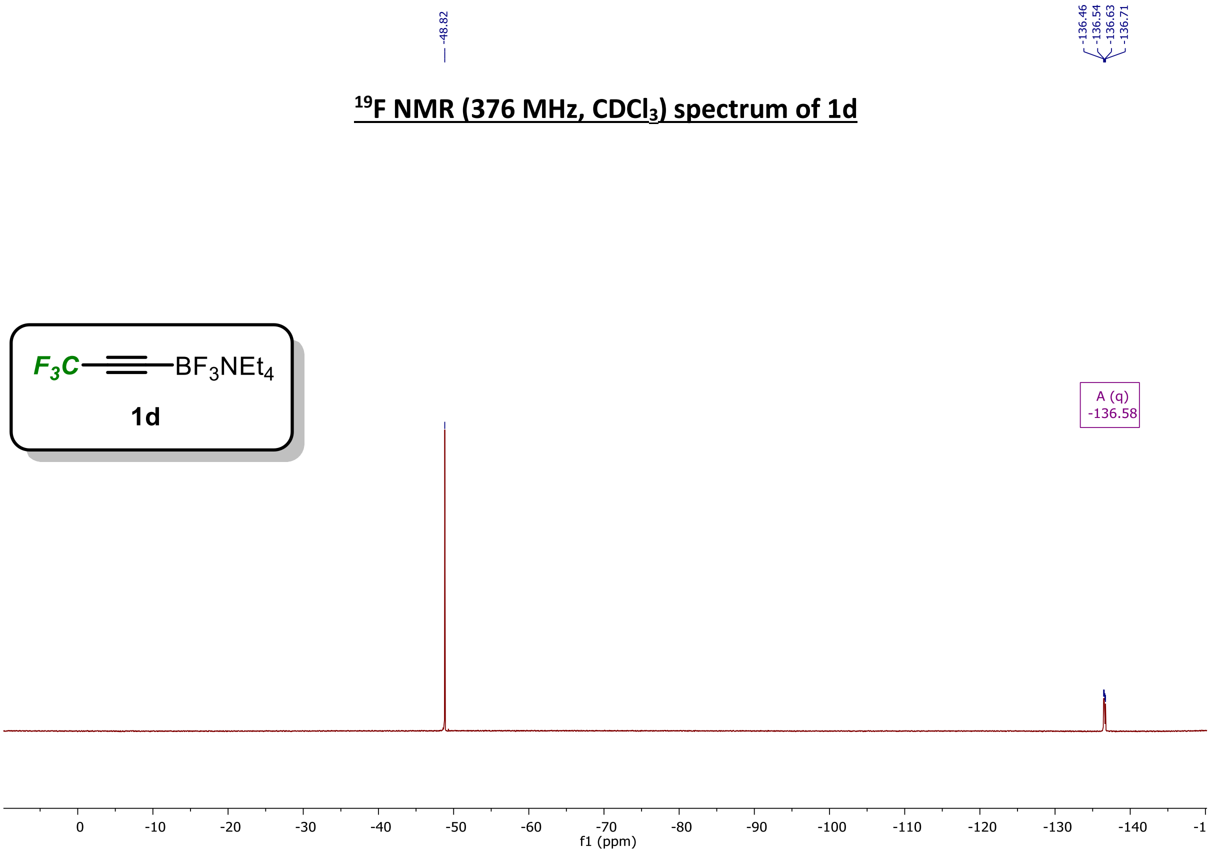

^{11}B NMR (128 MHz, CD_3CN) spectrum of 1d

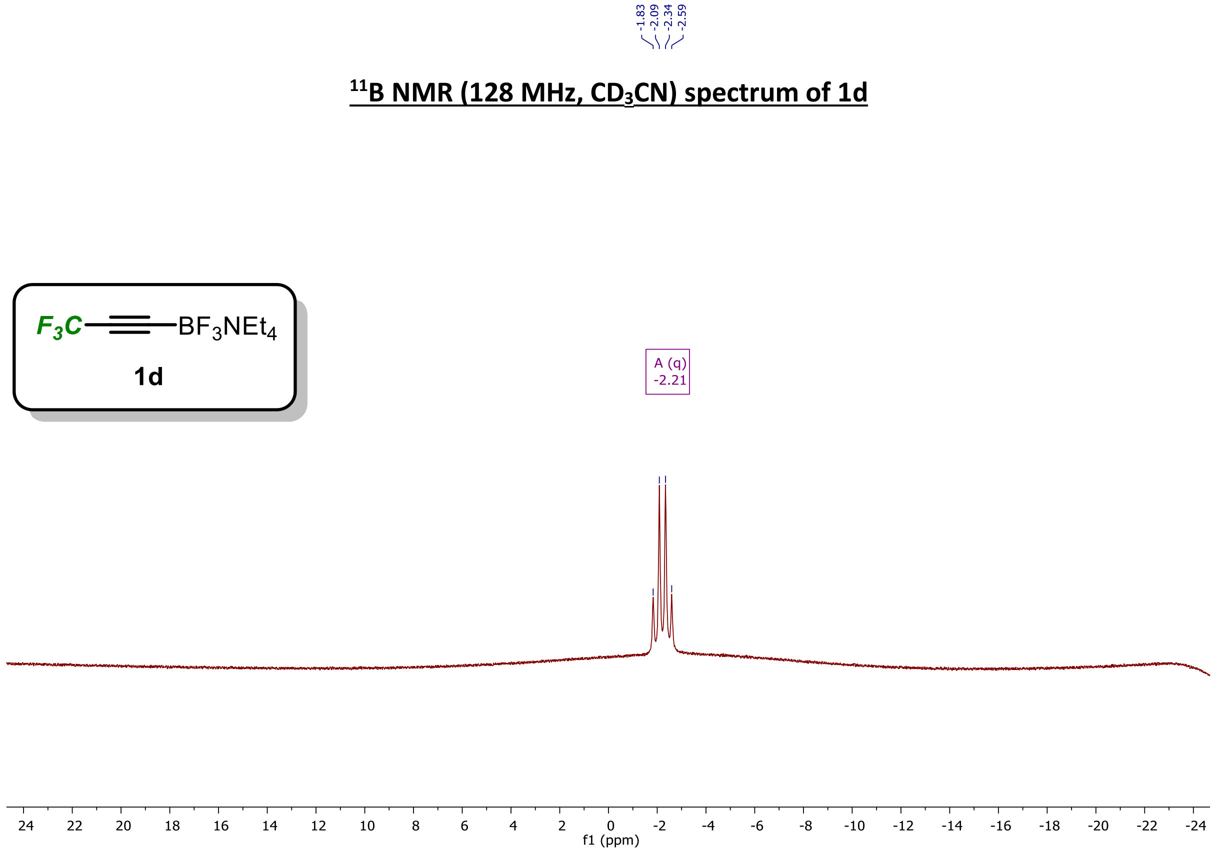

^{1}H NMR (400 MHz, $CDCl_3$) spectrum of 2b

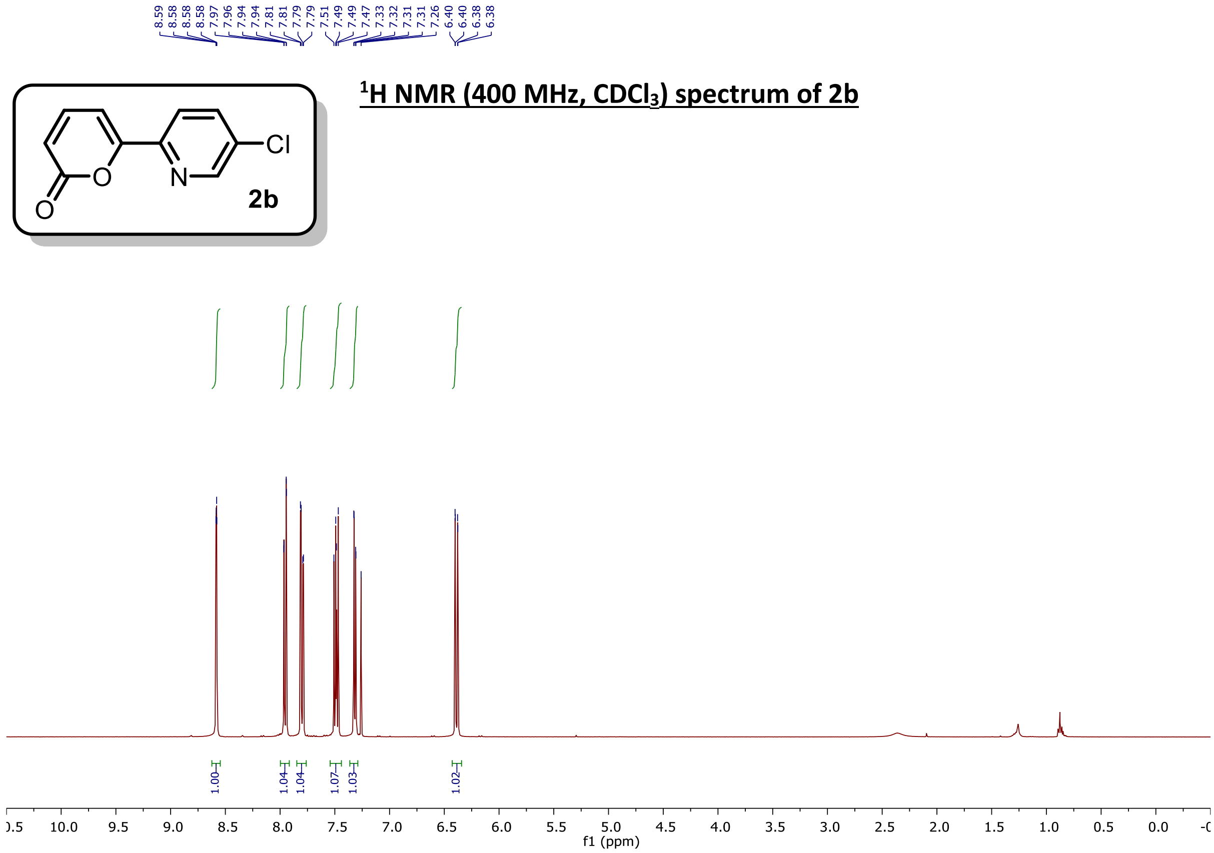

$^{13}C\{^1H\}$ NMR (101 MHz, $CDCl_3$) spectrum of 2b

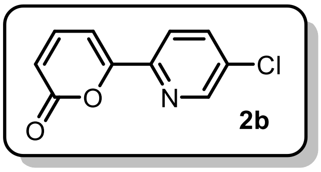

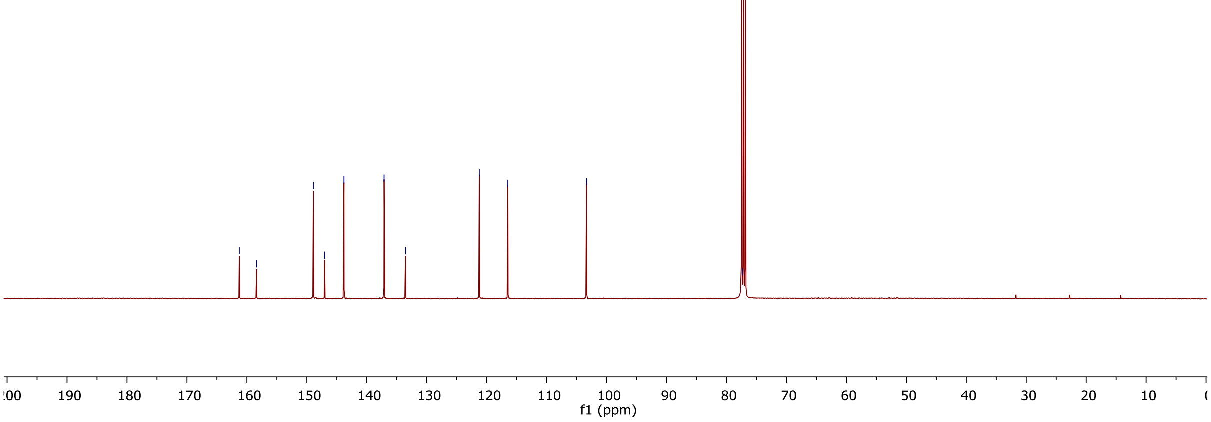

^1H NMR (400 MHz, $CDCl_3$) spectrum of 2d

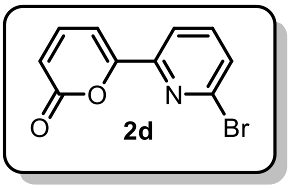

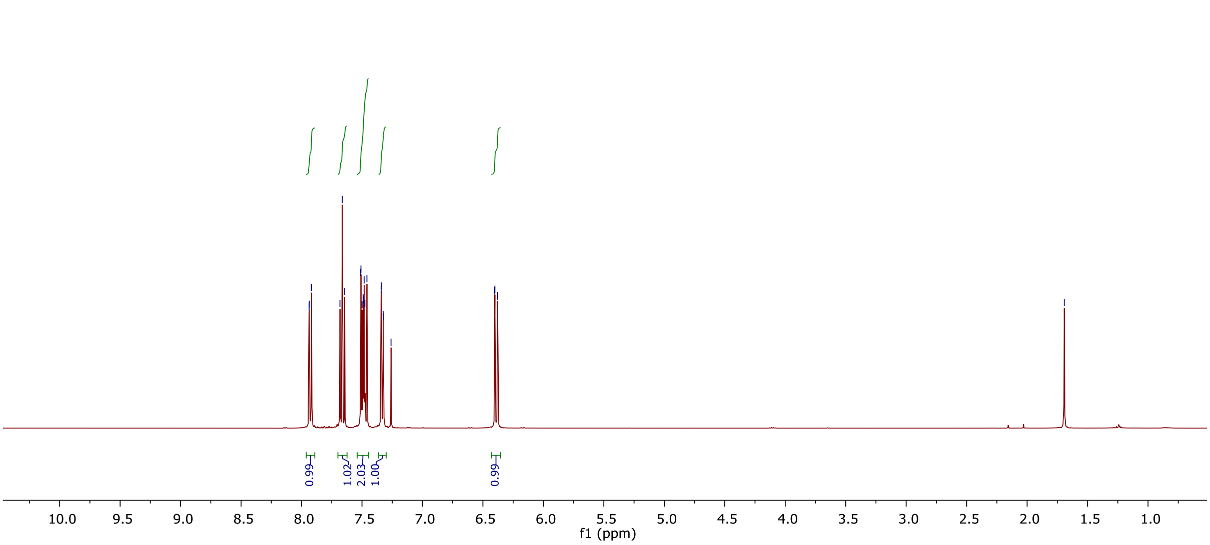

$^{13}C\{^1H\}$ NMR (101 MHz, $CDCl_3$) spectrum of 2d

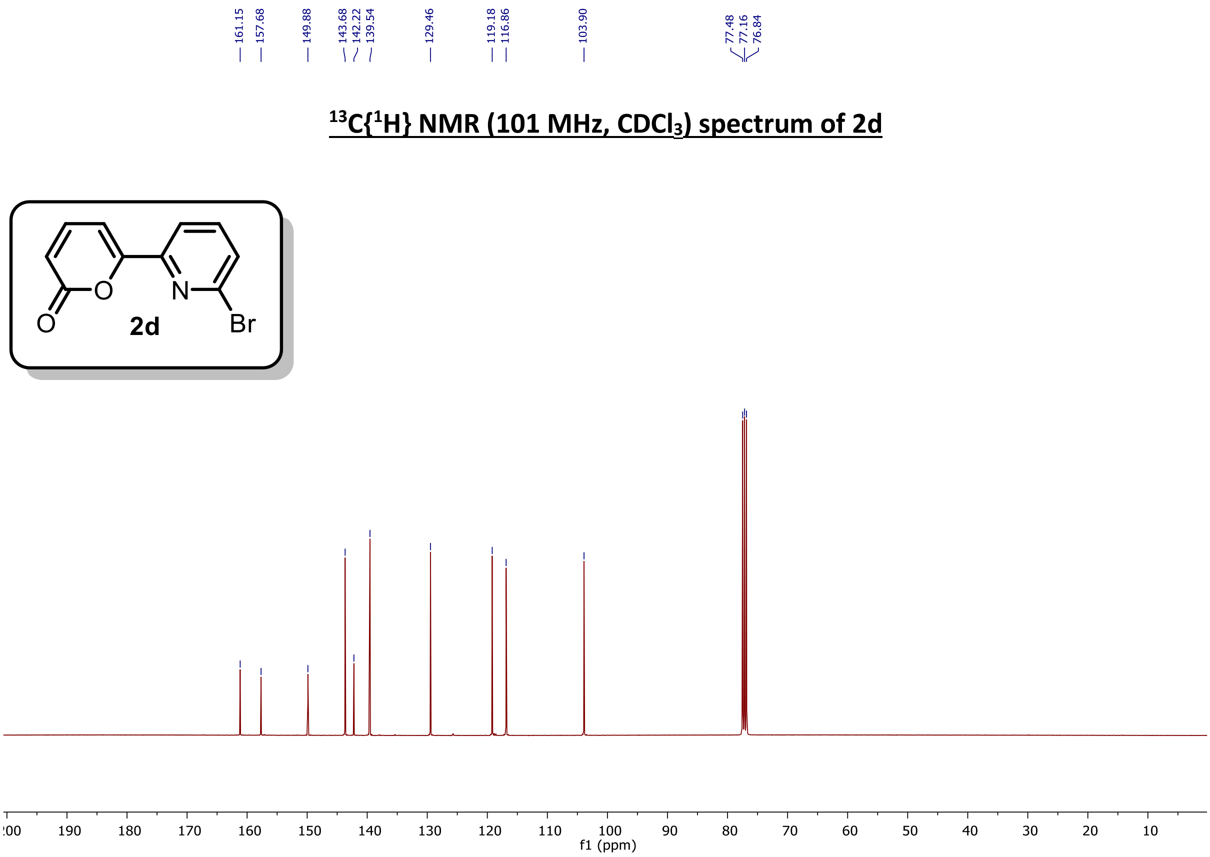

^1H NMR (400 MHz, $CDCl_3$) spectrum of 2e

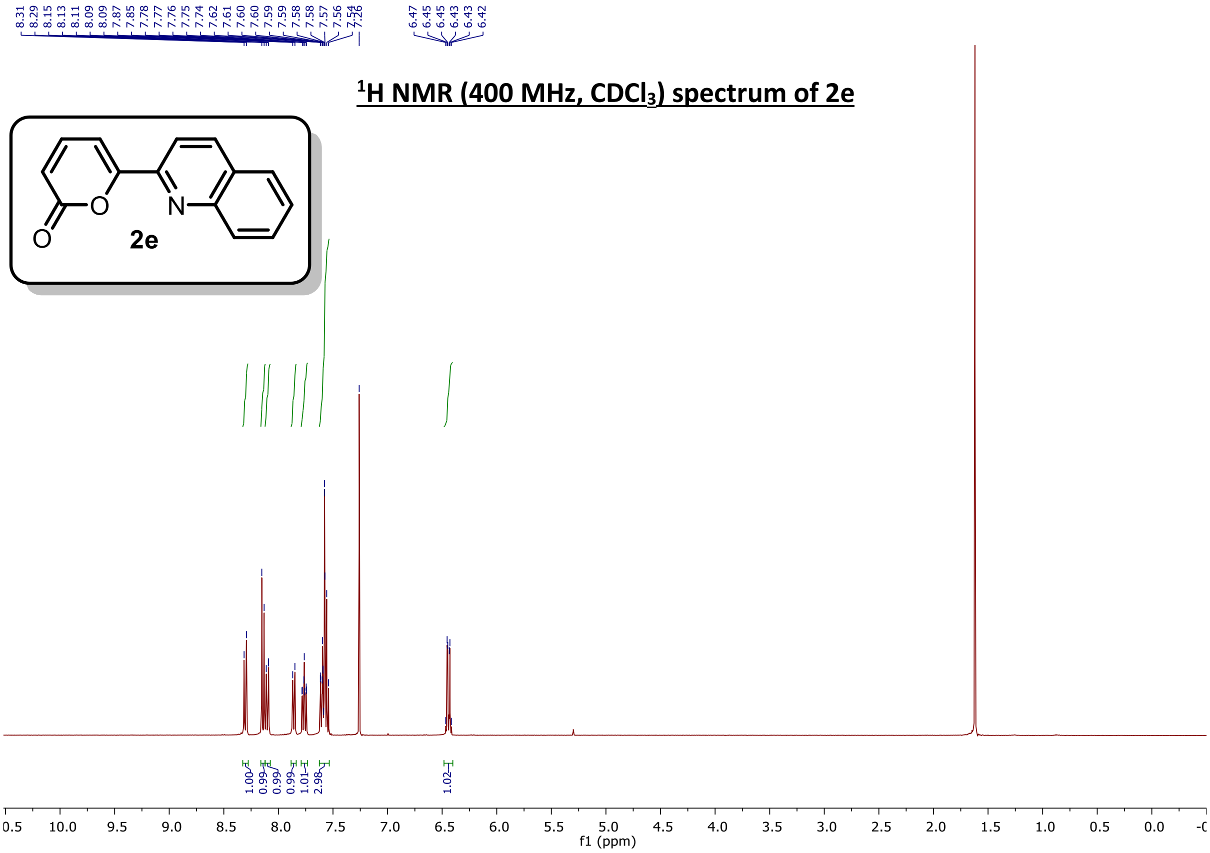

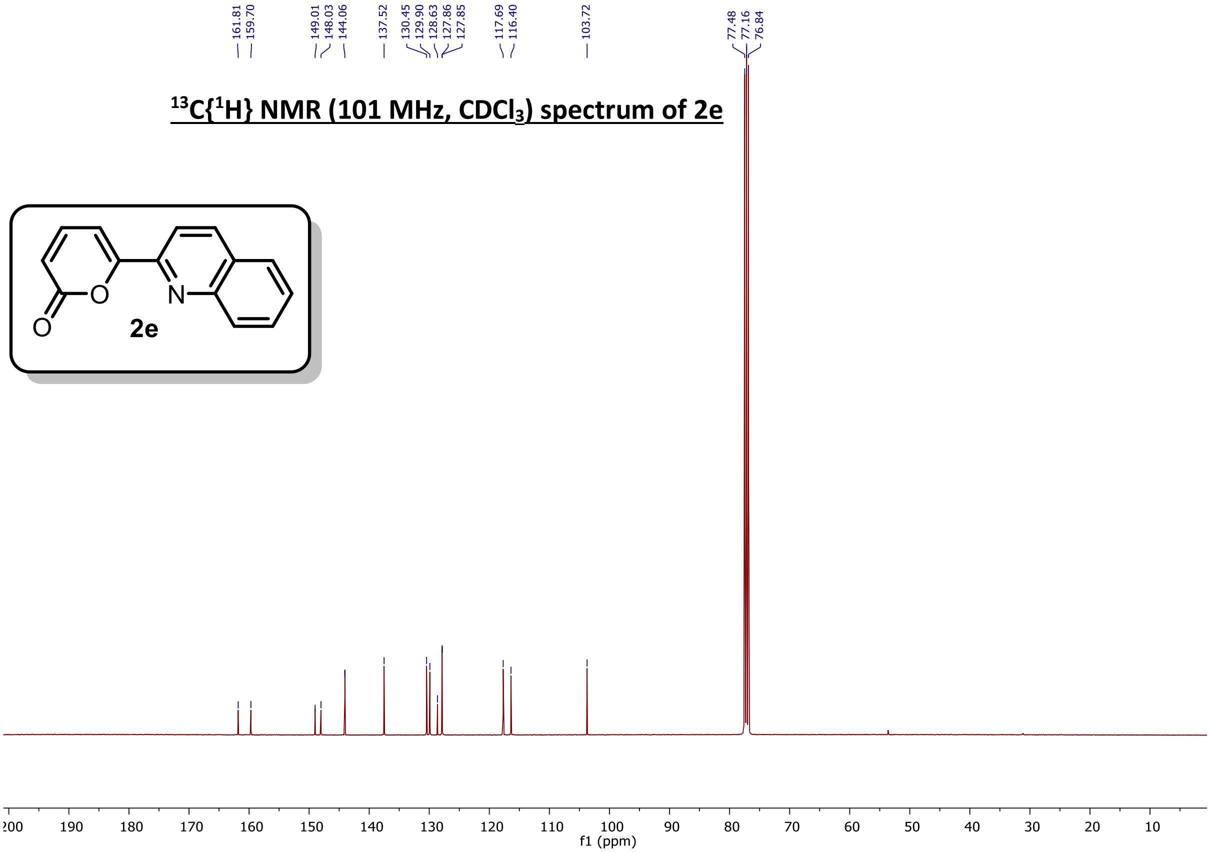

$^{13}C\{^{1}H\}$ NMR (101 MHz, $CDCl_3$) spectrum of 2e

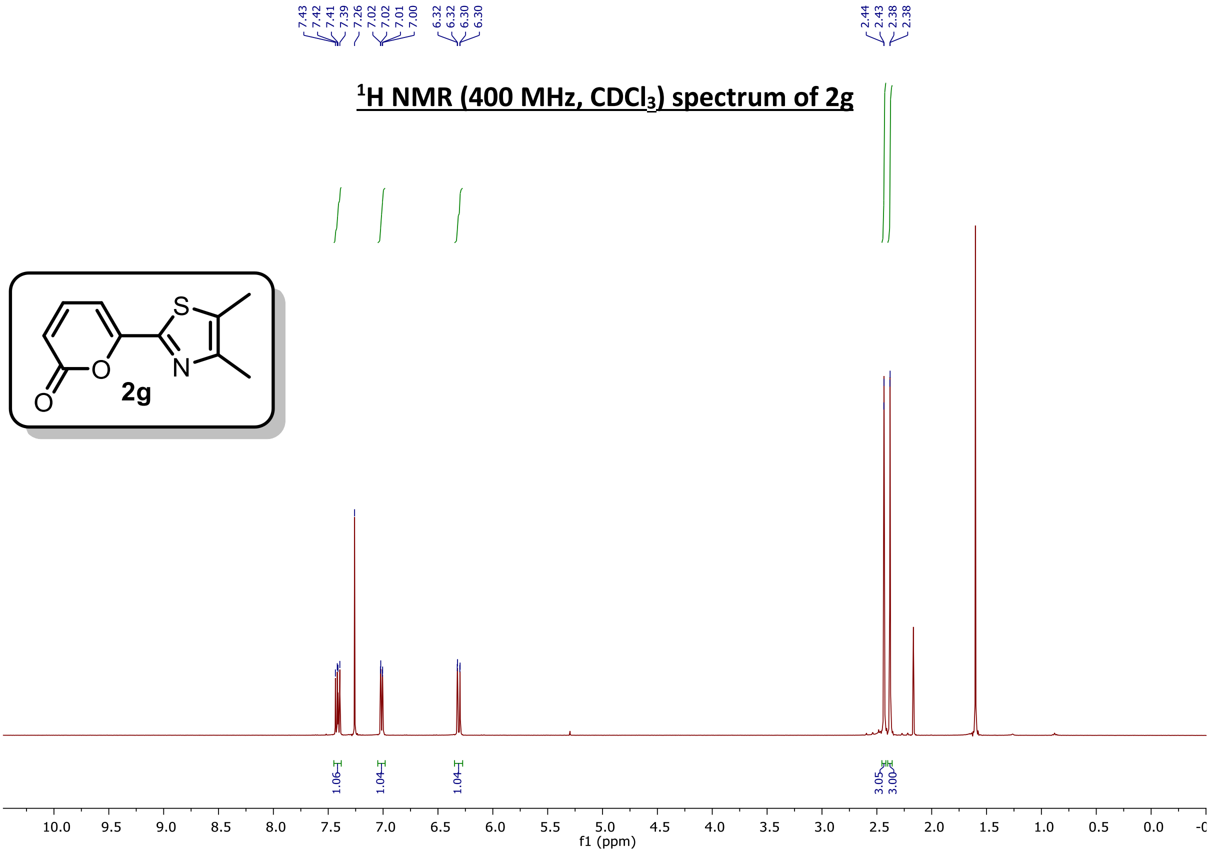

^{1}H NMR (400 MHz, $CDCl_3$) spectrum of 2g

$^{13}C\{^{1}H\}$ NMR (101 MHz, $CDCl_3$) spectrum of 2g

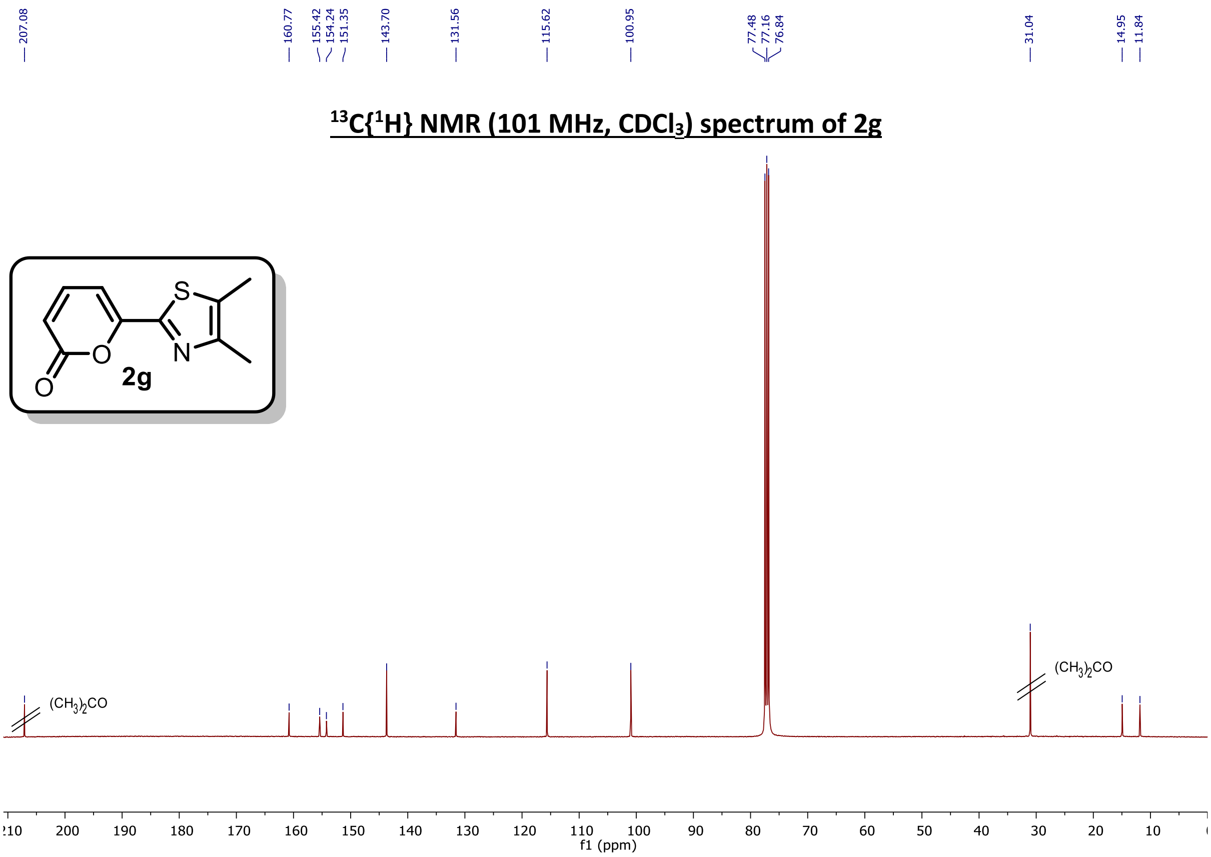

^{1}H NMR (400 MHz, CD_2Cl_2) spectrum of 2h

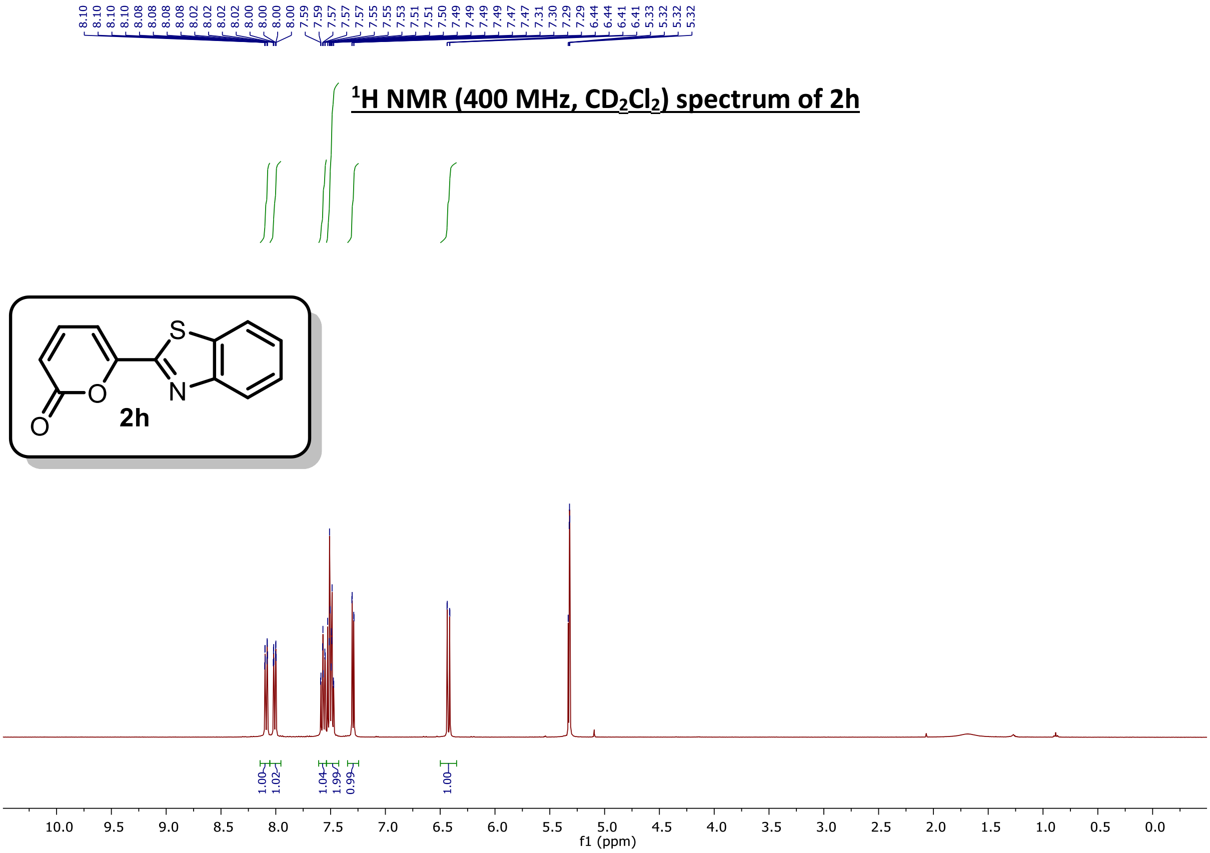

$^{13}C\{^{1}H\}$ NMR (101 MHz, CD_2Cl_2) spectrum of 2h

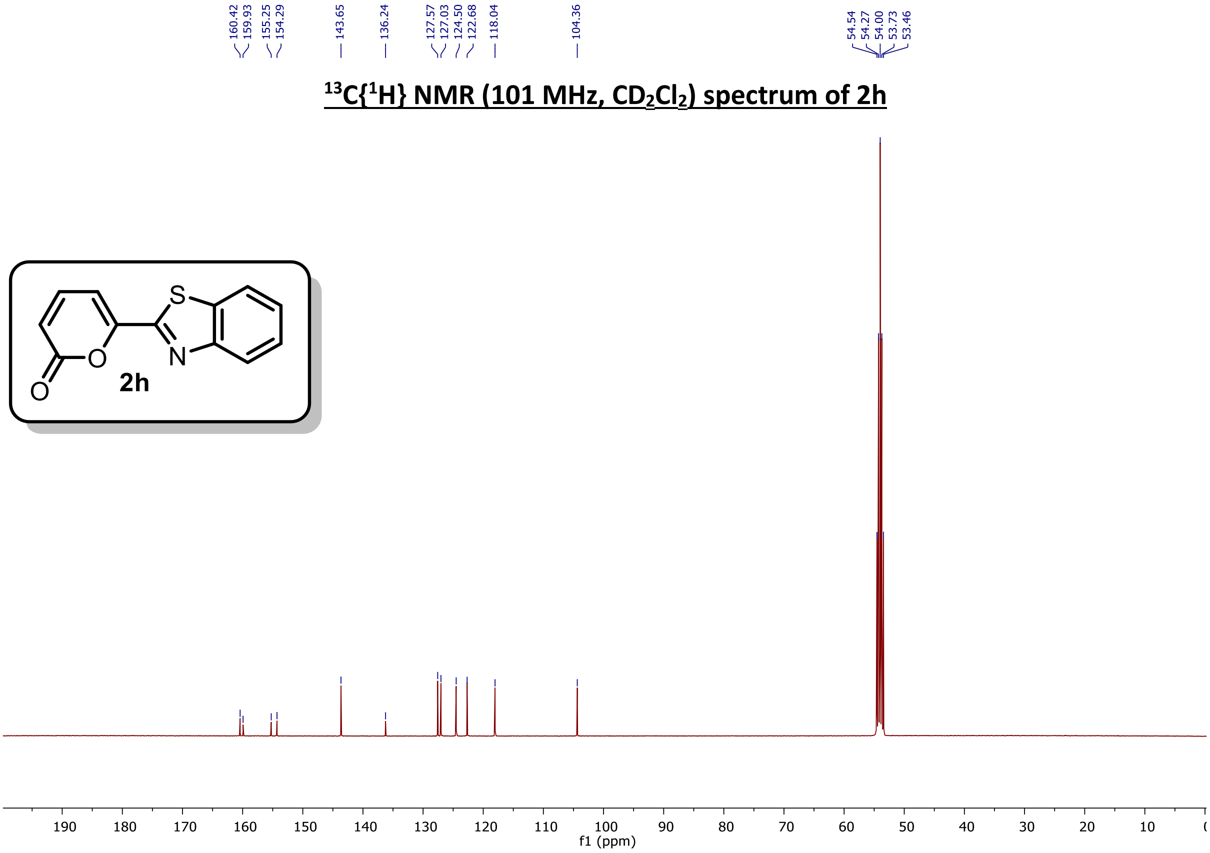

^{1}H NMR (400 MHz, acetone-d^{6}) spectrum of 2l

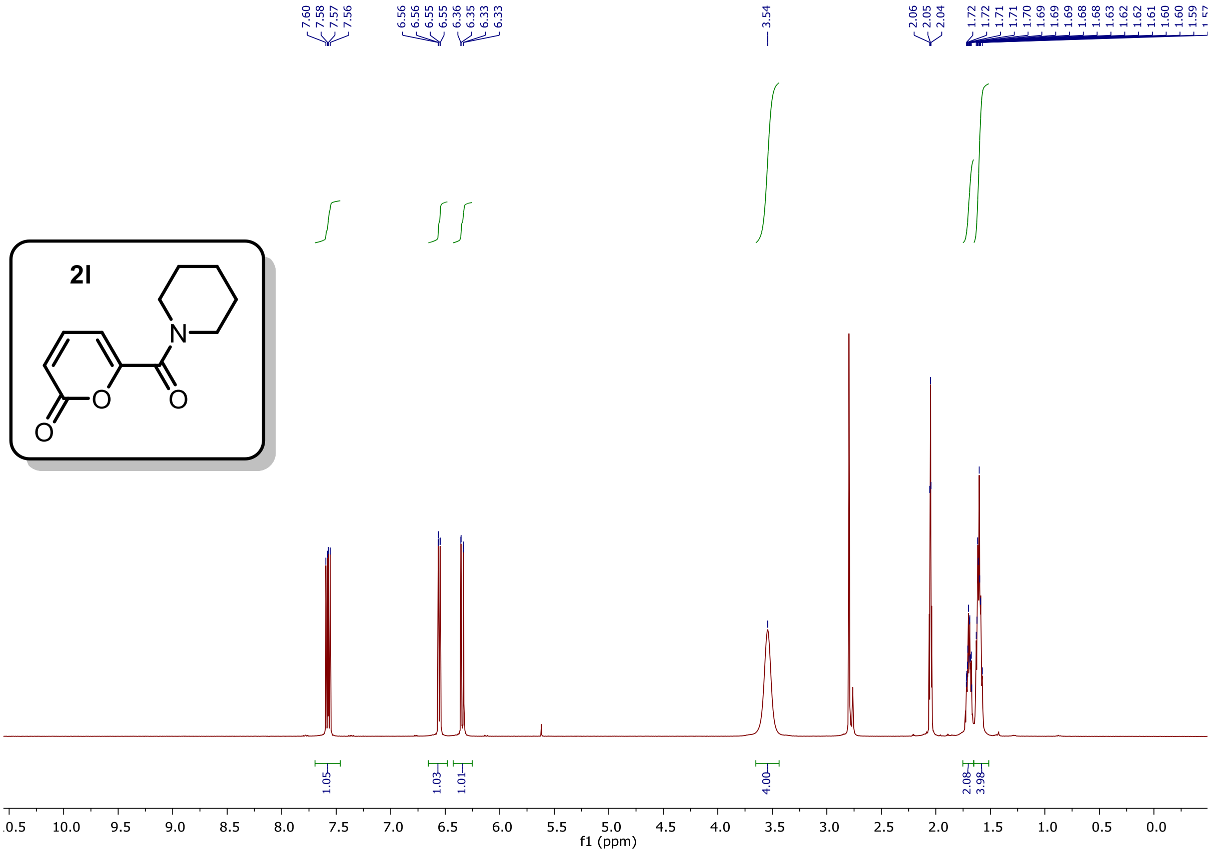

$^{13}C\{^{1}H\}$ NMR (101 MHz, acetone-d^{6}) spectrum of 2l

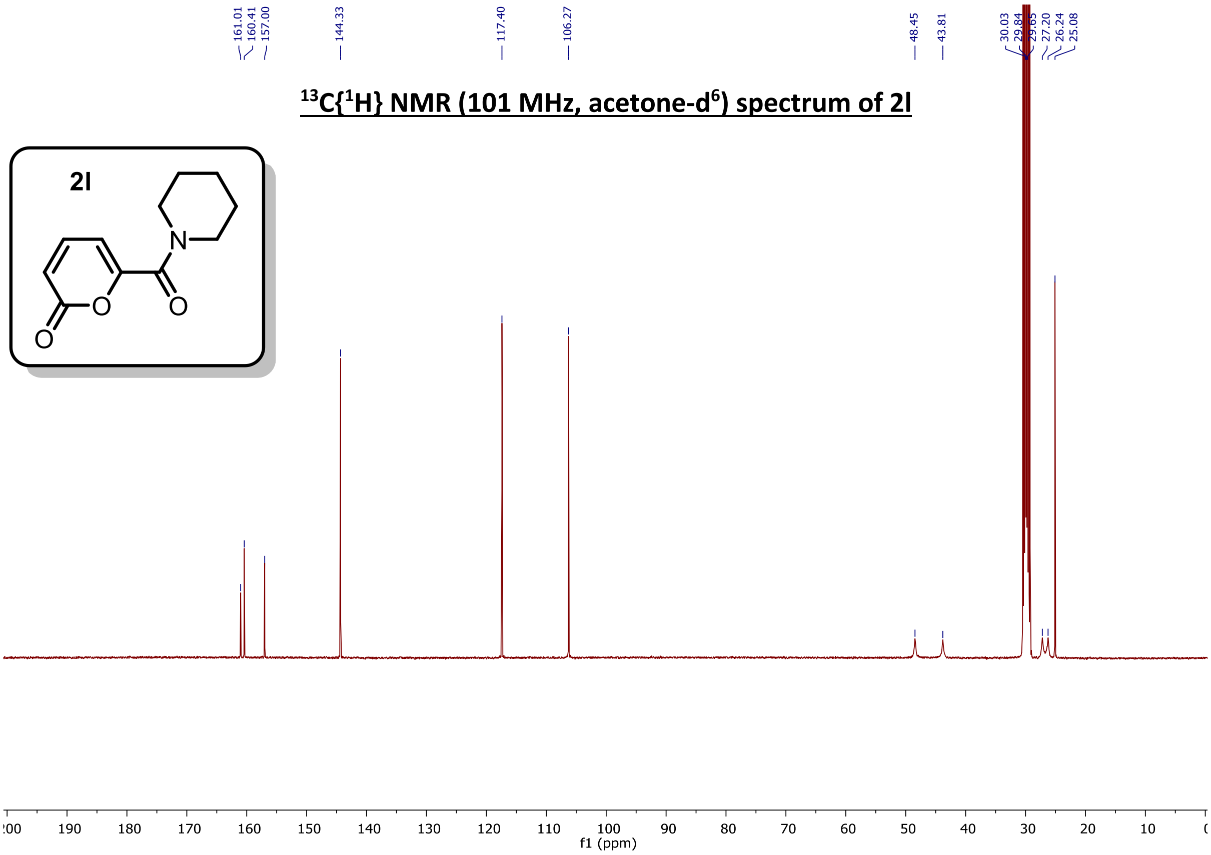

^{1}H NMR (400 MHz, acetone-d^{6}) spectrum of 3a

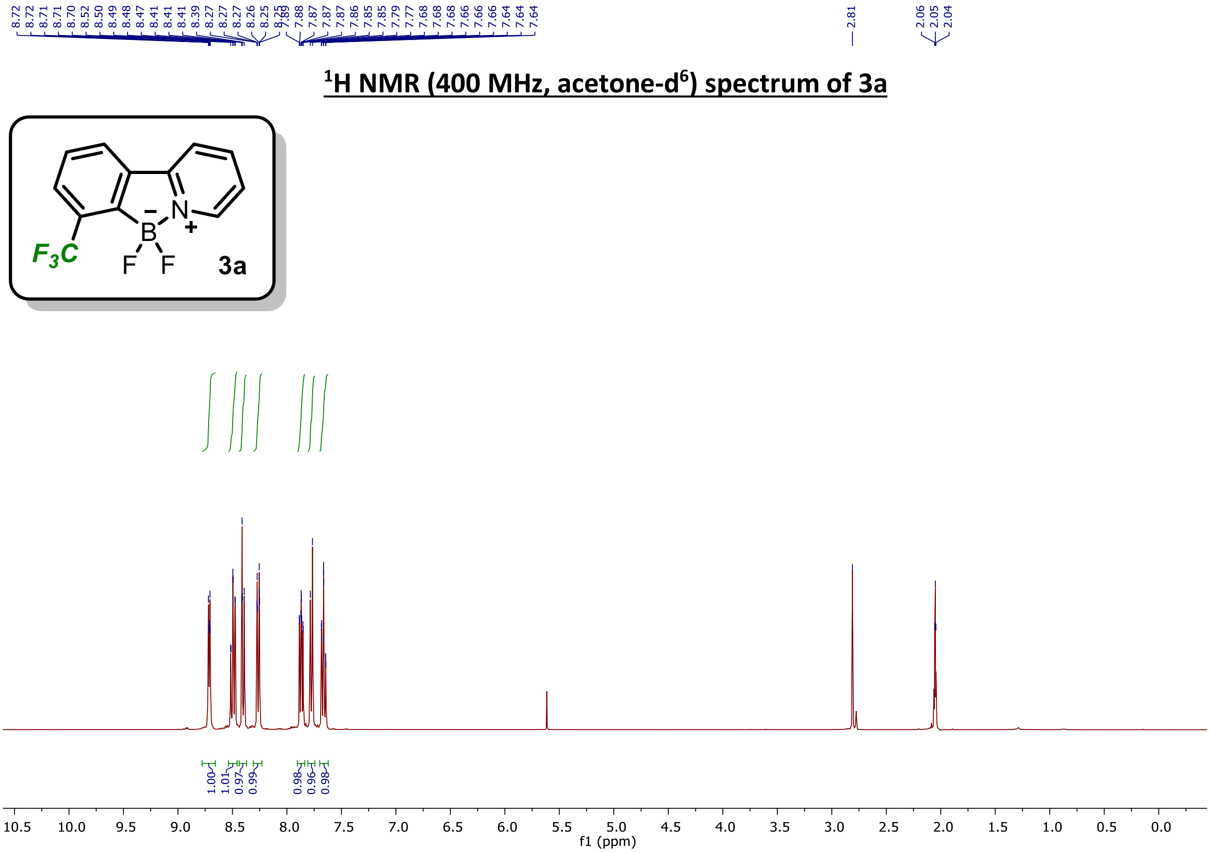

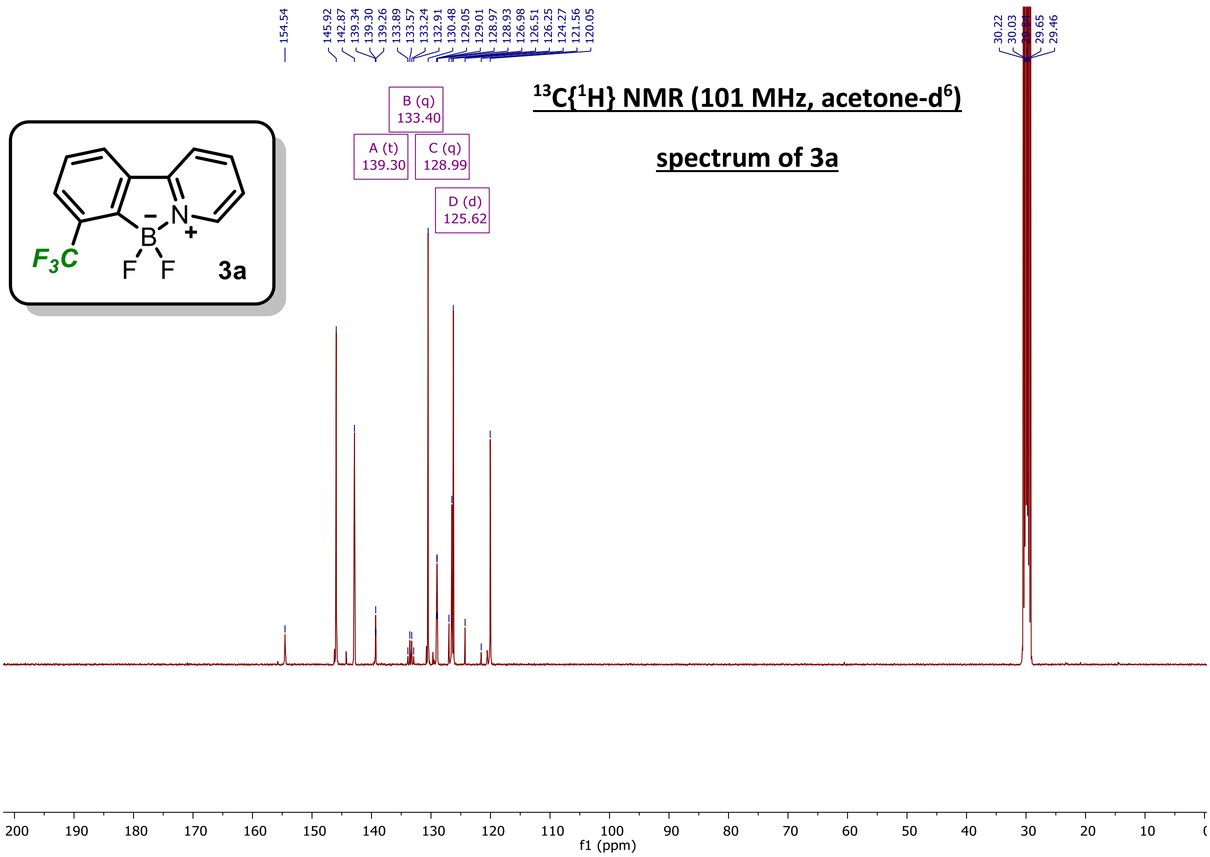
$^{13}C\{^1H\}$ NMR (101 MHz, acetone-d6) spectrum of 3a
3a
F3C
B (q) 133.40
A (t) 139.30
C (q) 128.99
D (d) 125.62
154.54
145.92
142.87
139.34
139.30
139.26
133.89
133.57
133.24
132.91
130.48
129.05
129.01
128.97
128.93
126.98
126.51
126.25
124.27
121.56
120.05
30.22
30.03
29.84
29.65
29.46
f1 (ppm)

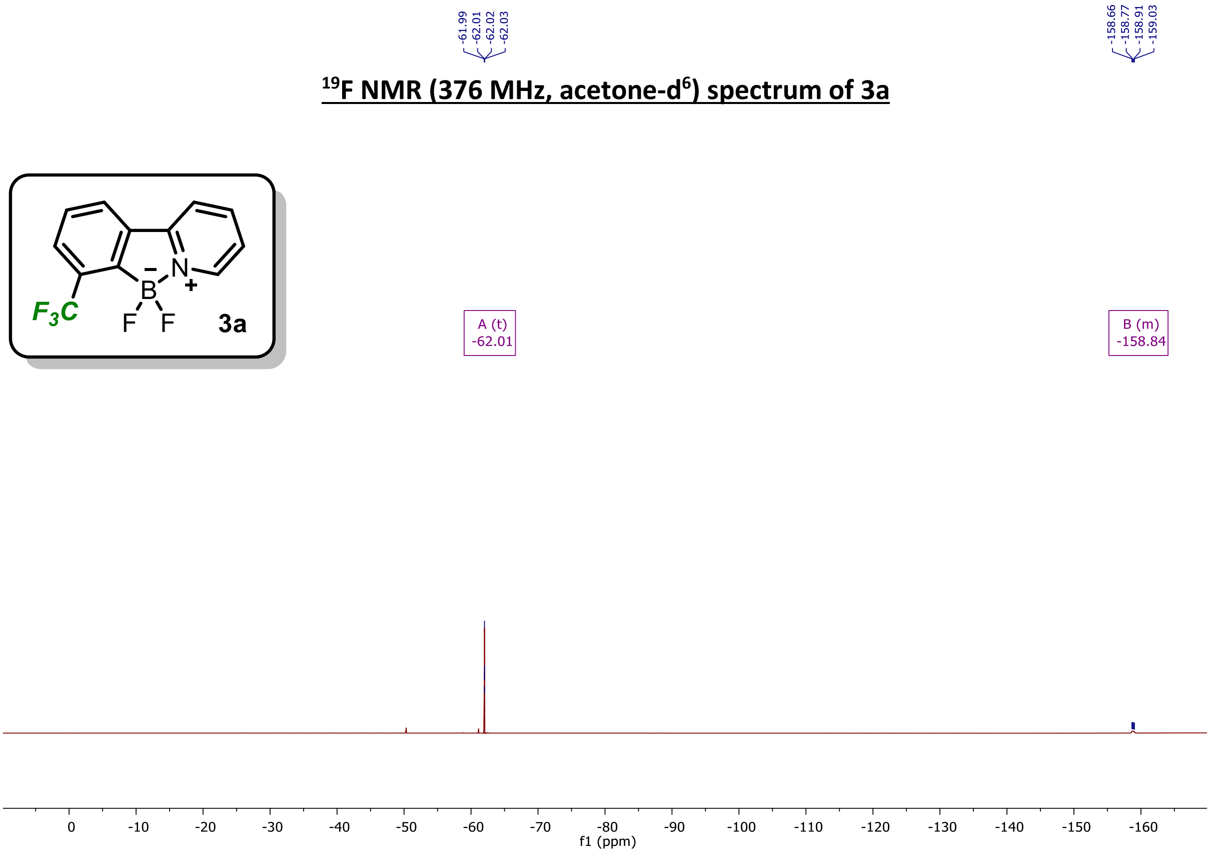
^{19}F NMR (376 MHz, acetone-d6) spectrum of 3a
3a
F3C
A (t) -62.01
B (m) -158.84
-61.99
-62.01
-62.02
-62.03
-158.66
-158.77
-158.91
-159.03
f1 (ppm)

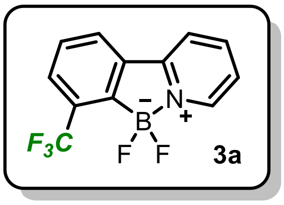

^{11}B NMR (128 MHz, acetone-d^{6}) spectrum of 3a

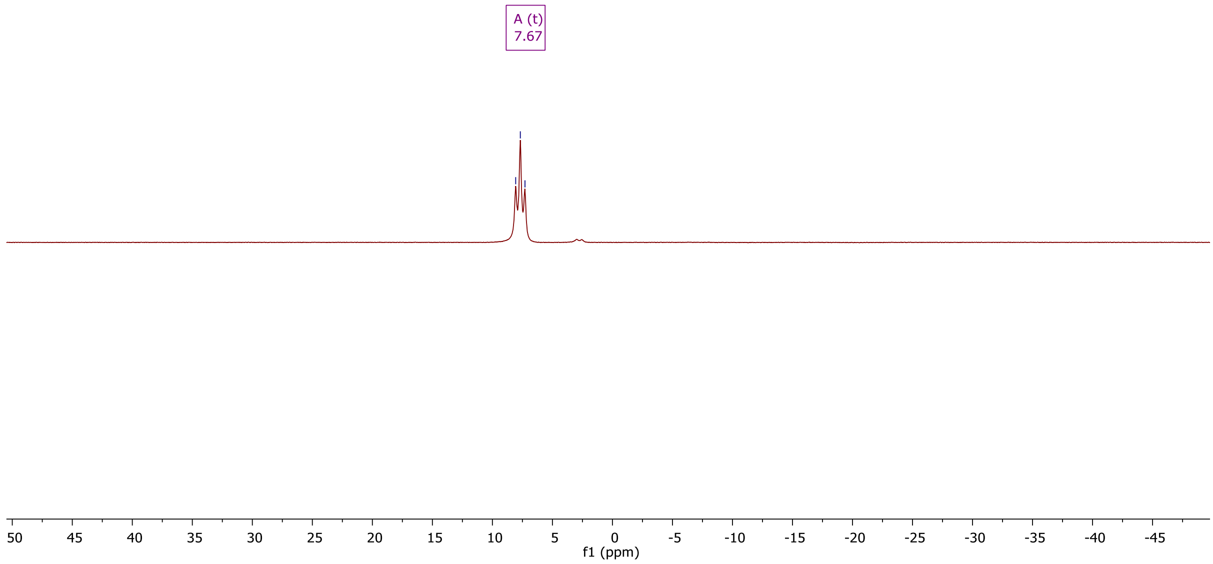

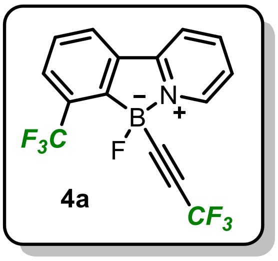

^{1}H NMR (400 MHz, acetone-d^{6}) spectrum of 4a

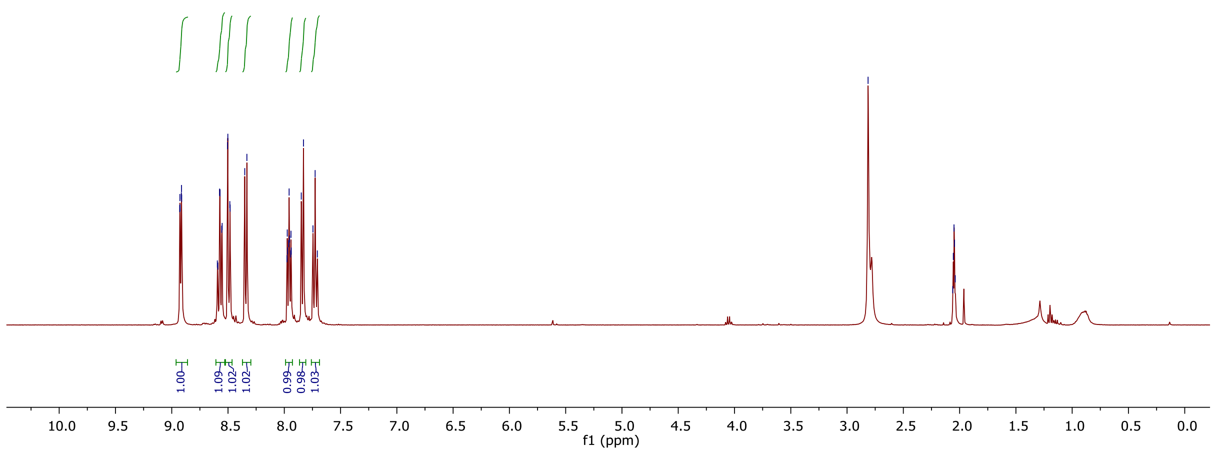

$^{13}C\{^1H\}$ NMR (101 MHz, acetone-d^6) spectrum of 4a

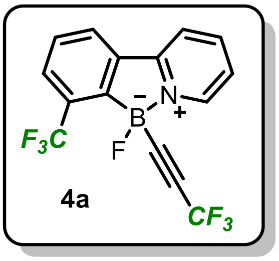

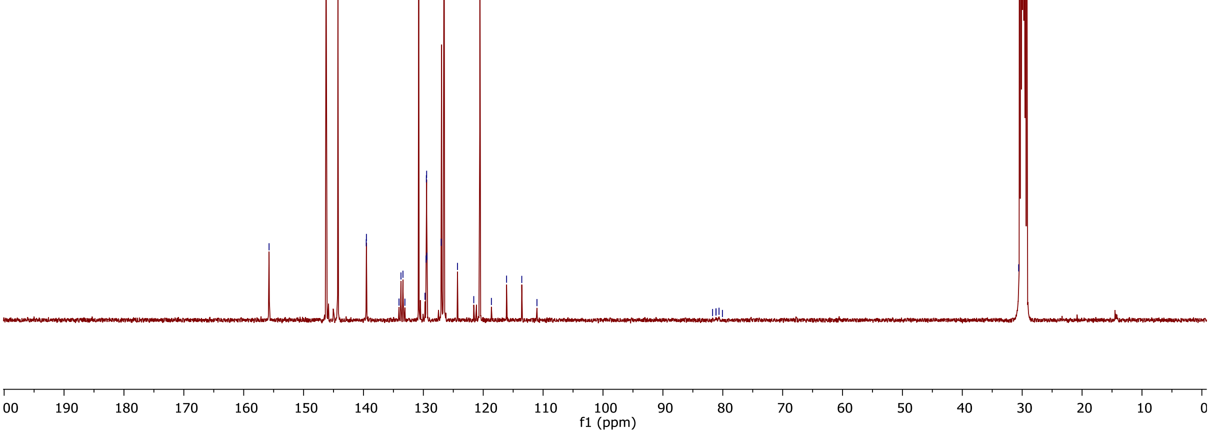

^{19}F NMR (376 MHz, acetone-d^6) spectrum of 4a

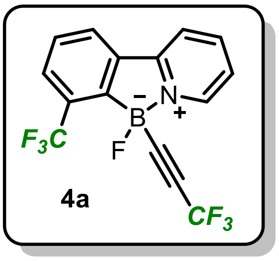

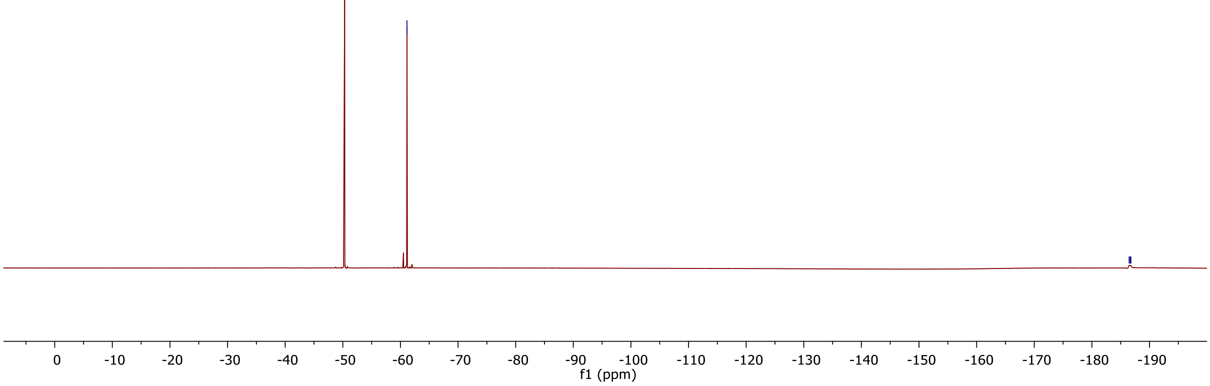

^{11}B NMR (128 MHz, acetone-d^6) spectrum of 4a

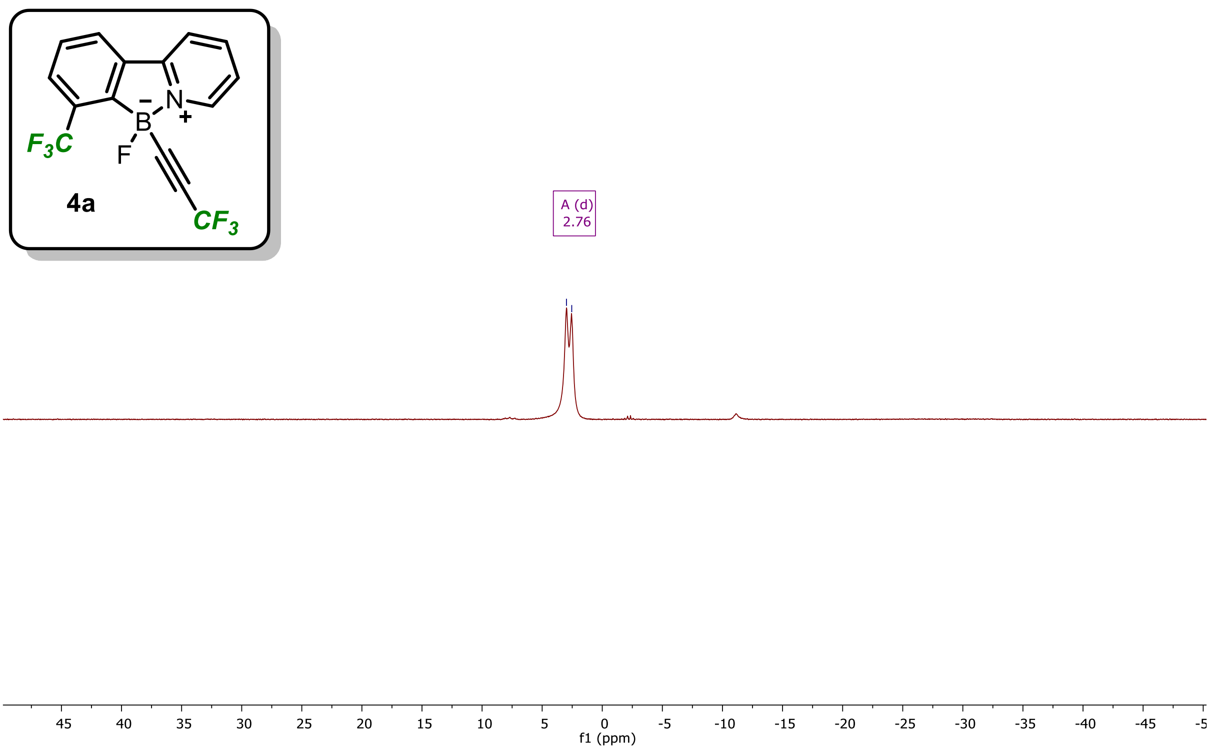

^{1}H NMR (400 MHz, acetone-d^6) spectrum of 5

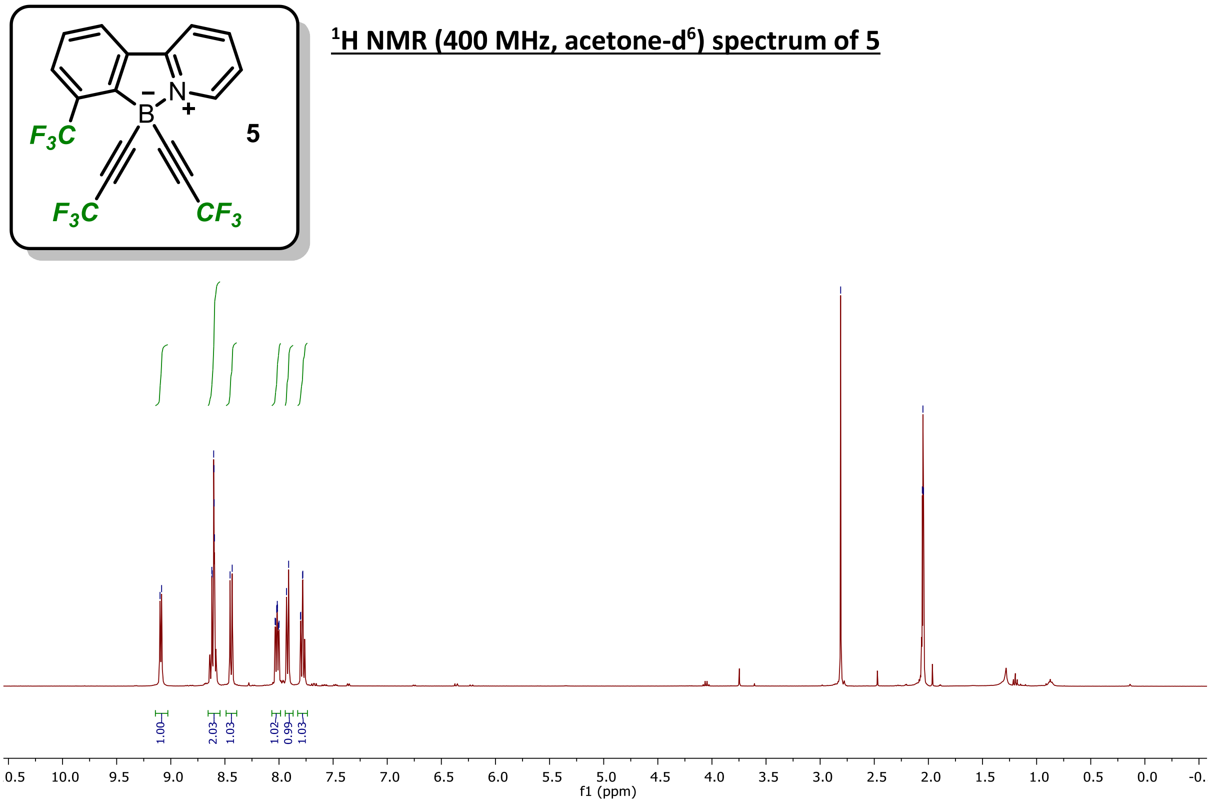

5

$^{13}C\{^{1}H\}$ NMR (101 MHz, acetone-d6) spectrum of 5

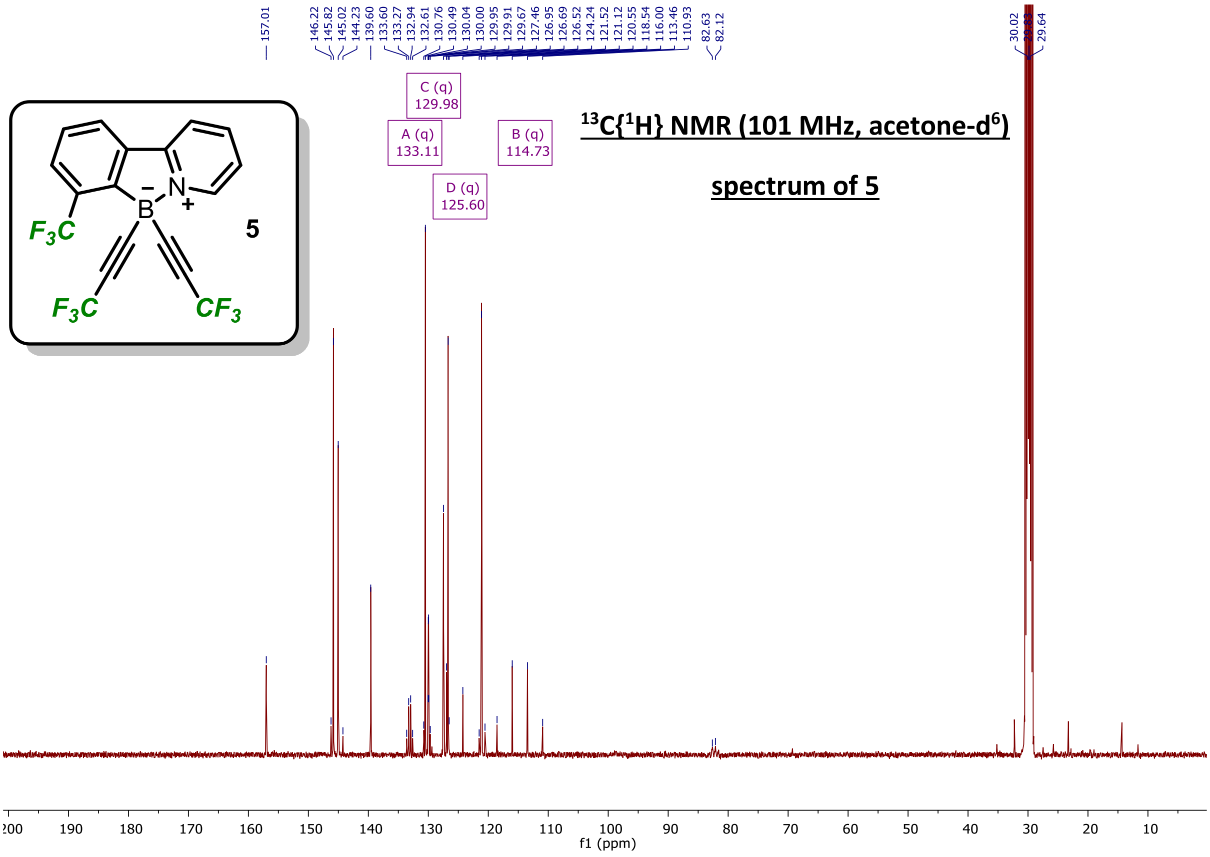

^{19}F NMR (376 MHz, acetone-d6) spectrum of 5

5

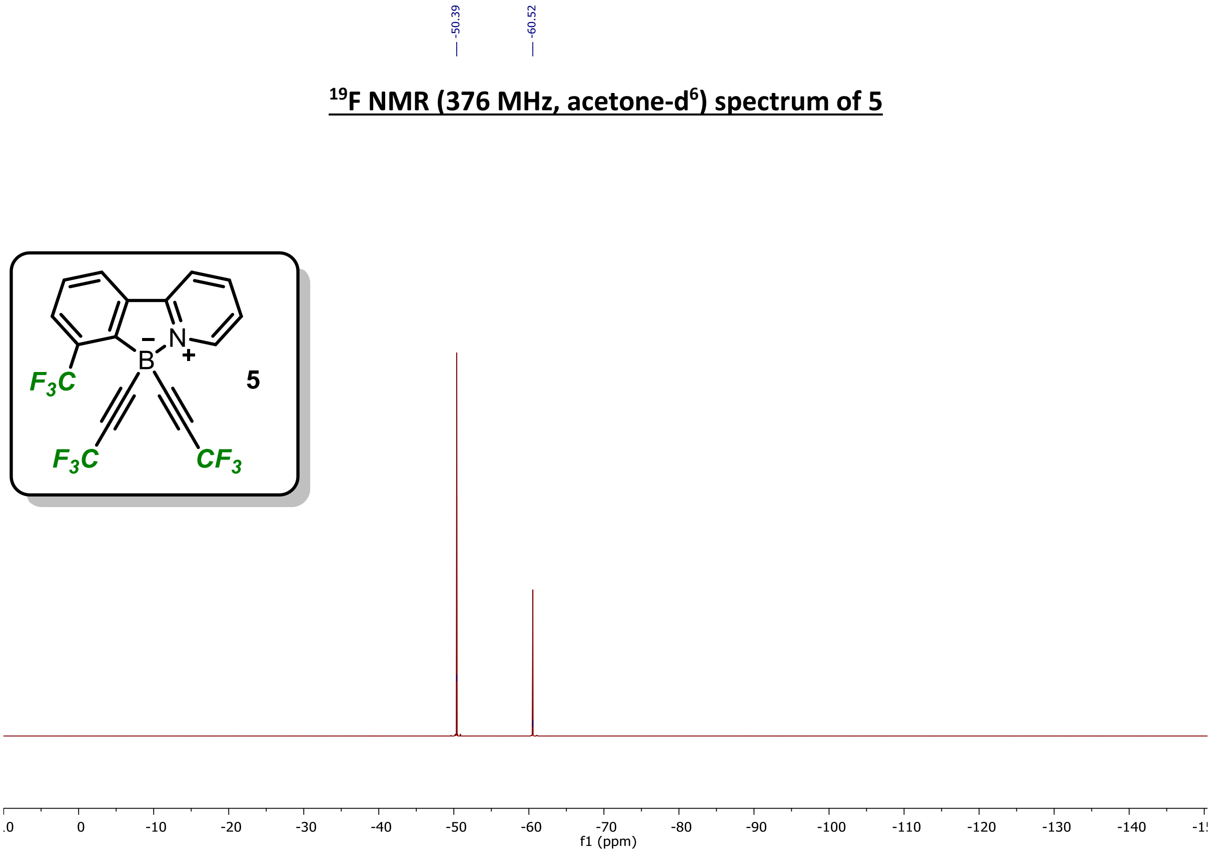

^{11}B NMR (128 MHz, acetone-d^{6}) spectrum of 5

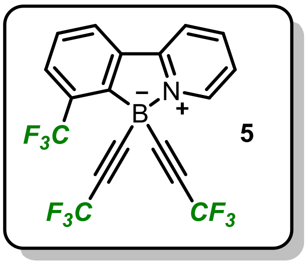

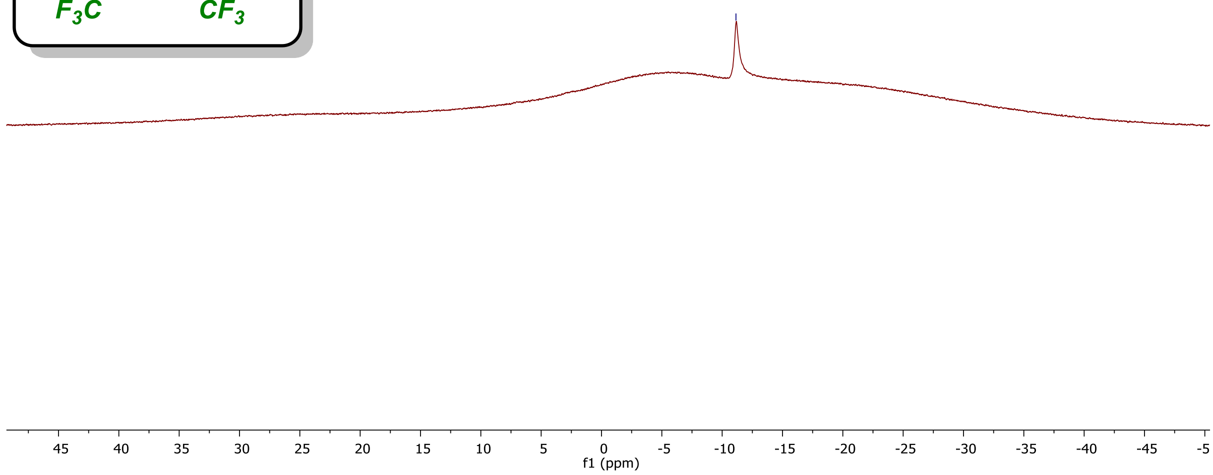

^{1}H NMR (400 MHz, acetone-d^{6}) spectrum of 4b

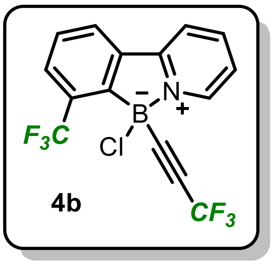

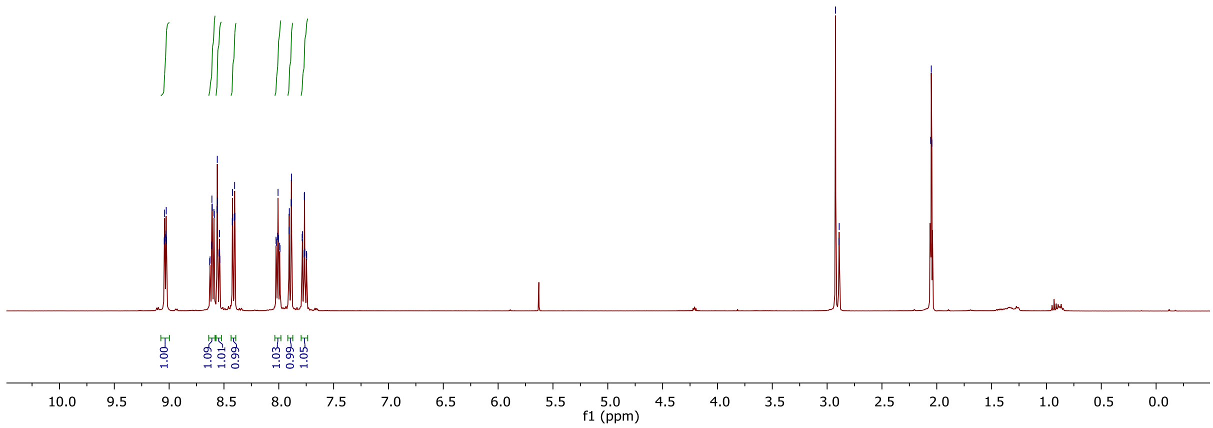

$^{13}C\{^{1}H\}$ NMR (101 MHz, acetone-d^{6}) spectrum of 4b

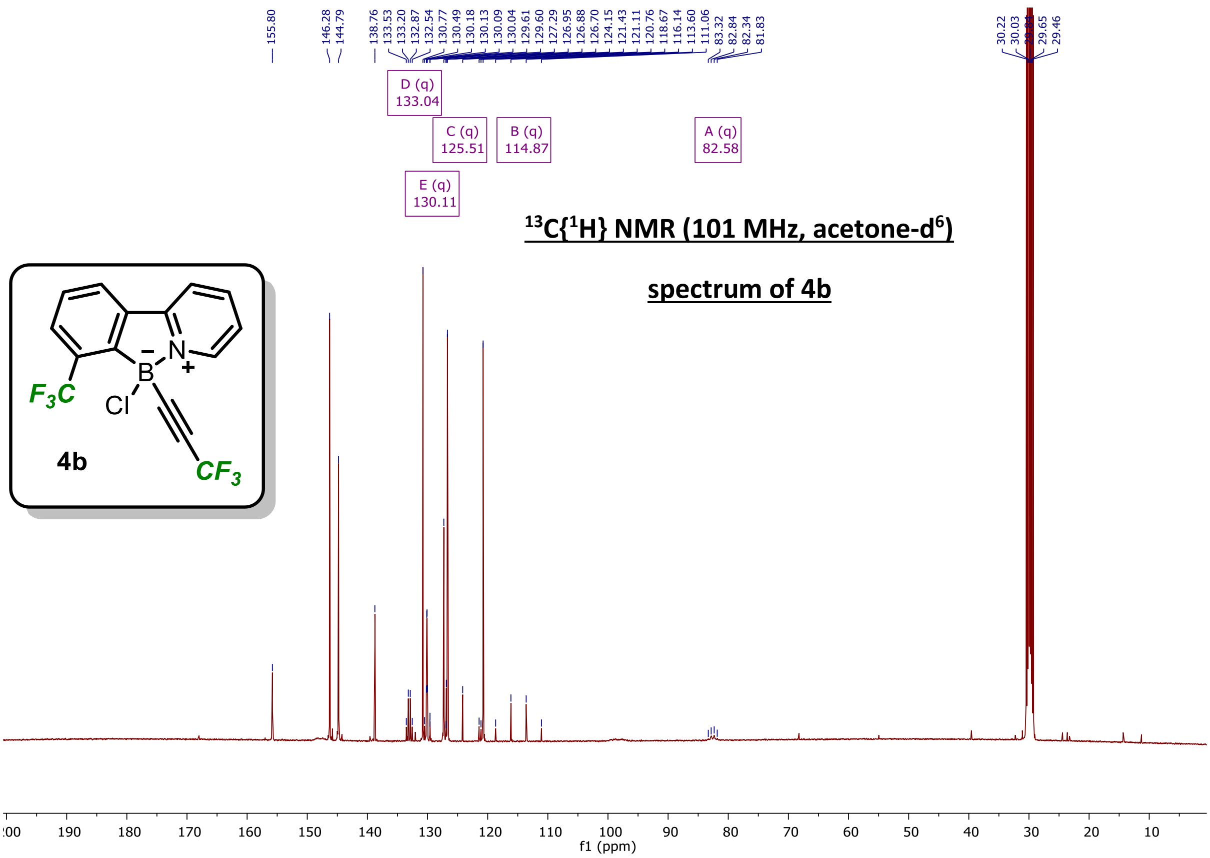

^{19}F NMR (376 MHz, acetone-d^{6}) spectrum of 4b

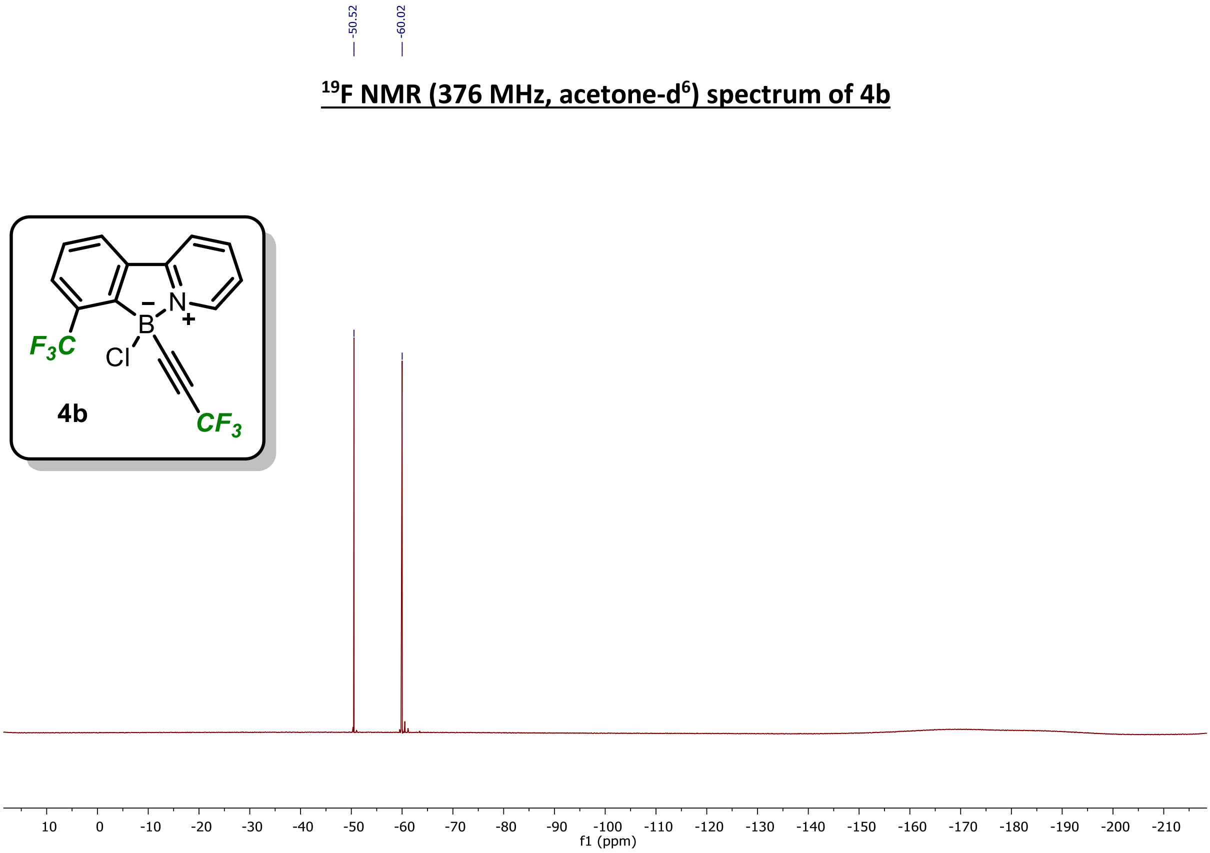

^{11}B NMR (128 MHz, acetone-d[6]) spectrum of 4b

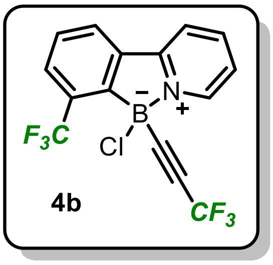

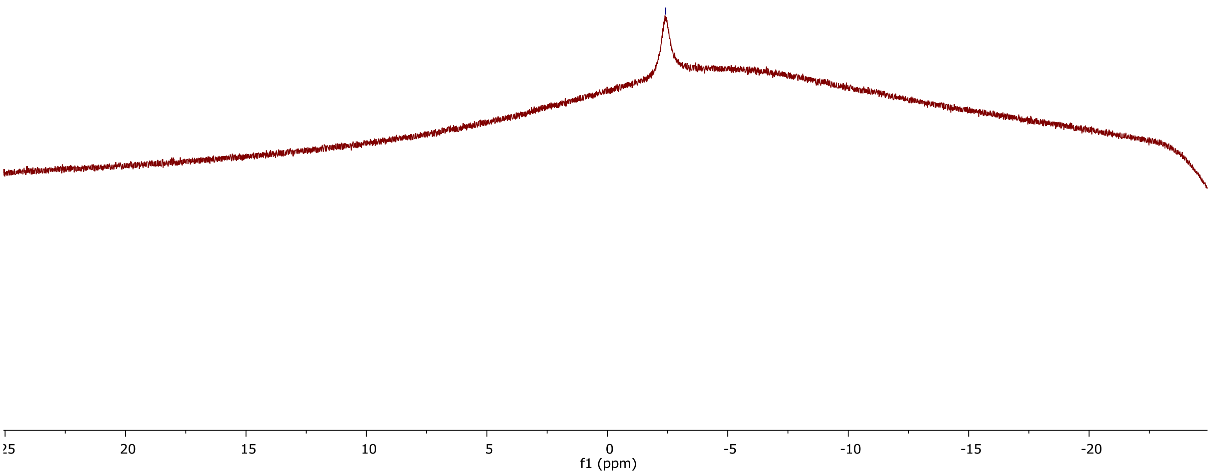

^{1}H NMR (400 MHz, acetone-d[6]) spectrum of 3b

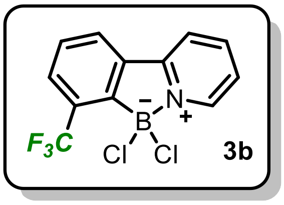

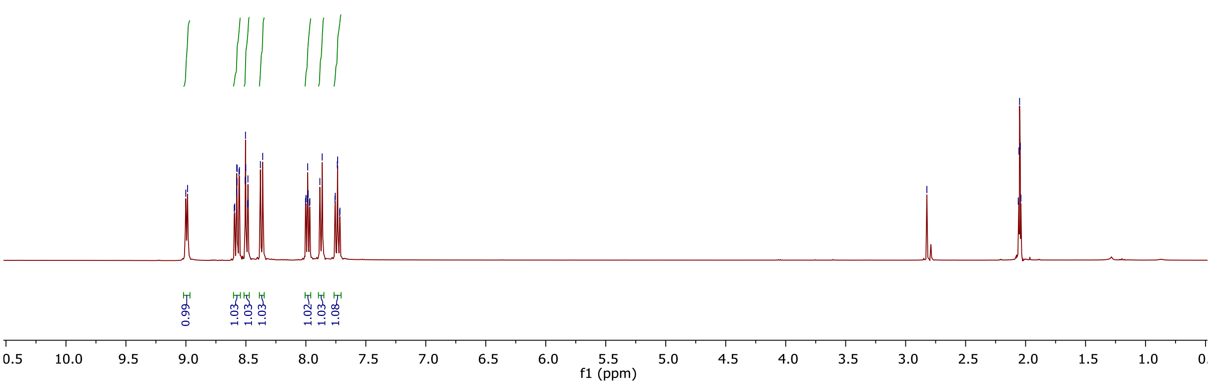

$^{13}C\{^1H\}$ NMR (101 MHz, acetone-d^6) spectrum of 3b

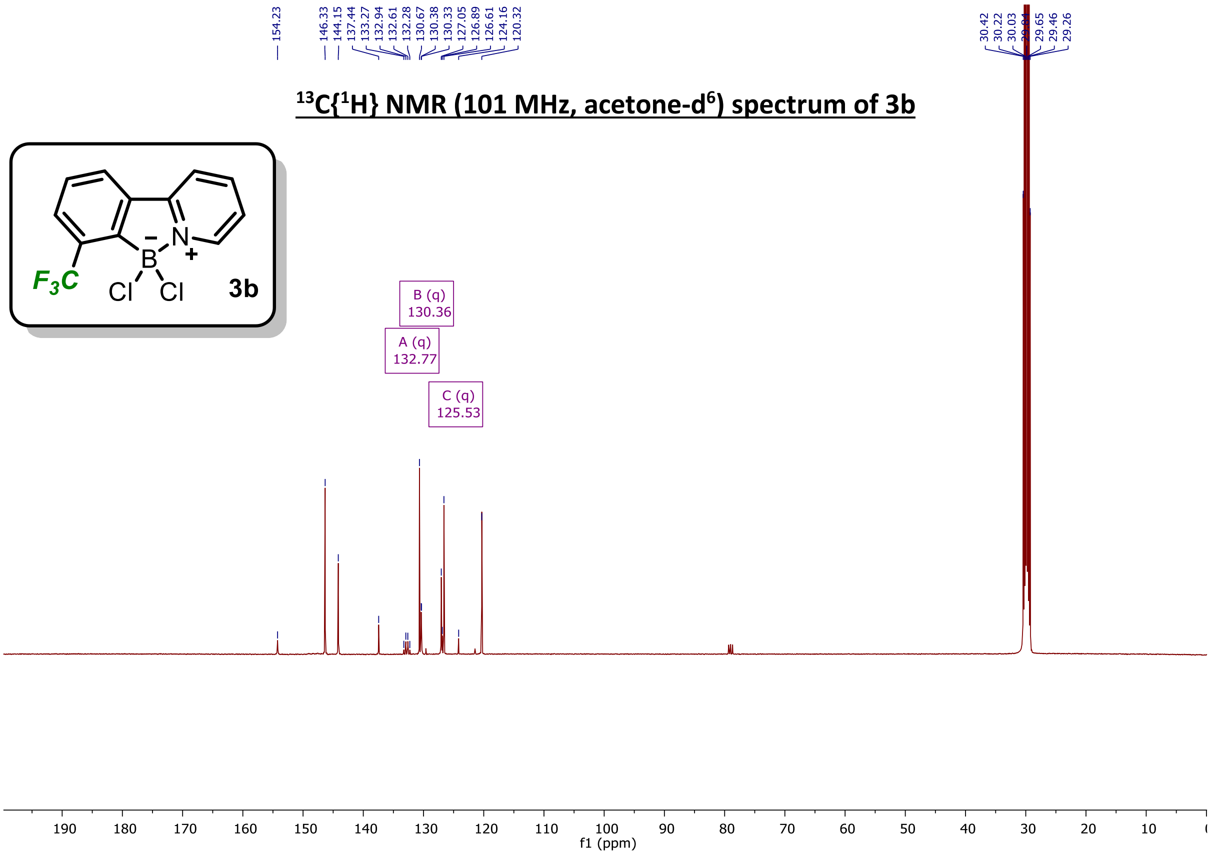

^{19}F NMR (376 MHz, acetone-d^6) spectrum of 3b

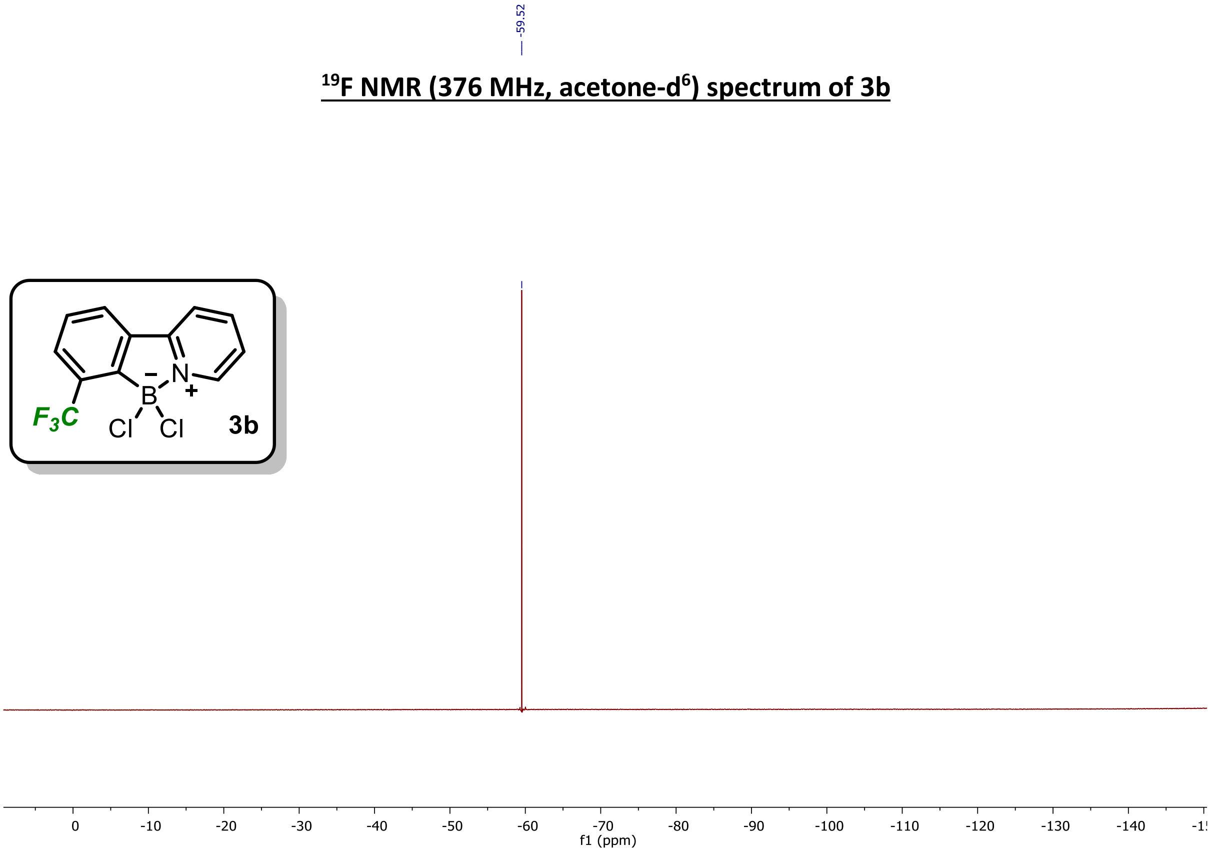

^{11}B NMR (128 MHz, acetone-d^6) spectrum of 3b

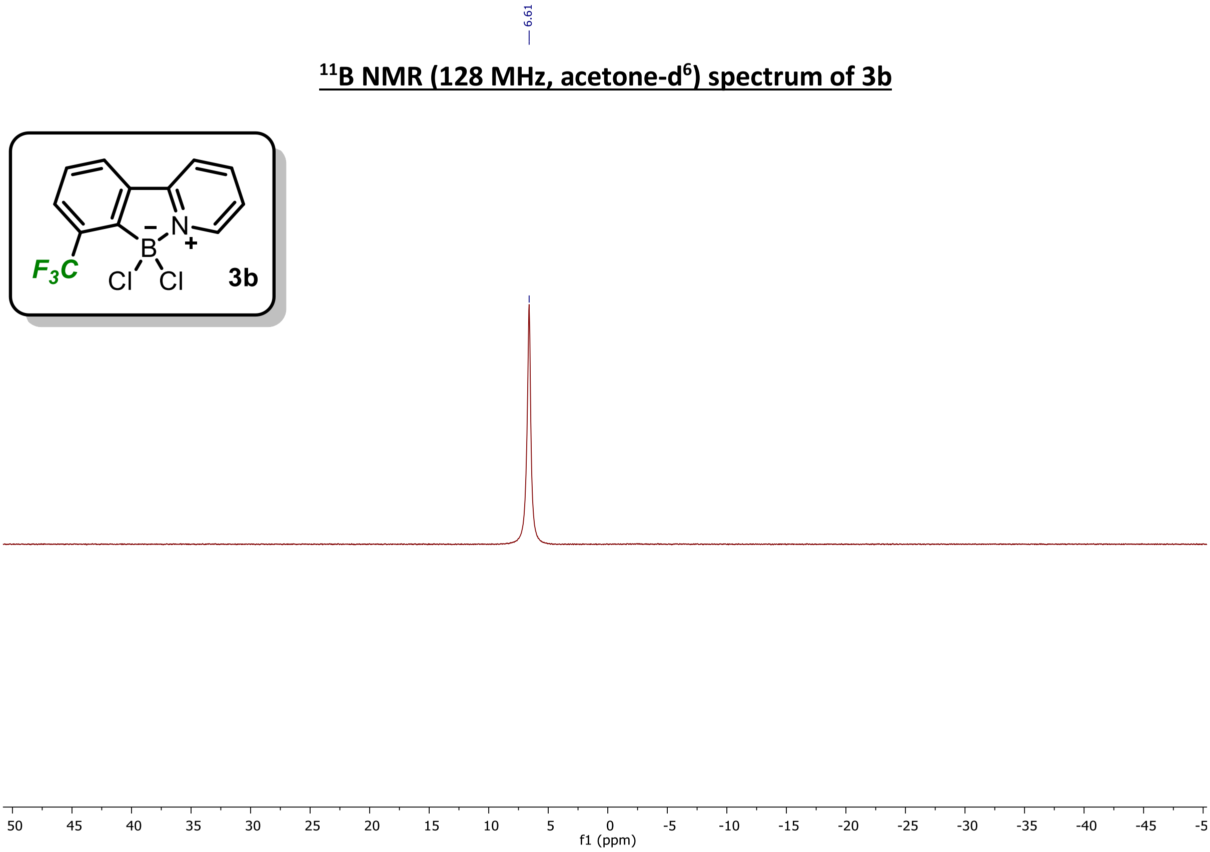

^{1}H NMR (400 MHz, acetone-d^6) spectrum of 6

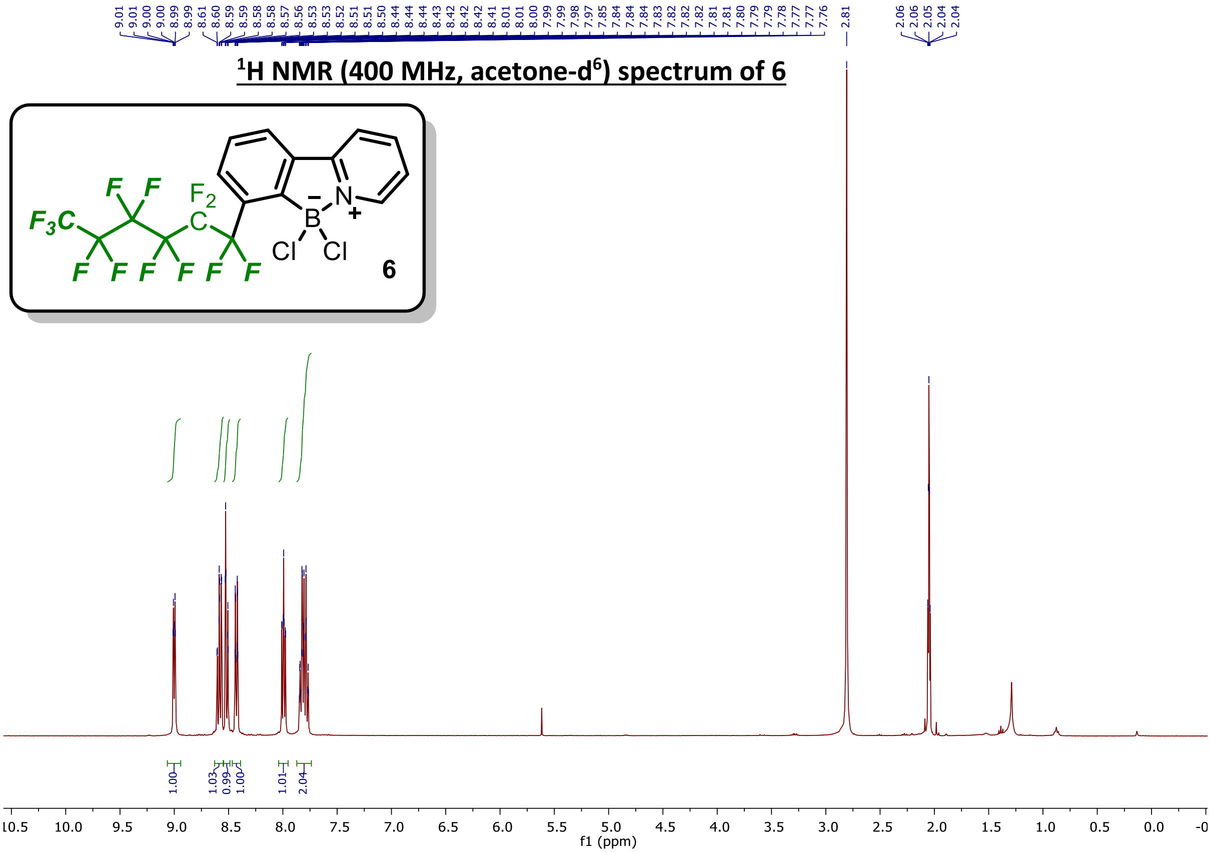

$^{13}C\{^{1}H\}$ NMR (101 MHz, acetone-d^{6})

spectrum of 6

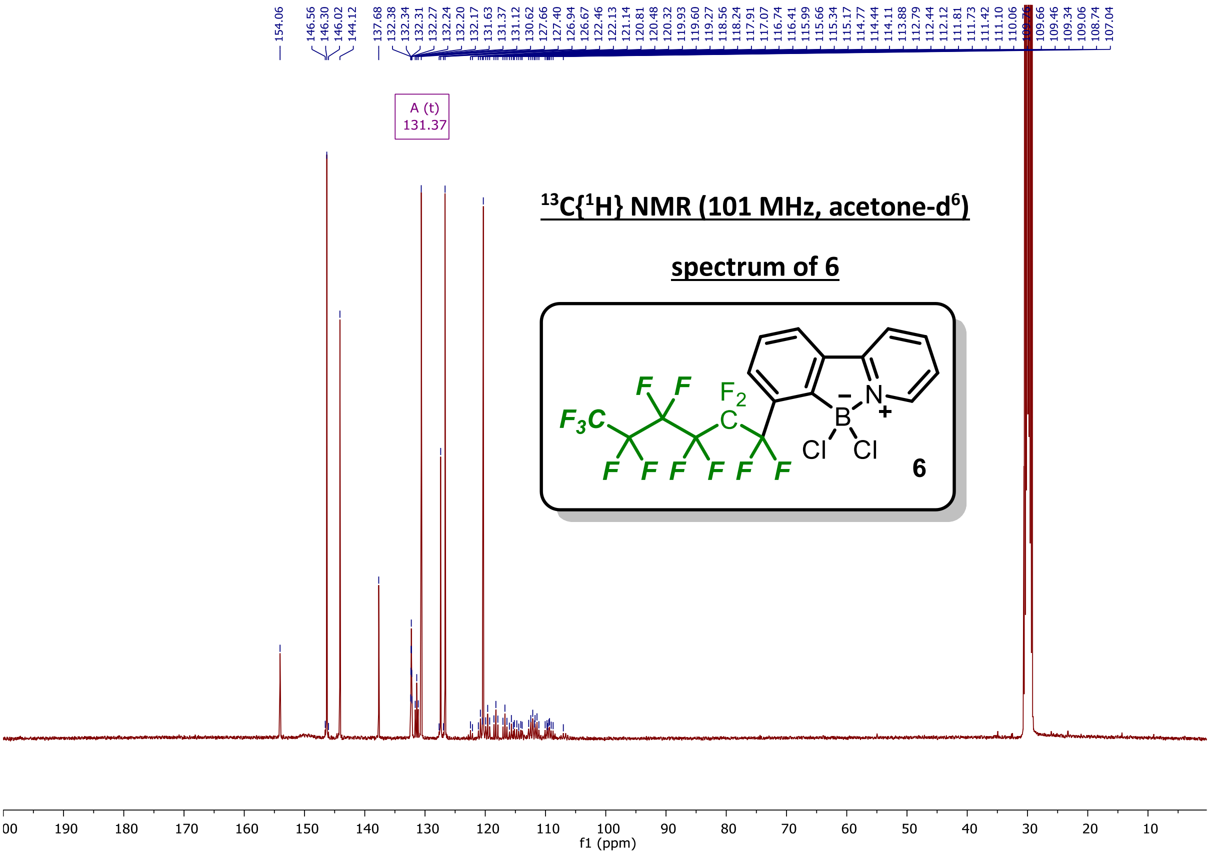

^{19}F NMR (376 MHz, acetone-d^{6}) spectrum of 6

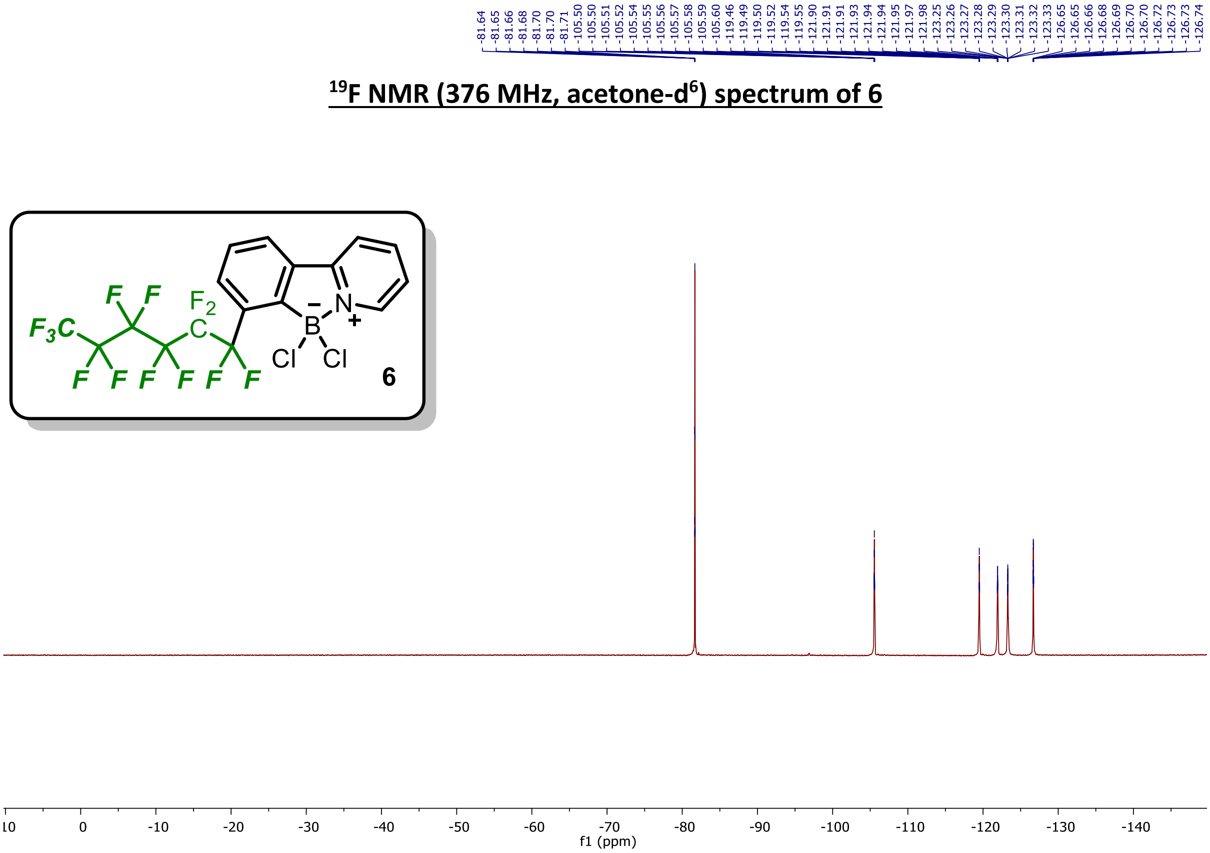

^{11}B NMR (128 MHz, acetone-d^6) spectrum of 6

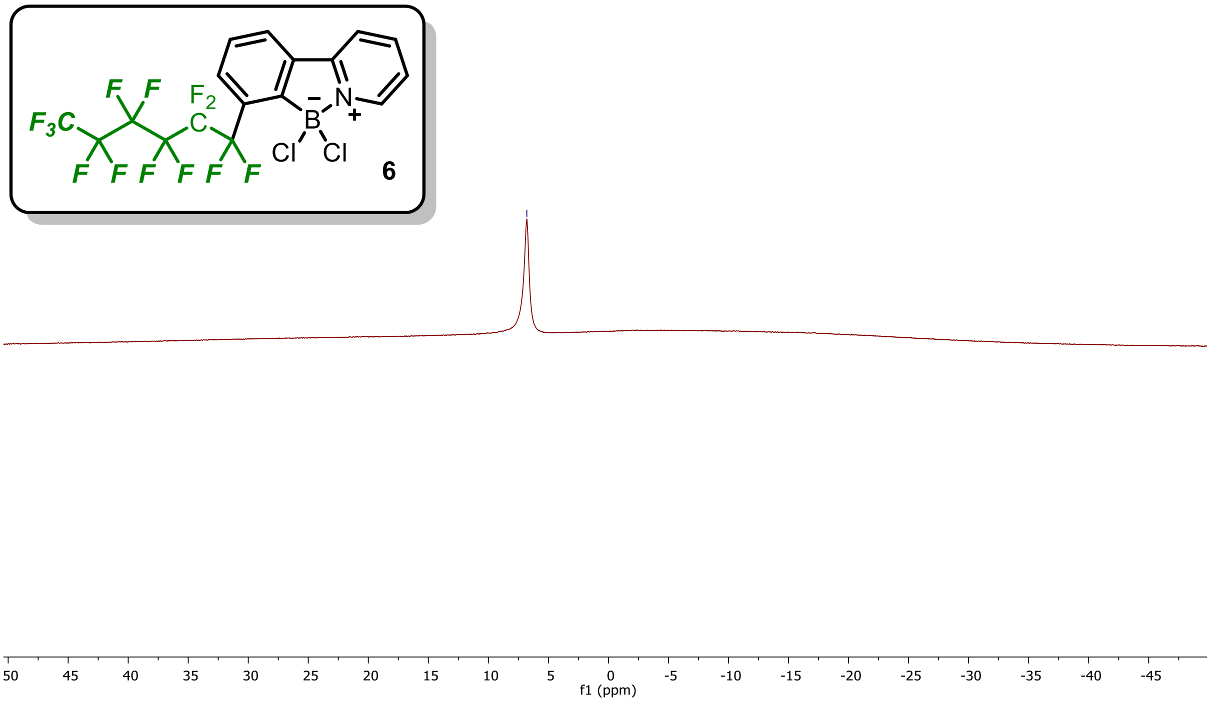

^{1}H NMR (400 MHz, acetone-d^6) spectrum of 7

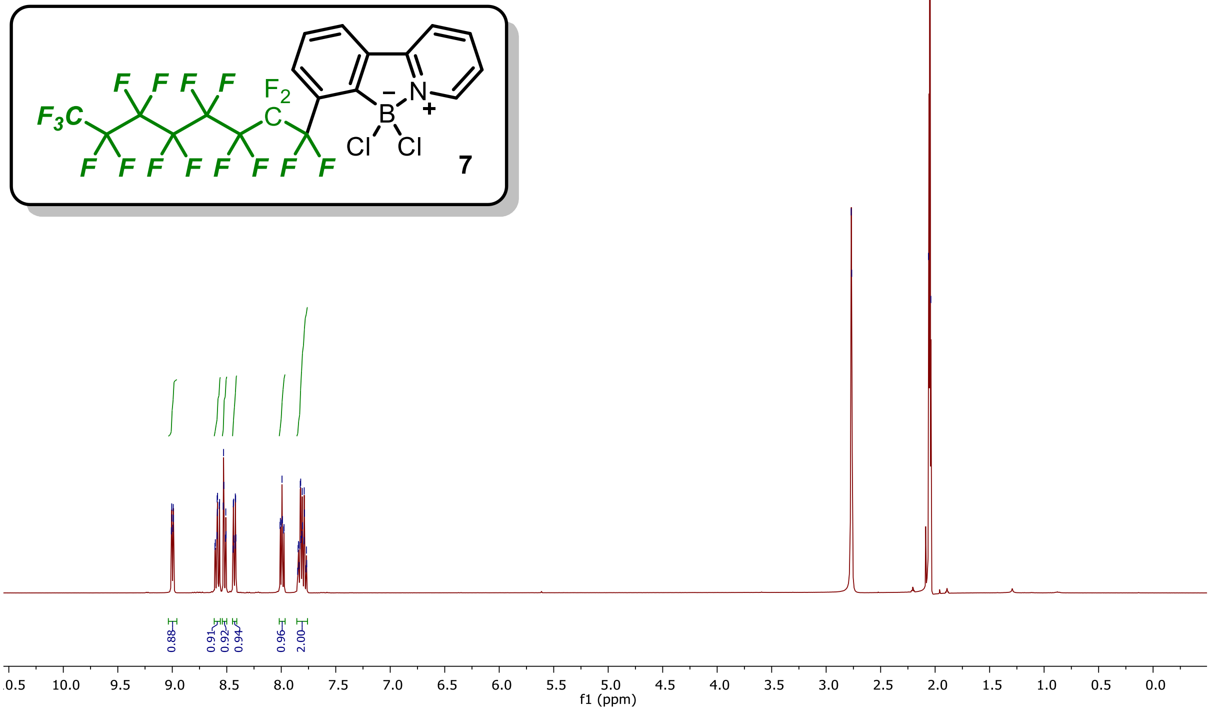

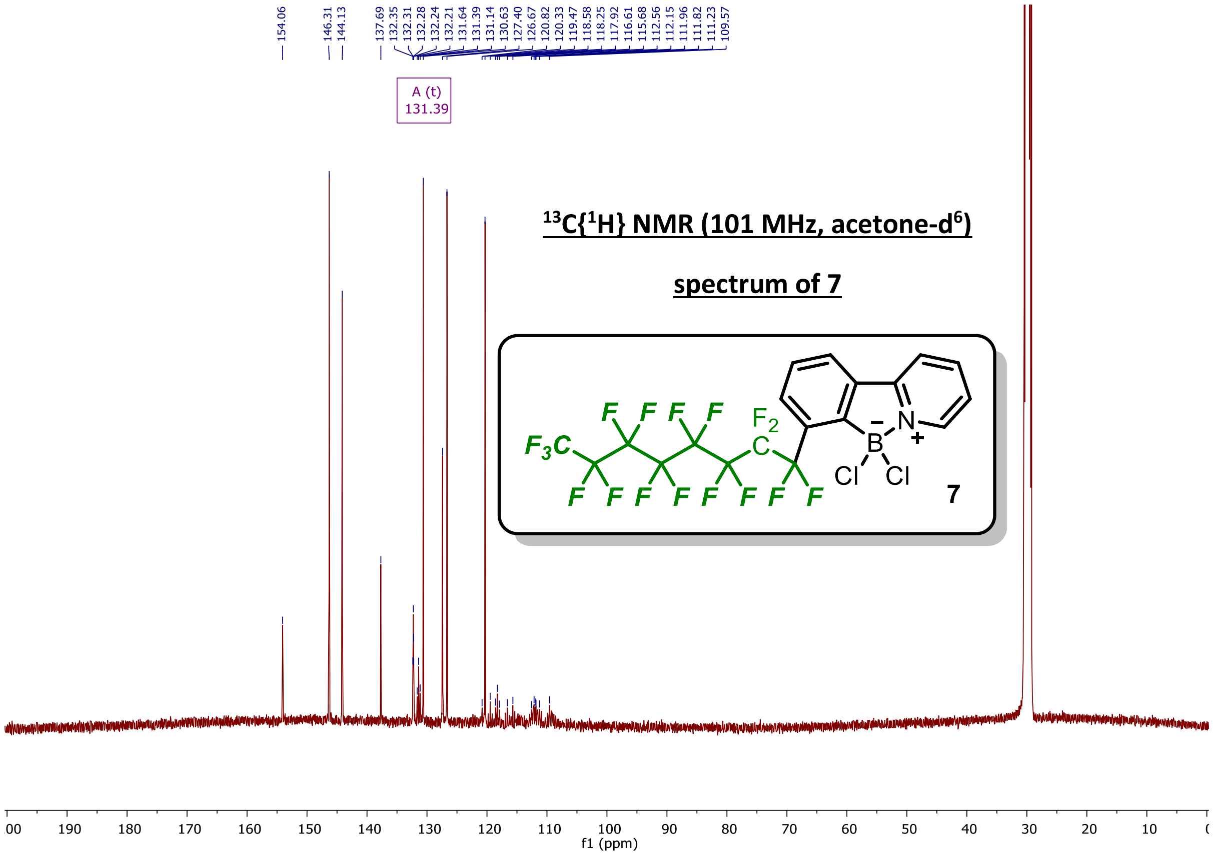

$^{13}C\{^{1}H\}$ NMR (101 MHz, acetone-d^{6}) spectrum of 7

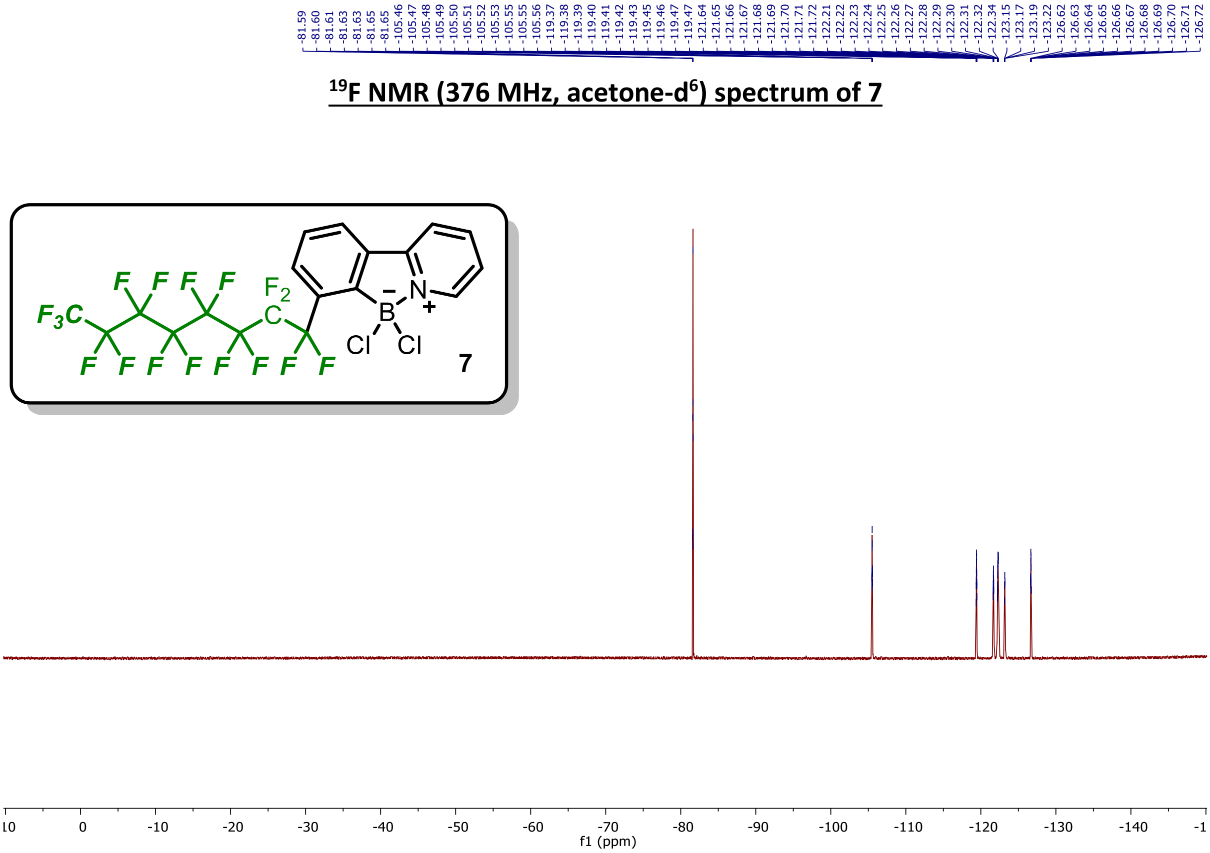

^{19}F NMR (376 MHz, acetone-d^{6}) spectrum of 7

^{11}B NMR (128 MHz, acetone-d^6) spectrum of 7

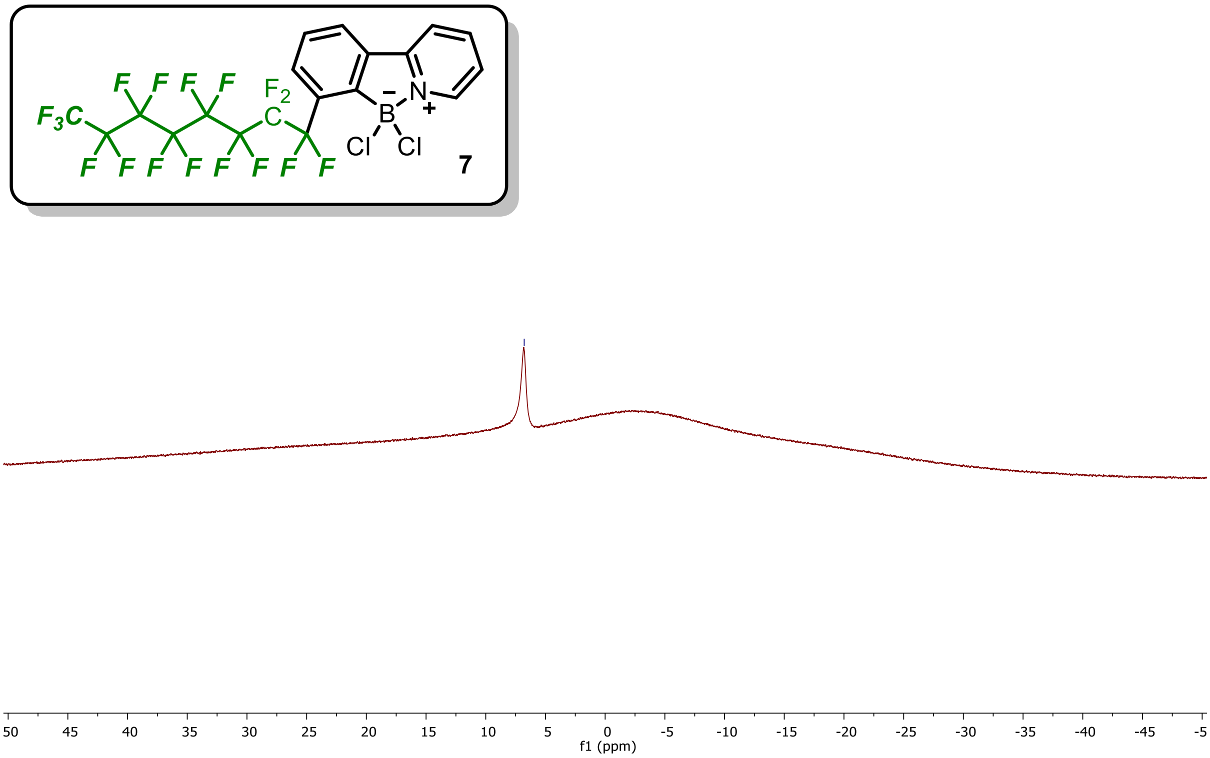

^{1}H NMR (400 MHz, acetone-d^6) spectrum of 3c

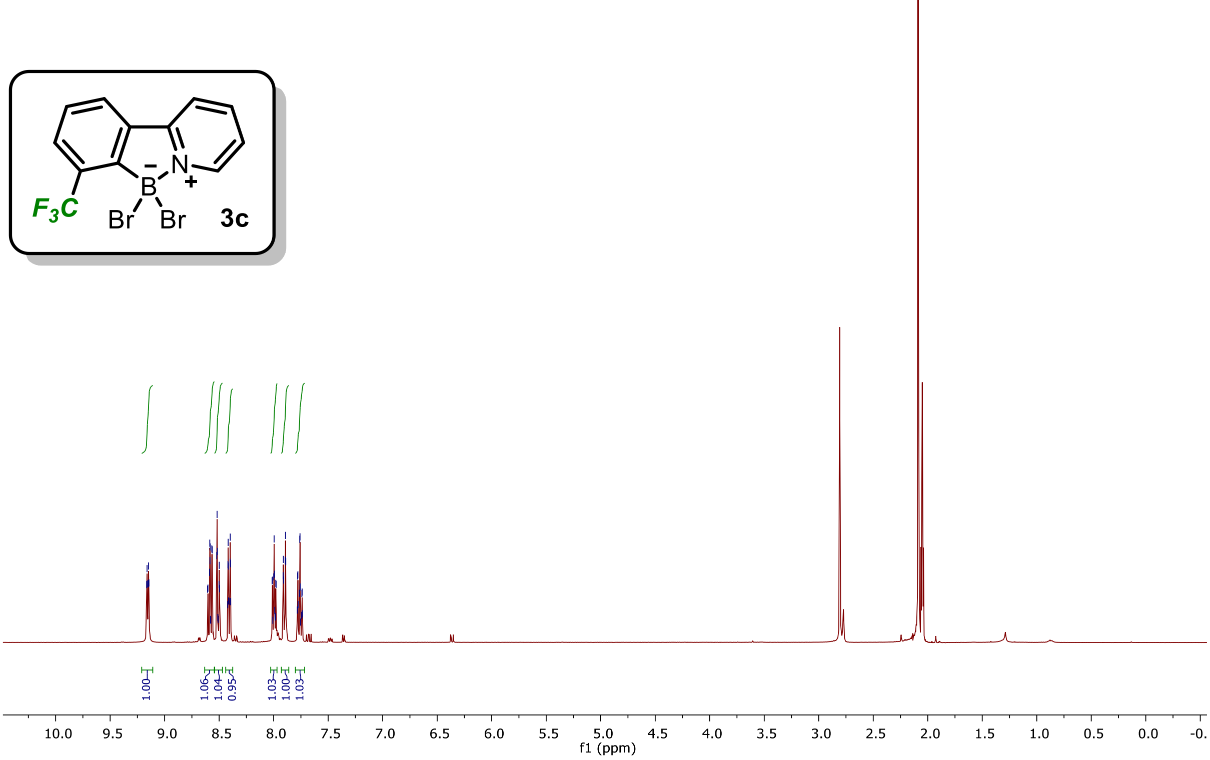

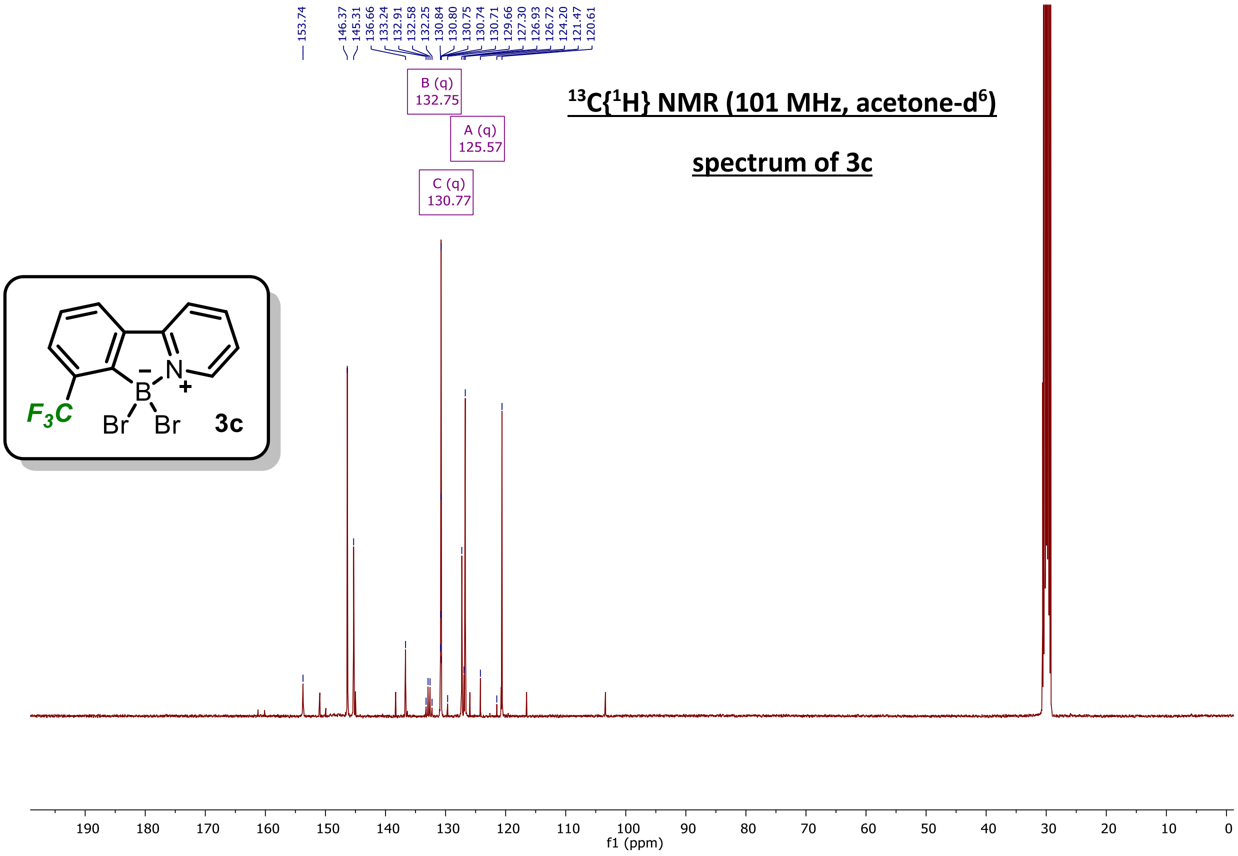
153.74
146.37
145.31
136.66
133.24
132.91
132.58
132.25
130.84
130.80
130.75
130.74
130.71
129.66
127.30
126.93
126.72
124.20
121.47
120.61
B (q)
132.75
A (q)
125.57
C (q)
130.77
$^{13}C\{^{1}H\}$ NMR (101 MHz, acetone-d6)
spectrum of 3c
F3C
B
N
Br
Br
3c
f1 (ppm)

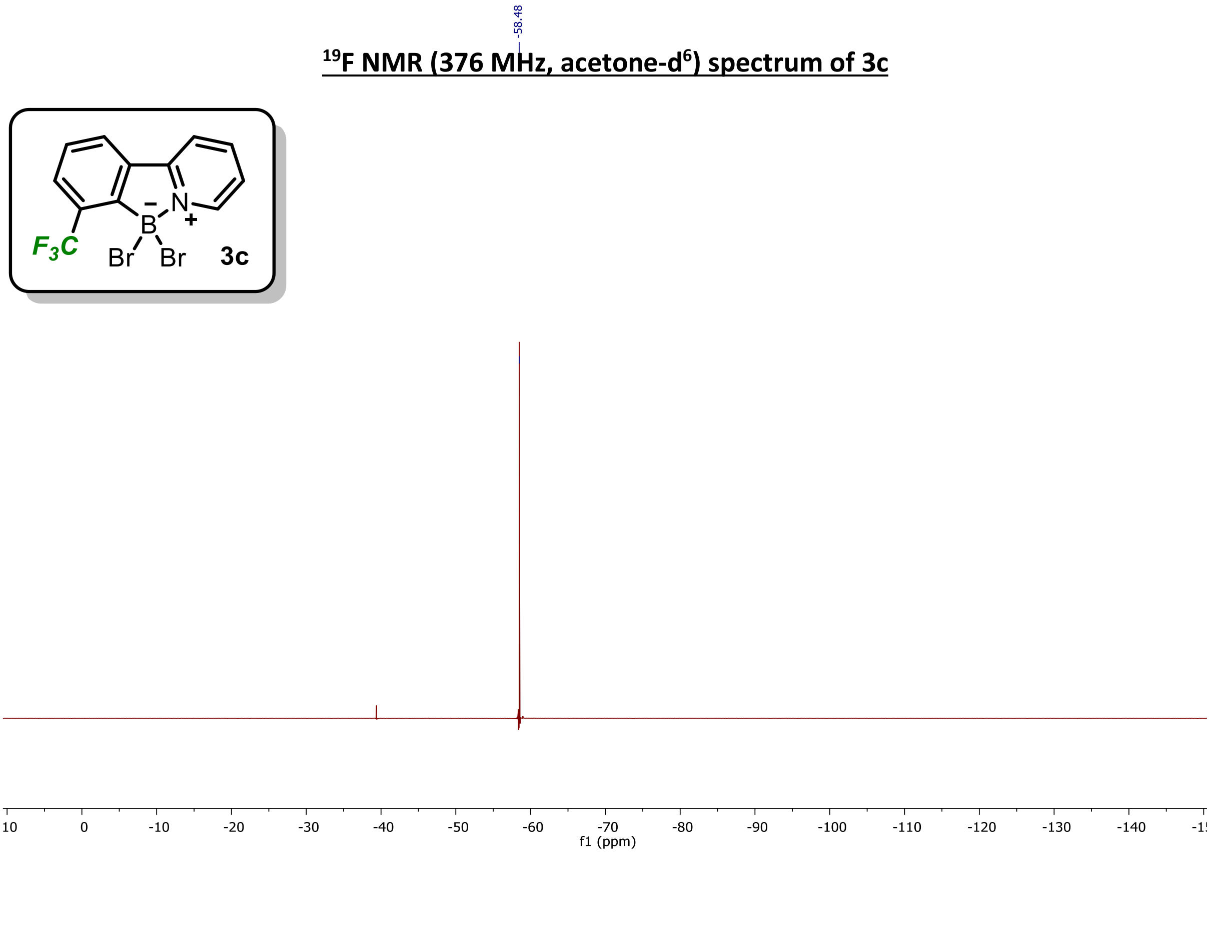
-58.48
^{19}F NMR (376 MHz, acetone-d6) spectrum of 3c
F3C
B
N
Br
Br
3c
f1 (ppm)

^{11}B NMR (128 MHz, acetone-d^6) spectrum of 3c

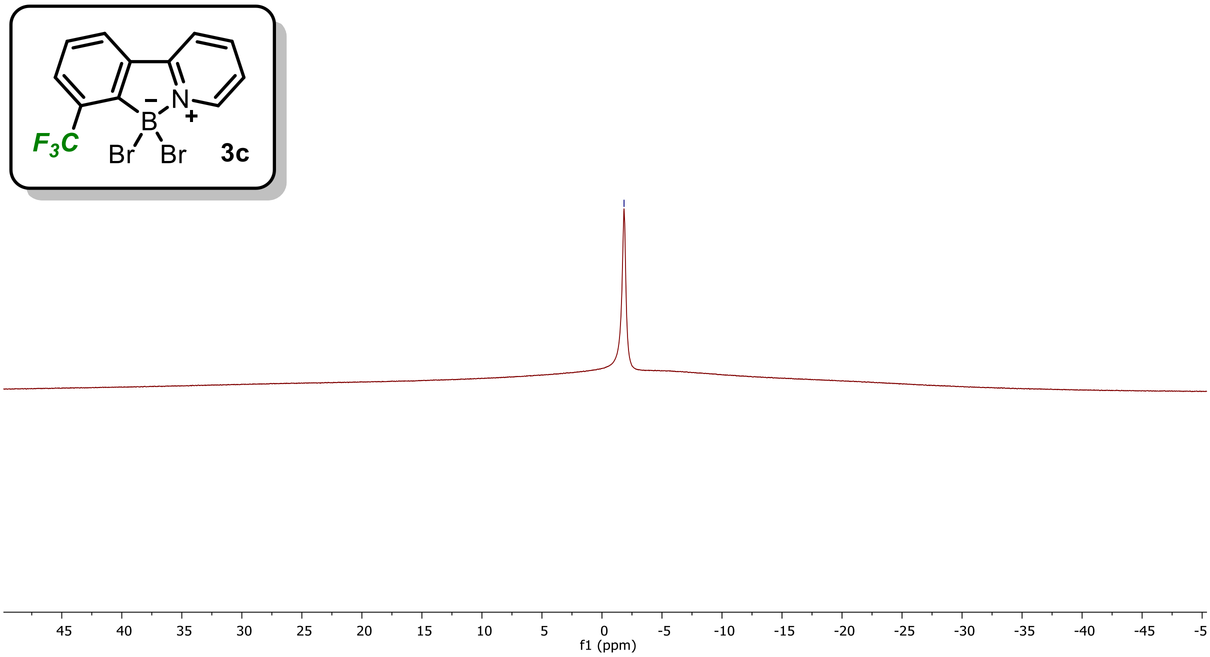

^{1}H NMR (400 MHz, acetone-d^6) spectrum of 8

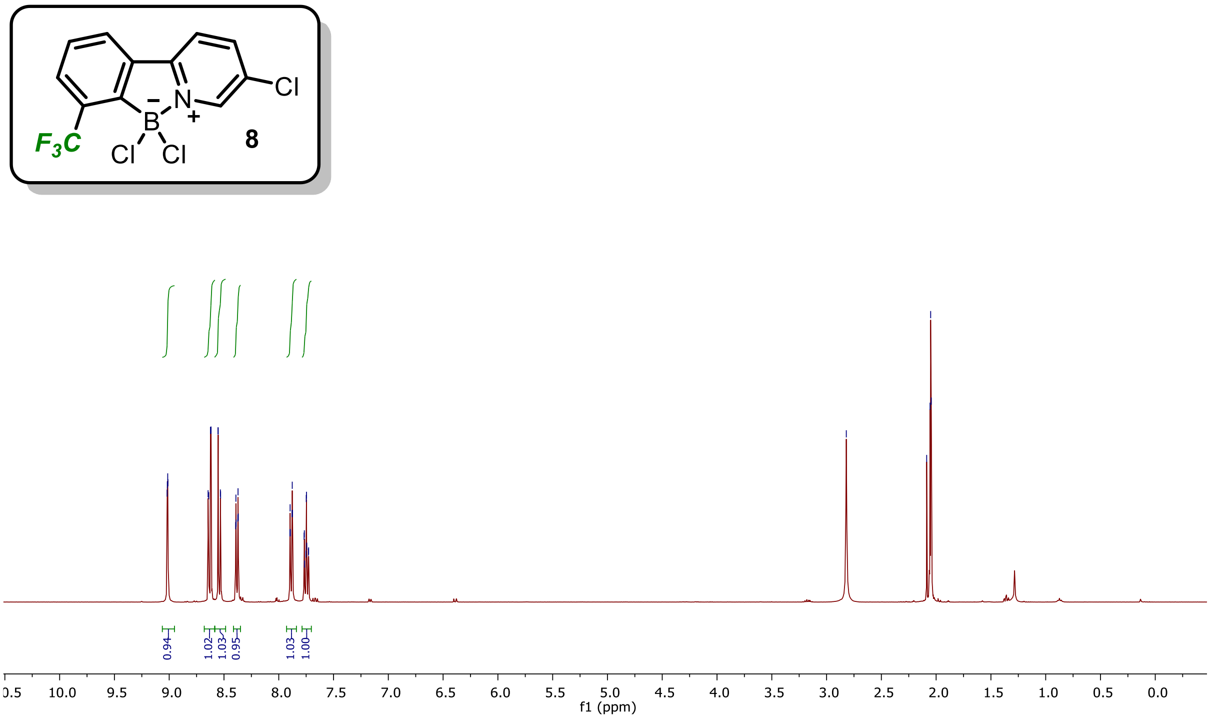

$^{13}C\{^1H\}$ NMR (101 MHz, acetone-d^6) spectrum of 8

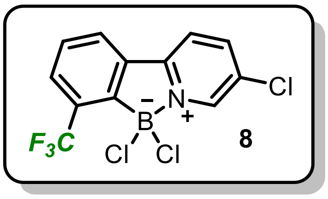

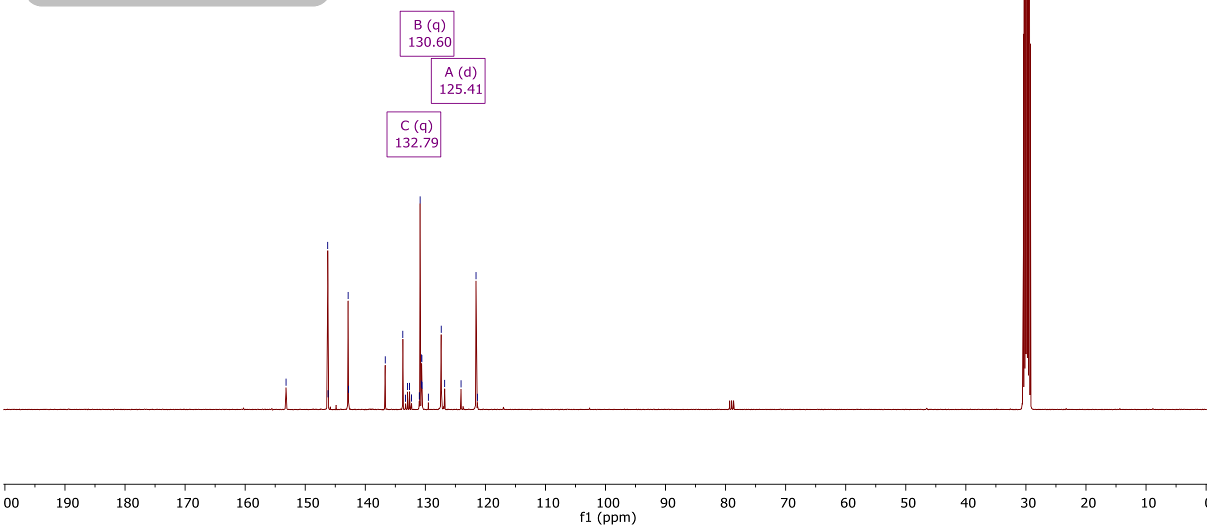

^{19}F NMR (376 MHz, acetone-d^6) spectrum of 8

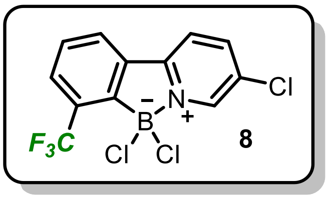

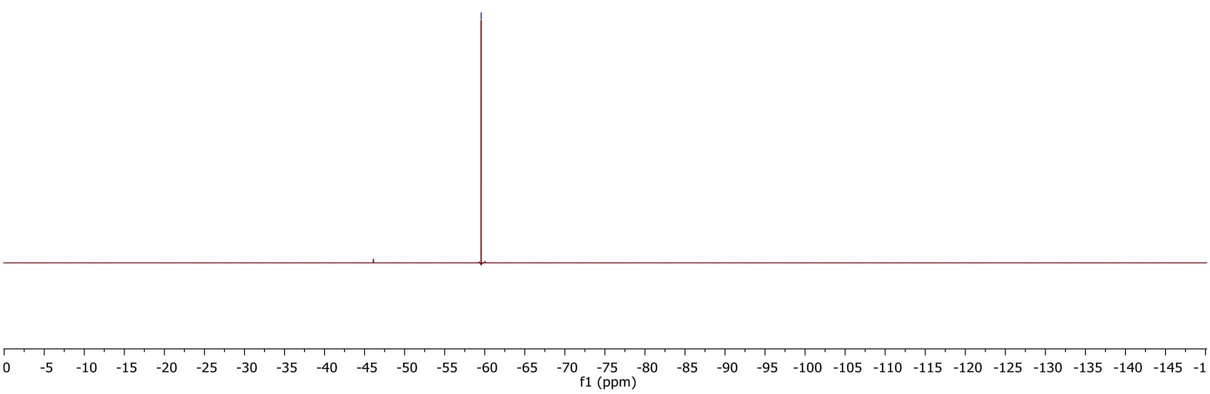

^{11}B NMR (128 MHz, acetone-d^6) spectrum of 8

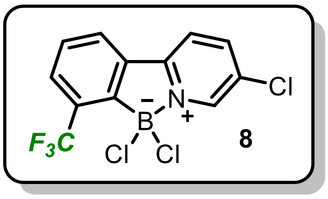

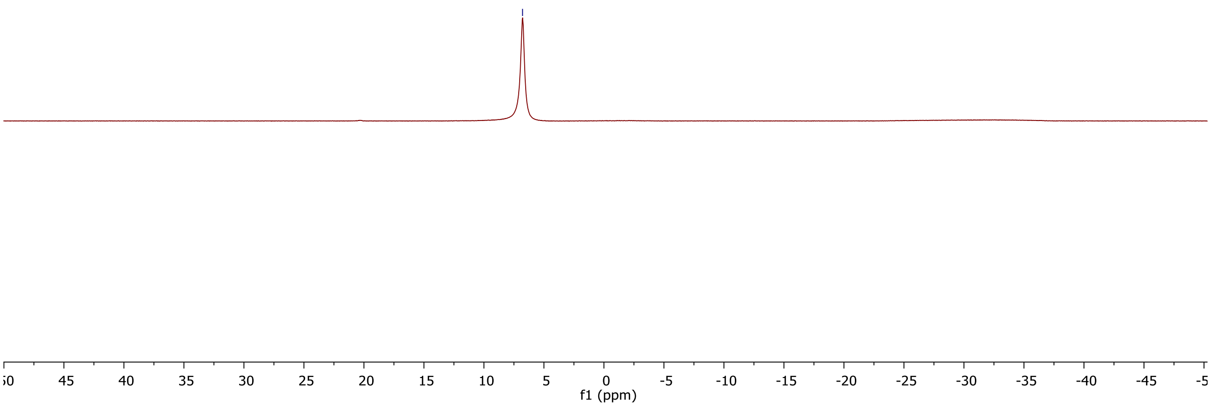

^{1}H NMR (400 MHz, acetone-d^6) spectrum of 9

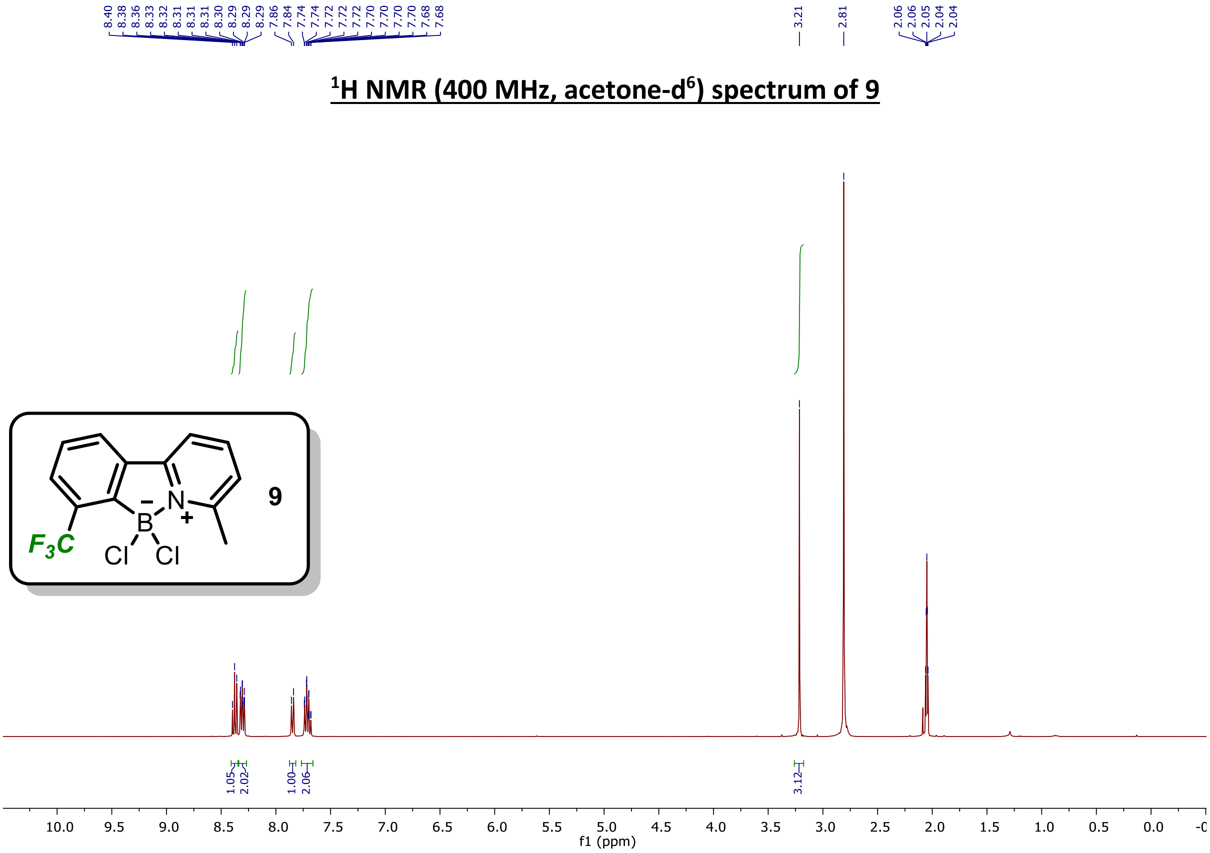

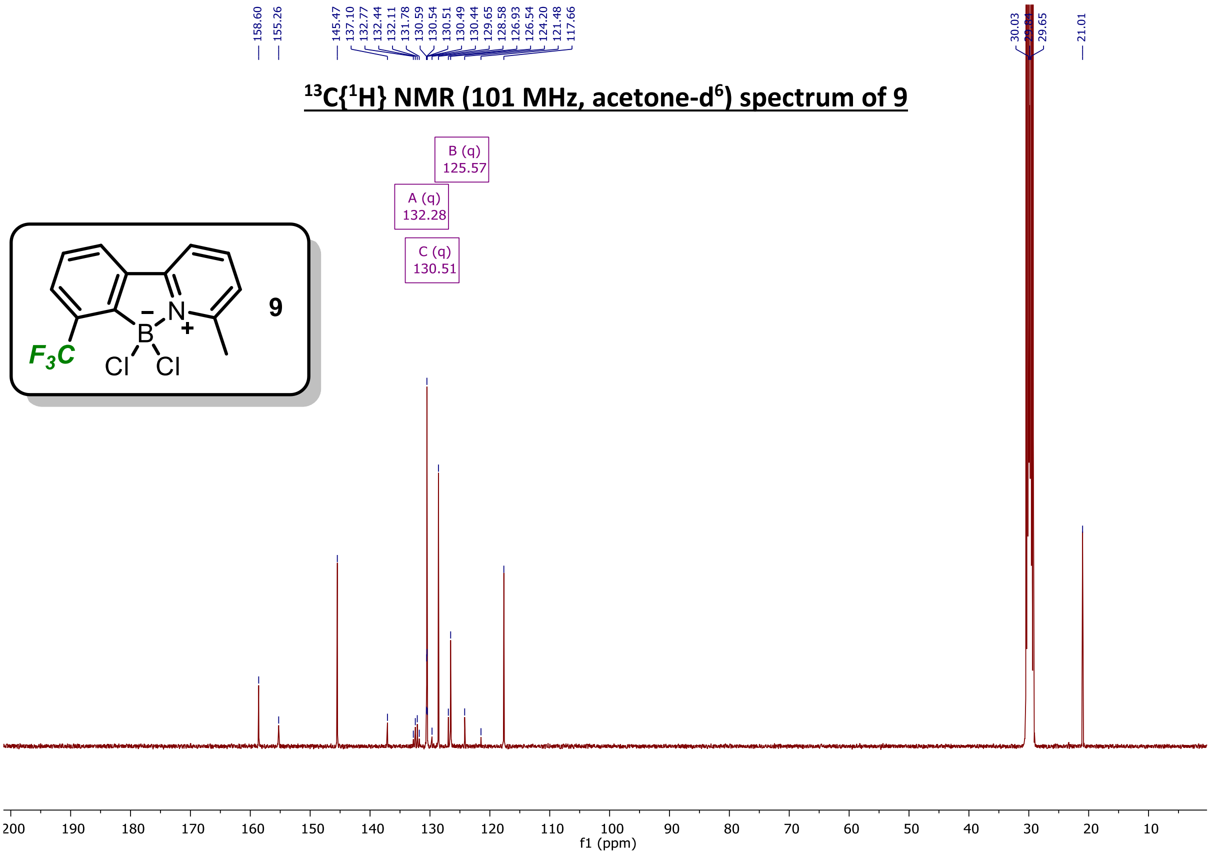

$^{13}C\{^{1}H\}$ NMR (101 MHz, acetone-d^{6}) spectrum of 9

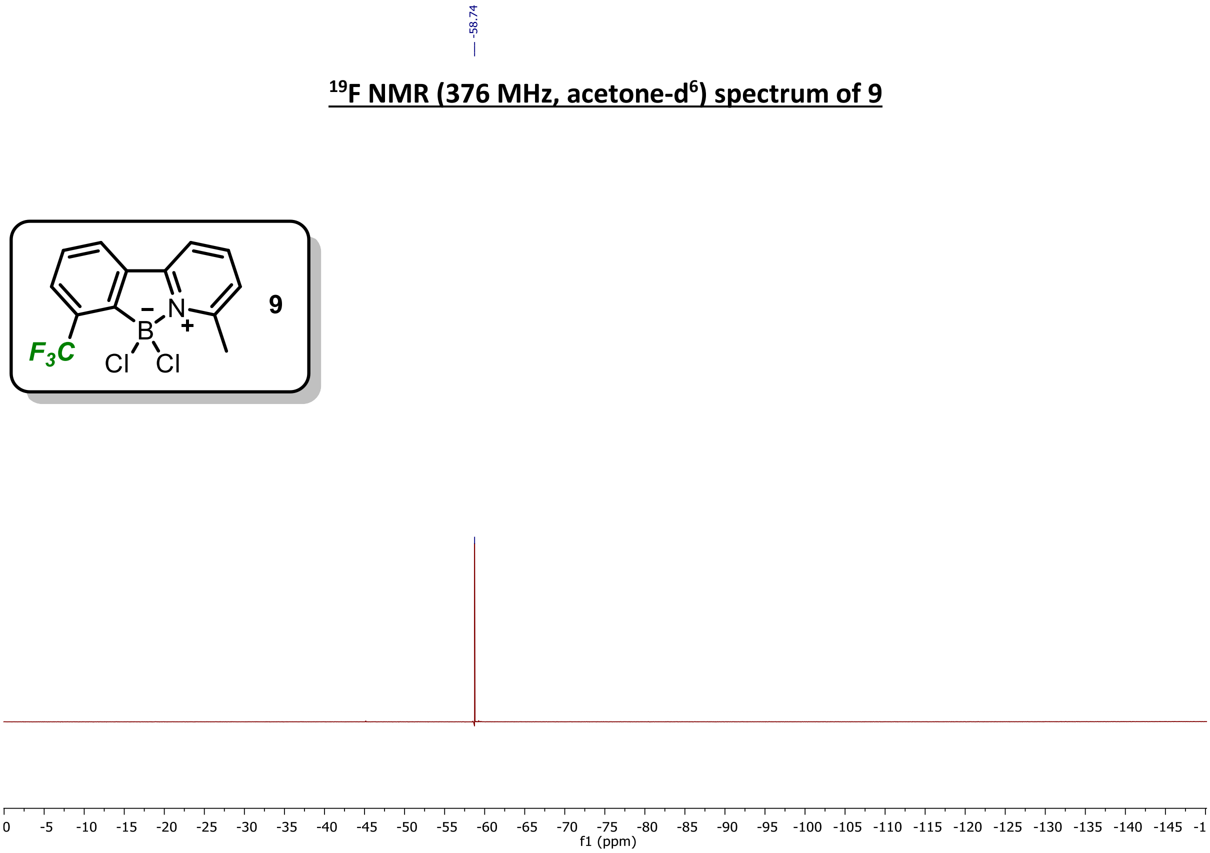

^{19}F NMR (376 MHz, acetone-d^{6}) spectrum of 9

^{11}B NMR (128 MHz, acetone-d^6) spectrum of 9

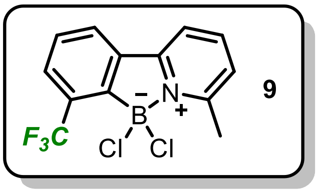

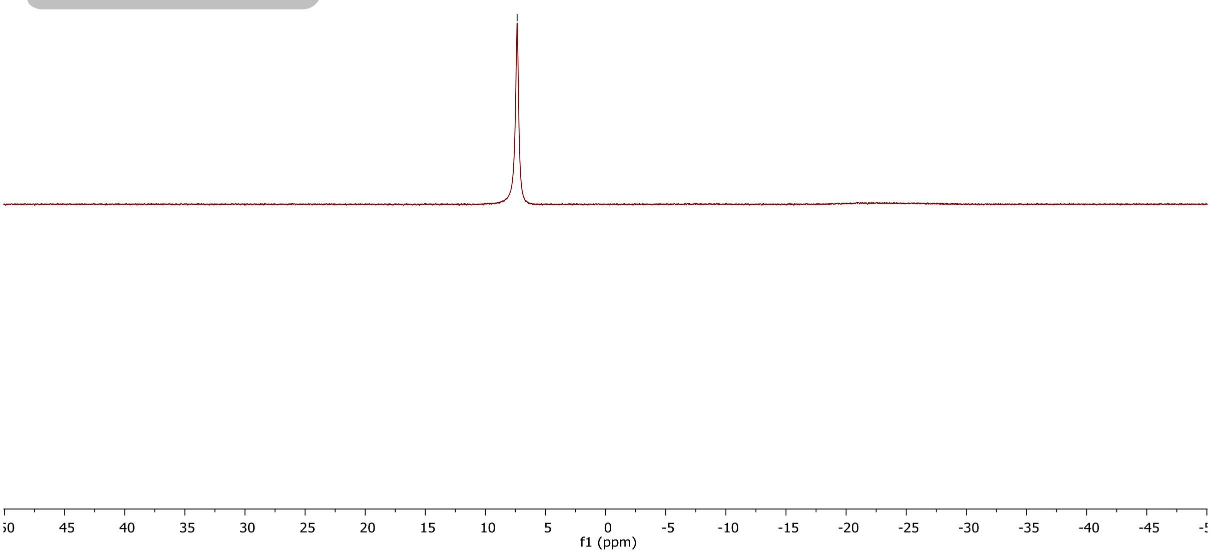

^{1}H NMR (400 MHz, DMSO-d^6) spectrum of 10

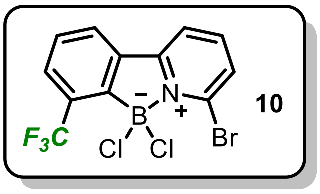

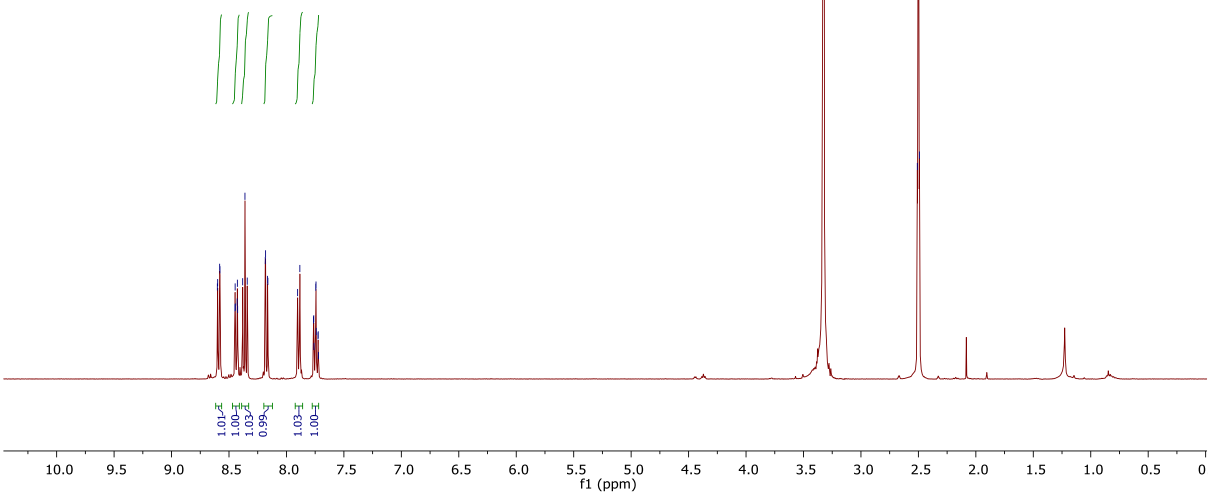

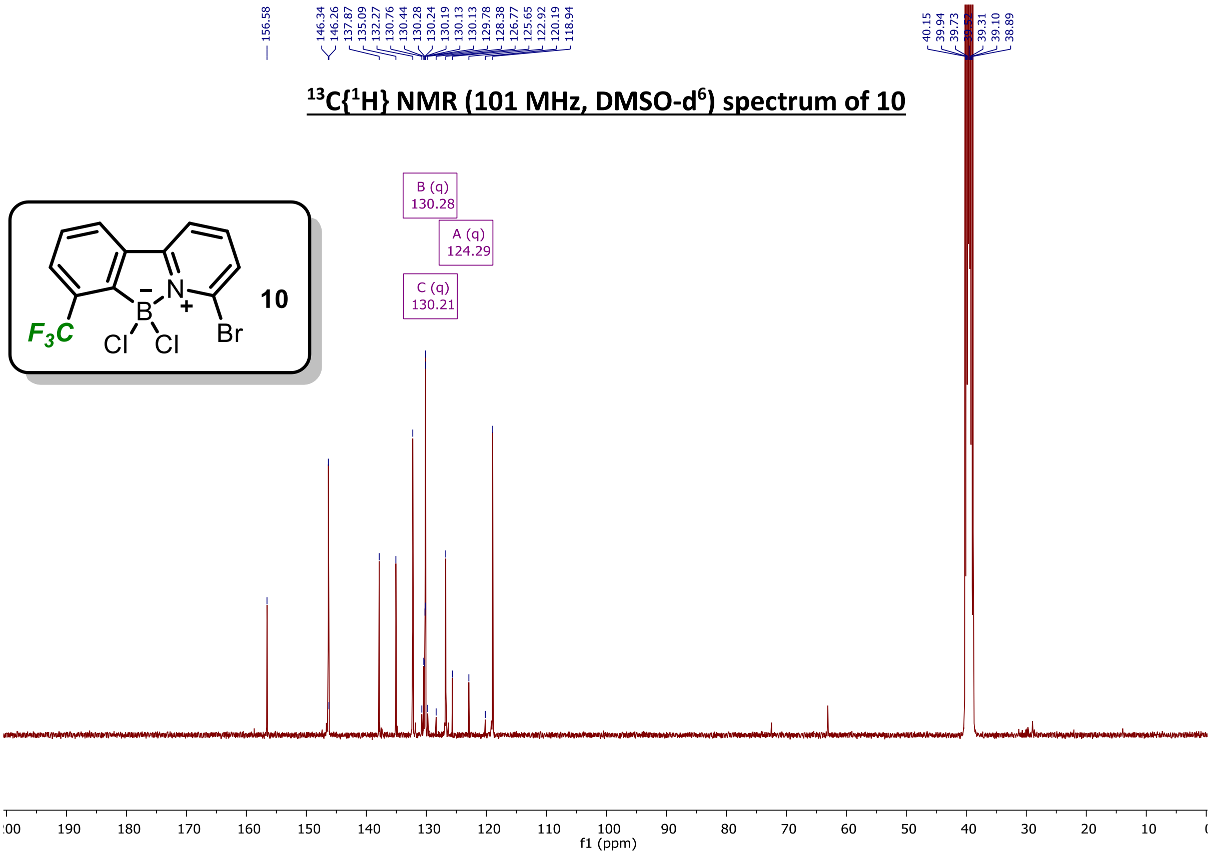

$^{13}C\{^{1}H\}$ NMR (101 MHz, DMSO-d^{6}) spectrum of 10

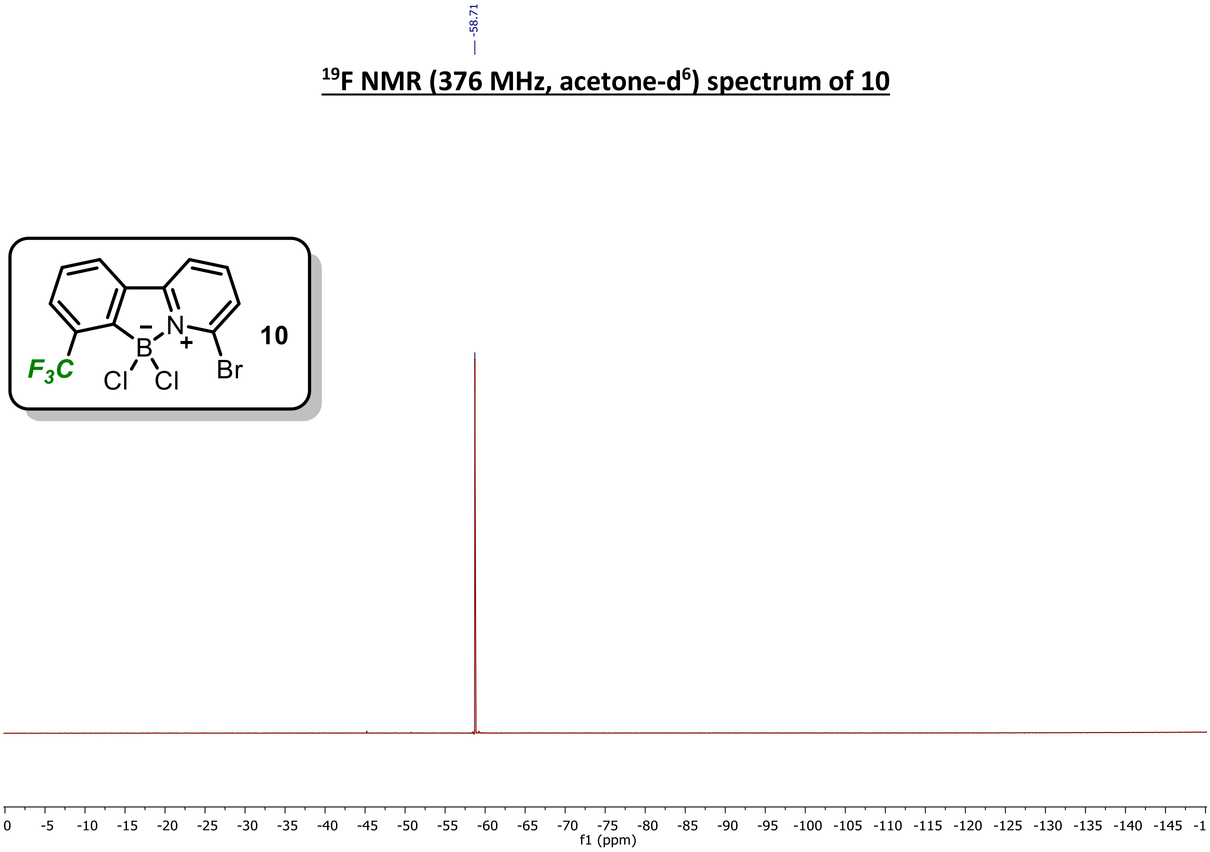

^{19}F NMR (376 MHz, acetone-d^{6}) spectrum of 10

^{11}B NMR (128 MHz, acetone-d^6) spectrum of 10

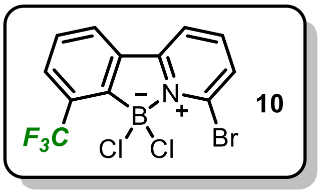

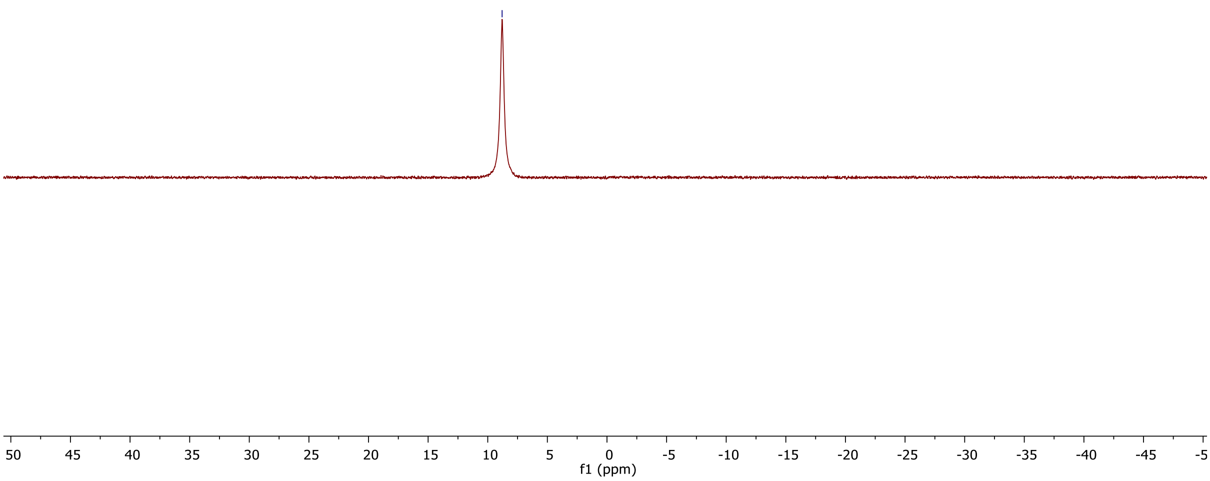

^{1}H NMR (400 MHz, DMSO-d^6) spectrum of 11

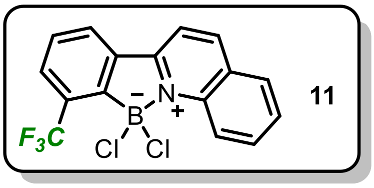

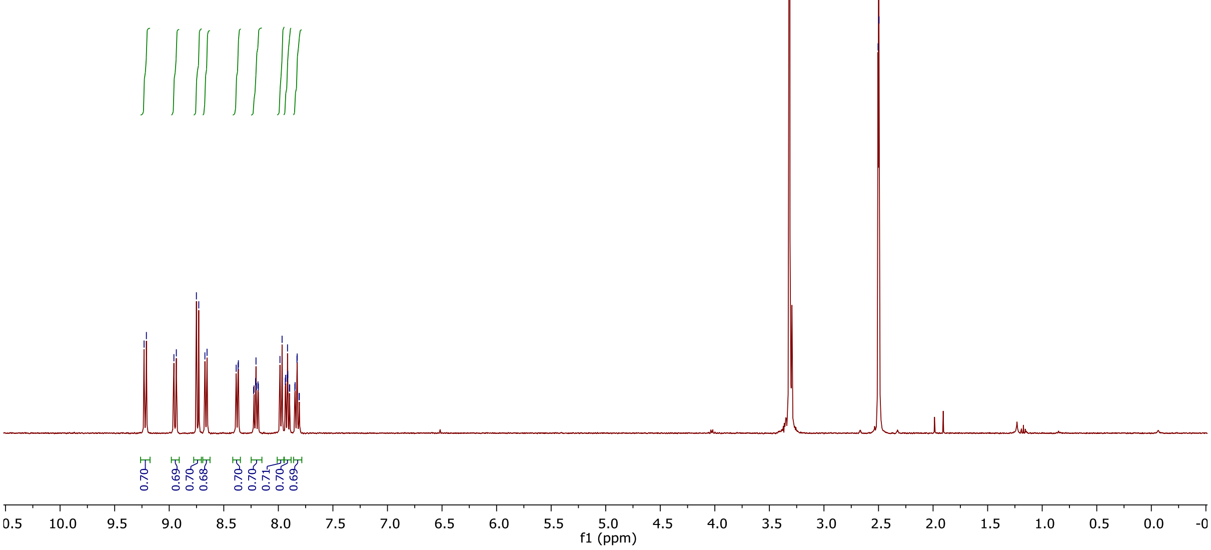

$^{13}C\{^{1}H\}$ NMR (101 MHz, DMSO-d^{6}) spectrum of 11

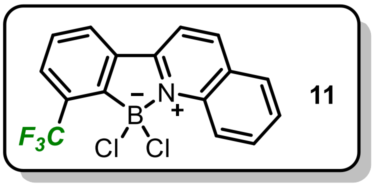

11

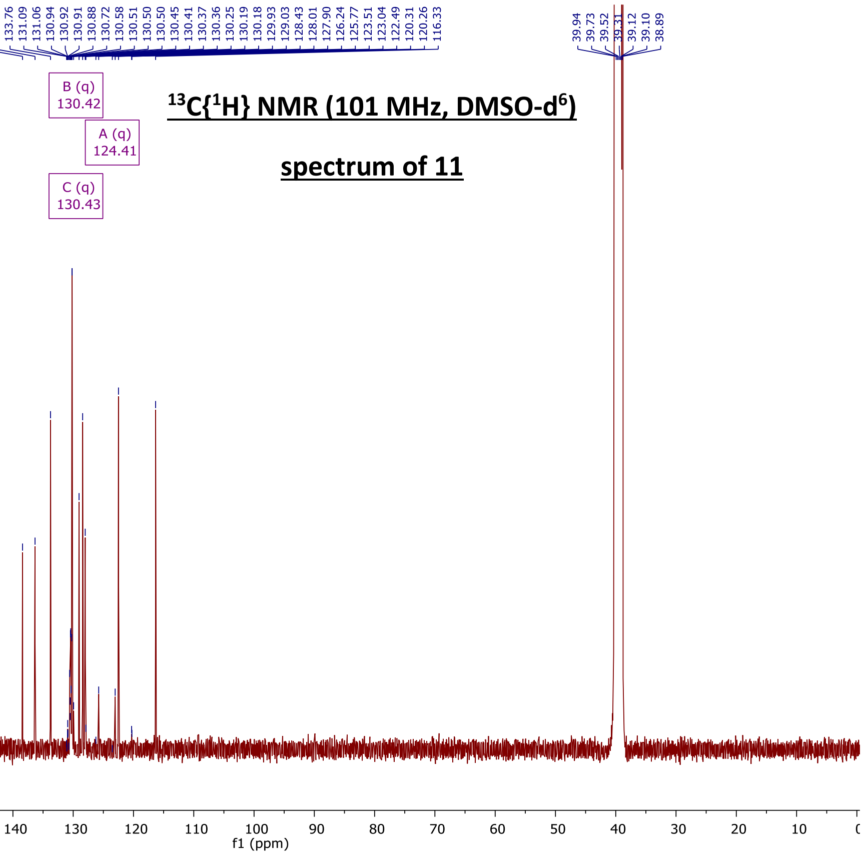

^{19}F NMR (376 MHz, DMSO-d^{6}) spectrum of 11

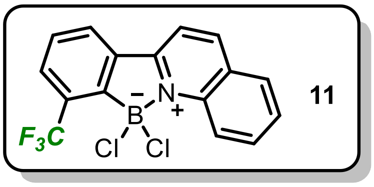

11

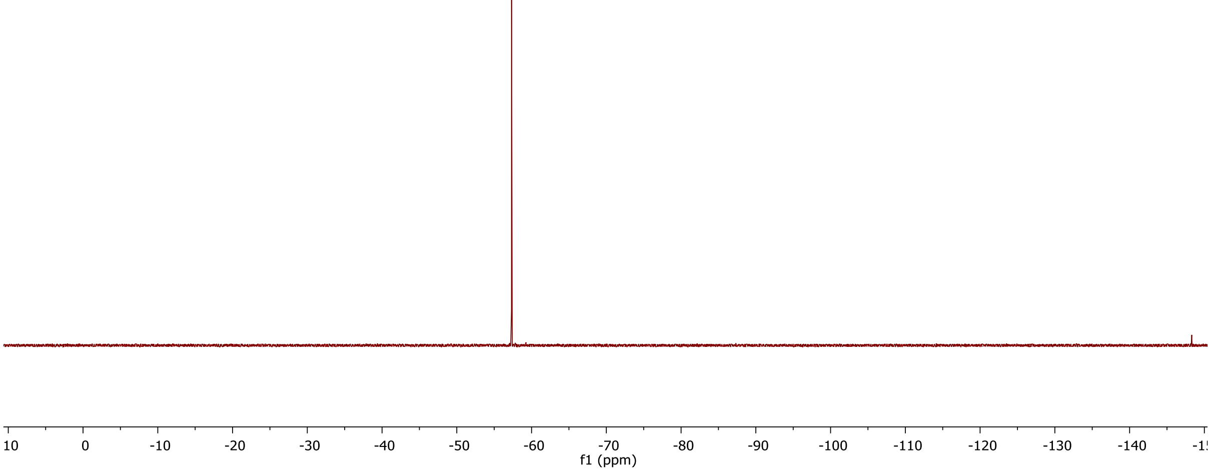

^{11}B NMR (128 MHz, DMSO-d^{6}) spectrum of 11

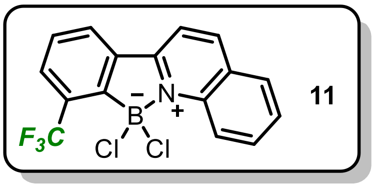

11

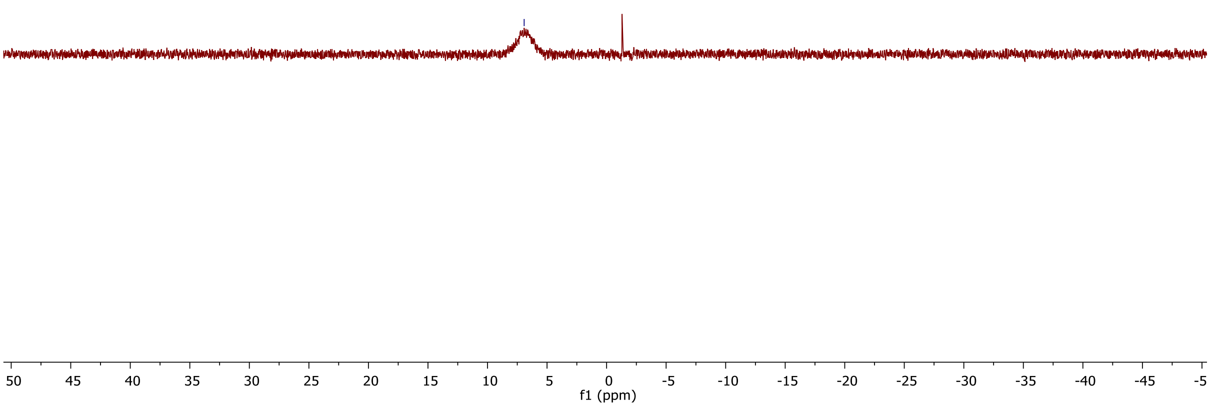

^{1}H NMR (400 MHz, acetone-d^{6}) spectrum of 12

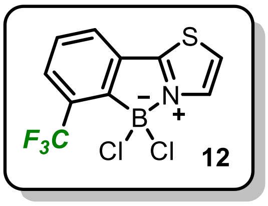

12

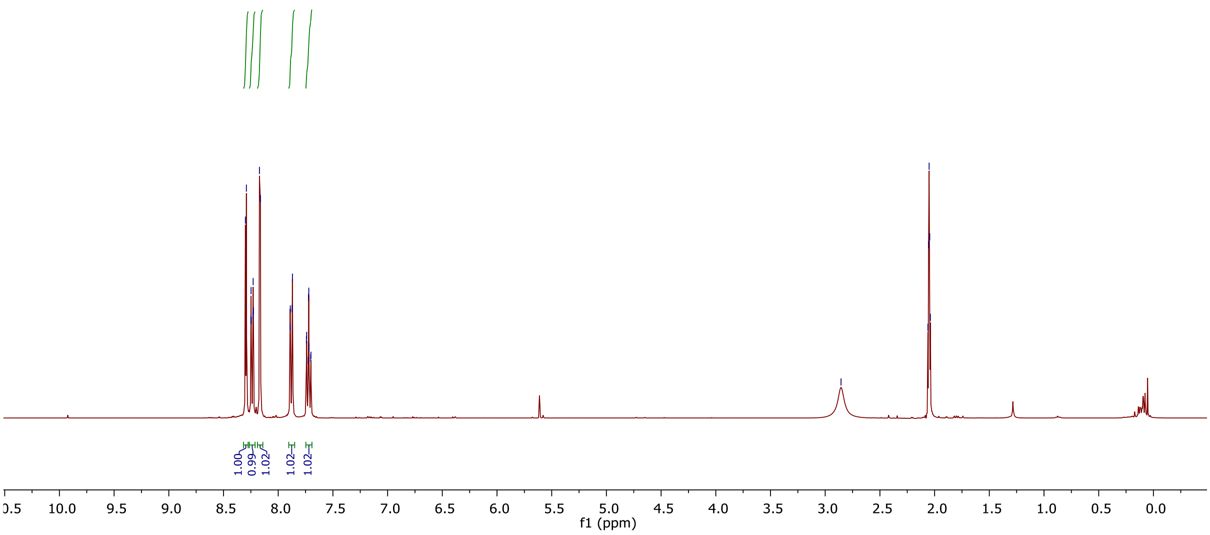

$^{13}C\{^{1}H\}$ NMR (101 MHz, acetone-d^{6}) spectrum of 12

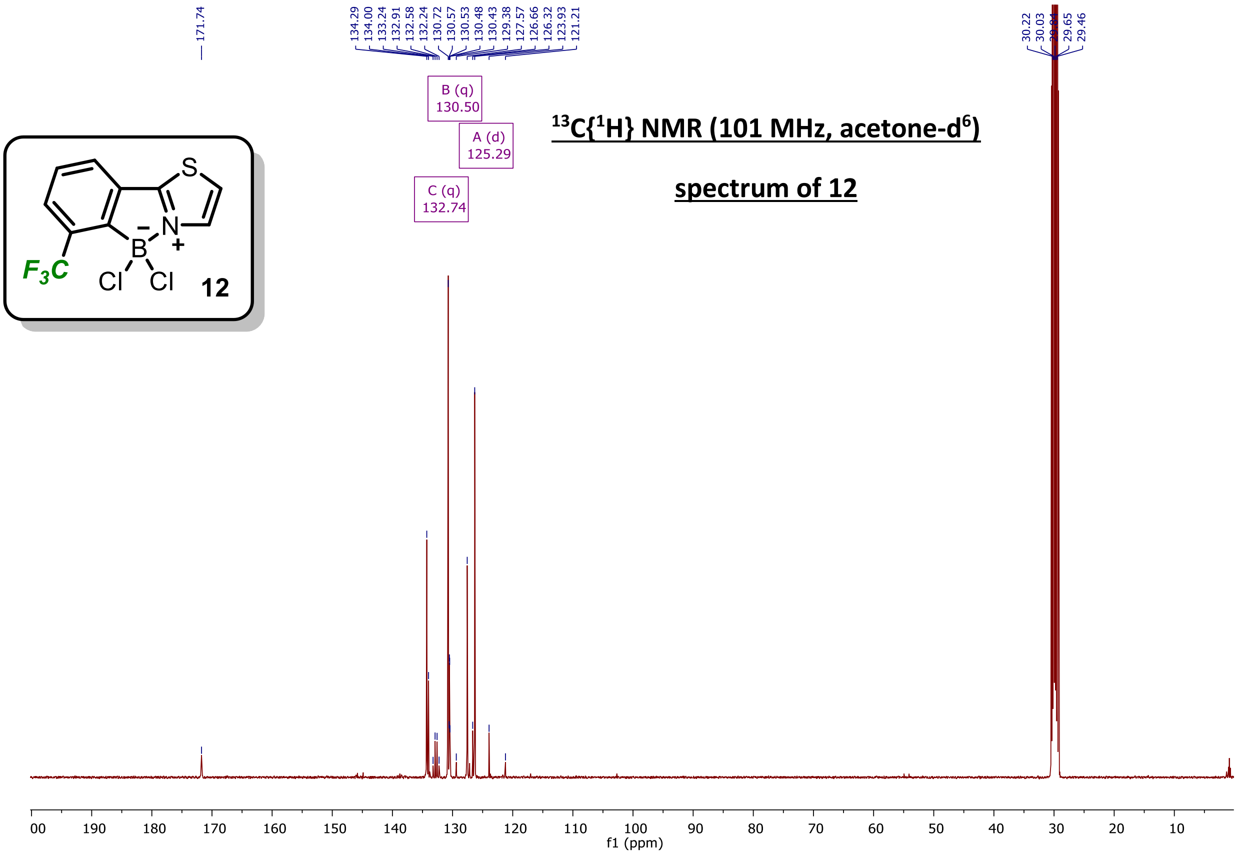

^{19}F NMR (376 MHz, acetone-d^{6}) spectrum of 12

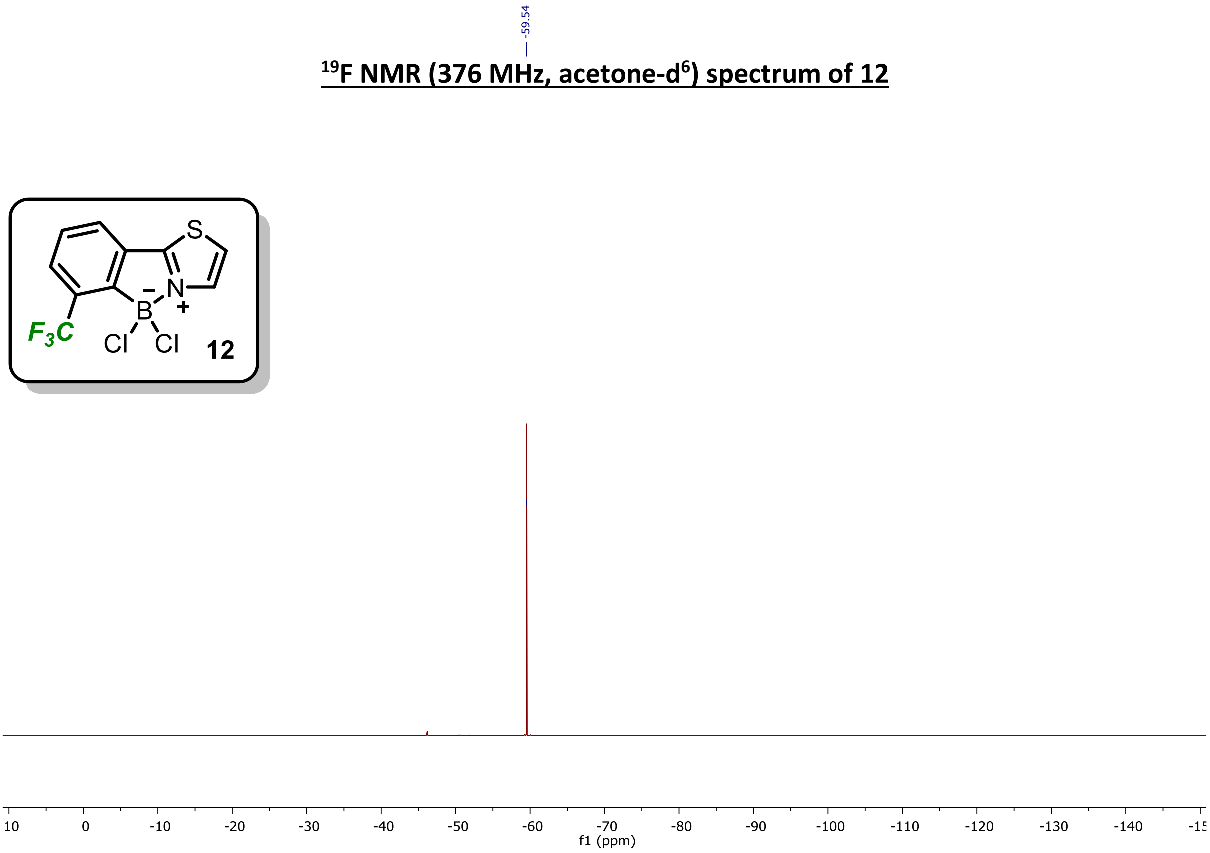

^{11}B NMR (128 MHz, acetone-d^6) spectrum of 12

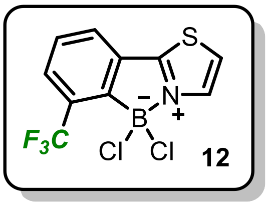

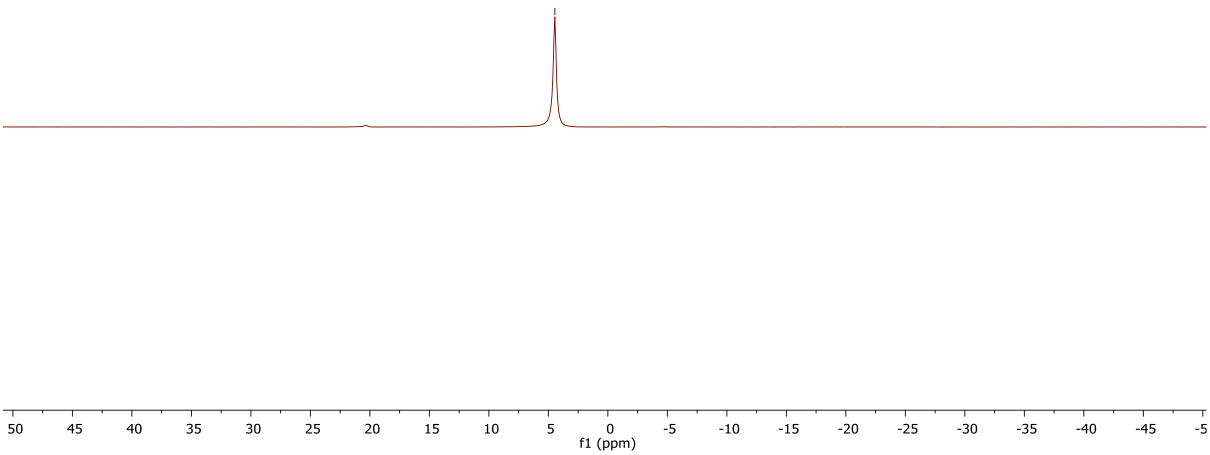

^{1}H NMR (400 MHz, acetone-d^6) spectrum of 13

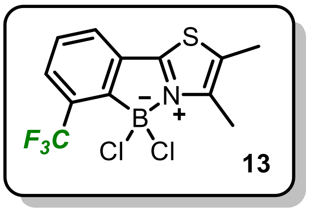

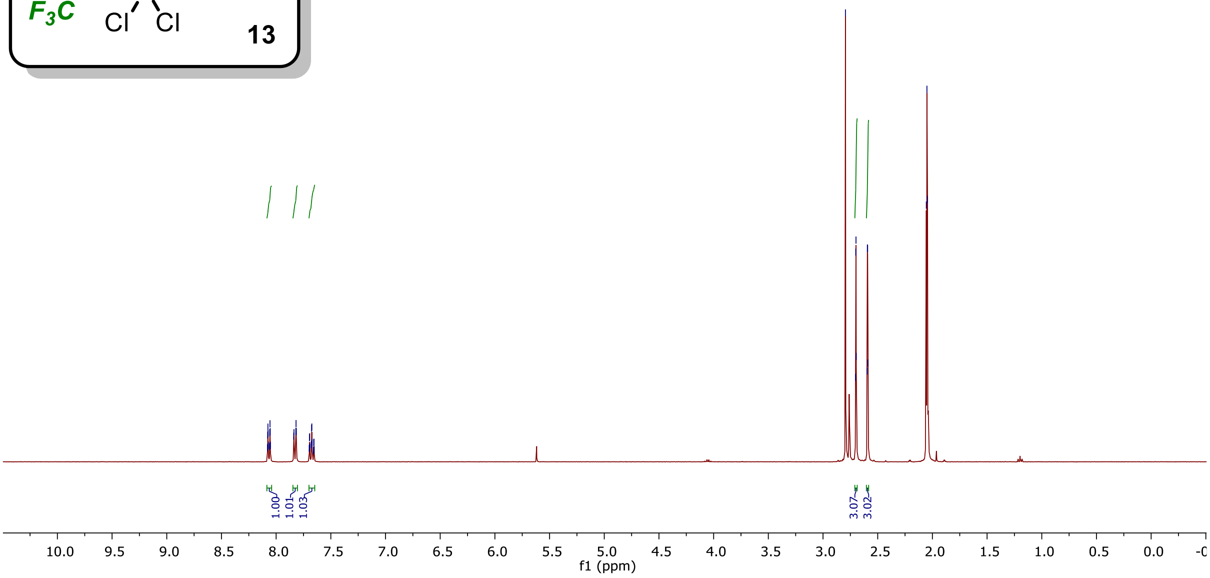

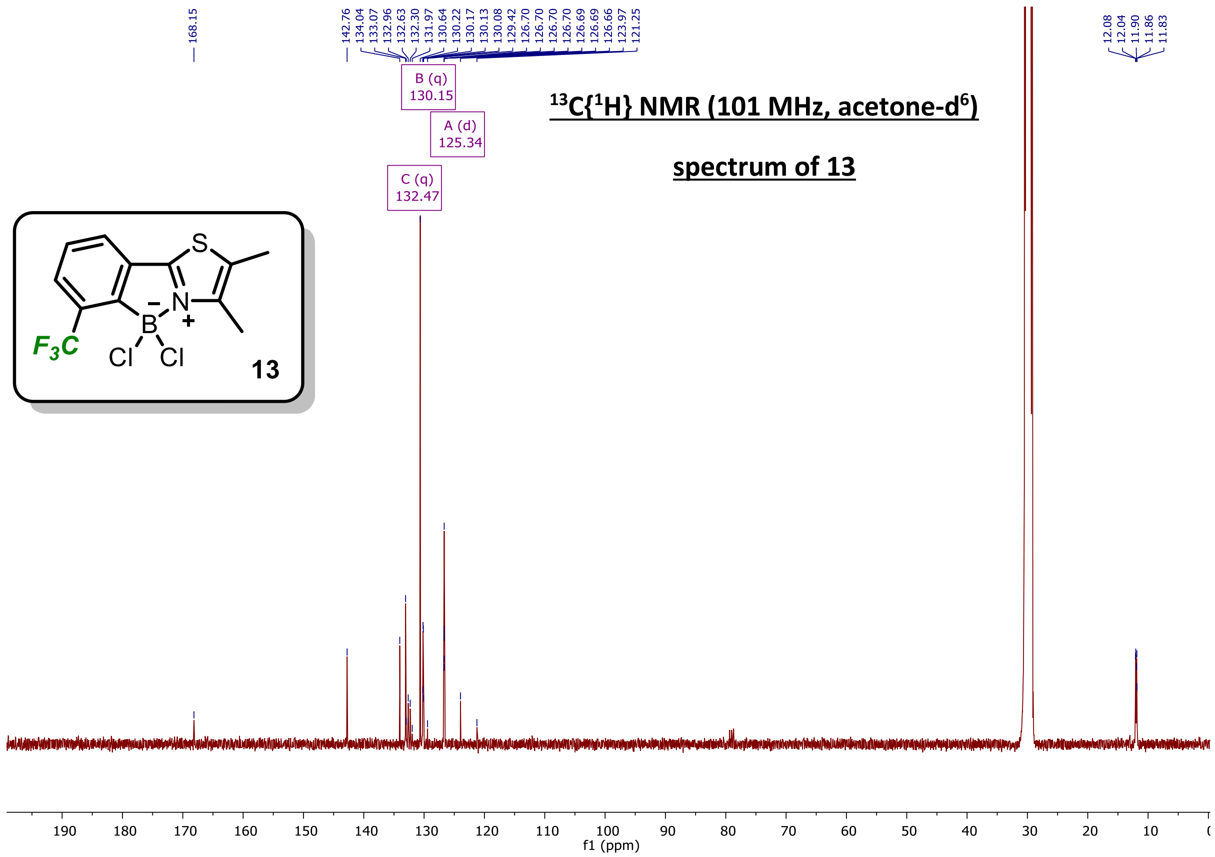

13C{1H} NMR (101 MHz, acetone-d6)
spectrum of 13
168.15
142.76
134.04
133.07
132.96
132.63
132.30
131.97
130.64
130.22
130.17
130.13
130.08
129.42
126.70
126.70
126.70
126.70
126.69
126.69
126.66
123.97
121.25
12.08
12.04
11.90
11.86
11.83
B (q)
130.15
A (d)
125.34
C (q)
132.47
F3C
S
N
B
Cl
Cl
13
f1 (ppm)

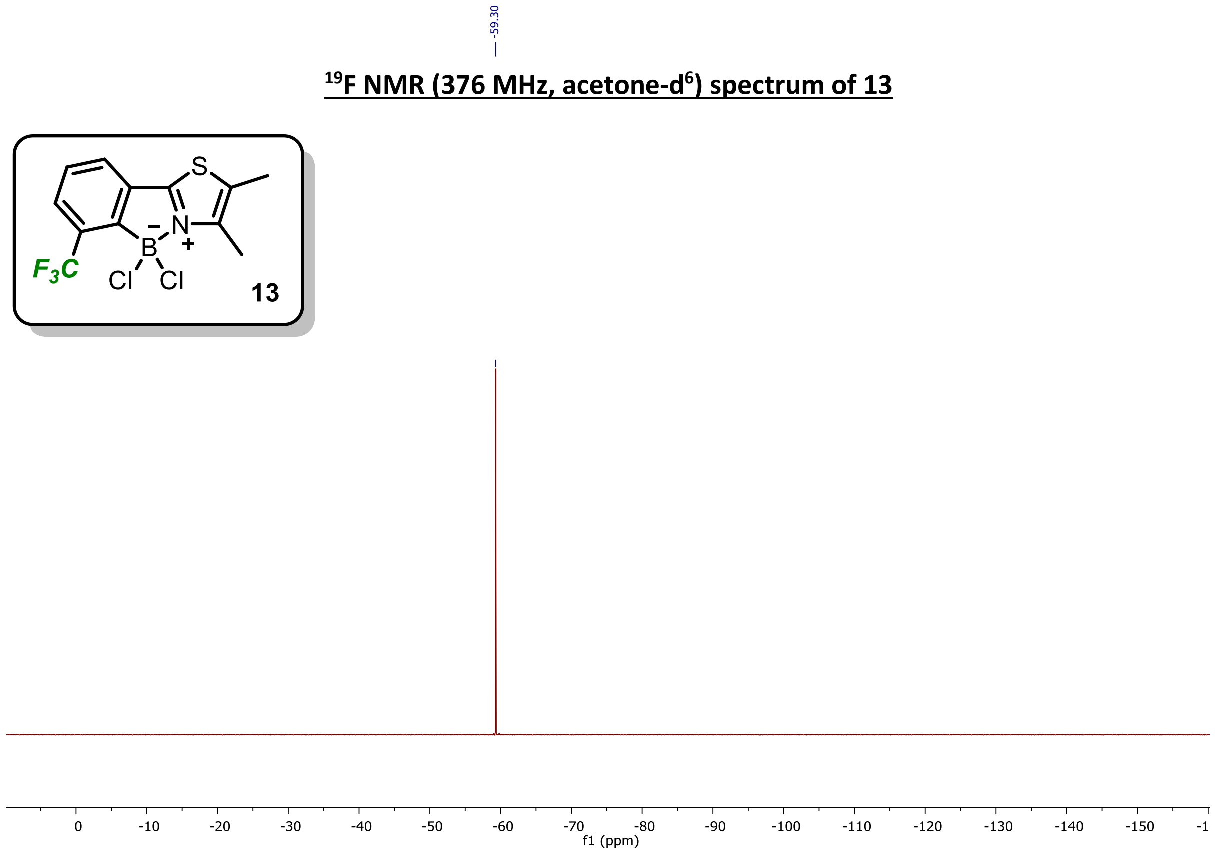

-59.30
19F NMR (376 MHz, acetone-d6) spectrum of 13
F3C
S
N
B
Cl
Cl
13
f1 (ppm)

^{11}B NMR (128 MHz, acetone-d^6) spectrum of 13

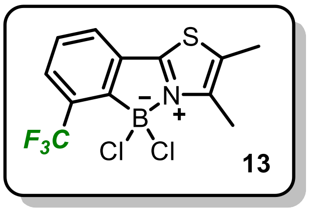

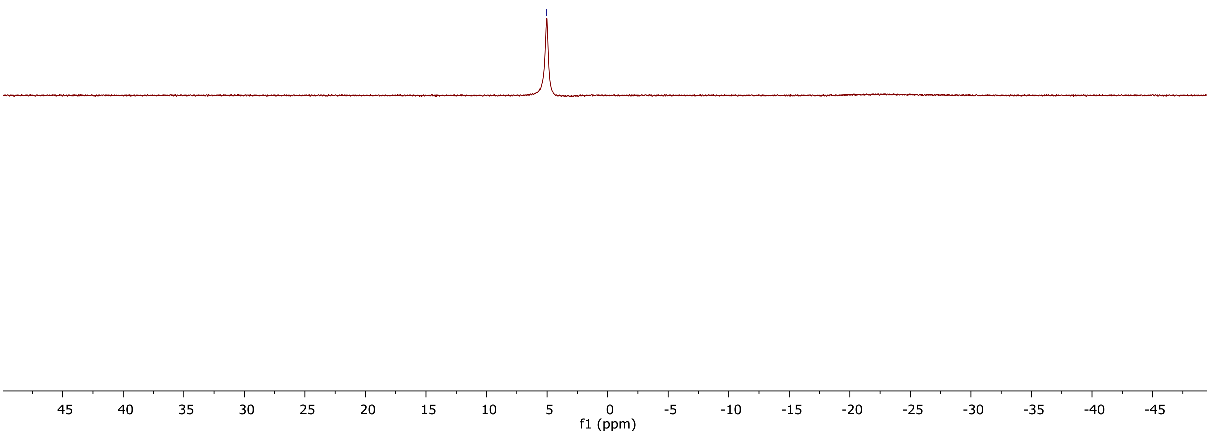

^{1}H NMR (400 MHz, DMSO-d^6) spectrum of 14

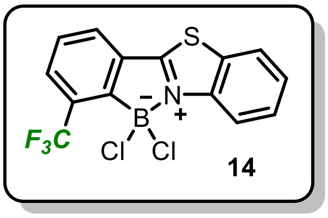

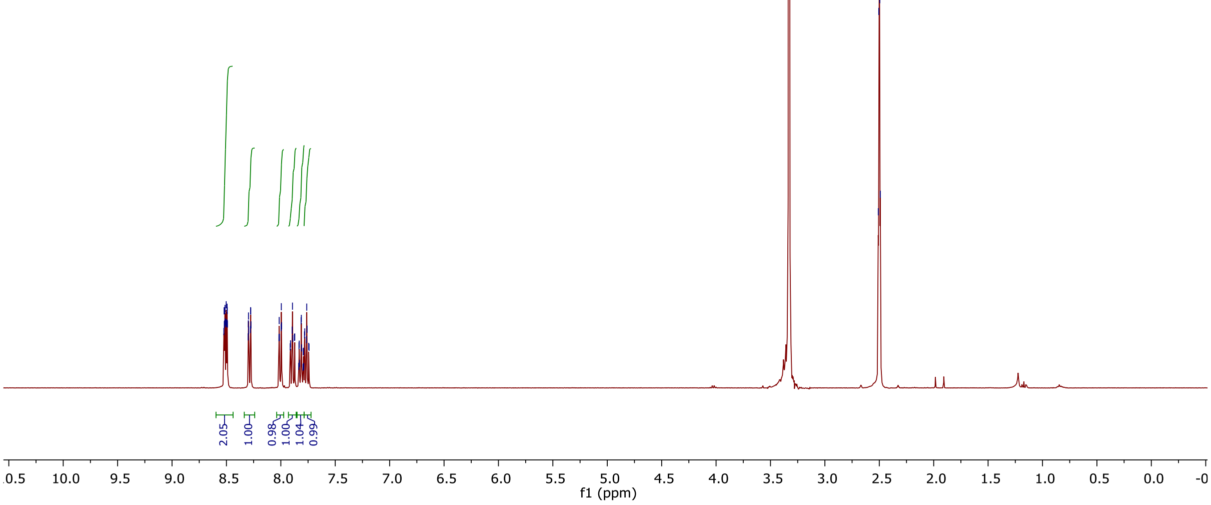

$^{13}C\{^{1}H\}$ NMR (101 MHz, DMSO-d^6) spectrum of 14

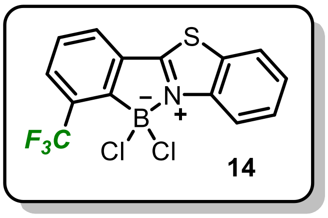

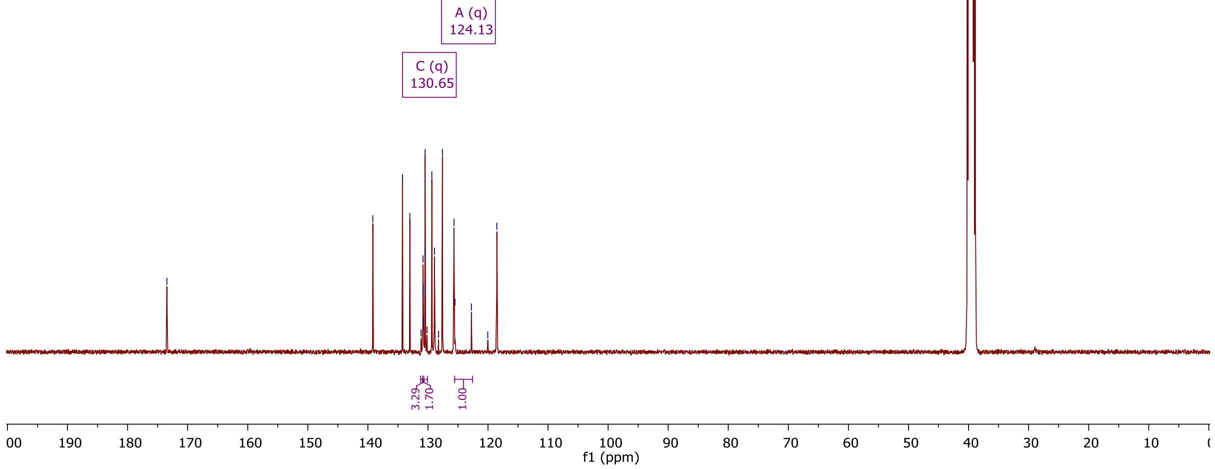

^{19}F NMR (376 MHz, DMSO-d^6) spectrum of 14

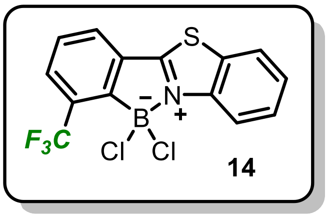

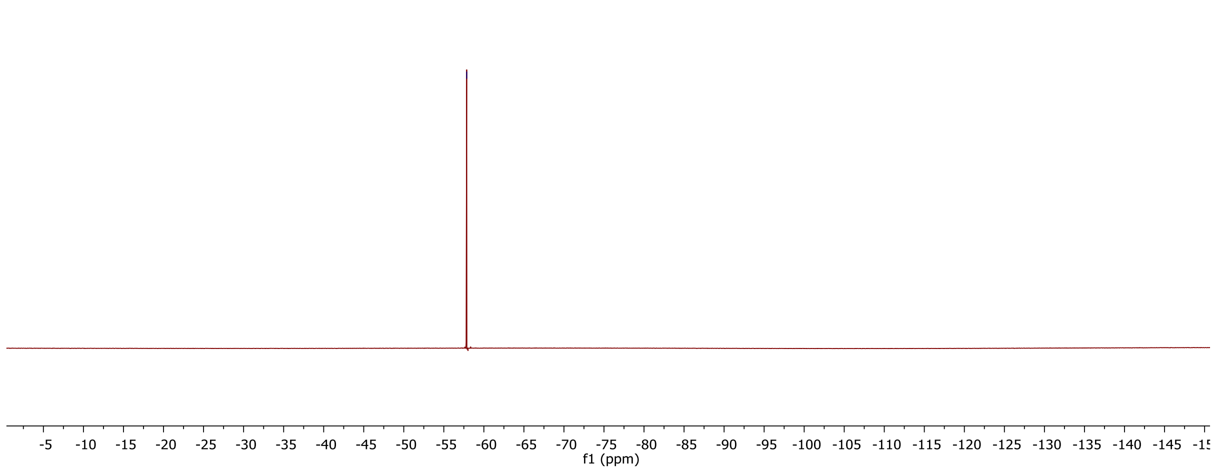

^{11}B NMR (128 MHz, DMSO-d^6) spectrum of 14

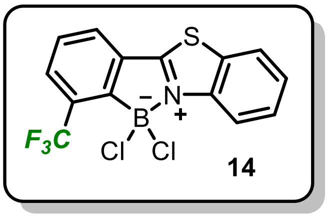

14

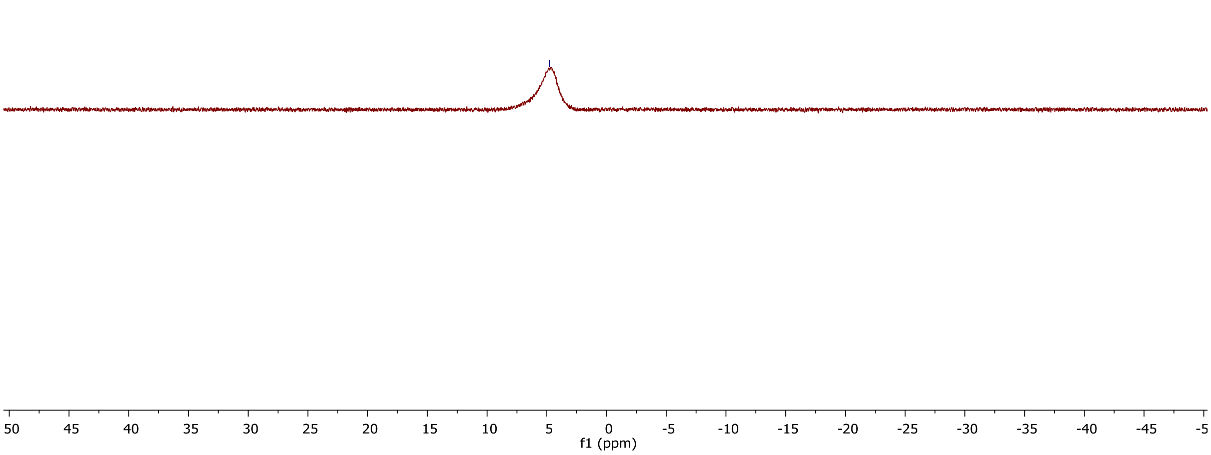

^{1}H NMR (400 MHz, acetone-d^6) spectrum of 15

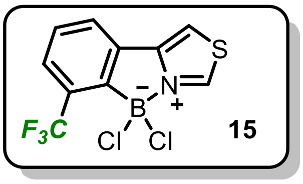

15

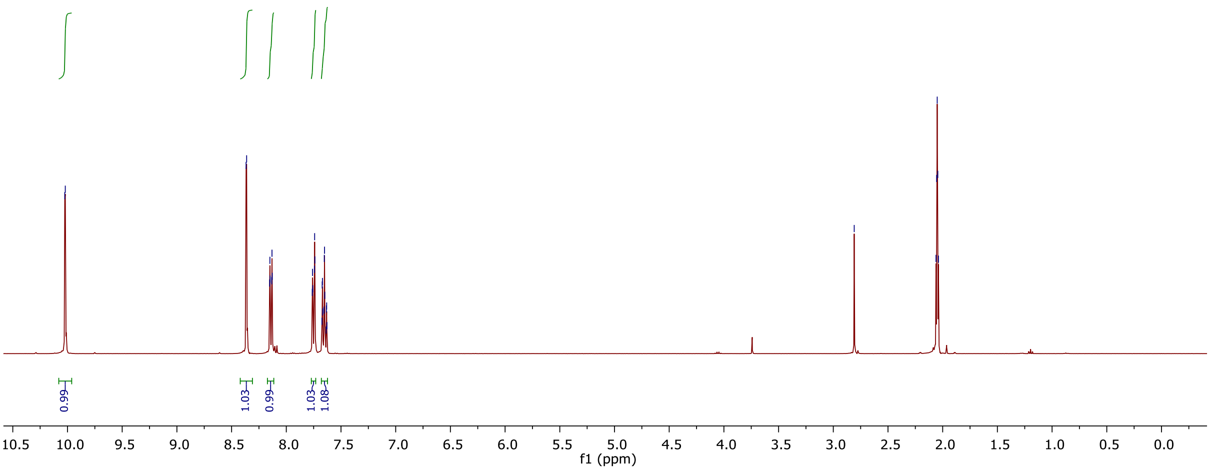

$^{13}C\{^1H\}$ NMR (101 MHz, acetone-d^6)

spectrum of 15

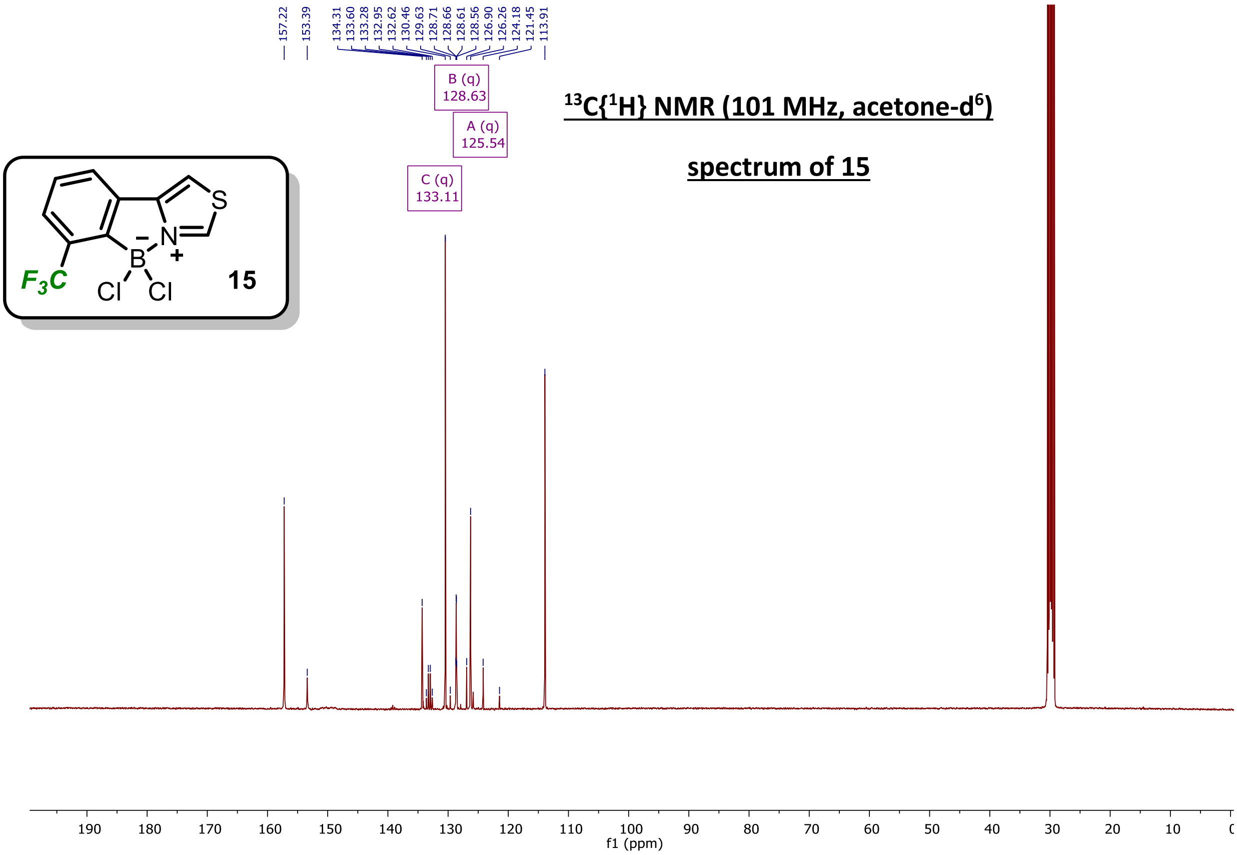

^{19}F NMR (376 MHz, acetone-d^6) spectrum of 15

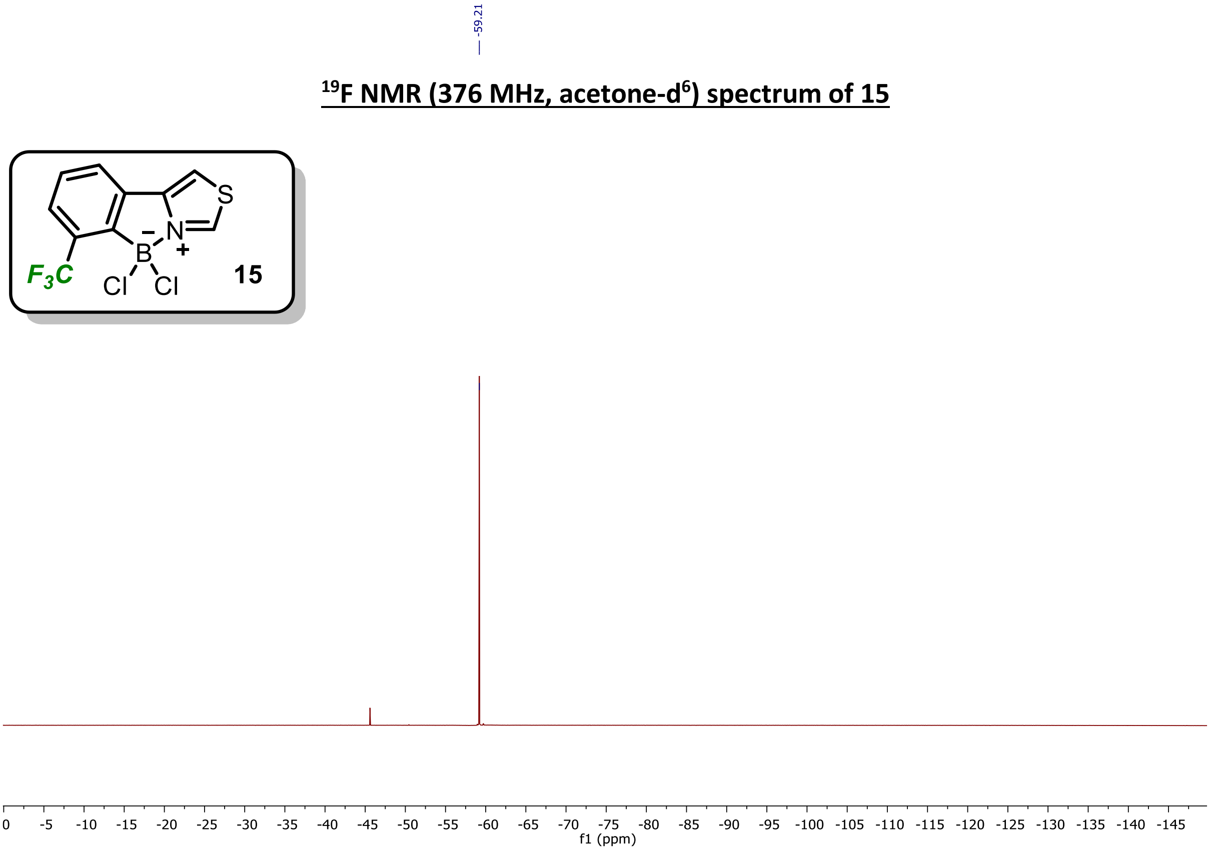

^{11}B NMR (128 MHz, acetone-d[6]) spectrum of 15

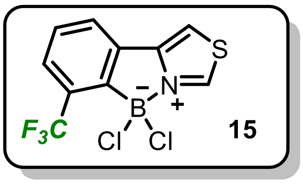

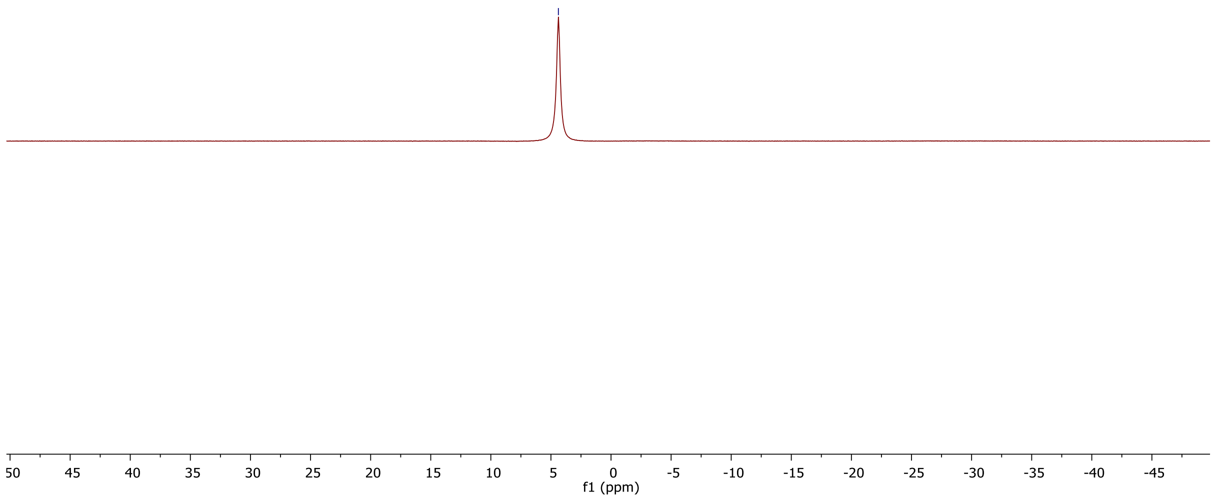

^{1}H NMR (400 MHz, acetone-d[6]) spectrum of 16

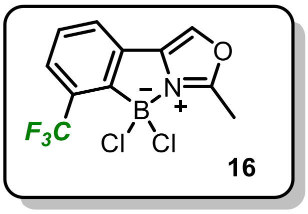

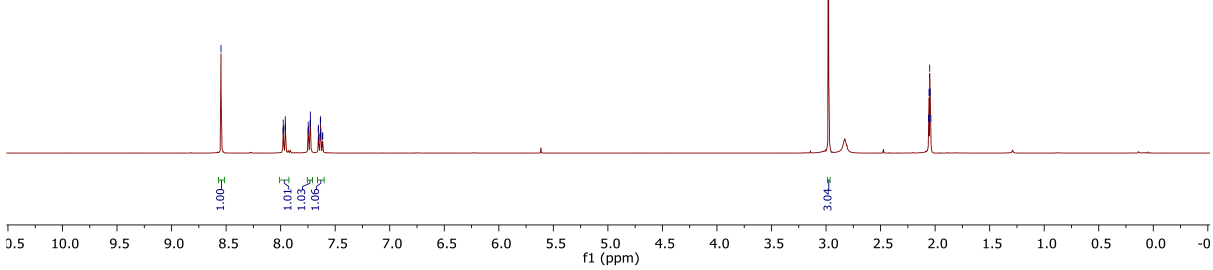

$^{13}C\{^{1}H\}$ NMR (101 MHz, acetone-d^{6}) spectrum of 16

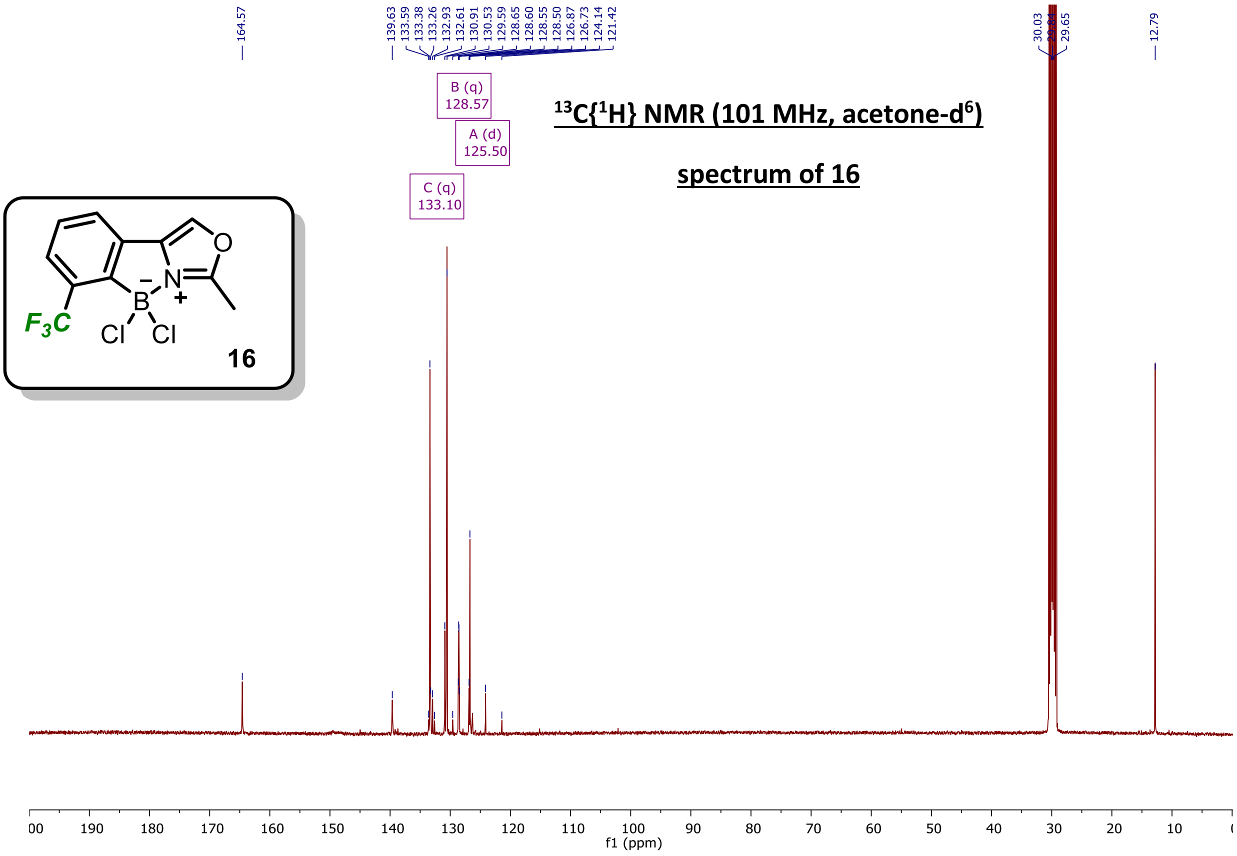

^{19}F NMR (376 MHz, acetone-d^{6}) spectrum of 16

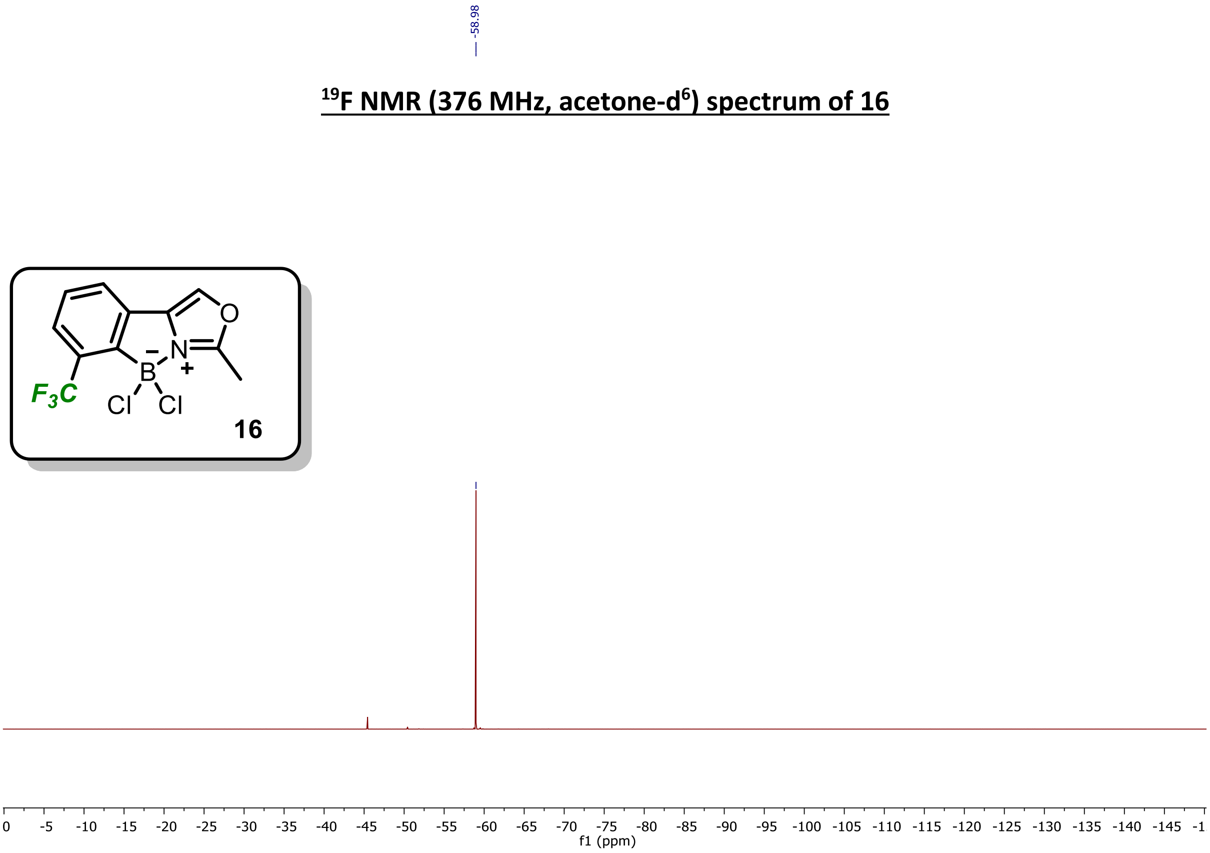

^{11}B NMR (128 MHz, acetone-d^6) spectrum of 16

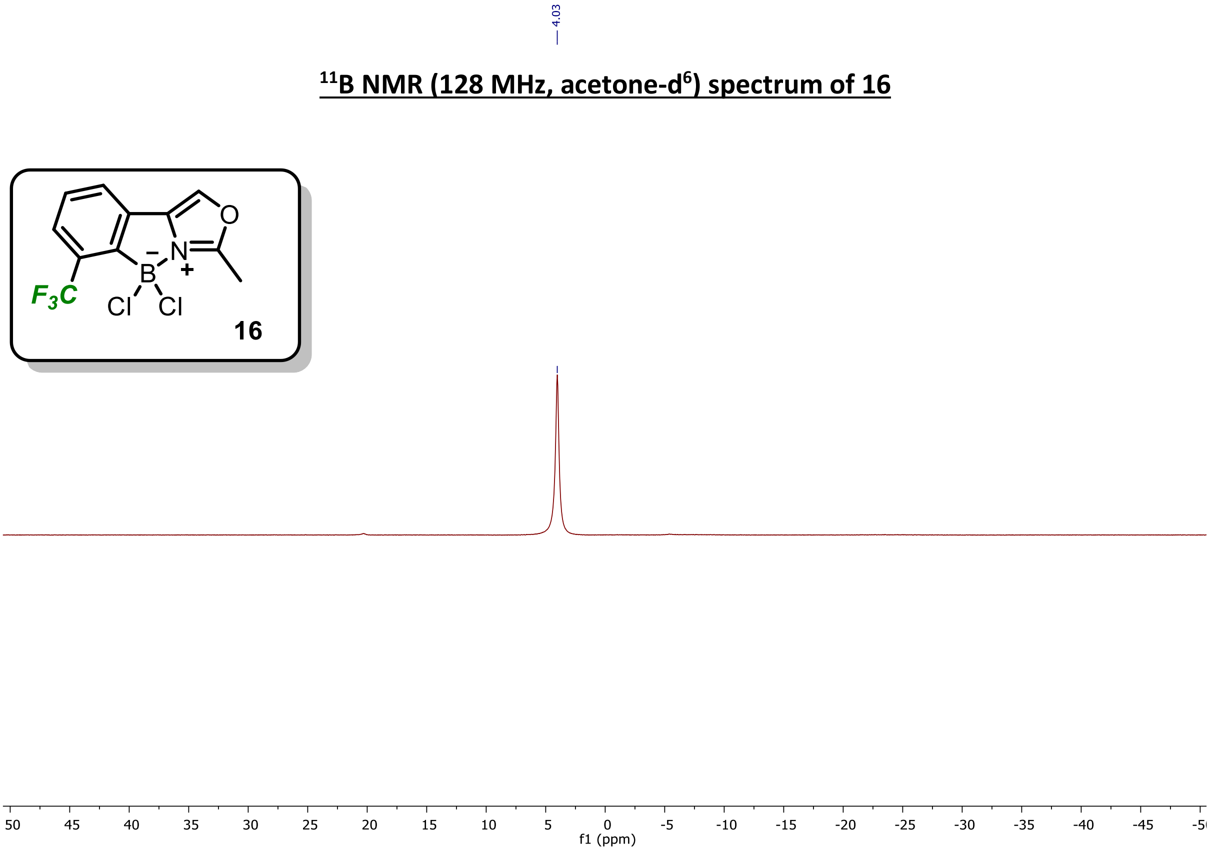

^{1}H NMR (400 MHz, acetone-d^6) spectrum of 17

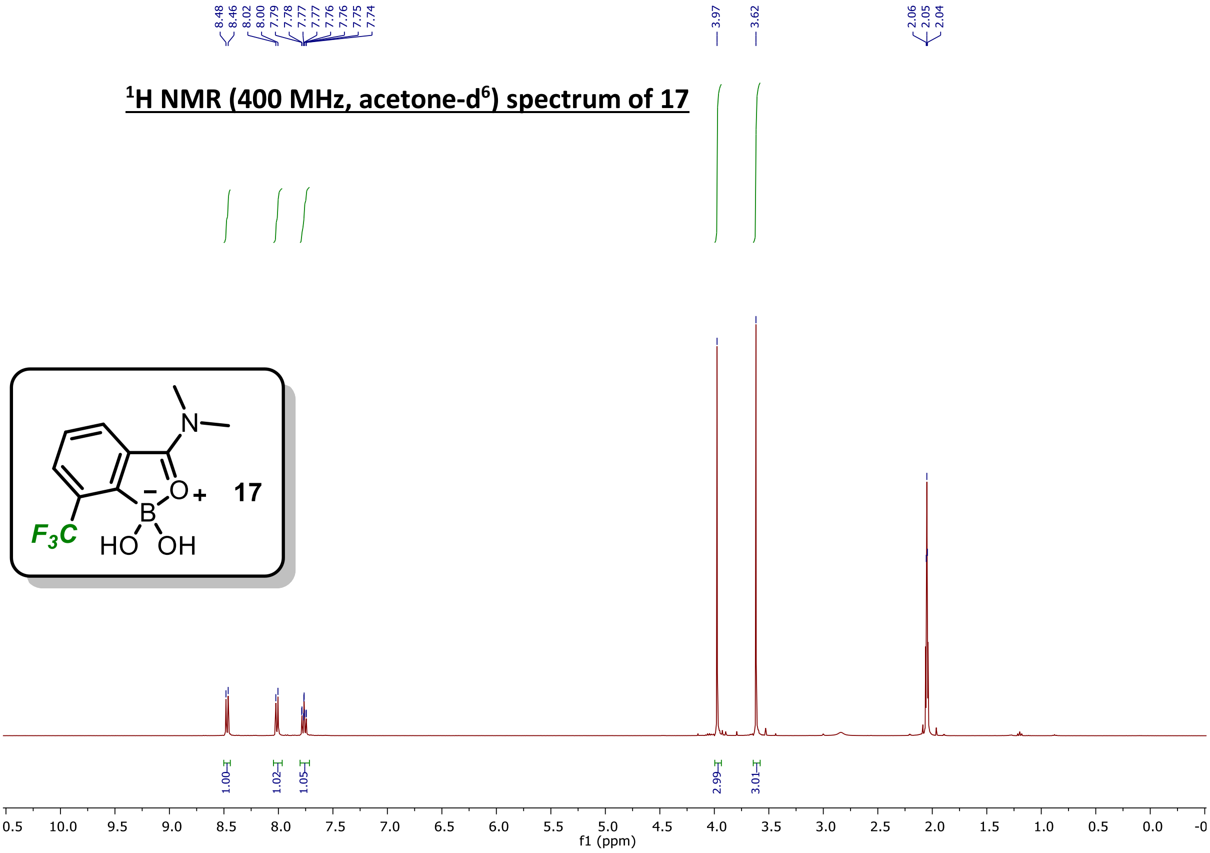

$^{13}C\{^{1}H\}$ NMR (101 MHz, acetone-d^{6}) spectrum of 17

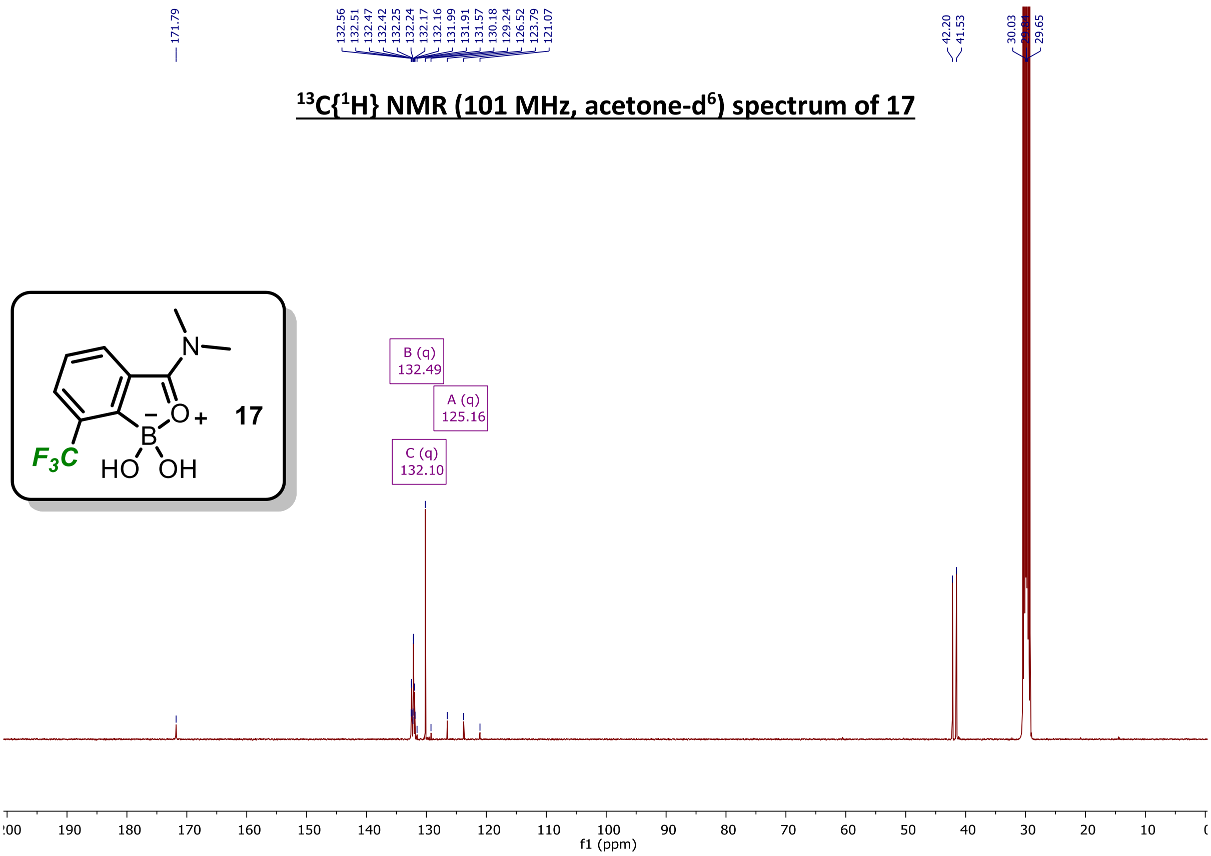

^{19}F NMR (376 MHz, acetone-d^{6}) spectrum of 17

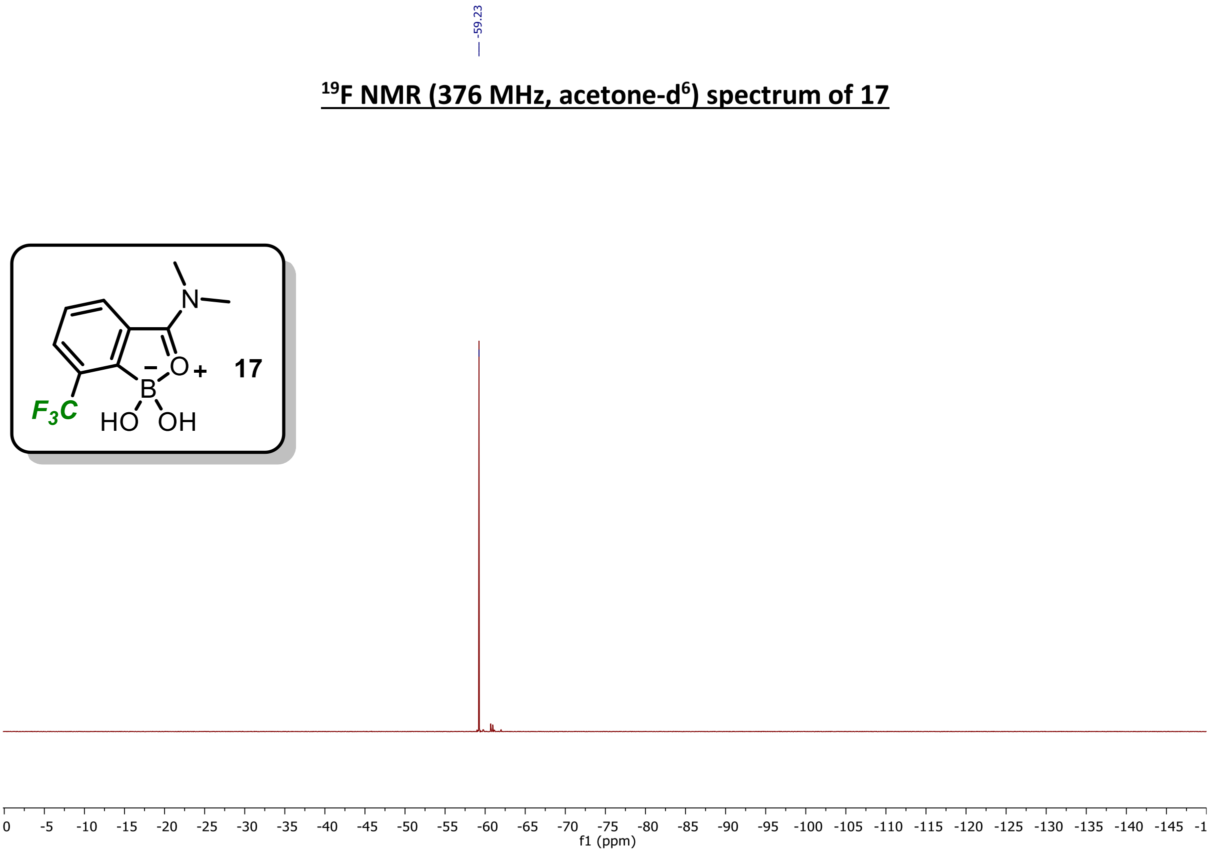

^{11}B NMR (128 MHz, acetone-d^6) spectrum of 17

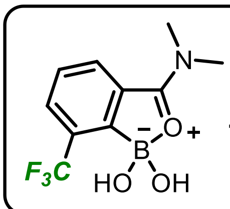

17

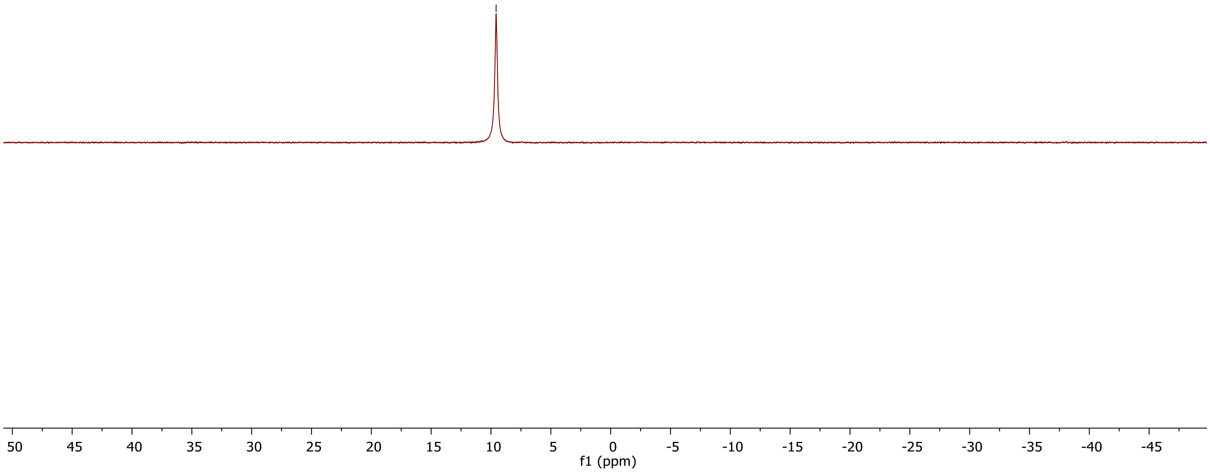

^{1}H NMR (400 MHz, CD_3CN) spectrum of 18

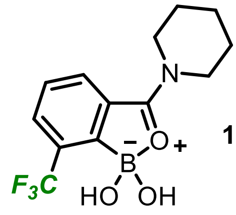

18

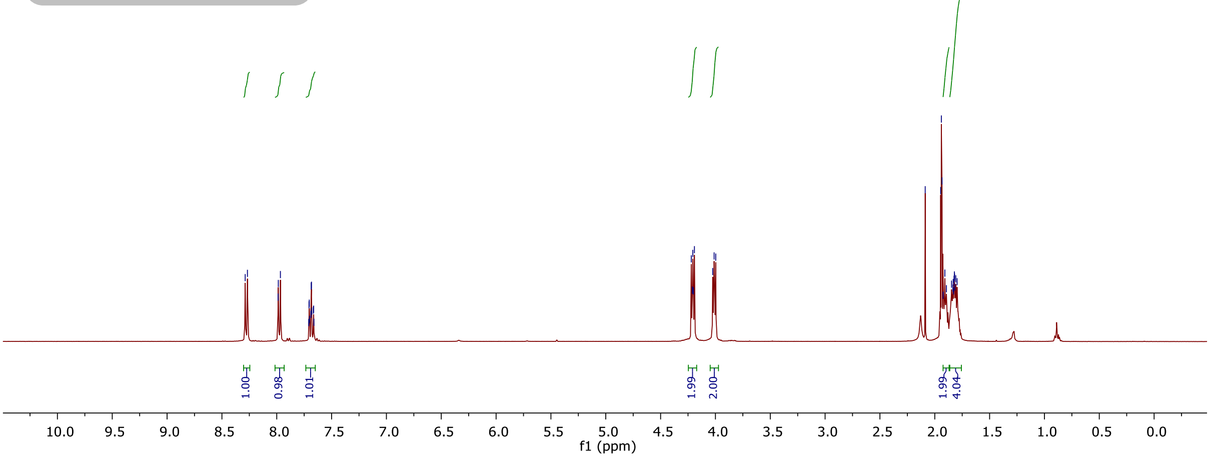

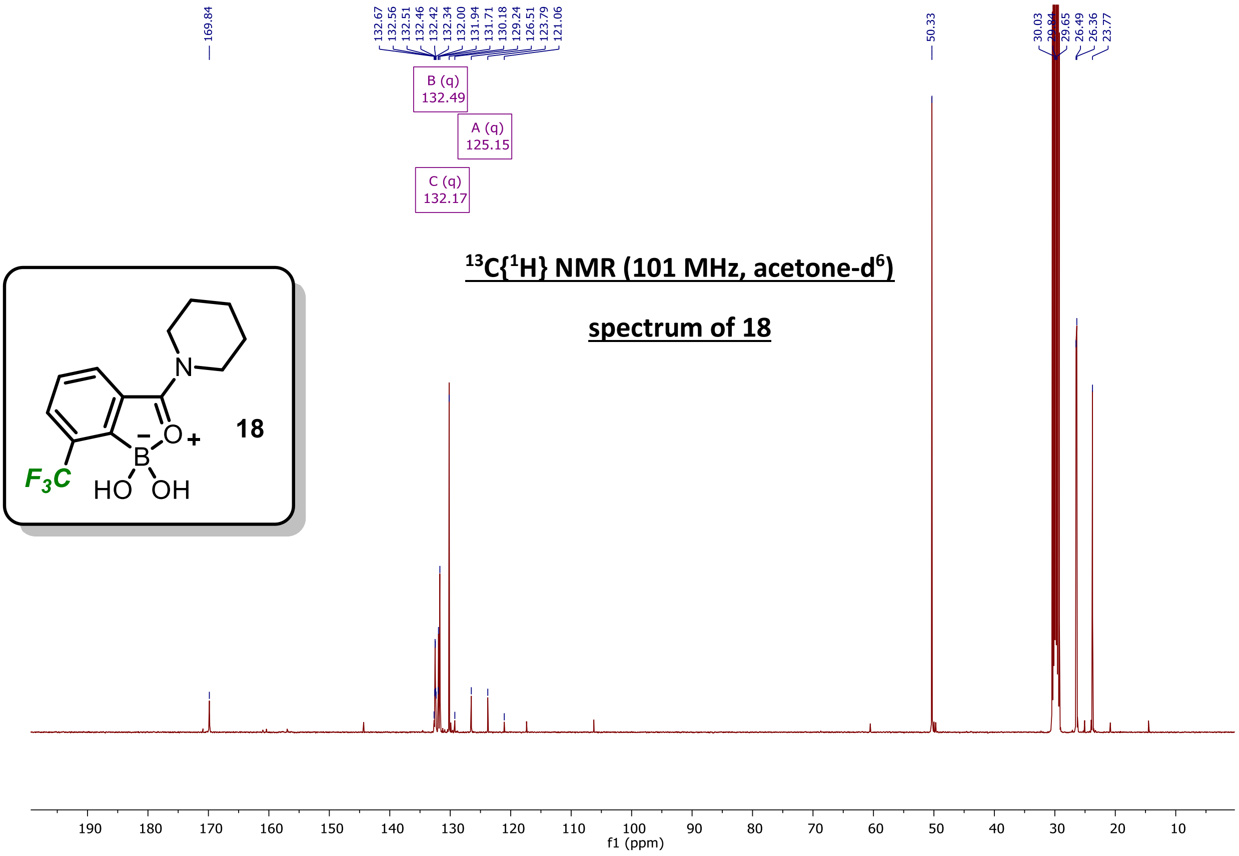

$^{13}C\{^{1}H\}$ NMR (101 MHz, acetone-d^{6}) spectrum of 18

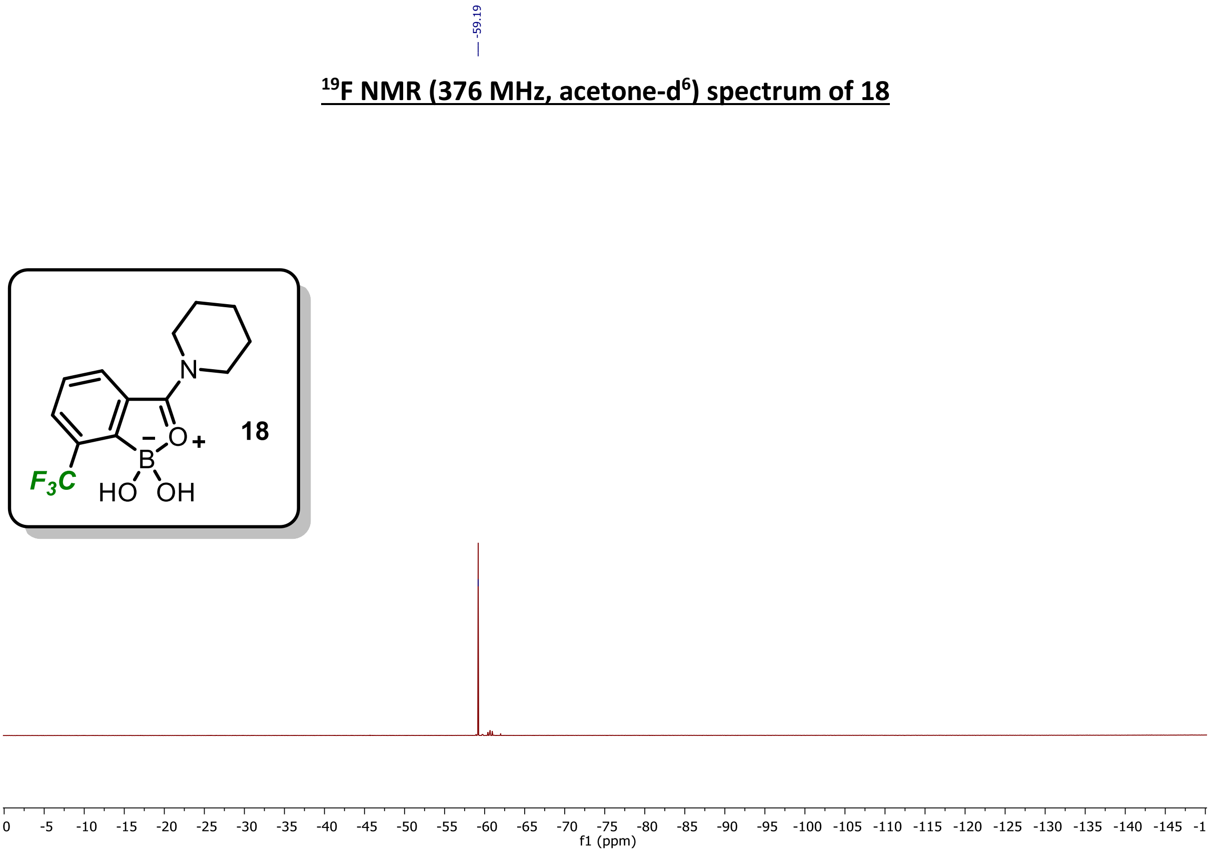

^{19}F NMR (376 MHz, acetone-d^{6}) spectrum of 18

^{11}B NMR (128 MHz, acetone-d^{6}) spectrum of 18

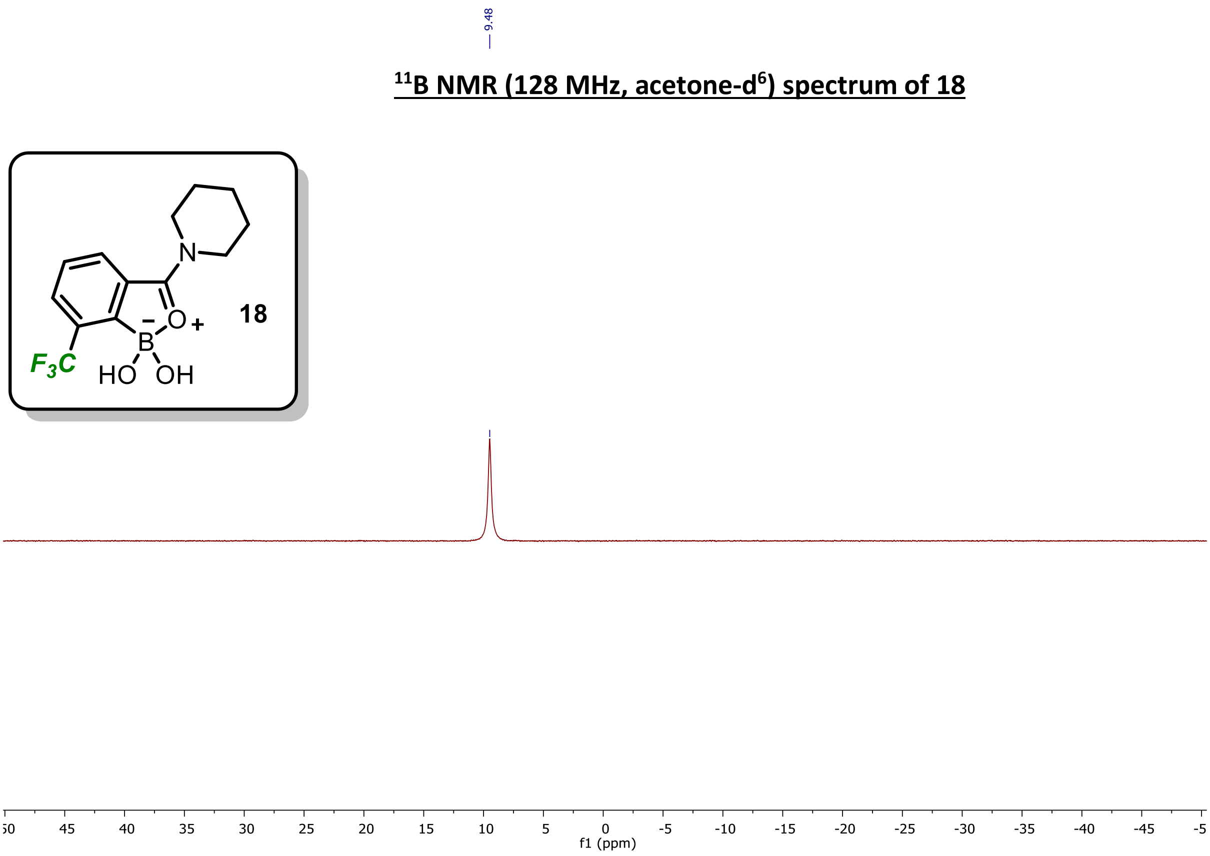

^{1}H NMR (400 MHz, CD_3CN) spectrum of 19

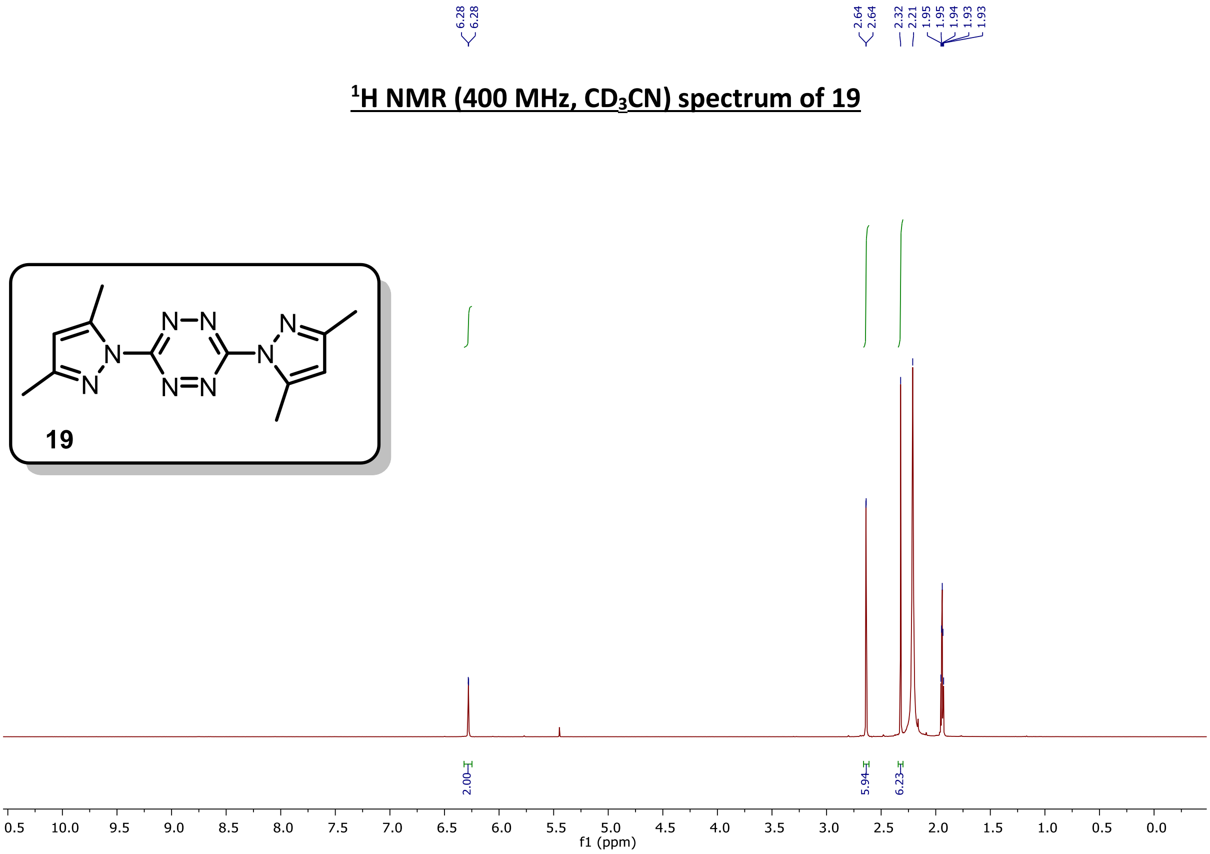

^{1}H NMR (400 MHz, CD_3CN) spectrum of 20

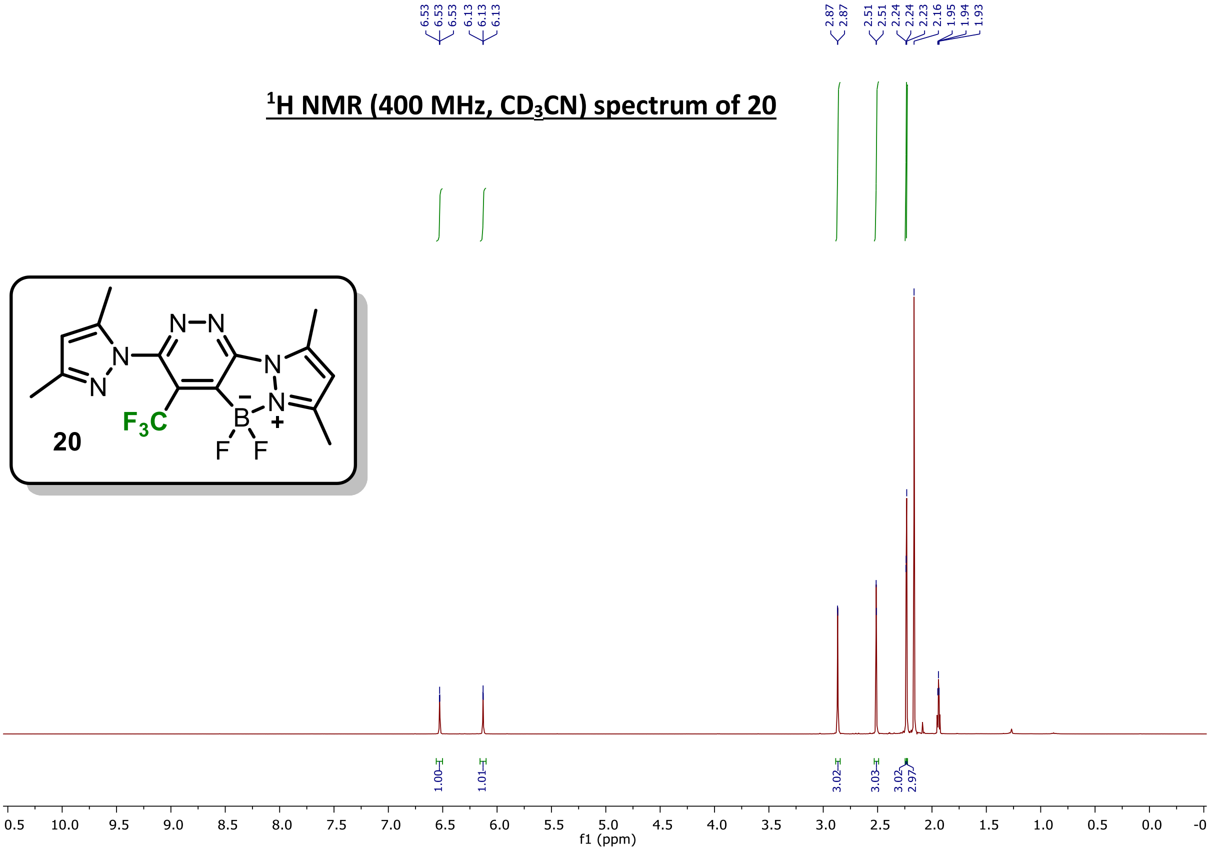

^{13}C{^{1}H} NMR (101 MHz, CD_3CN) spectrum of 20

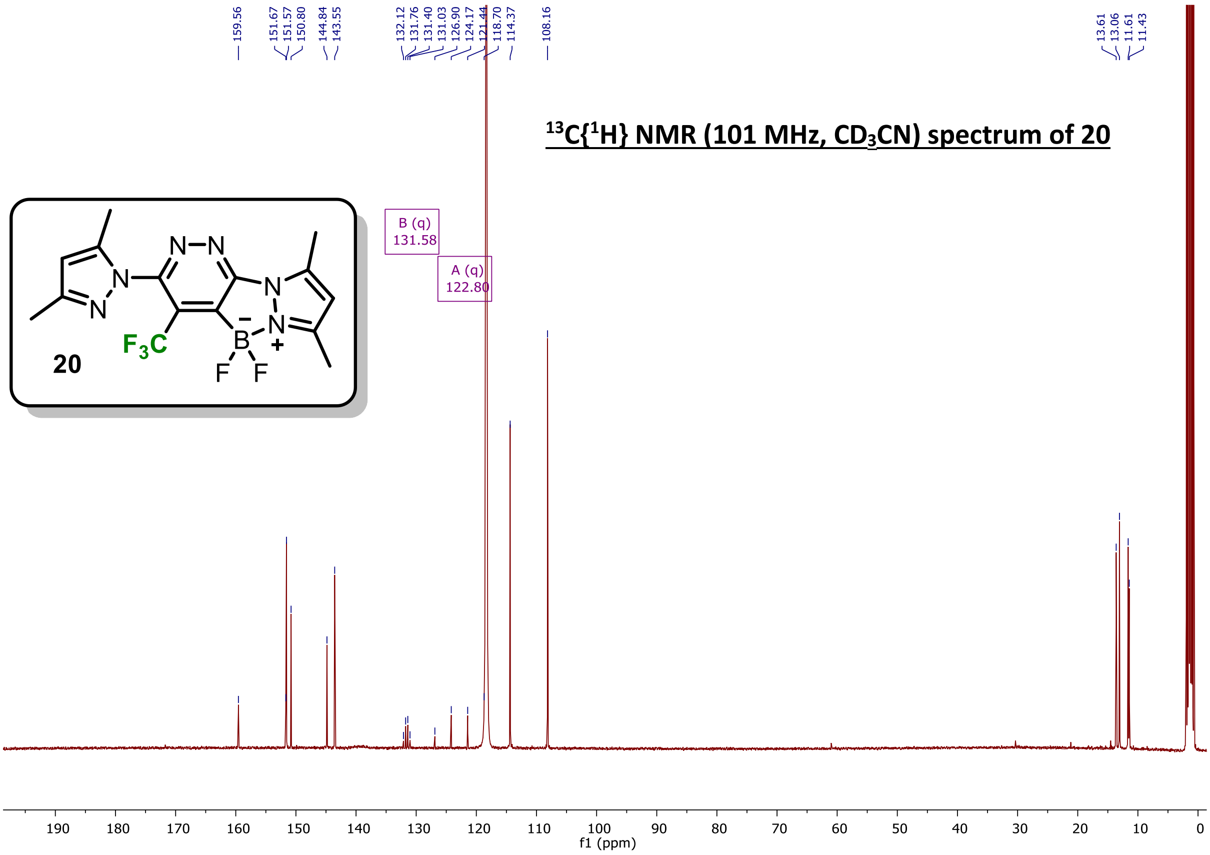

<u>**^{19}F NMR (376 MHz, CD_3CN) spectrum of 20**</u>

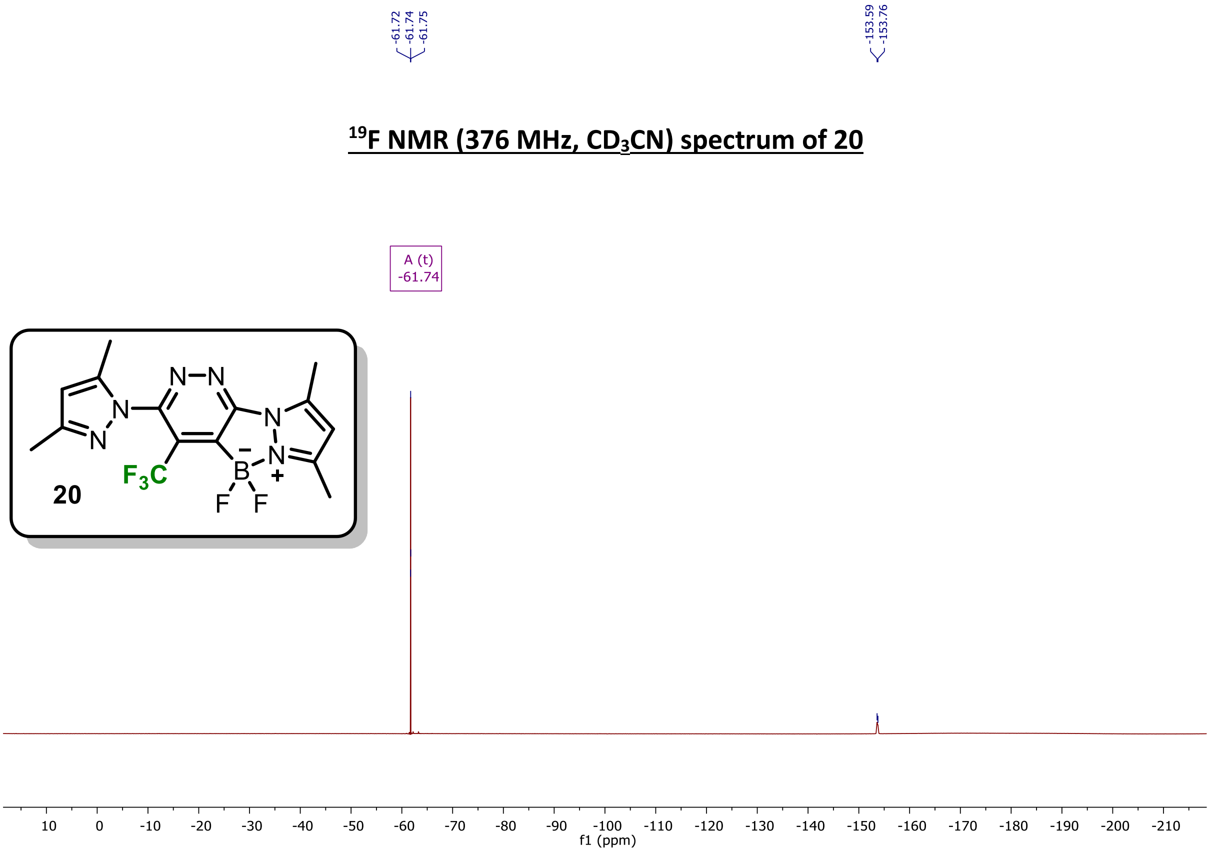

<u>**^{11}B NMR (128 MHz, CD_3CN) spectrum of 20**</u>

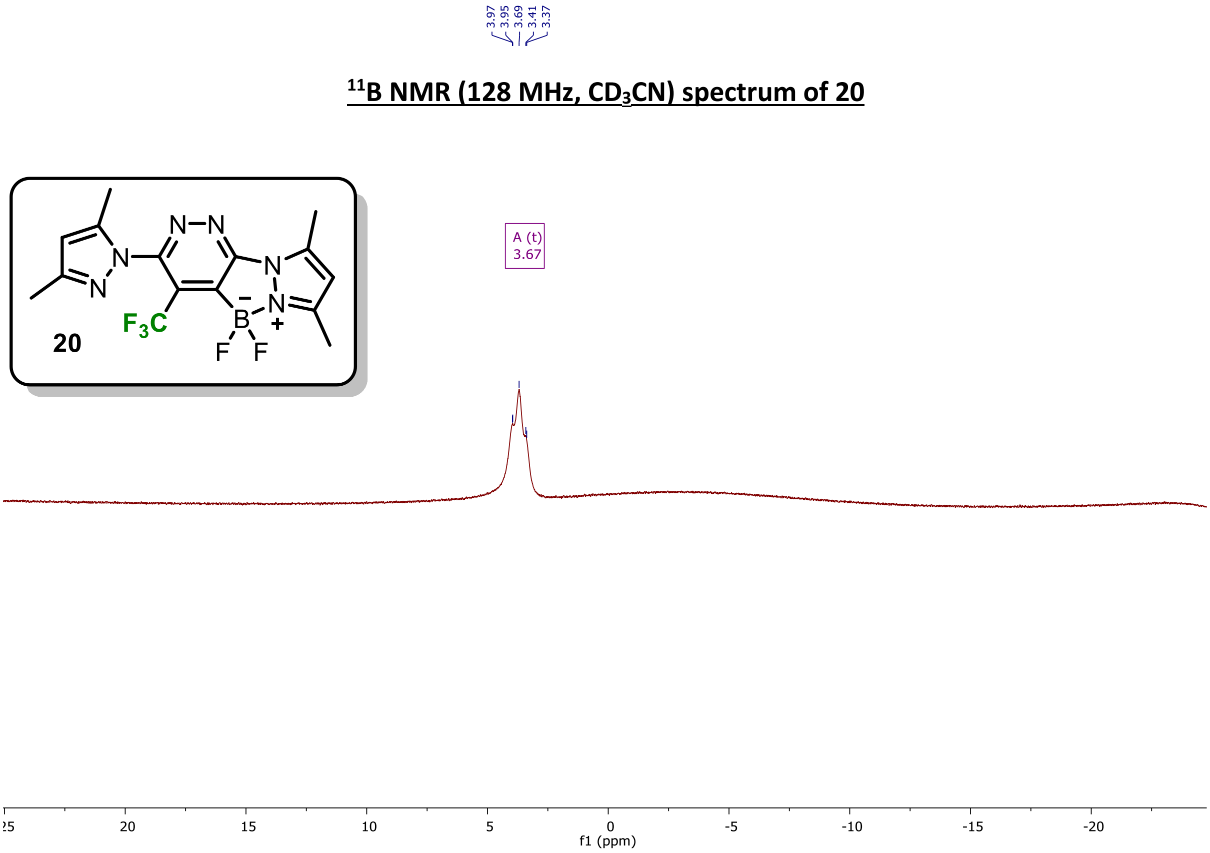

^{1}H NMR (400 MHz, $CDCl_3$) spectrum of 21

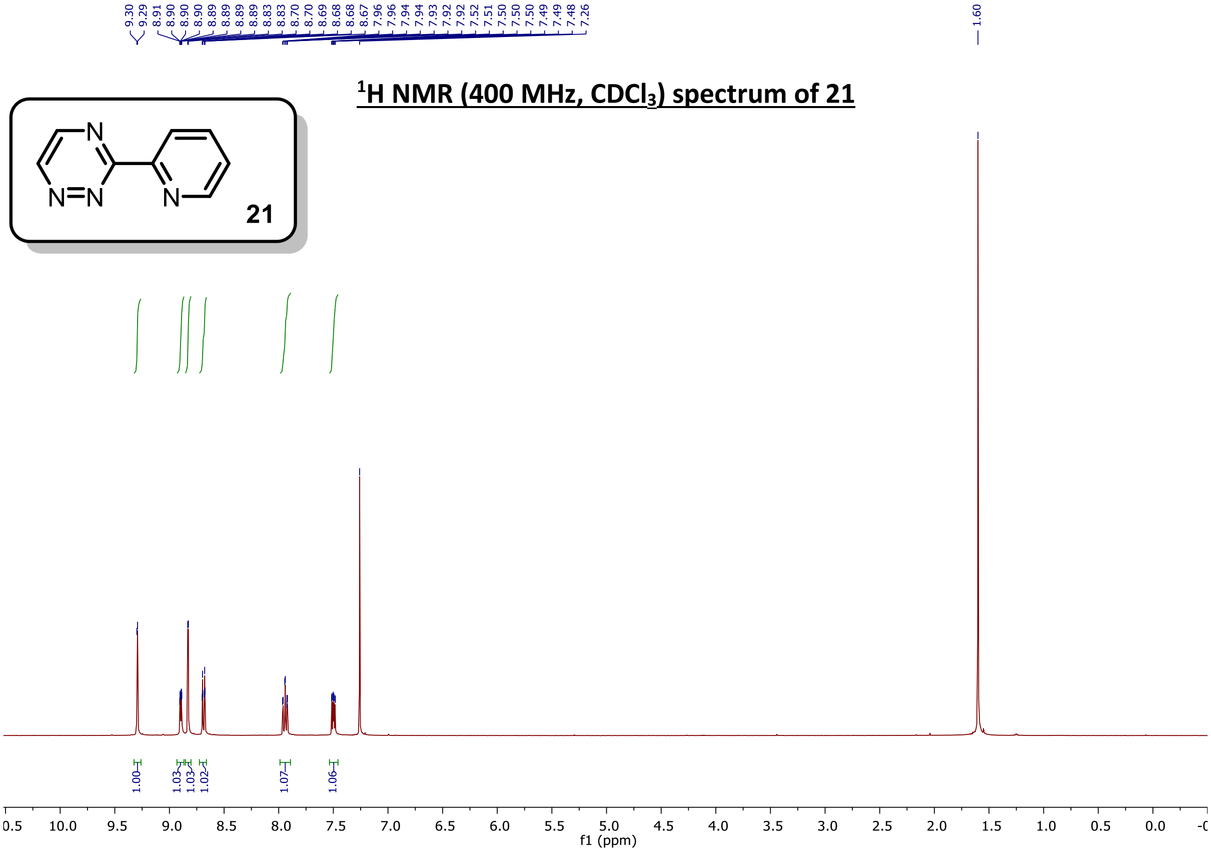

^{1}H NMR (400 MHz, acetone-d^{6}) spectrum of 22

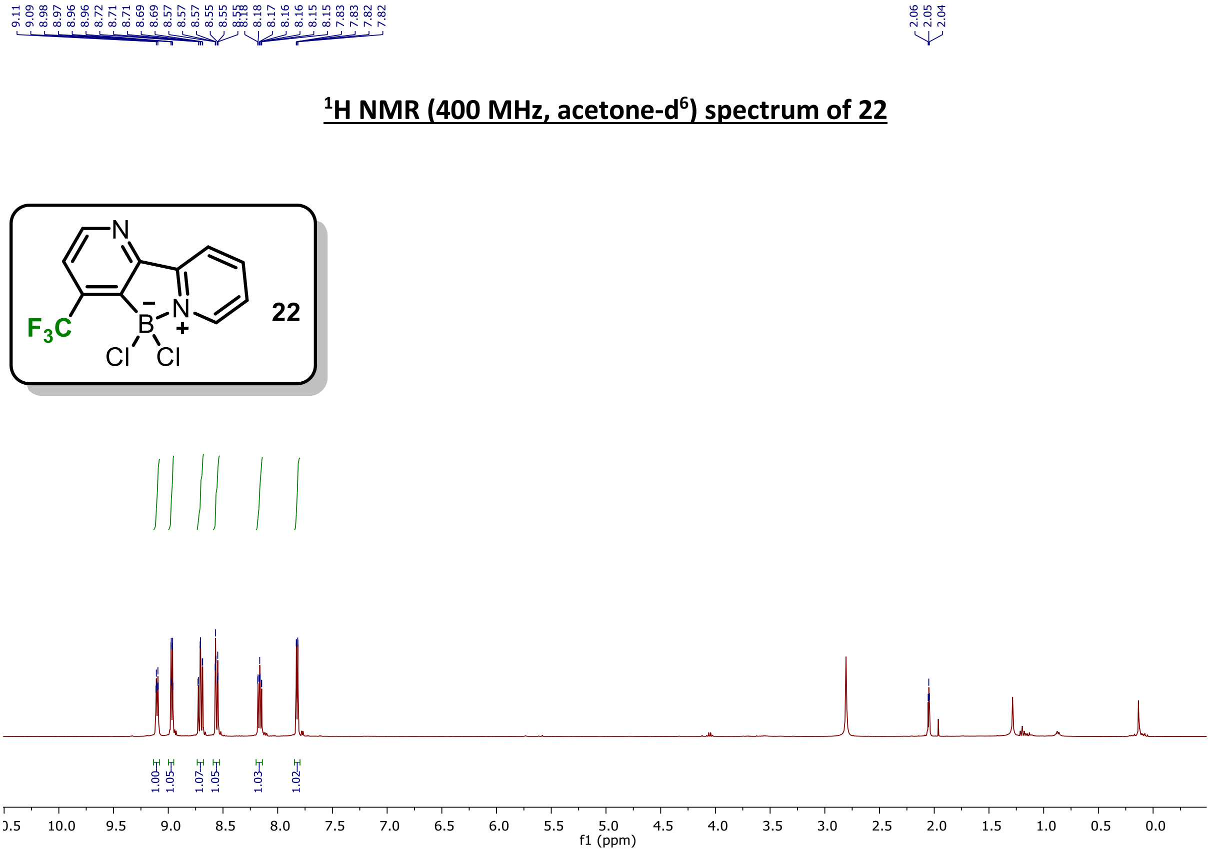

$^{13}C\{^{1}H\}$ NMR (101 MHz, acetone-d^{6}) spectrum of 22

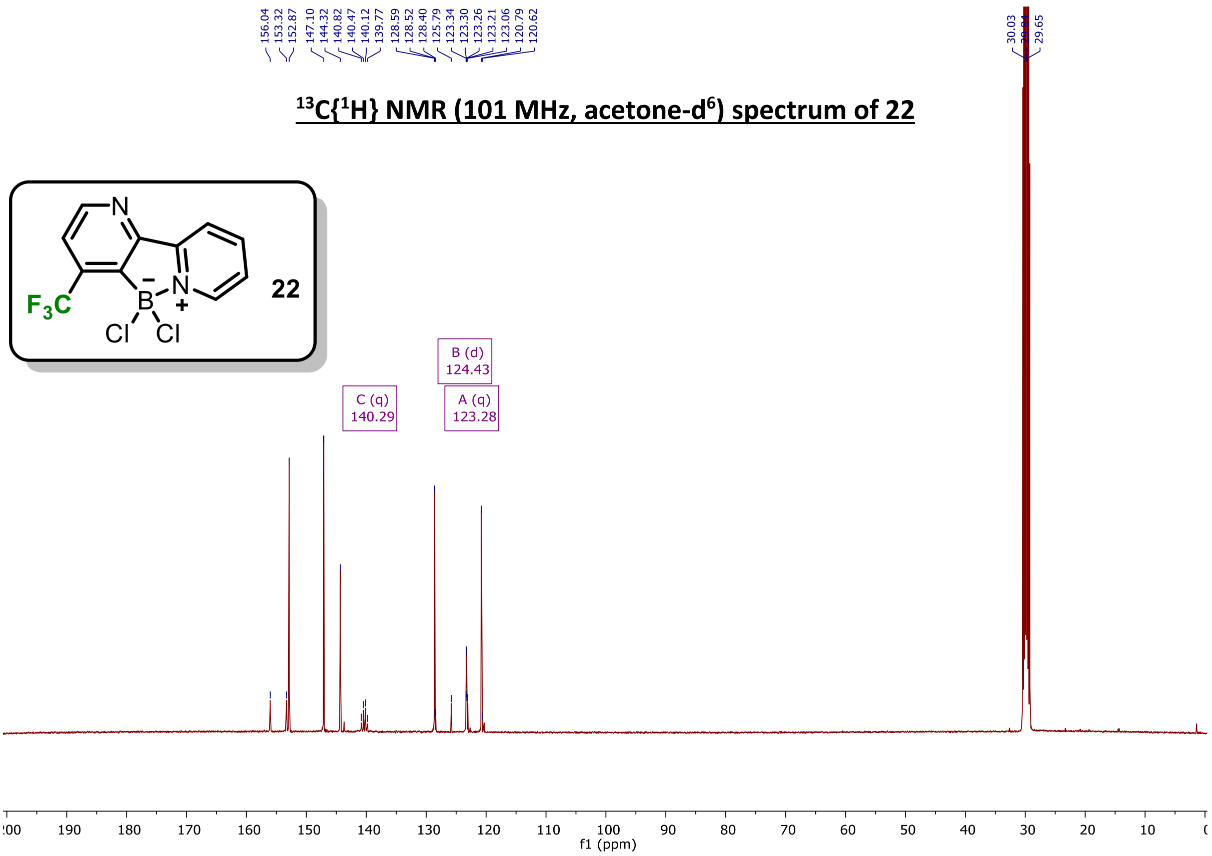

^{19}F NMR (376 MHz, acetone-d^{6}) spectrum of 22

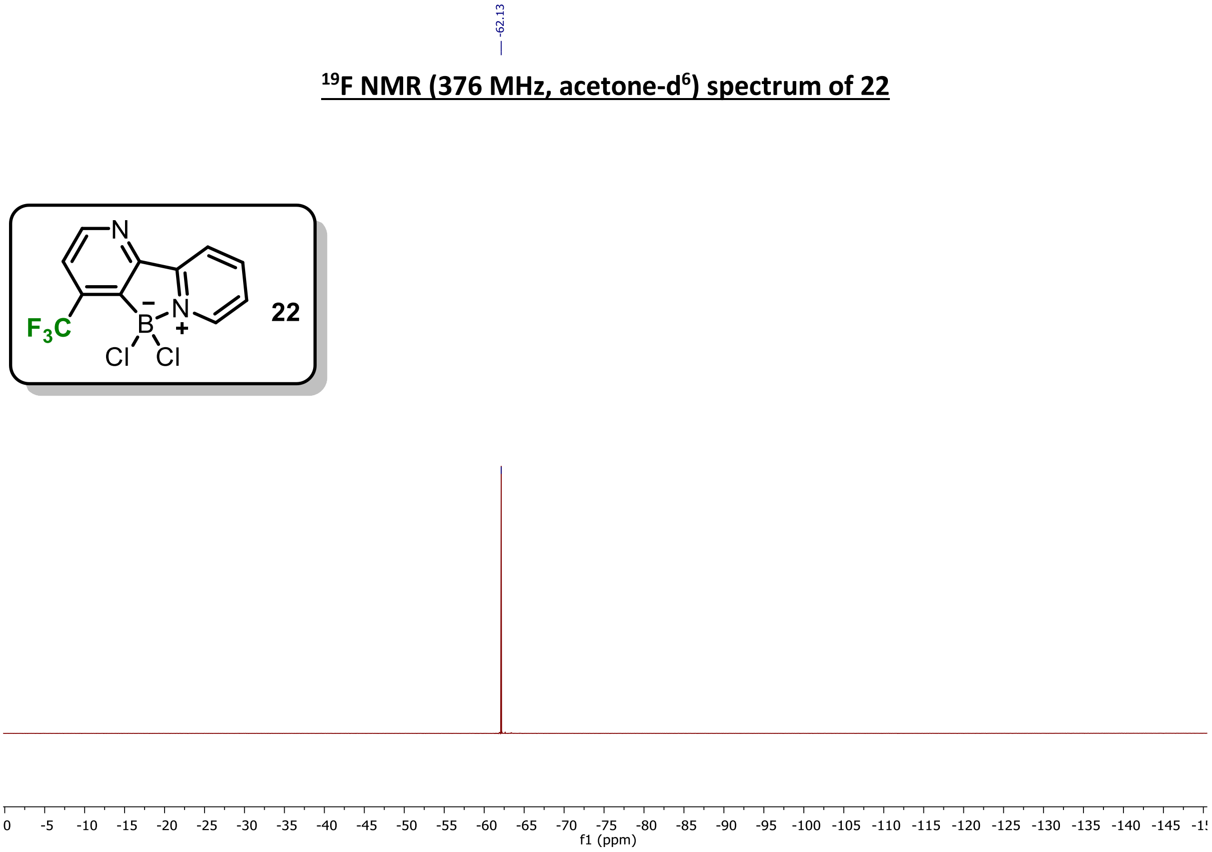

^{11}B NMR (128 MHz, acetone-d^6) spectrum of 22

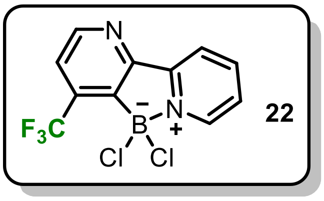

22

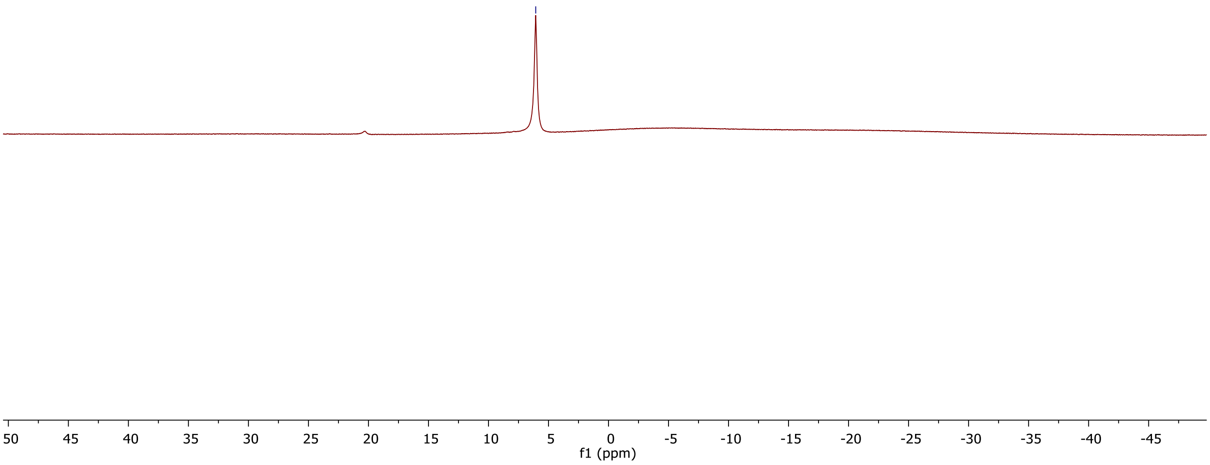

^{1}H NMR (400 MHz, $CDCl_3$) spectrum of 23

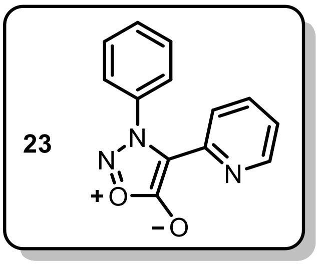

23

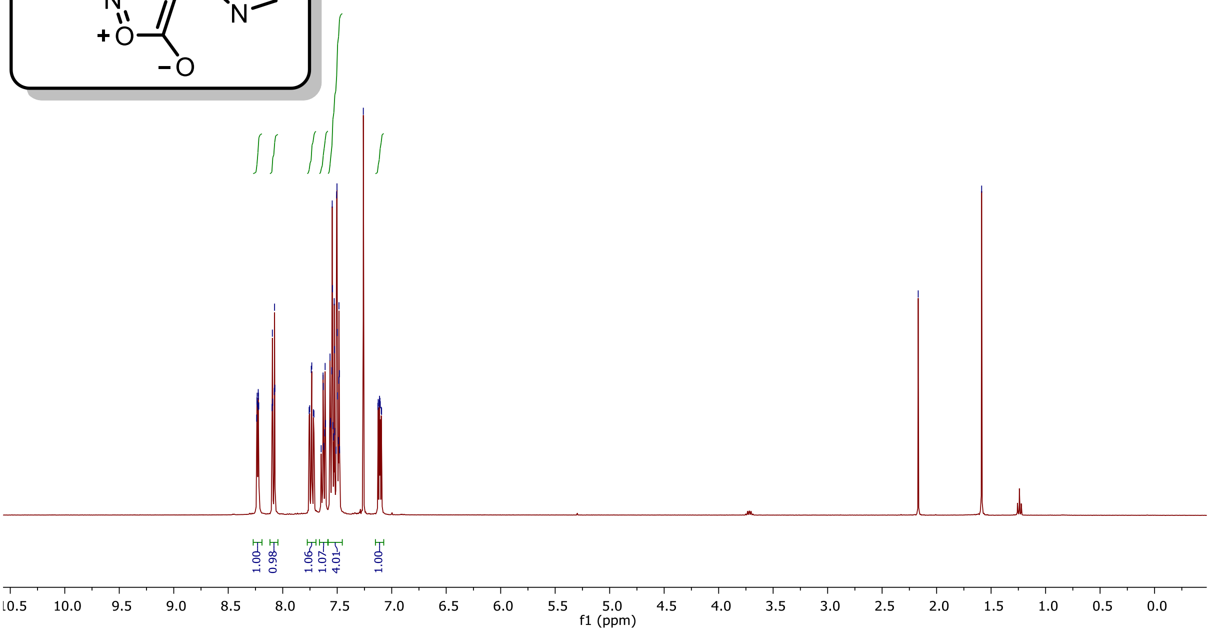

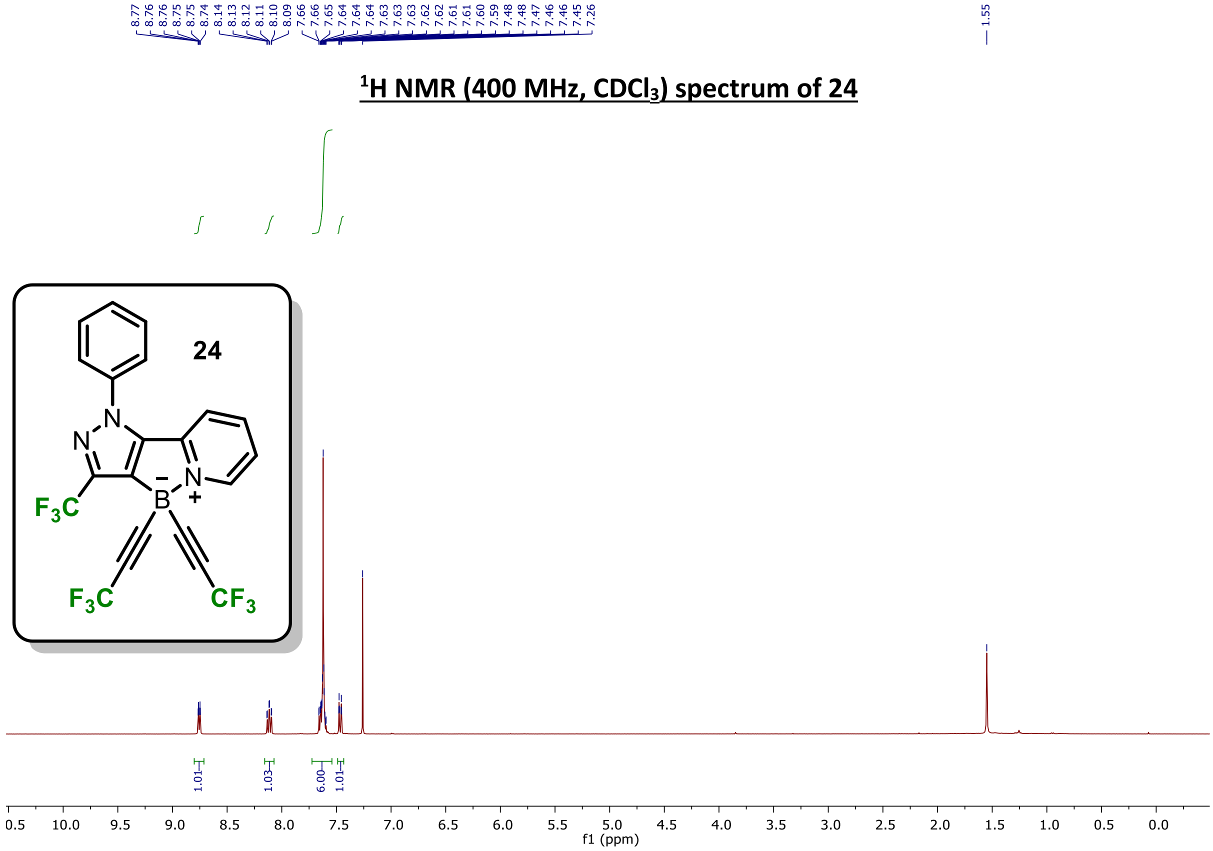
^{1}H NMR (400 MHz, $CDCl_3$) spectrum of 24
8.77 8.76 8.76 8.75 8.75 8.74 8.14 8.13 8.12 8.11 8.10 8.09 7.66 7.66 7.65 7.64 7.64 7.64 7.63 7.63 7.62 7.62 7.61 7.61 7.60 7.59 7.48 7.48 7.47 7.46 7.46 7.45 7.26
1.55
24
F_3C
CF_3
1.01 1.03 6.00 1.01
f1 (ppm)

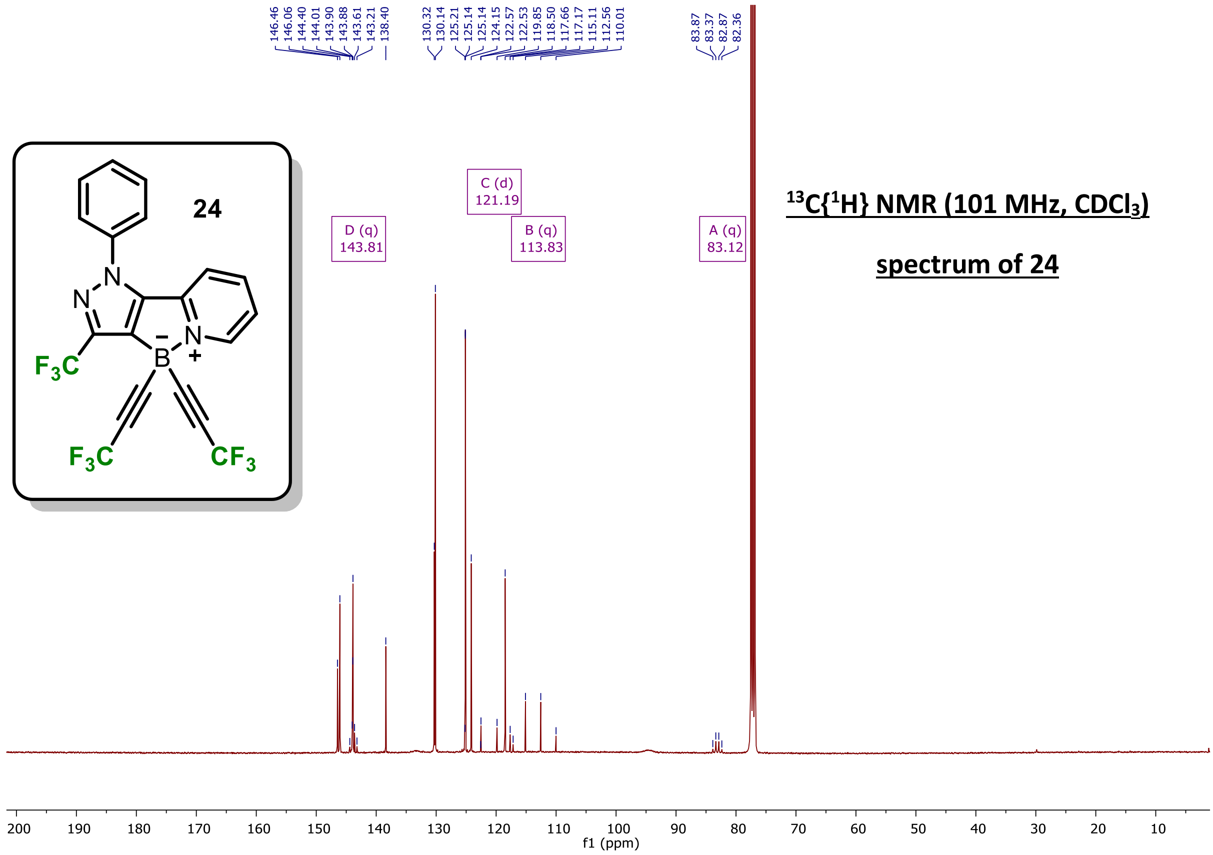
$^{13}C\{^1H\}$ NMR (101 MHz, $CDCl_3$) spectrum of 24
146.46 146.06 144.40 144.01 143.90 143.88 143.61 143.21 138.40
130.32 130.14 125.21 125.14 124.15 122.57 122.53 119.85 118.50 117.66 117.17 115.11 112.56 110.01
83.87 83.37 82.87 82.36
D (q) 143.81
C (d) 121.19
B (q) 113.83
A (q) 83.12
24
F_3C
CF_3
f1 (ppm)

^{19}F NMR (376 MHz, $CDCl_3$) spectrum of 24

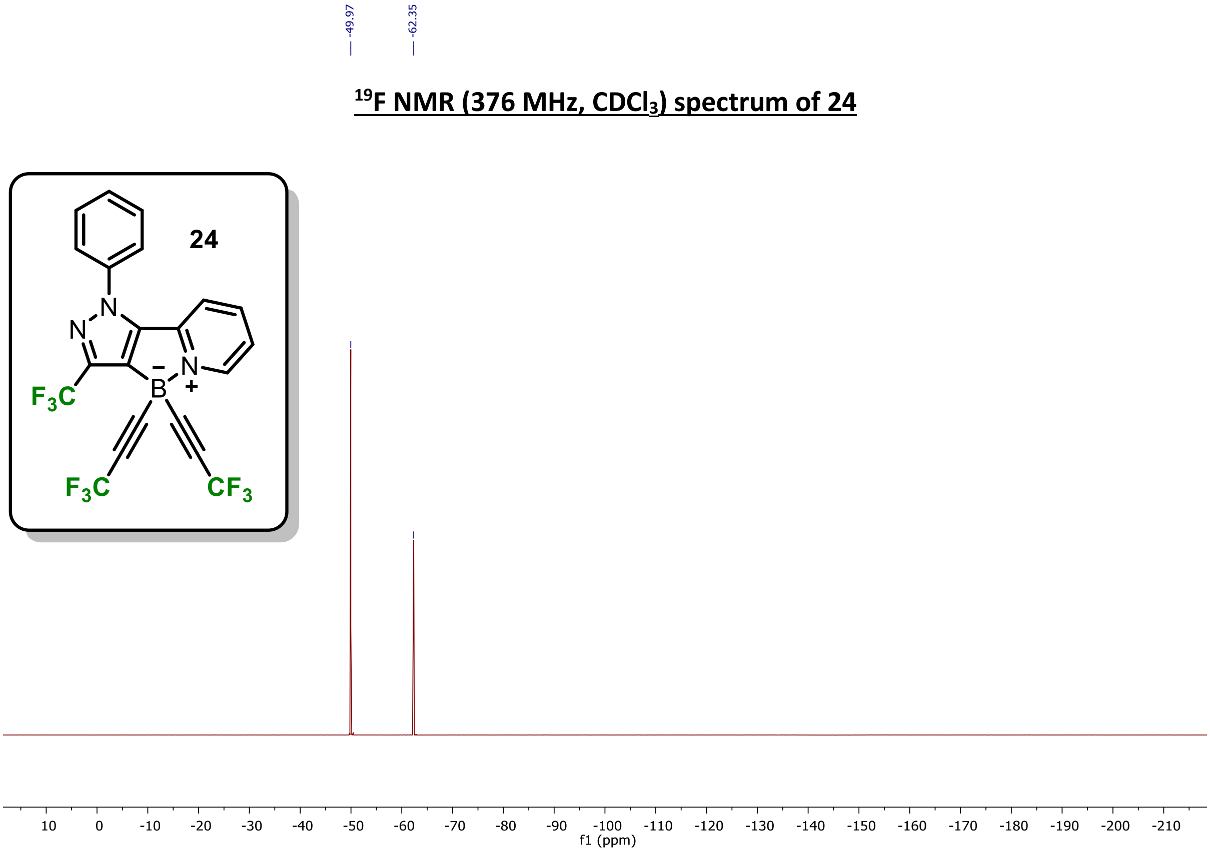

^{11}B NMR (128 MHz, $CDCl_3$) spectrum of 24

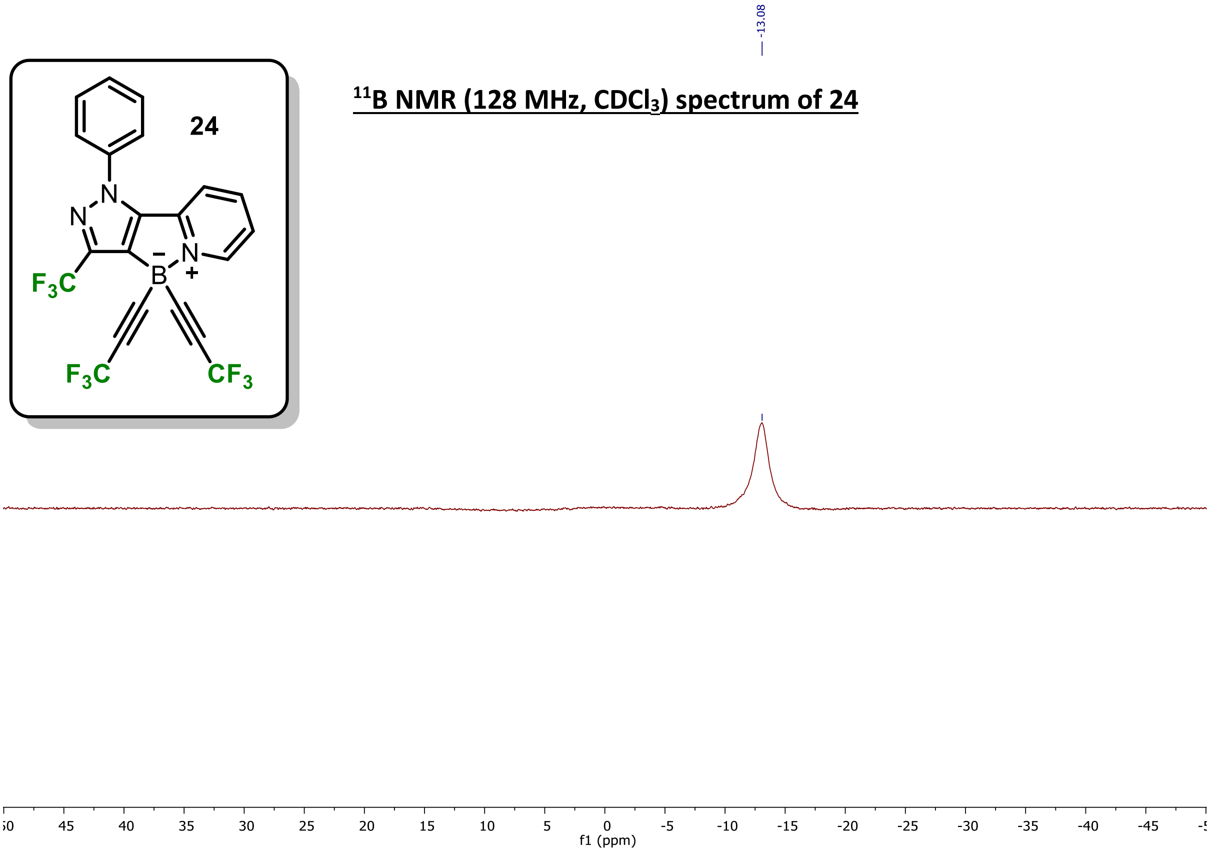

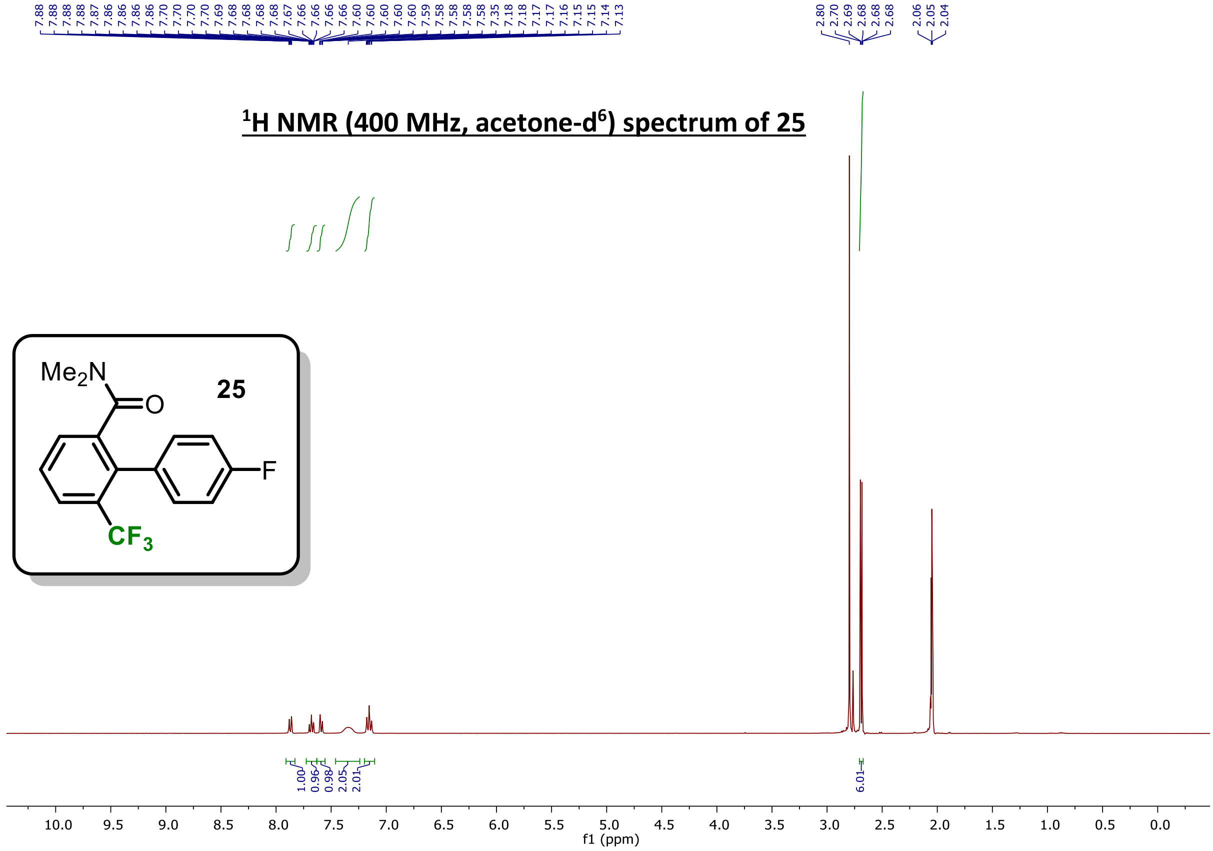
7.88
7.88
7.88
7.88
7.87
7.86
7.86
7.86
7.86
7.70
7.70
7.70
7.70
7.69
7.68
7.68
7.68
7.68
7.67
7.66
7.66
7.66
7.66
7.60
7.60
7.60
7.60
7.59
7.58
7.58
7.58
7.58
7.35
7.18
7.18
7.17
7.16
7.15
7.14
7.13
2.80
2.70
2.69
2.68
2.68
2.68
2.06
2.05
2.04
¹H NMR (400 MHz, acetone-d⁶) spectrum of 25
Me2N
O
F
CF3
25
1.00
0.96
0.98
2.05
2.01
6.01
10.0
9.5
9.0
8.5
8.0
7.5
7.0
6.5
6.0
5.5
5.0
4.5
4.0
3.5
3.0
2.5
2.0
1.5
1.0
0.5
0.0
f1 (ppm)

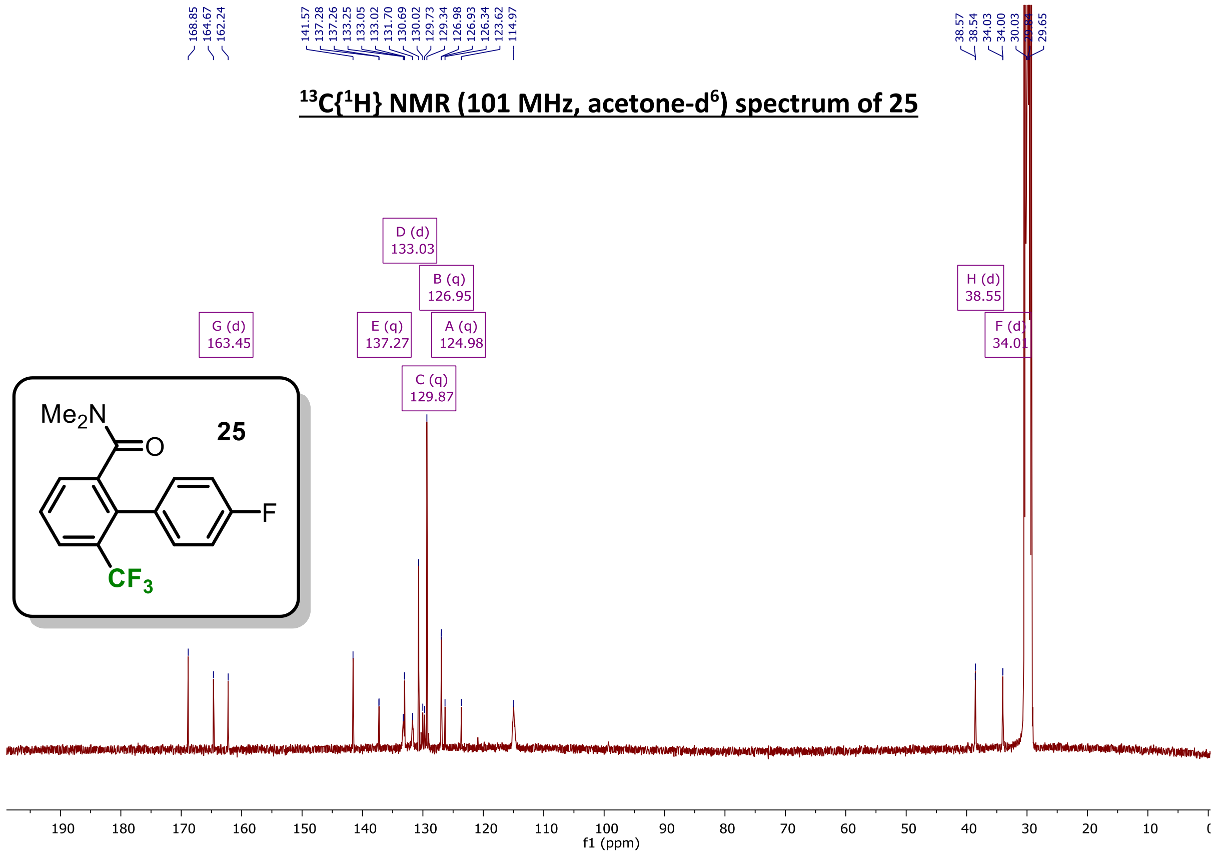
168.85
164.67
162.24
141.57
137.28
137.26
133.25
133.05
133.02
131.70
130.69
130.02
129.73
129.34
126.98
126.93
126.34
123.62
114.97
38.57
38.54
34.03
34.00
30.03
29.65
¹³C{¹H} NMR (101 MHz, acetone-d⁶) spectrum of 25
D (d)
133.03
B (q)
126.95
E (q)
137.27
A (q)
124.98
G (d)
163.45
C (q)
129.87
H (d)
38.55
Me2N
O
F
CF3
25
190
180
170
160
150
140
130
120
110
100
90
80
70
60
50
40
30
20
10
f1 (ppm)

^{19}F NMR (376 MHz, acetone-d^{6}) spectrum of 25

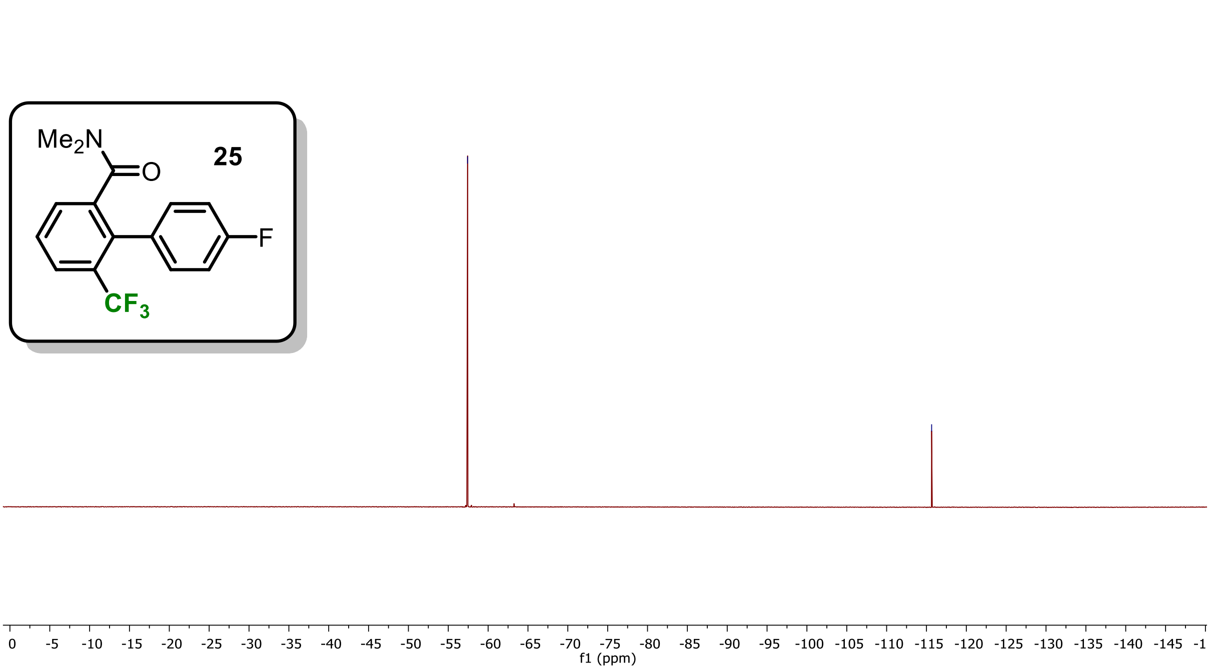

^{1}H NMR (400 MHz, acetone-d^{6}) spectrum of 26

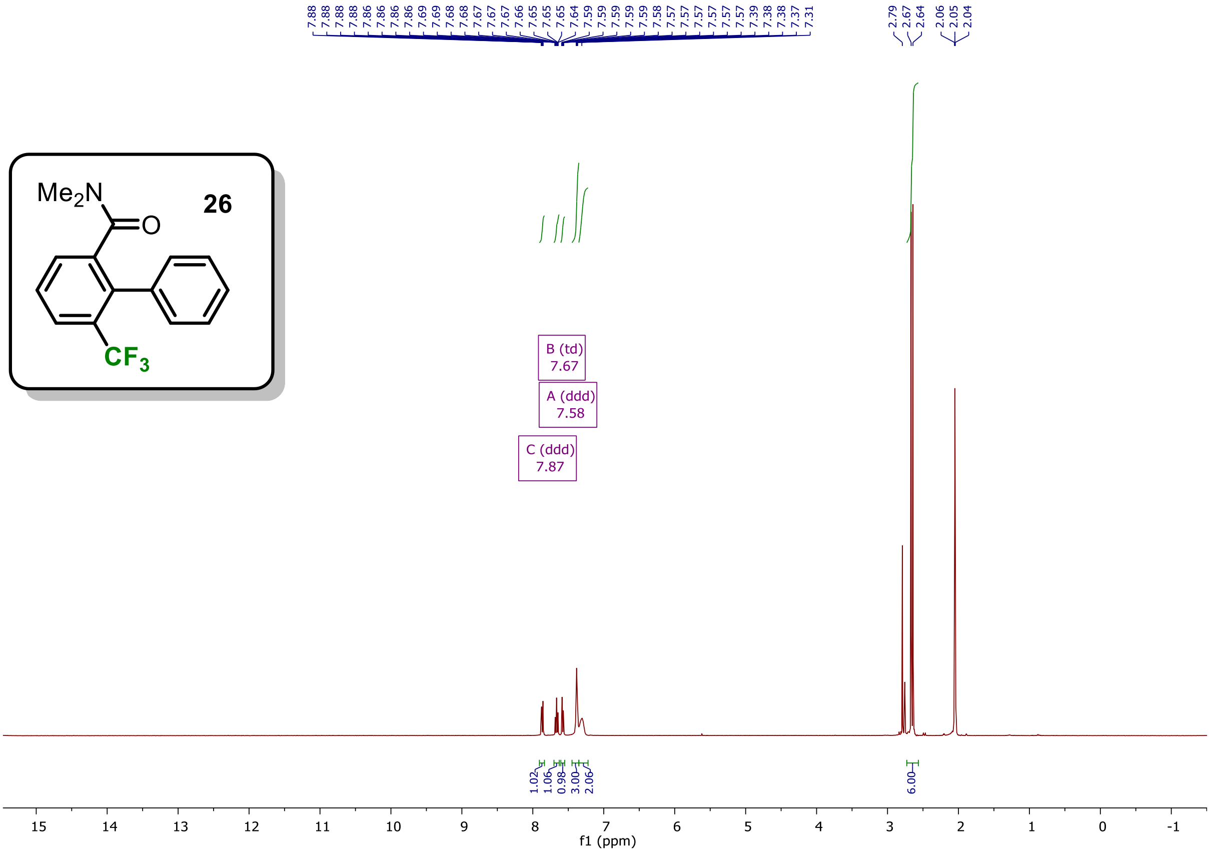

$^{13}C\{^{1}H\}$ NMR (101 MHz, acetone-d^{6}) spectrum of 26

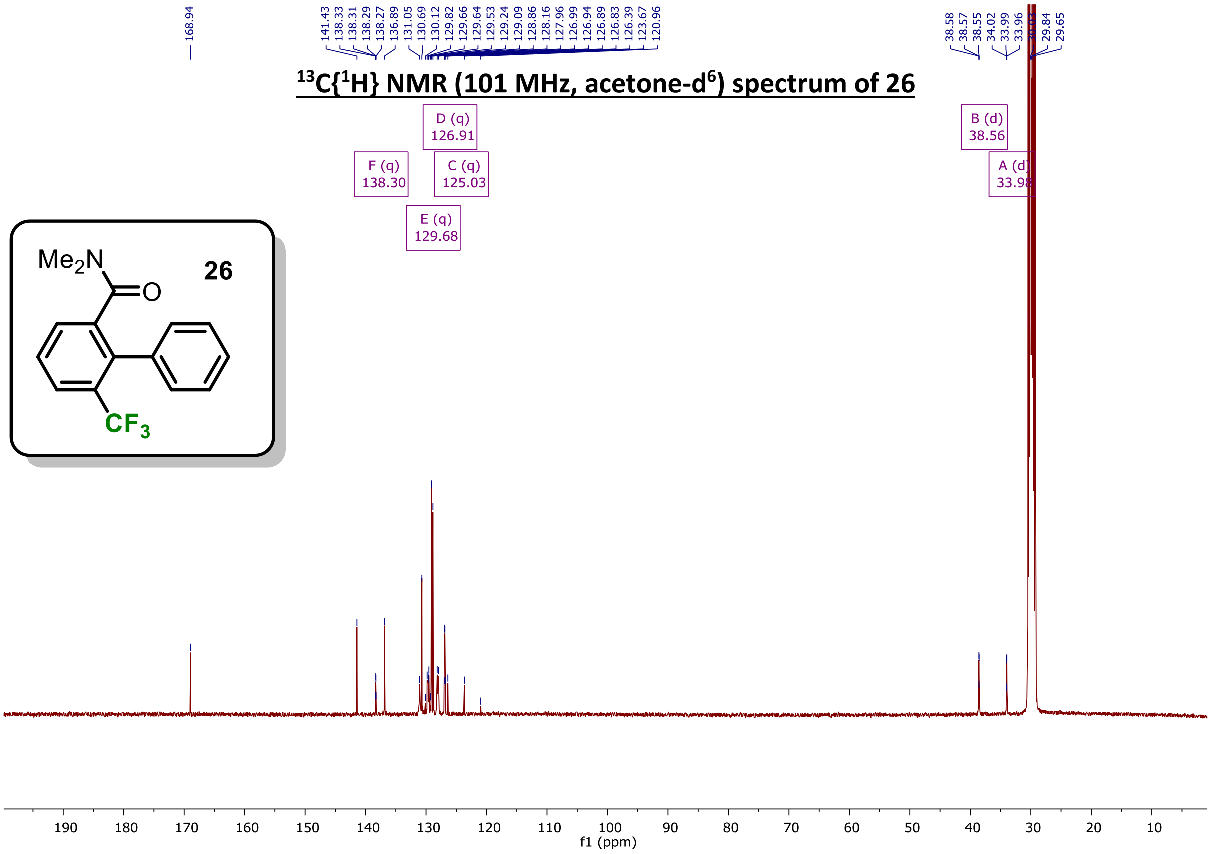

^{19}F NMR (376 MHz, acetone-d^{6}) spectrum of 26

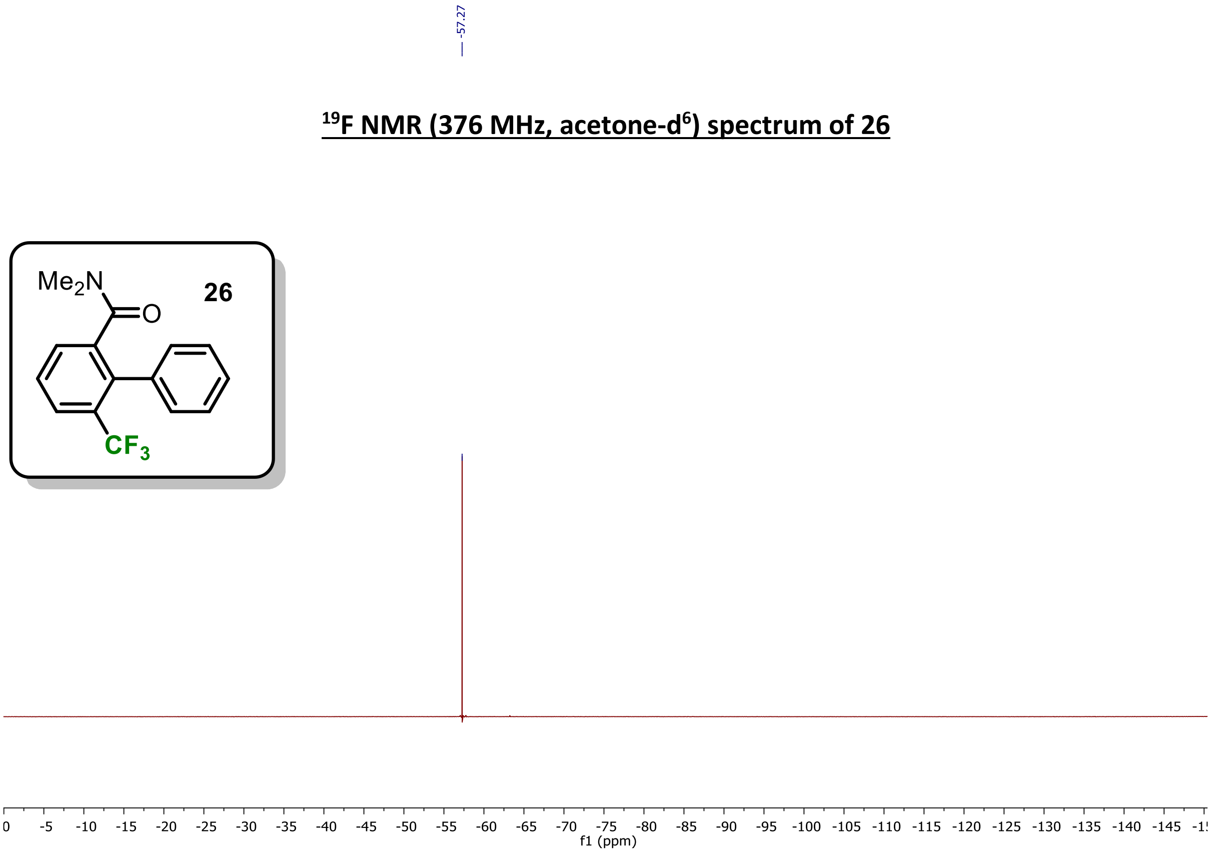

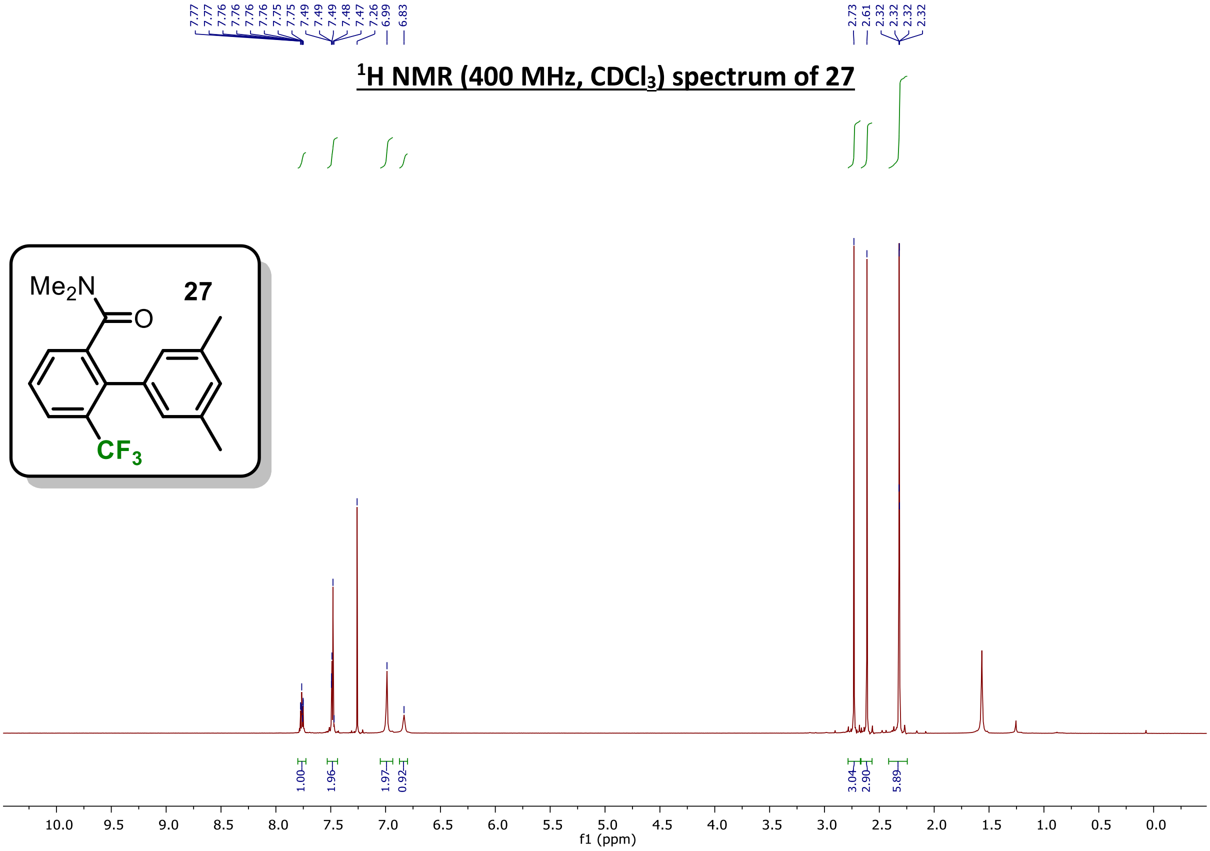

^{1}H NMR (400 MHz, $CDCl_3$) spectrum of 27

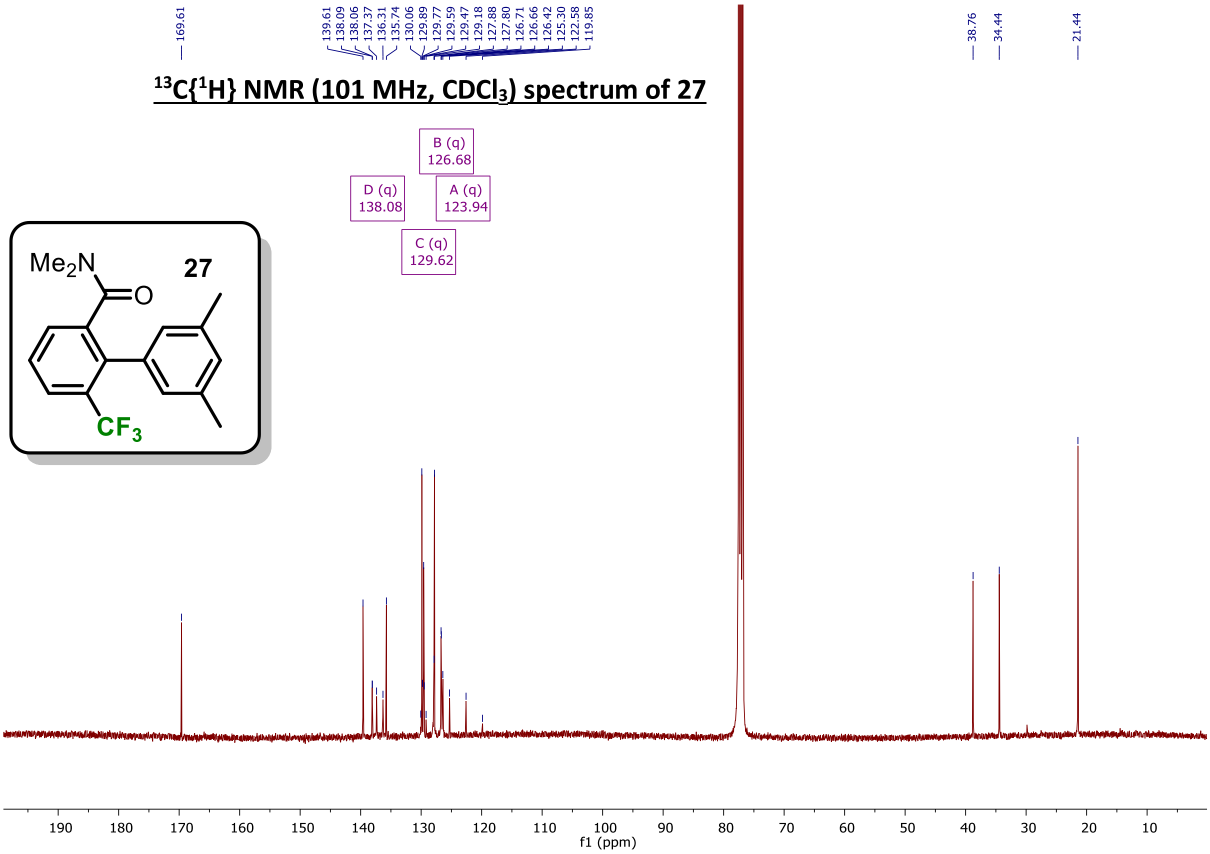

$^{13}C\{^{1}H\}$ NMR (101 MHz, $CDCl_3$) spectrum of 27

^{19}F NMR (376 MHz, $CDCl_3$) spectrum of 27

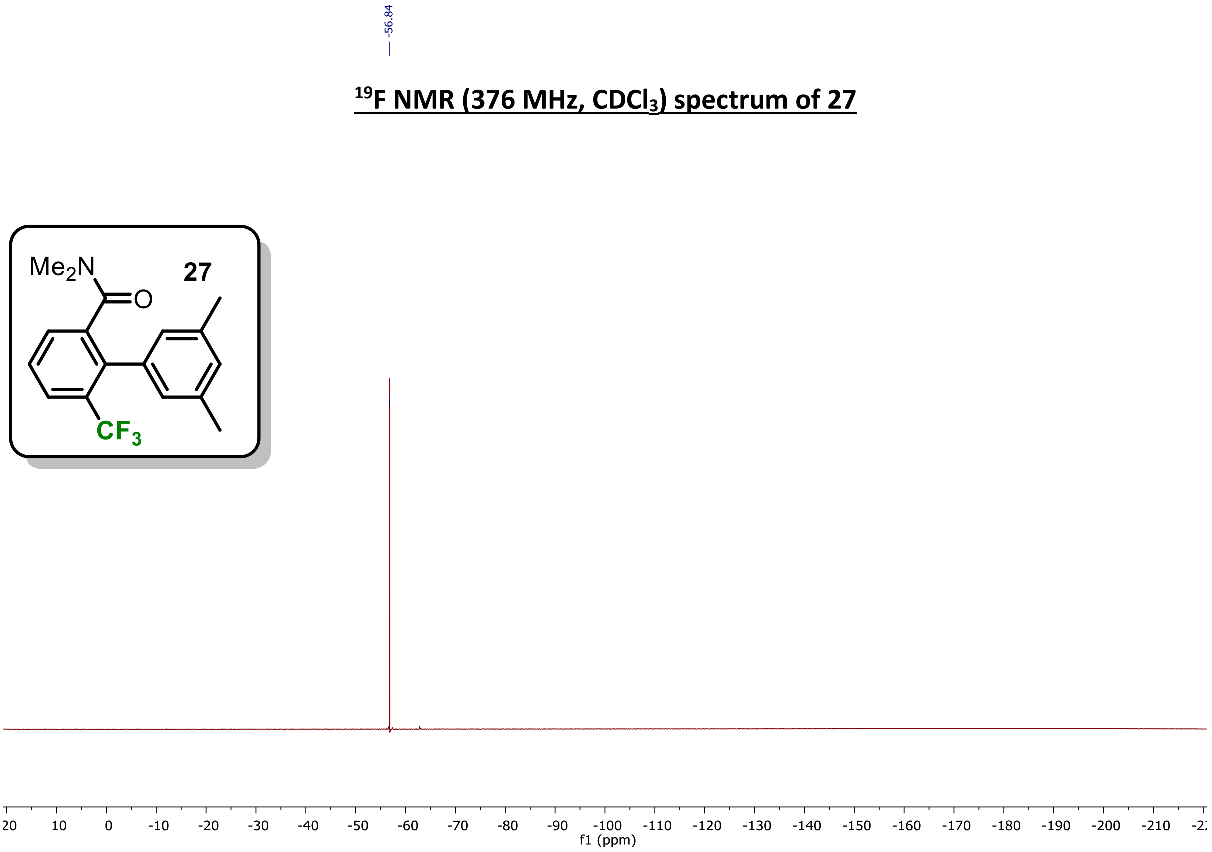

^{1}H NMR (400 MHz, acetone-d^{6}) spectrum of 28

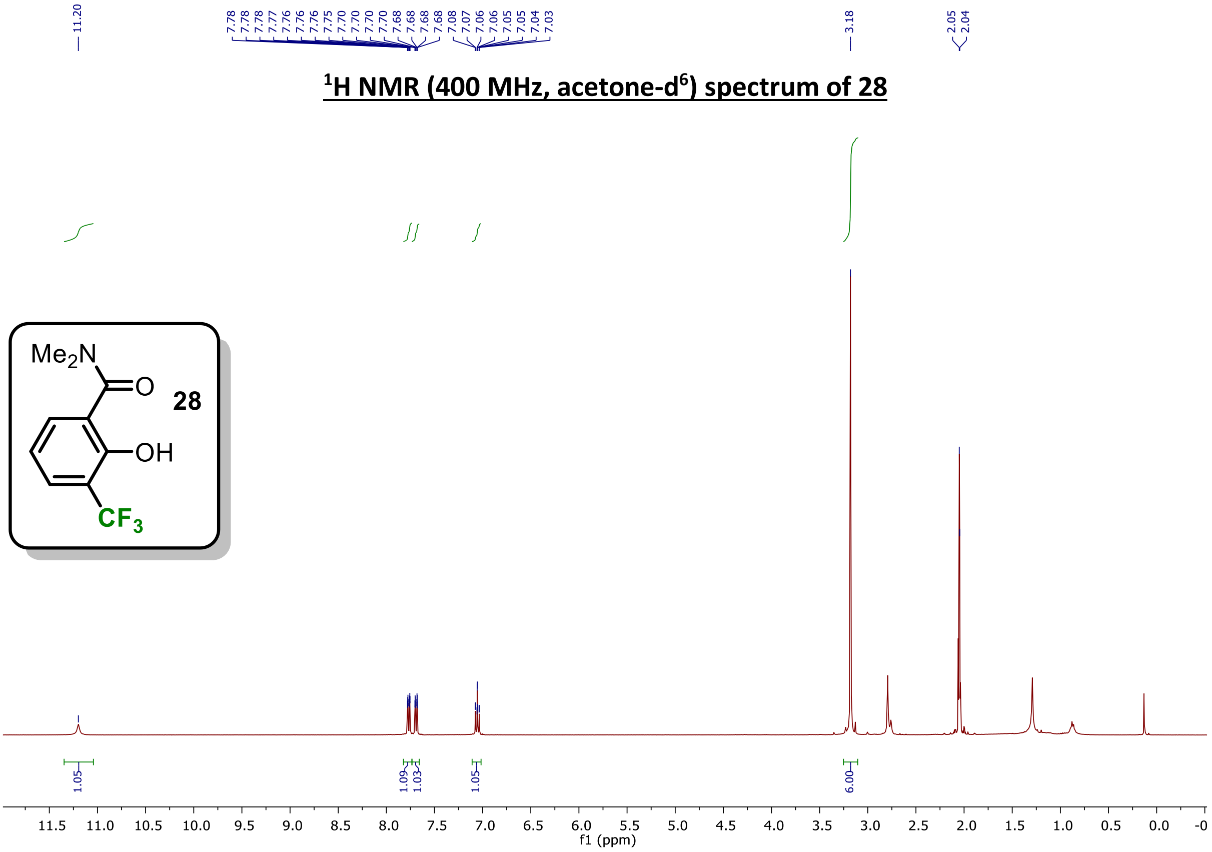

$^{13}C\{^{1}H\}$ NMR (101 MHz, acetone-d^{6}) spectrum of 28

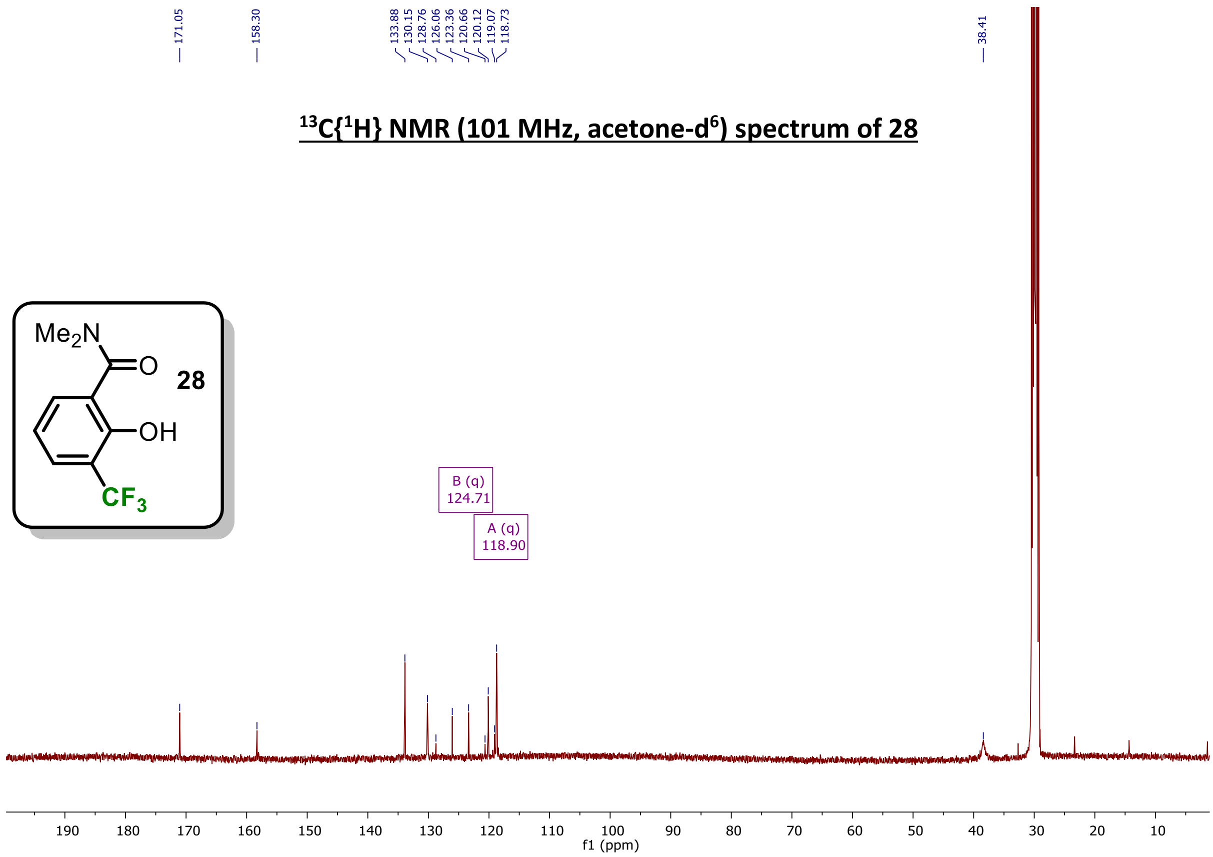

^{19}F NMR (376 MHz, acetone-d^{6}) spectrum of 28

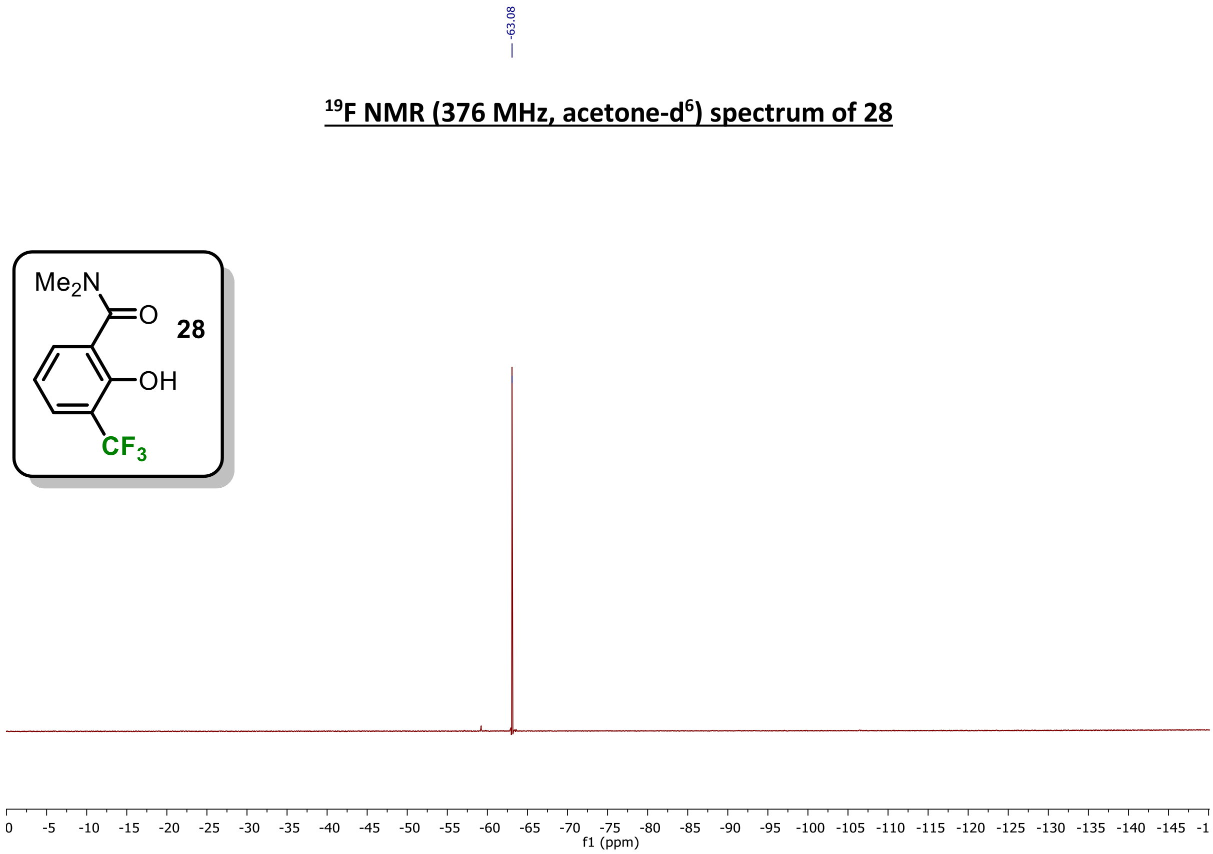

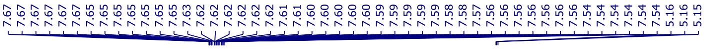

^{1}H NMR (400 MHz, $CDCl_3$) spectrum of 29

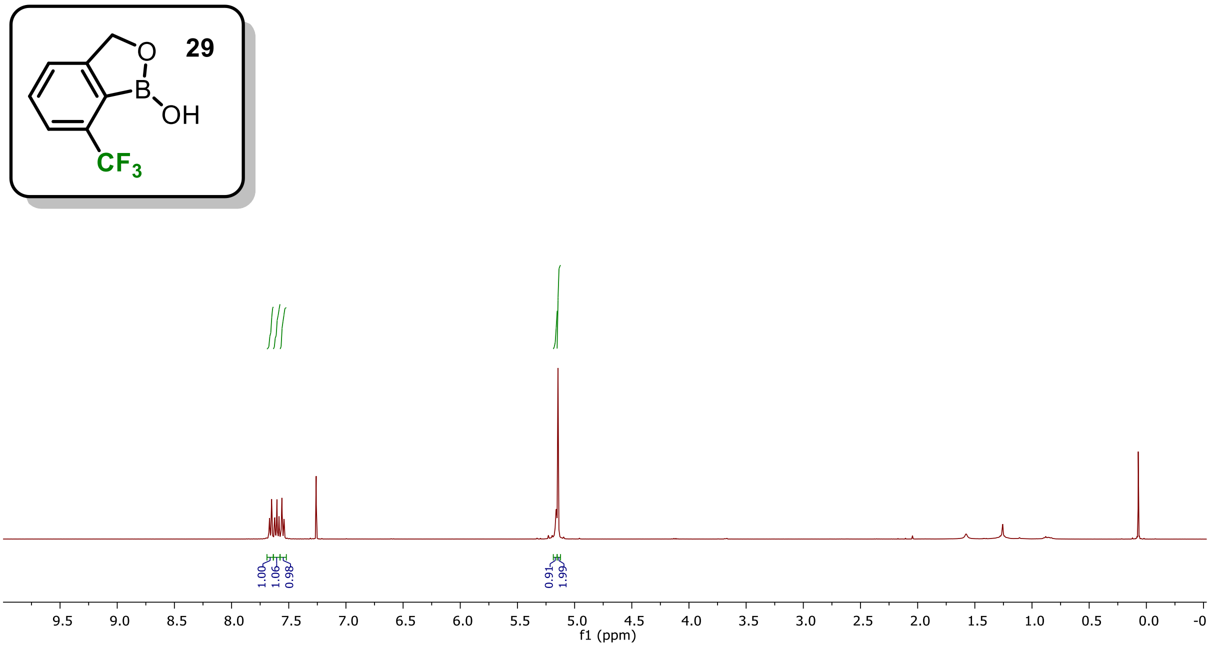

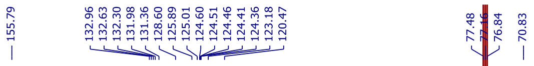

^{13}C{^{1}H} NMR (101 MHz, $CDCl_3$) spectrum of 29

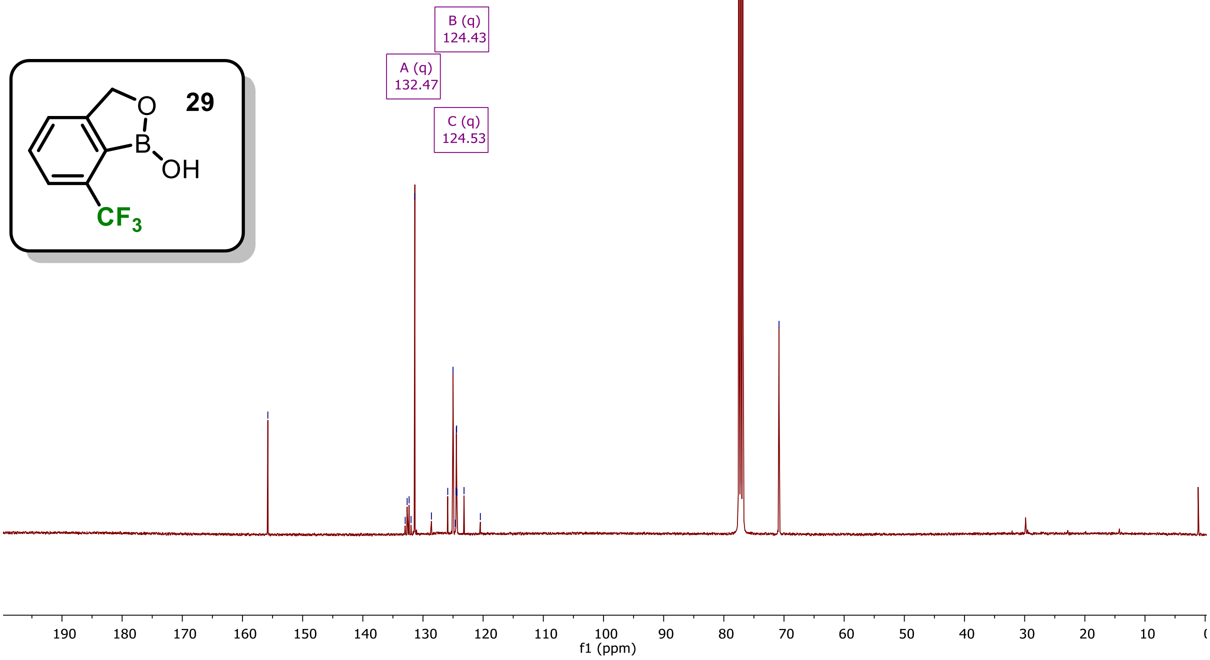

^{19}F NMR (376 MHz, $CDCl_3$) spectrum of 29

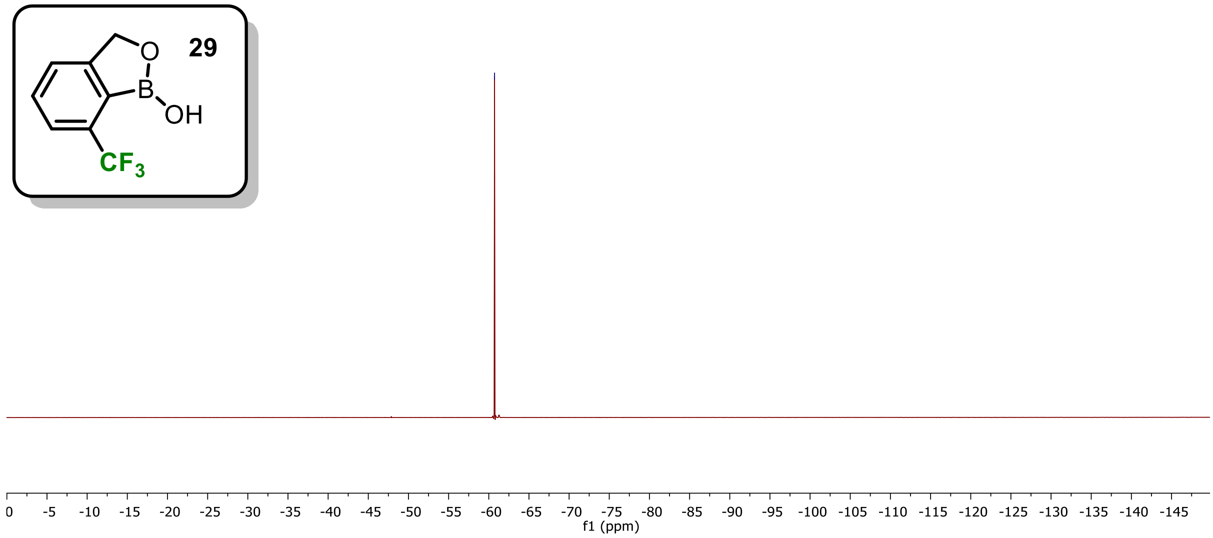

^{11}B NMR (128 MHz, $CDCl_3$) spectrum of 29

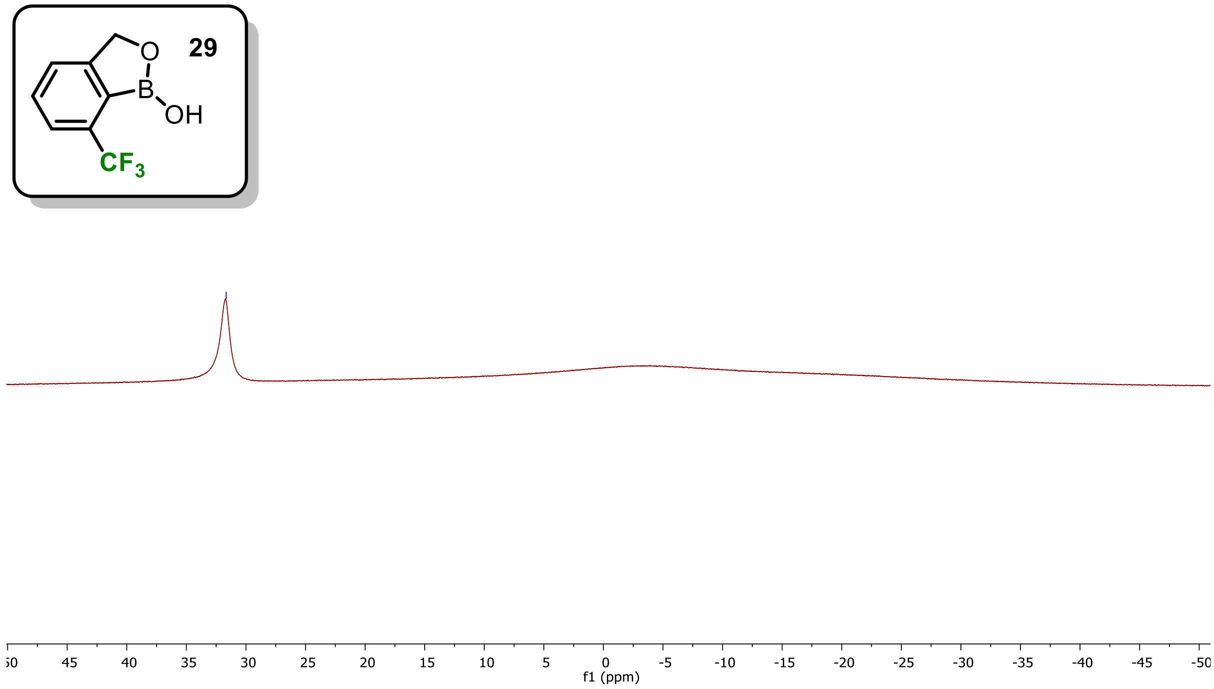

References

[1] D. D. Perrin, W. L. F. Armarego, *Purification of Laboratory Materials*, Pergamon, Oxford, **1988**.

[2] P. V. Ramachandran, W. Mitsuhashi, *J. Fluor. Chem.* **2016**, *190*, 7–11.

[3] V. V. Bardin, N. Y. Adonin, H. J. Frohn, *Organomet.* **2005**, *24*, 5311–5317.

[4] J. D. Kirkham, R. J. Butlin, J. P. A. Harrity, *Angew. Chem. Int. Ed.* **2012**, *51*, 6402–6405.

[5] N. Metanis, E. Keinan, P. E. Dawson, *J. Am. Chem. Soc.* **2005**, *127*, 5862–5868.

[6] D. F. P. Crépin, J. P. A. Harrity, J. Jiang, A. J. H. M. Meijer, A.-C. M. A. Nassoy, P. Raubo, *J. Am. Chem. Soc.* **2014**, *136*, 8642–8653.

[7] M. D. Coburn, G. A. Buntain, B. W. Harris, M. A. Hiskey, K.-Y. Lee, D. G. Ott, *J. Heterocycl. Chem.* **1991**, *28*, 2049–2050.

[8] J. Sauer, D. K. Heldmann, G. R. Pabst, *Eur. J. Org. Chem.* **1999**, 313–321.

[9] A. W. Brown, J. Comas-Barceló, J. P. A. Harrity, *Chem. Eur. J.* **2017**, *23*, 5228–5231.

X-ray crystallographic analysis for compound 3a

ORTEP of 3a, thermal ellipsoids are shown at 50% probability:

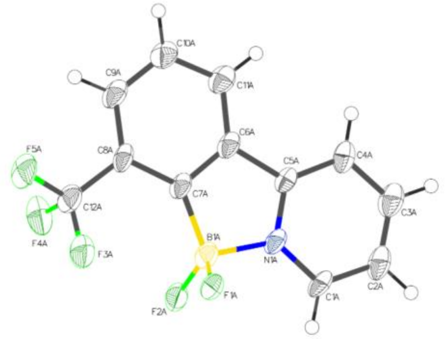

Table S1 Crystal data and structure refinement for 3a.

Identification code	OJH376k_0m
Empirical formula	$C_{12}H_7BF_5N$
Formula weight	271.00
Temperature/K	100
Crystal system	triclinic
Space group	P-1
a/Å	7.934(5)
b/Å	10.500(7)
c/Å	13.898(9)
α/°	70.75(3)
β/°	89.77(3)
γ/°	89.88(3)
Volume/Å^3	1093.1(13)
Z	4
ρ_{calc}g/cm^3	1.647
μ/mm^{-1}	0.156
F(000)	544.0
Crystal size/mm^3	0.416 × 0.32 × 0.223
Radiation	MoKα (λ = 0.71073)
2Θ range for data collection/°	3.104 to 55.47
Index ranges	$-10 \le h \le 10$, $-10 \le k \le 13$, $-15 \le l \le 18$
Reflections collected	13108
Independent reflections	4964 [R_{int} = 0.2141, R_{sigma} = 0.3625]
Data/restraints/parameters	4964/330/343
Goodness-of-fit on F^2	1.012
Final R indexes [I>=2σ (I)]	R_1 = 0.1296, wR_2 = 0.3321
Final R indexes [all data]	R_1 = 0.3174, wR_2 = 0.4144
Largest diff. peak/hole / e Å^{-3}	0.62/-0.66

Experimental

Single crystals of $C_{12}H_7BF_5N$ (3a) were grown from a saturated solution in CH_2Cl_2. A suitable crystal was selected and [MiTiGen microloop in fomblyn oil] on a 'Bruker APEX-II CCD' diffractometer. The crystal was kept at 100 K during data collection. Using Olex2 [1], the structure was solved with the XT [2] structure solution program using Intrinsic Phasing and refined with the XL [3] refinement package using Least Squares minimisation.

1. Dolomanov, O.V., Bourhis, L.J., Gildea, R.J, Howard, J.A.K. & Puschmann, H. (2009), J. Appl. Cryst. 42, 339-341.
2. Sheldrick, G.M. (2015). Acta Cryst. A71, 3-8.
3. Sheldrick, G.M. (2008). Acta Cryst. A64, 112-122.

Crystal structure determination of 3a

Crystal Data for $C_{12}H_7BF_5N$ (M =271.00 g/mol): triclinic, space group P-1 (no. 2), a = 7.934(5) Å, b = 10.500(7) Å, c = 13.898(9) Å, α = 70.75(3)°, β = 89.77(3)°, γ = 89.88(3)°, V = 1093.1(13) Å^3, Z = 4, T = 100 K, μ(MoKα) = 0.156 mm^{-1}, $Dcalc$ = 1.647 g/cm^3, 13108 reflections measured (3.104° ≤ 2Θ ≤ 55.47°), 4964 unique (R_{int} = 0.2141, R_{sigma} = 0.3625) which were used in all calculations. The final R_1 was 0.1296 (I > 2σ(I)) and wR_2 was 0.4144 (all data).

Table S2 Fractional Atomic Coordinates (×10^4) and Equivalent Isotropic Displacement Parameters (Å^2×10^3) for OJH376k_0m. U_{eq} is defined as 1/3 of of the trace of the orthogonalised U_{IJ} tensor.

Atom	*x*	*y*	*z*	U(eq)
F1A	8162(7)	4321(5)	2660(4)	34.6(14)
F2A	5696(7)	3208(5)	2444(4)	36.3(15)
F3A	5531(9)	4937(7)	3874(5)	58.1(19)
F4A	2925(8)	4909(6)	3489(5)	52.5(18)
F5A	3918(8)	6550(6)	3901(5)	50.7(18)
N1A	7297(10)	4509(8)	916(6)	28.3(17)
C1A	8212(12)	3583(10)	664(8)	31(2)
C2A	8703(13)	3851(11)	-353(8)	39(2)
C3A	8265(13)	5057(11)	-1084(9)	42(3)
C4A	7329(12)	6004(11)	-799(8)	34(2)
C5A	6859(11)	5687(9)	221(7)	23.8(19)
C6A	5863(12)	6496(10)	711(8)	30(2)
C7A	5657(12)	5801(9)	1745(8)	29(2)
C8A	4685(12)	6456(10)	2285(8)	31(2)
C9A	3953(13)	7699(10)	1817(8)	37(2)
C10A	4222(14)	8379(10)	794(9)	40(2)

C11A	5156(12)	7776(10)	213(9)	35(2)
C12A	4299(15)	5718(11)	3354(8)	39(2)
B1A	6666(16)	4422(12)	2027(9)	33(2)
F1B	9285(7)	-1788(5)	7438(4)	35.7(14)
F2B	6846(7)	-659(5)	7647(4)	32.3(14)
F3B	12056(9)	-97(7)	8496(5)	57.2(19)
F4B	11079(9)	1554(6)	8898(5)	52.7(18)
F5B	9452(8)	-66(7)	8874(5)	55.2(19)
N1B	7700(11)	-469(8)	5908(6)	33.1(19)
C1B	6778(13)	-1401(10)	5646(8)	35(2)
C2B	6334(13)	-1154(11)	4668(8)	37(2)
C3B	6737(13)	60(11)	3928(9)	39(2)
C4B	7661(12)	1009(11)	4205(8)	34(2)
C5B	8150(13)	704(10)	5211(8)	34(2)
C6B	9138(12)	1520(10)	5697(8)	32(2)
C7B	9335(12)	816(10)	6727(8)	29(2)
C8B	10345(13)	1433(10)	7294(8)	37(2)
C9B	11059(13)	2709(10)	6808(8)	35(2)
C10B	10771(13)	3373(11)	5789(8)	38(2)
C11B	9846(13)	2758(10)	5228(9)	36(2)
C12B	10715(14)	729(11)	8358(8)	38(2)
B1B	8339(16)	-612(12)	7027(10)	36(2)

Table S3 Anisotropic Displacement Parameters (Å^2×10^3) for OJH376k_0m. The Anisotropic displacement factor exponent takes the form: -2π^2[h^{2}a*^{2}U$_{11}$+2hka*b*U$_{12}$+…].

Atom	U_{11}	U_{22}	U_{33}	U_{23}	U_{13}	U_{12}
F1A	44(3)	30(3)	34(3)	-17(3)	7(2)	6(3)
F2A	37(3)	33(3)	42(3)	-17(3)	14(2)	-1(2)
F3A	65(4)	70(5)	36(4)	-13(3)	13(3)	21(3)
F4A	59(4)	61(4)	43(4)	-25(3)	20(3)	-18(3)
F5A	71(4)	46(4)	49(4)	-34(3)	17(3)	-1(3)
N1A	28(4)	24(4)	39(4)	-19(3)	7(3)	0(3)
C1A	30(5)	27(5)	45(5)	-25(4)	3(4)	3(4)
C2A	40(6)	45(5)	45(5)	-33(4)	10(4)	-4(4)
C3A	43(6)	45(5)	50(5)	-32(4)	9(4)	-4(4)
C4A	30(5)	42(5)	39(4)	-26(4)	7(4)	-4(4)
C5A	20(4)	25(4)	34(4)	-19(3)	1(3)	-2(3)
C6A	33(5)	26(4)	39(4)	-21(3)	7(3)	-1(3)
C7A	32(5)	24(4)	38(4)	-19(3)	9(3)	-2(3)

C8A	31(5)	28(4)	43(4)	-24(3)	14(3)	-6(3)
C9A	37(6)	29(4)	55(5)	-25(4)	13(4)	-3(4)
C10A	48(6)	24(5)	53(5)	-20(4)	13(4)	-5(4)
C11A	34(5)	31(4)	46(5)	-21(4)	2(4)	-2(4)
C12A	48(5)	32(5)	42(4)	-20(3)	11(3)	2(4)
B1A	40(5)	29(4)	36(4)	-17(3)	8(3)	1(3)
F1B	41(3)	31(3)	38(3)	-16(3)	6(2)	5(2)
F2B	41(3)	30(3)	30(3)	-15(2)	12(2)	-7(2)
F3B	61(4)	66(4)	55(4)	-33(3)	2(3)	17(3)
F4B	79(5)	50(4)	41(4)	-32(3)	3(3)	-2(3)
F5B	58(4)	68(4)	41(4)	-20(3)	7(3)	-18(3)
N1B	41(5)	28(4)	39(4)	-23(3)	10(3)	0(3)
C1B	40(6)	27(5)	49(5)	-28(4)	11(4)	0(4)
C2B	34(6)	42(5)	50(5)	-37(4)	13(4)	5(4)
C3B	35(6)	44(5)	49(5)	-31(4)	5(4)	7(4)
C4B	32(5)	35(5)	42(5)	-22(4)	12(4)	6(4)
C5B	36(5)	31(4)	43(4)	-24(3)	9(3)	3(4)
C6B	37(5)	24(4)	40(4)	-20(3)	7(3)	5(4)
C7B	31(5)	27(4)	37(4)	-20(3)	11(3)	5(3)
C8B	41(5)	36(4)	40(4)	-22(3)	13(3)	-2(4)
C9B	30(5)	34(5)	48(5)	-24(4)	13(4)	-1(4)
C10B	43(6)	30(5)	48(5)	-22(4)	12(4)	2(4)
C11B	41(6)	29(4)	43(5)	-20(4)	10(4)	3(4)
C12B	44(5)	37(5)	39(4)	-23(3)	12(3)	-2(4)
B1B	42(5)	28(4)	41(5)	-16(3)	7(3)	3(4)

Table S4 Bond Lengths for OJH376k_0m.

Atom	Atom	Length/Å	Atom	Atom	Length/Å
F1A	B1A	1.464(14)	F1B	B1B	1.396(14)
F2A	B1A	1.438(12)	F2B	B1B	1.453(14)
F3A	C12A	1.327(13)	F3B	C12B	1.345(12)
F4A	C12A	1.357(12)	F4B	C12B	1.353(11)
F5A	C12A	1.367(12)	F5B	C12B	1.349(11)
N1A	C1A	1.347(12)	N1B	C1B	1.365(12)
N1A	C5A	1.341(12)	N1B	C5B	1.340(12)
N1A	B1A	1.594(14)	N1B	B1B	1.597(15)
C1A	C2A	1.400(14)	C1B	C2B	1.346(14)
C2A	C3A	1.381(15)	C2B	C3B	1.386(15)
C3A	C4A	1.397(14)	C3B	C4B	1.392(14)

C4A	C5A	1.394(13)	C4B	C5B	1.385(14)
C5A	C6A	1.479(13)	C5B	C6B	1.481(14)
C6A	C7A	1.389(14)	C6B	C7B	1.387(14)
C6A	C11A	1.410(14)	C6B	C11B	1.368(13)
C7A	C8A	1.402(13)	C7B	C8B	1.424(14)
C7A	B1A	1.585(15)	C7B	B1B	1.623(15)
C8A	C9A	1.380(14)	C8B	C9B	1.405(14)
C8A	C12A	1.463(14)	C8B	C12B	1.450(15)
C9A	C10A	1.380(15)	C9B	C10B	1.377(14)
C10A	C11A	1.390(15)	C10B	C11B	1.379(14)

Table S5 Bond Angles for OJH376k_0m.

Atom	Atom	Atom	Angle/°	Atom	Atom	Atom	Angle/°
C1A	N1A	B1A	127.1(9)	C1B	N1B	B1B	126.0(9)
C5A	N1A	C1A	121.7(9)	C5B	N1B	C1B	121.2(9)
C5A	N1A	B1A	111.2(8)	C5B	N1B	B1B	112.8(8)
N1A	C1A	C2A	119.2(10)	C2B	C1B	N1B	119.8(10)
C3A	C2A	C1A	120.2(10)	C1B	C2B	C3B	120.8(10)
C2A	C3A	C4A	119.4(11)	C2B	C3B	C4B	118.9(10)
C5A	C4A	C3A	118.3(10)	C5B	C4B	C3B	118.8(10)
N1A	C5A	C4A	121.2(9)	N1B	C5B	C4B	120.4(9)
N1A	C5A	C6A	109.8(8)	N1B	C5B	C6B	110.1(9)
C4A	C5A	C6A	128.9(9)	C4B	C5B	C6B	129.4(10)
C7A	C6A	C5A	110.6(9)	C7B	C6B	C5B	109.9(9)
C7A	C6A	C11A	123.6(9)	C11B	C6B	C5B	127.1(10)
C11A	C6A	C5A	125.8(10)	C11B	C6B	C7B	122.9(9)
C6A	C7A	C8A	115.6(9)	C6B	C7B	C8B	116.8(9)
C6A	C7A	B1A	108.9(8)	C6B	C7B	B1B	110.0(9)
C8A	C7A	B1A	135.5(10)	C8B	C7B	B1B	133.2(10)
C7A	C8A	C12A	117.9(9)	C7B	C8B	C12B	120.6(9)
C9A	C8A	C7A	122.2(10)	C9B	C8B	C7B	119.9(10)
C9A	C8A	C12A	119.6(9)	C9B	C8B	C12B	119.5(9)
C8A	C9A	C10A	120.6(10)	C10B	C9B	C8B	120.3(10)
C9A	C10A	C11A	119.9(11)	C9B	C10B	C11B	120.0(10)
C10A	C11A	C6A	117.9(11)	C6B	C11B	C10B	119.9(11)
F3A	C12A	F4A	105.7(9)	F3B	C12B	F4B	104.5(9)
F3A	C12A	F5A	104.5(9)	F3B	C12B	F5B	104.7(9)
F3A	C12A	C8A	115.3(9)	F3B	C12B	C8B	113.3(9)
F4A	C12A	F5A	103.5(8)	F4B	C12B	C8B	114.0(9)

F4A	C12A	C8A	113.9(9)	F5B	C12B	F4B	105.2(8)
F5A	C12A	C8A	112.8(9)	F5B	C12B	C8B	114.1(9)
F1A	B1A	N1A	107.5(8)	F1B	B1B	F2B	110.0(9)
F1A	B1A	C7A	116.0(8)	F1B	B1B	N1B	110.9(9)
F2A	B1A	F1A	108.3(9)	F1B	B1B	C7B	117.4(9)
F2A	B1A	N1A	108.4(8)	F2B	B1B	N1B	106.9(9)
F2A	B1A	C7A	116.4(9)	F2B	B1B	C7B	113.4(9)
C7A	B1A	N1A	99.3(8)	N1B	B1B	C7B	97.2(8)

Table S6 Hydrogen Atom Coordinates (Å×10^4) and Isotropic Displacement Parameters (Å^2×10^3) for OJH376k_0m.

**Atom**	***x***	***y***	***z***	**U(eq)**
H1A	8520	2758	1169	37
H2A	9339	3202	-541	47
H3A	8597	5240	-1774	50
H4A	7021	6843	-1287	41
H9A	3258	8091	2203	45
H10A	3770	9258	488	48
H11A	5312	8213	-498	42
H1B	6451	-2220	6153	42
H2B	5738	-1821	4482	44
H3B	6389	242	3242	46
H4B	7950	1851	3713	41
H9B	11744	3116	7185	42
H10B	11209	4254	5472	46
H11B	9701	3194	4516	43

X-ray crystallographic analysis for compound 4a

ORTEP of 4a, thermal ellipsoids are shown at 50% probability:

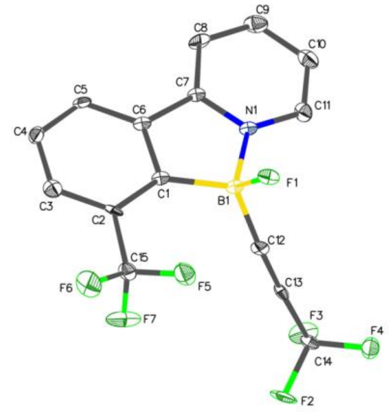

Table S7 Crystal data and structure refinement for 4a

Identification code	OJH399v_0m
Empirical formula	$C_{15}H_7BF_7N$
Formula weight	345.03
Temperature/K	100.01
Crystal system	monoclinic
Space group	$P2_1/n$
a/Å	11.0976(5)
b/Å	8.9576(4)
c/Å	13.9824(6)
α/°	90
β/°	95.627(3)
γ/°	90
Volume/Å^3	1383.26(11)
Z	4
ρ_{calc}g/cm^3	1.657
μ/mm^{-1}	1.461
F(000)	688.0
Crystal size/mm^3	0.204 × 0.158 × 0.02
Radiation	CuKα (λ = 1.54178)
2Θ range for data collection/°	9.724 to 133.54
Index ranges	$-13 \leq h \leq 11, -10 \leq k \leq 9, -15 \leq l \leq 16$
Reflections collected	21294
Independent reflections	2410 [R_{int} = 0.1367, R_{sigma} = 0.0797]
Data/restraints/parameters	2410/204/193
Goodness-of-fit on F^2	1.267
Final R indexes [I>=2σ (I)]	R_1 = 0.1308, wR_2 = 0.2202
Final R indexes [all data]	R_1 = 0.1540, wR_2 = 0.2283
Largest diff. peak/hole / e Å^{-3}	0.47/-0.54

Crystals of 4a were grown from a saturated solution in CH_2Cl_2.

Crystal structure determination of 4a

Crystal Data for $C_{15}H_7BF_7N$ (M =345.03 g/mol): monoclinic, space group P2$_1$/n (no. 14), a = 11.0976(5) Å, b = 8.9576(4) Å, c = 13.9824(6) Å, β = 95.627(3)°, V = 1383.26(11) Å^3, Z = 4, T = 100.01 K, μ(CuKα) = 1.461 mm^{-1}, $Dcalc$ = 1.657 g/cm^3, 21294 reflections measured (9.724° ≤ 2Θ ≤ 133.54°), 2410 unique (R_{int} = 0.1367, R_{sigma} = 0.0797) which were used in all calculations. The final R_1 was 0.1308 (I > 2σ(I)) and wR_2 was 0.2283 (all data).

Table S8 Fractional Atomic Coordinates (×10^4) and Equivalent Isotropic Displacement Parameters (Å^2×10^3) for OJH399v_0m. U_{eq} is defined as 1/3 of of the trace of the orthogonalised U_{IJ} tensor.

Atom	*x*	*y*	*z*	U(eq)
F1	7003(3)	7916(4)	7128(3)	14.4(9)
F2	4296(4)	3994(5)	9202(3)	26.4(11)
F3	5043(4)	2365(5)	8304(3)	24.1(10)
F4	6187(4)	3462(5)	9413(3)	20.1(10)
F5	4754(4)	9083(5)	7672(3)	28.1(11)
F6	3227(4)	10289(5)	7021(3)	32.2(12)
F7	3066(4)	7963(5)	7351(3)	33.4(12)
N1	6852(5)	5939(6)	5926(4)	9.5(6)
C1	5124(6)	7610(7)	5942(5)	9.5(6)
C2	4144(6)	8558(8)	6030(5)	11.1(13)
C3	3436(7)	9091(8)	5229(5)	15.5(15)
C4	3659(6)	8658(8)	4311(5)	14.7(15)
C5	4618(6)	7694(8)	4202(5)	14.8(15)
C6	5324(6)	7204(7)	4997(5)	9.5(6)
C7	6379(6)	6200(7)	5014(5)	9.5(6)
C8	6879(7)	5507(8)	4253(5)	15.3(15)
C9	7865(7)	4599(8)	4453(5)	20.1(16)
C10	8338(7)	4367(8)	5392(5)	19.1(16)
C11	7807(6)	5051(8)	6129(5)	15.8(15)
C12	5754(6)	5742(8)	7477(5)	11.9(14)
C13	5507(6)	4856(8)	8062(5)	11.7(14)
C14	5267(6)	3679(7)	8743(5)	11.6(13)
C15	3805(6)	8989(8)	7014(5)	15.6(14)
B1	6164(7)	6888(8)	6688(5)	9.5(6)

Table S9 Anisotropic Displacement Parameters (Å^2×10^3) for OJH399v_0m. The Anisotropic displacement factor exponent takes the form: -2π^2[h^{2}a*^{2}U$_{11}$+2hka*b*U$_{12}$+…].

Atom	U_{11}	U_{22}	U_{33}	U_{23}	U_{13}	U_{12}
F1	16(2)	12(2)	16(2)	-5.6(16)	6.1(16)	-3.6(16)
F2	19(2)	32(3)	32(3)	3(2)	21(2)	4(2)
F3	37(3)	12(2)	24(2)	-2.7(18)	8(2)	-12.2(19)

Table S9 Anisotropic Displacement Parameters (Å^2×10^3) for OJH399v_0m. The Anisotropic displacement factor exponent takes the form: -2π^2[h^{2}a*^{2}U$_{11}$+2hka*b*U$_{12}$+…].

Atom	U_{11}	U_{22}	U_{33}	U_{23}	U_{13}	U_{12}
F4	18(2)	22(2)	19(2)	3.7(18)	-1.9(17)	0.0(18)
F5	20(2)	44(3)	20(2)	-13(2)	0.6(18)	6(2)
F6	38(3)	27(3)	32(3)	-10(2)	7(2)	20(2)
F7	41(3)	37(3)	25(3)	-9(2)	21(2)	-16(2)
N1	13.7(15)	4.3(14)	10.8(14)	-0.5(11)	3.5(12)	-3.2(11)
C1	13.7(15)	4.3(14)	10.8(14)	-0.5(11)	3.5(12)	-3.2(11)
C2	8(3)	8(3)	19(3)	1(2)	9(2)	-2(2)
C3	15(4)	12(4)	20(3)	3(3)	3(3)	-2(3)
C4	13(3)	18(4)	13(3)	5(3)	-1(3)	-2(3)
C5	15(4)	22(4)	8(3)	3(3)	5(3)	-2(3)
C6	13.7(15)	4.3(14)	10.8(14)	-0.5(11)	3.5(12)	-3.2(11)
C7	13.7(15)	4.3(14)	10.8(14)	-0.5(11)	3.5(12)	-3.2(11)
C8	22(4)	16(4)	7(3)	-6(3)	3(3)	-4(3)
C9	26(4)	15(4)	20(3)	-1(3)	11(3)	4(3)
C10	15(4)	18(4)	25(3)	1(3)	6(3)	3(3)
C11	16(4)	10(4)	21(4)	3(3)	3(3)	3(3)
C12	16(4)	10(3)	11(3)	-5(2)	5(3)	4(3)
C13	5(3)	12(3)	18(3)	-1(2)	1(3)	-2(3)
C14	11(3)	9(3)	16(3)	-4(2)	6(2)	-1(3)
C15	16(3)	14(3)	18(3)	-1(3)	4(3)	4(3)
B1	13.7(15)	4.3(14)	10.8(14)	-0.5(11)	3.5(12)	-3.2(11)

Table S10 Bond Lengths for OJH399v_0m.

Atom	Atom	Length/Å	Atom	Atom	Length/Å
F1	B1	1.408(8)	C2	C3	1.389(10)
F2	C14	1.337(7)	C2	C15	1.512(9)
F3	C14	1.340(8)	C3	C4	1.386(10)
F4	C14	1.331(8)	C4	C5	1.391(10)
F5	C15	1.332(8)	C5	C6	1.368(10)
F6	C15	1.330(8)	C6	C7	1.476(9)
F7	C15	1.347(8)	C7	C8	1.393(9)
N1	C7	1.351(8)	C8	C9	1.371(10)
N1	C11	1.333(9)	C9	C10	1.380(10)
N1	B1	1.613(9)	C10	C11	1.379(10)
C1	C2	1.395(9)	C12	C13	1.190(10)
C1	C6	1.409(9)	C12	B1	1.605(10)
C1	B1	1.612(10)	C13	C14	1.463(9)

Table S11 Bond Angles for OJH399v_0m.

Atom	Atom	Atom	Angle/°	Atom	Atom	Atom	Angle/°
C7	N1	B1	111.5(5)	N1	C11	C10	119.7(7)
C11	N1	C7	122.1(6)	C13	C12	B1	176.5(7)
C11	N1	B1	126.3(6)	C12	C13	C14	175.3(7)
C2	C1	C6	115.8(6)	F2	C14	F3	106.4(5)
C2	C1	B1	134.6(6)	F2	C14	C13	111.6(6)
C6	C1	B1	109.6(6)	F3	C14	C13	111.9(6)
C1	C2	C15	120.1(6)	F4	C14	F2	107.0(5)
C3	C2	C1	121.5(6)	F4	C14	F3	106.8(5)
C3	C2	C15	118.3(6)	F4	C14	C13	112.8(6)
C4	C3	C2	120.8(7)	F5	C15	F7	105.7(6)
C3	C4	C5	118.9(7)	F5	C15	C2	113.2(6)
C6	C5	C4	119.6(6)	F6	C15	F5	106.8(6)
C1	C6	C7	109.9(6)	F6	C15	F7	106.5(6)
C5	C6	C1	123.3(6)	F6	C15	C2	113.1(6)
C5	C6	C7	126.8(6)	F7	C15	C2	111.0(6)
N1	C7	C6	110.8(6)	F1	B1	N1	107.3(5)
N1	C7	C8	119.8(6)	F1	B1	C1	114.9(6)
C8	C7	C6	129.4(6)	F1	B1	C12	109.7(6)
C9	C8	C7	118.5(7)	C1	B1	N1	98.0(5)
C8	C9	C10	120.3(7)	C12	B1	N1	107.6(5)
C9	C10	C11	119.5(7)	C12	B1	C1	117.9(6)

Table S12 Torsion Angles for OJH399v_0m.

A	B	C	D	Angle/°	A	B	C	D	Angle/°
N1	C7	C8	C9	-1.5(10)	C6	C1	B1	N1	-4.3(7)
C1	C2	C3	C4	2.0(11)	C6	C1	B1	C12	-119.1(6)
C1	C2	C15	F5	32.7(9)	C6	C7	C8	C9	-179.2(7)
C1	C2	C15	F6	154.4(6)	C7	N1	C11	C10	-0.1(10)
C1	C2	C15	F7	-85.9(8)	C7	N1	B1	F1	-114.6(6)
C1	C6	C7	N1	0.5(8)	C7	N1	B1	C1	4.7(7)
C1	C6	C7	C8	178.4(7)	C7	N1	B1	C12	127.4(6)
C2	C1	C6	C5	0.1(10)	C7	C8	C9	C10	0.7(11)
C2	C1	C6	C7	-179.7(6)	C8	C9	C10	C11	0.4(11)
C2	C1	B1	F1	-67.8(10)	C9	C10	C11	N1	-0.7(11)
C2	C1	B1	N1	178.8(7)	C11	N1	C7	C6	179.3(6)
C2	C1	B1	C12	64.0(10)	C11	N1	C7	C8	1.2(10)
C2	C3	C4	C5	-0.9(11)	C11	N1	B1	F1	62.3(8)
C3	C2	C15	F5	-149.3(6)	C11	N1	B1	C1	-178.4(6)
C3	C2	C15	F6	-27.6(9)	C11	N1	B1	C12	-55.6(8)
C3	C2	C15	F7	92.1(8)	C15	C2	C3	C4	-176.0(6)
C3	C4	C5	C6	-0.5(10)	B1	N1	C7	C6	-3.6(7)

Table S12 Torsion Angles for OJH399v_0m.

A	B	C	D	Angle/°	A	B	C	D	Angle/°
C4	C5	C6	C1	0.9(11)	B1	N1	C7	C8	178.3(6)
C4	C5	C6	C7	-179.3(6)	B1	N1	C11	C10	-176.7(6)
C5	C6	C7	N1	-179.3(6)	B1	C1	C2	C3	175.2(7)
C5	C6	C7	C8	-1.4(12)	B1	C1	C2	C15	-6.8(11)
C6	C1	C2	C3	-1.6(10)	B1	C1	C6	C5	-177.4(6)
C6	C1	C2	C15	176.4(6)	B1	C1	C6	C7	2.7(7)
C6	C1	B1	F1	109.1(6)					

Table S13 Hydrogen Atom Coordinates (Å×10^{4}) and Isotropic Displacement Parameters (Å^{2}×10^{3}) for OJH399v_0m.

Atom	*x*	*y*	*z*	U(eq)
H3	2790.13	9761.18	5310.79	19
H4	3164.25	9014.4	3765.32	18
H5	4781.9	7377.44	3579.05	18
H8	6544.23	5661.55	3608.61	18
H9	8225.35	4125.25	3943.9	24
H10	9024.35	3741.9	5529.6	23
H11	8119.86	4890.38	6777.23	19

X-ray crystallographic analysis for compound 5

ORTEP of 5, thermal ellipsoids are shown at 50% probability:

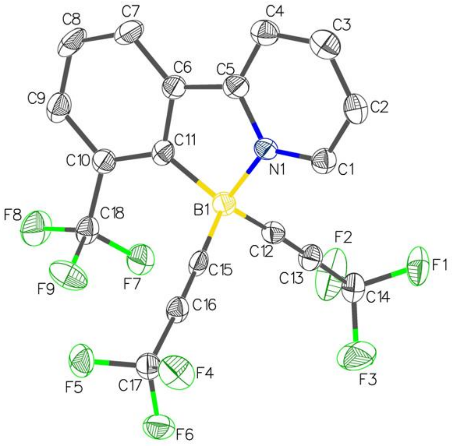

Table S14 Crystal data and structure refinement for 5.

Identification code	OJH401v_0m
Empirical formula	$C_{18}H_7BF_9N$
Formula weight	419.06
Temperature/K	100.0
Crystal system	monoclinic
Space group	$P2_1/c$
a/Å	8.7139(7)
b/Å	25.541(2)
c/Å	8.6369(7)
α/°	90
β/°	118.650(4)
γ/°	90
Volume/Å^3	1686.9(2)
Z	4
ρ_{calc}g/cm^3	1.650
μ/mm^{-1}	1.498
F(000)	832.0
Crystal size/mm^3	0.388 × 0.208 × 0.08
Radiation	CuKα (λ = 1.54178)
2Θ range for data collection/°	6.922 to 134.36
Index ranges	$-7 \leq h \leq 10, -30 \leq k \leq 30, -10 \leq l \leq 9$
Reflections collected	8121
Independent reflections	2925 [R_{int} = 0.0532, R_{sigma} = 0.0553]
Data/restraints/parameters	2925/0/264
Goodness-of-fit on F^2	1.077
Final R indexes [I>=2σ (I)]	R_1 = 0.0715, wR_2 = 0.2040
Final R indexes [all data]	R_1 = 0.0791, wR_2 = 0.2227
Largest diff. peak/hole / e Å^{-3}	0.50/-0.46

Crystals of 5 were grown from a saturated solution in acetone

Crystal structure determination of 5

Crystal Data for $C_{18}H_7BF_9N$ (M =419.06 g/mol): monoclinic, space group P2$_1$/c (no. 14), a = 8.7139(7) Å, b = 25.541(2) Å, c = 8.6369(7) Å, β = 118.650(4)°, V = 1686.9(2) Å^3, Z = 4, T = 100.0 K, μ(CuKα) = 1.498 mm^{-1}, $Dcalc$ = 1.650 g/cm^3, 8121 reflections measured (6.922° ≤ 2Θ ≤ 134.36°), 2925 unique (R_{int} = 0.0532, R_{sigma} = 0.0553) which were used in all calculations. The final R_1 was 0.0715 (I > 2σ(I)) and wR_2 was 0.2227 (all data).

Table S15 Fractional Atomic Coordinates (×10^4) and Equivalent Isotropic Displacement Parameters (Å^2×10^3) for OJH401v_0m. U_{eq} is defined as 1/3 of of the trace of the orthogonalised U_{IJ} tensor.

Atom	*x*	*y*	*z*	U(eq)
F1	2350(5)	5126.6(12)	5973(4)	41.4(8)
F2	3395(7)	5819.9(14)	7472(5)	57.3(12)
F3	5085(5)	5252.0(17)	7263(5)	58.1(11)
F4	7975(5)	5677.6(14)	657(5)	42.2(8)
F5	8671(4)	6460.4(12)	1610(5)	40.4(8)
F6	9266(4)	5833.8(13)	3455(4)	37.6(8)
F7	5301(4)	6937.0(13)	5421(5)	41.2(8)
F8	5723(5)	7753.0(13)	5245(5)	46.0(9)
F9	6591(4)	7192.1(15)	4002(4)	41.7(8)
N1	1652(5)	6067.7(15)	139(5)	21.7(8)
C1	1341(6)	5571.5(18)	-409(7)	25.0(10)
C2	-148(7)	5439.1(19)	-1984(7)	28.3(10)
C3	-1285(7)	5838(2)	-2952(7)	33.6(11)
C4	-944(7)	6350(2)	-2381(7)	29.6(11)
C5	554(6)	6459.1(19)	-786(7)	26.2(10)
C6	1189(6)	6958.7(18)	99(7)	24.6(10)
C7	417(7)	7449(2)	-493(7)	31.7(11)
C8	1216(8)	7882.9(19)	503(8)	34.9(12)
C9	2801(7)	7835.8(19)	2097(8)	31.8(12)
C10	3554(6)	7345.3(18)	2655(7)	24.7(10)
C11	2764(6)	6893.7(18)	1684(6)	22.9(10)
C12	3326(6)	5987.4(17)	3560(6)	23.4(10)
C13	3416(7)	5757.5(17)	4813(7)	25.0(10)
C14	3552(7)	5495.7(19)	6360(7)	30.0(11)
C15	5019(6)	6160.6(16)	1828(7)	22.7(10)
C16	6372(7)	6082.4(17)	1815(7)	24.0(10)
C17	8043(7)	6017.8(19)	1873(7)	26.1(10)
C18	5274(7)	7309.5(18)	4328(7)	25.4(10)
B1	3298(7)	6278(2)	1929(7)	21.8(11)

Table S16 Anisotropic Displacement Parameters (Å^2×10^3) for OJH401v_0m. The Anisotropic displacement factor exponent takes the form: -2π^2[h^{2}a*^{2}U$_{11}$+2hka*b*U$_{12}$+…].

Atom	U_{11}	U_{22}	U_{33}	U_{23}	U_{13}	U_{12}
F1	56(2)	31.6(16)	39.8(18)	1.8(14)	25.7(16)	-16.6(15)
F2	112(4)	36.1(18)	47(2)	-9.5(17)	57(2)	-15(2)
F3	45(2)	74(3)	48(2)	36.2(19)	16.8(17)	12.5(18)
F4	40.9(18)	47.0(18)	47(2)	-17.8(15)	27.9(15)	0.4(15)
F5	39.6(18)	33.0(16)	62(2)	6.8(14)	35.1(17)	-2.8(13)
F6	25.2(15)	43.8(17)	43.4(19)	11.2(14)	16.1(13)	6.6(13)
F7	36.8(18)	39.3(17)	42.9(19)	10.2(14)	15.4(14)	-4.6(13)
F8	54(2)	27.0(15)	49(2)	-13.5(14)	17.5(16)	-0.6(14)
F9	25.5(16)	65(2)	39.1(18)	-6.6(16)	19.1(13)	0.9(15)
N1	17.5(18)	23.5(18)	26(2)	2.0(15)	12.0(15)	1.6(15)
C1	26(2)	23(2)	29(2)	-0.8(18)	16(2)	1.5(18)
C2	31(3)	28(2)	30(3)	-3(2)	18(2)	-6(2)
C3	31(3)	41(3)	31(3)	-3(2)	16(2)	-2(2)
C4	26(2)	35(3)	26(2)	3(2)	12(2)	1(2)
C5	24(2)	28(2)	33(3)	5(2)	19(2)	4.7(19)
C6	23(2)	23(2)	33(3)	3.7(19)	18(2)	3.1(18)
C7	33(3)	28(2)	37(3)	12(2)	19(2)	12(2)
C8	46(3)	19(2)	50(3)	10(2)	31(3)	14(2)
C9	38(3)	21(2)	45(3)	0(2)	26(3)	1(2)
C10	24(2)	24(2)	33(3)	3.0(19)	18(2)	2.0(18)
C11	26(2)	23(2)	28(2)	5.8(19)	20(2)	1.8(18)
C12	19(2)	21(2)	30(2)	-0.5(19)	11.5(18)	0.9(17)
C13	28(2)	22(2)	26(2)	-1.0(19)	14.1(19)	-0.3(17)
C14	34(3)	24(2)	34(3)	1(2)	18(2)	-4(2)
C15	29(3)	12.5(18)	31(2)	1.4(17)	17(2)	0.3(17)
C16	27(2)	18(2)	33(3)	-1.6(17)	20(2)	0.4(17)
C17	26(2)	24(2)	32(3)	-2.0(19)	18(2)	0.3(19)
C18	32(3)	18(2)	34(3)	-2.7(19)	22(2)	0.5(18)
B1	21(3)	21(2)	25(3)	-1(2)	12(2)	0.1(19)

Table S17 Bond Lengths for OJH401v_0m.

Atom	Atom	Length/Å	Atom	Atom	Length/Å
F1	C14	1.328(6)	C5	C6	1.453(7)
F2	C14	1.324(7)	C6	C7	1.397(7)
F3	C14	1.334(6)	C6	C11	1.408(7)
F4	C17	1.342(6)	C7	C8	1.371(8)
F5	C17	1.322(6)	C8	C9	1.413(9)
F6	C17	1.352(6)	C9	C10	1.389(7)
F7	C18	1.333(6)	C10	C11	1.398(7)

Table S17 Bond Lengths for OJH401v_0m.

Atom	Atom	Length/Å	Atom	Atom	Length/Å
F8	C18	1.329(6)	C10	C18	1.506(7)
F9	C18	1.340(6)	C11	B1	1.626(7)
N1	C1	1.334(6)	C12	C13	1.200(7)
N1	C5	1.349(6)	C12	B1	1.582(7)
N1	B1	1.615(7)	C13	C14	1.447(7)
C1	C2	1.398(7)	C15	C16	1.201(7)
C2	C3	1.388(8)	C15	B1	1.572(7)
C3	C4	1.378(8)	C16	C17	1.443(7)
C4	C5	1.399(7)			

Table S18 Bond Angles for OJH401v_0m.

Atom	Atom	Atom	Angle/˚	Atom	Atom	Atom	Angle/˚
C1	N1	C5	121.9(4)	F1	C14	C13	113.0(4)
C1	N1	B1	126.2(4)	F2	C14	F1	106.4(4)
C5	N1	B1	111.9(4)	F2	C14	F3	106.9(5)
N1	C1	C2	120.7(4)	F2	C14	C13	112.9(4)
C3	C2	C1	118.0(5)	F3	C14	C13	111.8(4)
C4	C3	C2	120.8(5)	C16	C15	B1	177.3(5)
C3	C4	C5	118.7(5)	C15	C16	C17	176.3(5)
N1	C5	C4	119.9(5)	F4	C17	F6	106.5(4)
N1	C5	C6	110.9(4)	F4	C17	C16	112.4(4)
C4	C5	C6	129.3(5)	F5	C17	F4	106.6(4)
C7	C6	C5	126.8(5)	F5	C17	F6	106.3(4)
C7	C6	C11	122.4(5)	F5	C17	C16	113.0(4)
C11	C6	C5	110.8(4)	F6	C17	C16	111.6(4)
C8	C7	C6	119.0(5)	F7	C18	F9	105.2(4)
C7	C8	C9	120.5(5)	F7	C18	C10	113.2(4)
C10	C9	C8	119.6(5)	F8	C18	F7	106.6(4)
C9	C10	C11	121.4(5)	F8	C18	F9	106.2(4)
C9	C10	C18	118.3(4)	F8	C18	C10	113.2(4)
C11	C10	C18	120.2(4)	F9	C18	C10	111.8(4)
C6	C11	B1	109.0(4)	N1	B1	C11	97.4(4)
C10	C11	C6	117.1(4)	C12	B1	N1	108.9(4)
C10	C11	B1	133.8(5)	C12	B1	C11	116.8(4)
C13	C12	B1	177.1(5)	C15	B1	N1	108.5(4)
C12	C13	C14	178.1(5)	C15	B1	C11	113.5(4)
F1	C14	F3	105.3(4)	C15	B1	C12	110.7(4)

Table S19 Torsion Angles for OJH401v_0m.

A	B	C	D	Angle/°	A	B	C	D	Angle/°
N1	C1	C2	C3	0.0(7)	C6	C11	B1	C15	113.7(5)
N1	C5	C6	C7	178.3(5)	C7	C6	C11	C10	-0.7(7)
N1	C5	C6	C11	-0.6(6)	C7	C6	C11	B1	-178.6(4)
C1	N1	C5	C4	-1.1(7)	C7	C8	C9	C10	0.1(8)
C1	N1	C5	C6	179.8(4)	C8	C9	C10	C11	-0.9(7)
C1	N1	B1	C11	-179.4(4)	C8	C9	C10	C18	178.0(5)
C1	N1	B1	C12	-57.8(6)	C9	C10	C11	C6	1.2(7)
C1	N1	B1	C15	62.7(6)	C9	C10	C11	B1	178.4(5)
C1	C2	C3	C4	0.6(8)	C9	C10	C18	F7	132.6(5)
C2	C3	C4	C5	-1.3(8)	C9	C10	C18	F8	11.1(7)
C3	C4	C5	N1	1.6(7)	C9	C10	C18	F9	-108.7(5)
C3	C4	C5	C6	-179.5(5)	C10	C11	B1	N1	-177.5(5)
C4	C5	C6	C7	-0.7(8)	C10	C11	B1	C12	67.0(7)
C4	C5	C6	C11	-179.7(5)	C10	C11	B1	C15	-63.6(7)
C5	N1	C1	C2	0.3(7)	C11	C6	C7	C8	-0.1(8)
C5	N1	B1	C11	-0.2(5)	C11	C10	C18	F7	-48.5(6)
C5	N1	B1	C12	121.4(4)	C11	C10	C18	F8	-170.0(4)
C5	N1	B1	C15	-118.1(4)	C11	C10	C18	F9	70.2(6)
C5	C6	C7	C8	-178.9(5)	C18	C10	C11	C6	-177.7(4)
C5	C6	C11	C10	178.3(4)	C18	C10	C11	B1	-0.5(8)
C5	C6	C11	B1	0.5(5)	B1	N1	C1	C2	179.4(4)
C6	C7	C8	C9	0.4(8)	B1	N1	C5	C4	179.7(4)
C6	C11	B1	N1	-0.2(5)	B1	N1	C5	C6	0.5(5)
C6	C11	B1	C12	-115.7(5)					

Table S20 Hydrogen Atom Coordinates (Å×10^4) and Isotropic Displacement Parameters (Å^2×10^3) for OJH401v_0m.

Atom	*x*	*y*	*z*	U(eq)
H1	2139.27	5305.33	278.2	30
H2	-374.28	5086.37	-2379.12	34
H3	-2311.21	5757.84	-4021.42	40
H4	-1711.61	6623.75	-3058.87	36
H7	-645.7	7481.01	-1569.11	38
H8	698.67	8218.03	117.52	42
H9	3349.52	8137.73	2783.4	38

X-ray crystallographic analysis for compound 9

ORTEP of 9, thermal ellipsoids are shown at 50% probability:

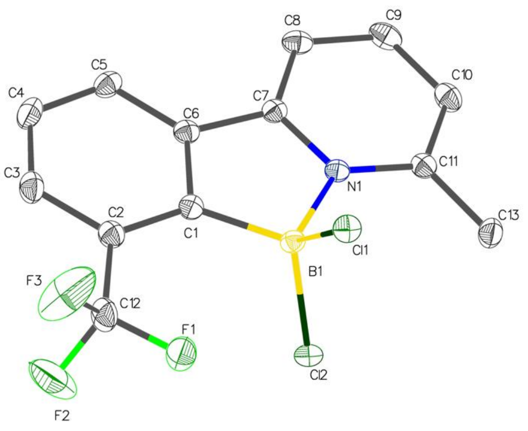

Table S21 Crystal data and structure refinement for 9.

Identification code	ojh398s_100k
Empirical formula	$C_{13}H_9BCl_2F_3N$
Formula weight	317.92
Temperature/K	100
Crystal system	monoclinic
Space group	$P2_1/c$
a/Å	7.0207(4)
b/Å	11.6007(7)
c/Å	16.1032(10)
α/°	90
β/°	92.0694(14)
γ/°	90
Volume/Å^3	1310.67(14)
Z	4
ρ_{calc}g/cm^3	1.611
μ/mm^{-1}	0.516
F(000)	640.0
Crystal size/mm^3	0.541 × 0.214 × 0.141
Radiation	MoKα (λ = 0.71073)
2Θ range for data collection/°	4.328 to 57.62
Index ranges	$-9 \leq h \leq 9, 0 \leq k \leq 15, 0 \leq l \leq 21$
Reflections collected	3506
Independent reflections	3506 [R_{int} = ?, R_{sigma} = 0.0148]
Data/restraints/parameters	3506/0/183
Goodness-of-fit on F^2	1.057
Final R indexes [I>=2σ (I)]	R_1 = 0.0274, wR_2 = 0.0711
Final R indexes [all data]	R_1 = 0.0301, wR_2 = 0.0728
Largest diff. peak/hole / e Å^{-3}	0.50/-0.35

Crystals of 9 were grown from a saturated solution in acetone

Crystal structure determination of 9

Crystal Data for $C_{13}H_9BCl_2F_3N$ (M =317.92 g/mol): monoclinic, space group P2$_1$/c (no. 14), a = 7.0207(4) Å, b = 11.6007(7) Å, c = 16.1032(10) Å, β = 92.0694(14)°, V = 1310.67(14) Å^3, Z = 4, T = 100 K, μ(MoKα) = 0.516 mm^{-1}, $Dcalc$ = 1.611 g/cm^3, 3506 reflections measured (4.328° ≤ 2Θ ≤ 57.62°), 3506 unique (R_{int} = ?, R_{sigma} = 0.0148) which were used in all calculations. The final R_1 was 0.0274 (I > 2σ(I)) and wR_2 was 0.0728 (all data).

Table S22 Fractional Atomic Coordinates (×10^4) and Equivalent Isotropic Displacement Parameters (Å^2×10^3) for ojh398s_100k. U_{eq} is defined as 1/3 of the trace of the orthogonalised U_{IJ} tensor.

Atom	*x*	*y*	*z*	U(eq)
Cl1	4776.1(5)	1208.2(3)	2113.1(2)	16.29(9)
Cl2	385.4(5)	1295.1(3)	2063.7(2)	16.25(9)
F1	2722(2)	3519.5(8)	1696.9(6)	30.0(2)
F2	994(3)	4405.2(15)	791.5(12)	66.3(6)
F3	4035(3)	4508.9(14)	765.5(11)	68.7(6)
N1	2474.5(18)	-326.0(10)	1177.9(7)	12.5(2)
C11	2401(2)	-1287.3(12)	1656.6(9)	14.7(3)
C10	2363(2)	-2360.7(13)	1266.7(10)	18.0(3)
C9	2393(3)	-2443.6(13)	408.1(10)	18.8(3)
C8	2472(2)	-1448.4(13)	-69.3(9)	16.7(3)
C7	2499(2)	-392.8(12)	331.1(8)	13.4(3)
C6	2559(2)	765.8(12)	-35.5(9)	14.0(3)
C5	2591(2)	1010.7(13)	-881.7(9)	17.2(3)
C4	2642(2)	2159.6(15)	-1126.3(9)	20.6(3)
C3	2655(2)	3026.8(14)	-532.8(9)	19.8(3)
C2	2619(2)	2764.7(13)	316.5(9)	16.9(3)
C1	2574(2)	1618.4(12)	585.1(8)	14.0(3)
C13	2343(2)	-1186.2(13)	2581.5(9)	20.3(3)
C12	2598(3)	3781.7(13)	898.0(10)	23.9(3)
B1	2542(2)	990.7(13)	1471.6(9)	13.3(3)

Table S23 Anisotropic Displacement Parameters (Å^2×10^3) for ojh398s_100k. The Anisotropic displacement factor exponent takes the form: $-2\pi^2[h^2a^{*2}U_{11}+2hka^*b^*U_{12}+\ldots]$.

Atom	U_{11}	U_{22}	U_{33}	U_{23}	U_{13}	U_{12}
Cl1	14.79(16)	17.53(17)	16.37(17)	-2.22(13)	-1.96(12)	-2.11(15)
Cl2	15.19(16)	17.49(17)	16.27(17)	-2.48(13)	3.42(12)	1.66(15)
F1	53.6(7)	16.3(4)	20.1(5)	-1.9(4)	2.4(5)	-0.9(5)
F2	93.2(13)	56.2(10)	47.2(9)	-24.0(8)	-28.2(8)	52.2(9)
F3	115.1(15)	44.6(9)	48.6(10)	-18.7(7)	36.0(9)	-56.3(9)

Table S23 Anisotropic Displacement Parameters (Å^2×10^3) for ojh398s_100k. The Anisotropic displacement factor exponent takes the form: -2π^2[h^{2}a*^{2}U$_{11}$+2hka*b*U$_{12}$+…].

Atom	U11	U22	U33	U23	U13	U12
N1	10.8(5)	13.1(5)	13.6(5)	-1.0(4)	0.5(5)	0.0(5)
C11	12.7(6)	13.9(6)	17.6(6)	0.3(5)	1.4(5)	-0.4(6)
C10	17.2(7)	13.1(6)	23.8(7)	1.4(5)	0.6(6)	-0.4(6)
C9	16.2(7)	14.3(6)	25.6(7)	-5.8(6)	-2.1(6)	-0.2(6)
C8	14.5(6)	18.0(7)	17.5(6)	-5.1(5)	0.0(5)	0.8(6)
C7	9.9(6)	16.5(6)	13.9(6)	-0.4(5)	0.0(5)	0.3(6)
C6	10.8(6)	16.1(6)	14.9(6)	-0.8(5)	0.1(5)	0.5(5)
C5	15.1(7)	23.1(7)	13.5(6)	-1.2(5)	0.3(5)	-0.4(6)
C4	18.5(7)	28.9(8)	14.3(6)	5.8(6)	1.0(6)	-0.5(7)
C3	20.0(7)	18.6(7)	20.9(7)	6.3(6)	1.5(6)	-0.7(6)
C2	17.2(7)	15.2(6)	18.2(6)	0.7(5)	0.6(6)	-0.1(6)
C1	12.0(6)	15.5(6)	14.6(6)	0.4(5)	0.0(5)	-0.3(5)
C13	29.9(8)	15.7(7)	15.5(7)	2.5(5)	2.2(6)	0.0(7)
C12	35.9(9)	12.9(6)	23.0(7)	2.6(6)	1.7(7)	-2.0(7)
B1	14.5(7)	12.6(7)	12.8(6)	-1.5(5)	1.9(6)	-1.0(6)

Table S24 Bond Lengths for ojh398s_100k.

Atom	Atom	Length/Å	Atom	Atom	Length/Å
Cl1	B1	1.8641(17)	C9	C8	1.389(2)
Cl2	B1	1.8519(17)	C8	C7	1.3837(19)
F1	C12	1.3218(18)	C7	C6	1.469(2)
F2	C12	1.344(2)	C6	C5	1.3929(19)
F3	C12	1.338(2)	C6	C1	1.4058(19)
N1	C11	1.3577(18)	C5	C4	1.391(2)
N1	C7	1.3666(17)	C4	C3	1.387(2)
N1	B1	1.5992(18)	C3	C2	1.402(2)
C11	C10	1.394(2)	C2	C1	1.399(2)
C11	C13	1.496(2)	C2	C12	1.507(2)
C10	C9	1.387(2)	C1	B1	1.603(2)

Table S25 Bond Angles for ojh398s_100k.

Atom	Atom	Atom	Angle/°	Atom	Atom	Atom	Angle/°
C11	N1	C7	121.48(12)	C3	C2	C12	115.93(13)
C11	N1	B1	128.16(11)	C1	C2	C3	120.60(14)
C7	N1	B1	110.36(11)	C1	C2	C12	123.46(13)
N1	C11	C10	118.57(13)	C6	C1	B1	108.26(12)
N1	C11	C13	120.26(12)	C2	C1	C6	116.66(13)
C10	C11	C13	121.17(13)	C2	C1	B1	135.08(13)

Table S25 Bond Angles for ojh398s_100k.

Atom	Atom	Atom	Angle/°	Atom	Atom	Atom	Angle/°
C9	C10	C11	120.67(14)	F1	C12	F2	105.86(16)
C10	C9	C8	119.76(13)	F1	C12	F3	106.07(16)
C7	C8	C9	118.54(13)	F1	C12	C2	115.02(13)
N1	C7	C8	120.97(13)	F2	C12	C2	111.65(15)
N1	C7	C6	110.52(12)	F3	C12	F2	105.87(16)
C8	C7	C6	128.50(13)	F3	C12	C2	111.73(15)
C5	C6	C7	125.54(13)	Cl2	B1	Cl1	112.09(8)
C5	C6	C1	123.50(13)	N1	B1	Cl1	107.96(10)
C1	C6	C7	110.96(12)	N1	B1	Cl2	108.57(10)
C4	C5	C6	118.30(13)	N1	B1	C1	99.89(10)
C3	C4	C5	119.96(13)	C1	B1	Cl1	113.16(11)
C4	C3	C2	120.98(14)	C1	B1	Cl2	114.20(11)

Table S26 Torsion Angles for ojh398s_100k.

A	B	C	D	Angle/°	A	B	C	D	Angle/°
N1	C11	C10	C9	-0.2(2)	C6	C1	B1	N1	-0.88(16)
N1	C7	C6	C5	-179.77(14)	C5	C6	C1	C2	0.1(2)
N1	C7	C6	C1	-0.03(18)	C5	C6	C1	B1	-179.63(14)
C11	N1	C7	C8	-0.7(2)	C5	C4	C3	C2	0.1(3)
C11	N1	C7	C6	179.38(13)	C4	C3	C2	C1	0.2(3)
C11	N1	B1	Cl1	62.48(17)	C4	C3	C2	C12	-178.83(15)
C11	N1	B1	Cl2	-59.25(17)	C3	C2	C1	C6	-0.2(2)
C11	N1	B1	C1	-179.08(14)	C3	C2	C1	B1	179.40(16)
C11	C10	C9	C8	0.3(3)	C3	C2	C12	F1	-174.70(16)
C10	C9	C8	C7	-0.6(2)	C3	C2	C12	F2	64.6(2)
C9	C8	C7	N1	0.8(2)	C3	C2	C12	F3	-53.7(2)
C9	C8	C7	C6	-179.31(17)	C2	C1	B1	Cl1	-66.0(2)
C8	C7	C6	C5	0.3(3)	C2	C1	B1	Cl2	63.8(2)
C8	C7	C6	C1	-179.91(15)	C2	C1	B1	N1	179.47(18)
C7	N1	C11	C10	0.4(2)	C1	C6	C5	C4	0.1(2)
C7	N1	C11	C13	-179.00(14)	C1	C2	C12	F1	6.3(3)
C7	N1	B1	Cl1	-117.56(11)	C1	C2	C12	F2	-114.3(2)
C7	N1	B1	Cl2	120.71(11)	C1	C2	C12	F3	127.34(19)
C7	N1	B1	C1	0.88(15)	C13	C11	C10	C9	179.19(15)
C7	C6	C5	C4	179.83(15)	C12	C2	C1	C6	178.67(15)

Table S26 Torsion Angles for ojh398s_100k.

A	B	C	D	Angle/°	A	B	C	D	Angle/°
C7	C6	C1	C2	-179.65(13)	C12	C2	C1	B1	-1.7(3)
C7	C6	C1	B1	0.62(18)	B1	N1	C11	C10	-179.64(15)
C6	C5	C4	C3	-0.2(3)	B1	N1	C11	C13	1.0(2)
C6	C1	B1	Cl1	113.65(12)	B1	N1	C7	C8	179.31(14)
C6	C1	B1	Cl2	-116.52(12)	B1	N1	C7	C6	-0.59(17)

Table S27 Hydrogen Atom Coordinates (Å×10^4) and Isotropic Displacement Parameters (Å^2×10^3) for ojh398s_100k.

Atom	*x*	*y*	*z*	U(eq)
H10	2316.41	-3027.79	1585.57	22
H9	2360.92	-3162.79	152.91	23
H8	2504.9	-1490.72	-645.31	20
H5	2577.4	420.95	-1273.04	21
H4	2668.2	2346.49	-1687.39	25
H3	2688.16	3793.32	-700.84	24
H13A	3284.99	-639.97	2775.95	30
H13B	2605.38	-1924.17	2829.99	30
H13C	1102.57	-929.44	2732.14	30
